# Organocatalytic DYKAT of *Si*-Stereogenic Silanes

**DOI:** 10.1021/jacs.3c00858

**Published:** 2023-02-24

**Authors:** Hui Zhou, Roberta Properzi, Markus Leutzsch, Paola Belanzoni, Giovanni Bistoni, Nobuya Tsuji, Jung Tae Han, Chendan Zhu, Benjamin List

**Affiliations:** † Max-Planck-Institut für Kohlenforschung, Kaiser-Wilhelm-Platz 1, 45470 Mülheim an der Ruhr, Germany; ‡ University of Perugia, Department of Chemistry, Biology and Biotechnology, 06122 Perugia, Italy; § Institute for Chemical Reaction Design and Discovery (WPI-ICReDD), Hokkaido University, Sapporo 001-0021, Japan

## Abstract

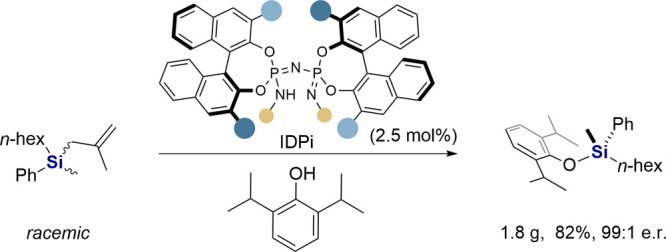

Chiral organosilanes do not exist in nature and are therefore absent from the “chiral pool”. As a consequence, synthetic approaches toward enantiopure silanes, stereogenic at silicon, are rather limited. While catalytic asymmetric desymmetrization reactions of symmetric organosilicon compounds have been developed, the utilization of *racemic* silanes in a dynamic kinetic asymmetric transformation (DYKAT) or dynamic kinetic resolution (DKR) would significantly expand the breadth of accessible *Si*-stereogenic compounds. We now report a DYKAT of racemic allyl silanes enabled by strong and confined imidodiphosphorimidate (IDPi) catalysts, providing access to *Si*-stereogenic silyl ethers. The products of this reaction are easily converted into useful enantiopure monohydrosilanes. We propose a spectroscopically and experimentally supported mechanism involving the epimerization of a catalyst-bound intermediate.

In light of their importance in chemical synthesis and technical materials, enantiopure silicon-containing compounds bearing a *Si*-stereogenic center have recently gained considerable attention.^1−10^ However, while *C*-stereogenic congeners are readily accessible via a plethora of asymmetric transformations of C=X π-bonds (X = C, O, N), the synthesis of silicon-stereogenic silanes has been notoriously difficult due to the instability and therefore unavailability of the corresponding Si=X-based molecules.^11,12^ Documented enantioselective approaches to *Si*-stereogenic organosilanes can be divided into four main categories: chromatographic resolution,^13^ chemical resolution with a stoichiometric chiral reagent,^14^ catalytic desymmetrization of symmetric silicon compounds,^3−5,7,15−17^ and catalytic kinetic resolutions (eq 1).^18−21^ We became interested in contributing potentially more general and higher-yielding synthetic strategies toward enantiopure organosilanes. Specifically, we anticipated to expand the synthetic toolbox by efficiently utilizing racemic silanes, either in a dynamic kinetic resolution (DKR) or a dynamic kinetic asymmetric transformation (DYKAT), which both have the inherent advantage of offering yields >50%. A rhodium-catalyzed dynamic kinetic asymmetric hydrosilylation has recently been described by Xu and co-workers, but organocatalysis has not previously been used in a DYKAT toward stereogenic silanes.^22^
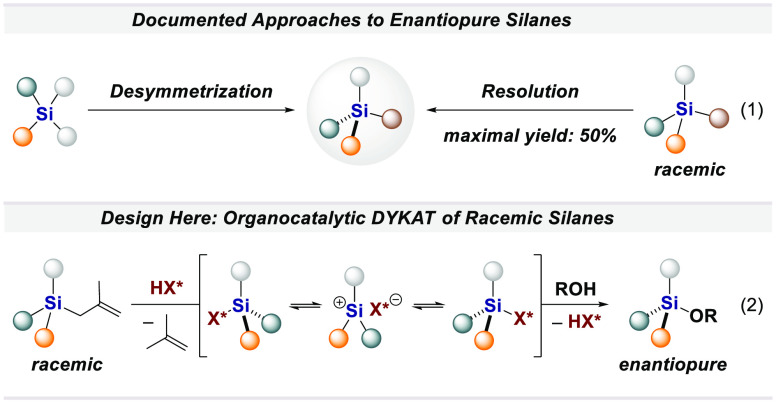
1

However, since preservation of stereochemical integrity is frequently observed in organosilicon chemistry, a challenge to overcome in such strategies is the required racemization of the *Si*-stereogenic center during the reaction conditions.^23−26^ Inspiration came from previous work on the desymmetrization of symmetrical silanes via a silicon hydrogen exchange reaction and general studies on silylium-based asymmetric counteranion-directed catalysis (*Si*-ACDC).^17,27−50^ Accordingly, we focused our attention on the design of a strong and confined imidodiphosphorimidate (IDPi)-catalyzed asymmetric synthesis of *Si*-stereogenic silyl ethers from racemic allyl silanes (eq 2). We anticipated that this approach could benefit from the configurational lability of the silylated IDPi intermediate, enabling high enantioselectivity and full conversion of the racemic starting material. Here we report the fruition of these considerations with an IDPi-catalyzed DYKAT of racemic allyl silanes with phenols, providing *Si*-stereogenic silyl ethers in excellent yield and enantioselectivity.

After identifying methallyl silane **1a** and propofol (**2**) as suitable reactants in an initial screening (see Tables S1–S3 in the Supporting Information), we began optimizing this model reaction as depicted in Table 1.^51^ First, different solvents were examined with IDPi catalyst **3a** (entries 1–4), and toluene was identified as a preferred solvent, for example, compared to methylcyclohexane, in which scarce solubility of the IDPi catalyst was observed. We also explored variations of the aryl group on the catalyst. Indeed, IDPi catalyst **3b**, bearing *p*-*tert*-butyl phenyl groups at the 3,3′-positions of the binaphthyl backbone and triflyl groups at the inner core, enabled the formation of the desired product in 94:6 e.r. (entry 5). Excitingly, the e.r. of product **4a** could be further improved to 99:1 by evolving the inner core (entries 6 and 7). It is noteworthy that increasing the reaction concentration suppressed the formation of side products, leading to satisfactory yields with no significant effect on the enantioselectivity (entries 8 and 9).

**Table 1:**
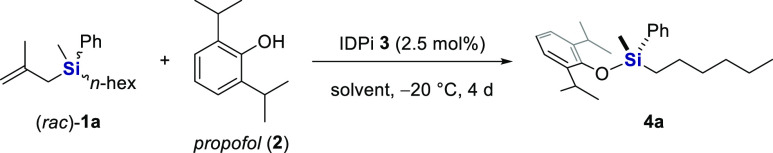

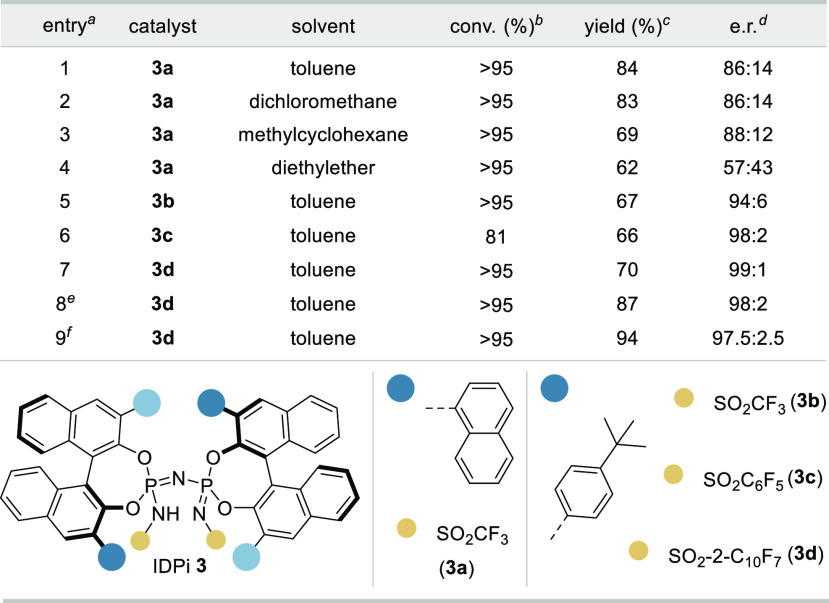
Reaction Development

a Unless stated otherwise, reactions were performed with *rac*-**1a** (0.025 mmol), propofol **2** (1.5 equiv), and IDPi catalysts **3a**–**d** (2.5 mol %) in solvent (0.25 mL, 0.1 M).

b Estimated by ^1^H NMR or TLC analysis.

c Determined by ^1^H NMR using CH_2_Br_2_ as internal standard.

d Enantiomeric ratios (e.r.) determined by HPLC.

e 0.2 M.

f 0.4 M.

Under the optimized conditions, the scope and generality of the IDPi-catalyzed DYKAT of silanes was evaluated and the results are outlined in Table 2. Chiral silyl ether **4a** was obtained in 70% yield with 99:1 e.r. when the reaction of *rac*-**1a** and phenol **2** was conducted on a 0.2 mmol scale. Similarly, racemic silanes bearing different linear alkyl chains could all be accommodated to provide the corresponding products **4b**–**f** with good to excellent results (67–84% yields and 85:15 to 99:1 e.r.). Additionally, a branched-alkyl-substituted silane was also efficiently converted, furnishing the corresponding product **4g** in 95% yield and 98:2 e.r.. We found that high levels of enantioinduction were maintained when silane starting materials bearing substituents with diverse electronic properties in *para* and *meta* positions on the phenyl group were subjected to the reaction conditions. Organosilanes **4h**–**l** were readily obtained with excellent results (77–87% yields and 96.5:3.5 to 99:1 e.r.). In contrast, a moderate e.r. was obtained from the reaction of silane **1m**, which bears an *ortho* substituent. 2-Naphthyl and thienyl moieties were compatible with the DYKAT process, affording products **4n**,**o** in good yields and excellent enantioselectivities. In addition to being competent at the synthesis of organosilanes bearing saturated alkyl chains, a range of racemic silanes featuring alkenyl groups were tolerated equally well, enabling access to products **4p**–**r** in 61–76% yield and 86:14 to 97:3 e.r. However, the enantiodifferentiation was less effective in the presence of a benzyl-substituted racemic silane, and product **4s** was obtained in 70% yield and 75:25 e.r. It is noteworthy that the reactivity of the starting silanes is highly dependent on the nature of the allyl leaving groups; in fact, the lack of reactivity of internal alkenyl and simple allyl moieties correlates to their nucleophilicity.^52^ Moreover, we were curious to investigate substrates with an existing carbon stereocenter in the deracemization reaction with propofol. The transformation of a 1:1 diastereomeric mixture of the enantiopure starting material **1v** proved to be exquisitely catalyst controlled, and the two enantiomers of IDPi **3d** gave products **4t**,**u** with 18:1 and >20:1 d.r., respectively. Furthermore, our reaction is readily scalable and a preparative synthesis of *Si*-stereogenic silyl ether **4a** (1.8 g) was accomplished in 82% isolated yield without erosion of enantioselectivity and the catalyst was recovered in 88% yield. More importantly, monohydrosilane **5** could easily be obtained without significant loss of enantiopurity by the reductive removal of the phenolic unit with retention of *Si*-stereochemistry.^53^ The described two-step approach here may facilitate the wide utilization of such hydrosilanes as chiral auxiliaries, protecting groups, reagents, and synthetic precursors.^54^

**Table 2:**
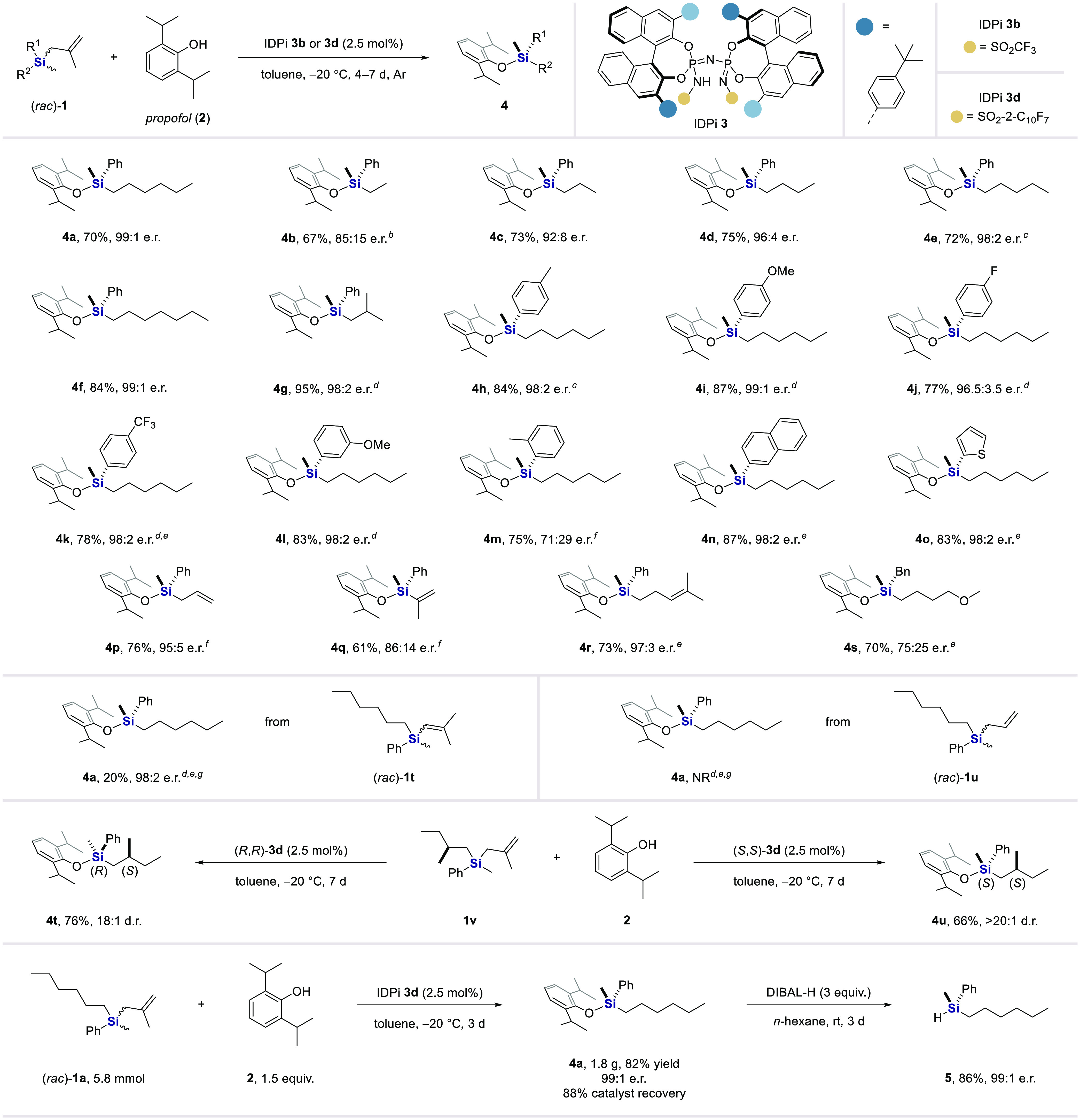
Substrate Scope^a^

a Unless specified otherwise, reactions were performed with *rac*-**1** (0.2 mmol, 1.0 equiv), 2,6-diisopropylphenol **2** (0.3 mmol, 1.5 equiv), and IDPi **3d** (2.5 mol %) in toluene (0.1 M) at −20 °C for 4–7 days. Isolated yields are given, and enantiomeric ratios (e.r.) were determined by HPLC. Conditions of reactions starting from a silicon compound bearing a *C*-stereocenter: **1v** (0.1 mmol, 1.0 equiv), **2** (0.15 mmol, 1.5 equiv), and IDPi **3d** (2.5 mol %) in toluene (0.1 M) at −20 °C for 7 days. Conditions for gram-scale synthesis and product elaboration: *rac*-**1** (5.8 mmol, 1.0 equiv), **2** (8.7 mmol, 1.5 equiv), and IDPi **3d** (2.5 mol %) in toluene (0.1 M) at −20 °C for 3 days; **4a** (0.3 mmol) and DIBAL-H (3 equiv) in hexanes (0.15 M) at rt for 3 days.

b –40 °C.

c 0.4 M.

d 0.2 M.

e rt.

f With IDPi **3b** as catalyst.

g With 5 mol % catalyst.

To shed light on the underlying reaction mechanism of the discovered DYKAT, we conducted a time-course study for the reaction of allyl silane **1a** with propofol **2** catalyzed by IDPi **3d** in toluene at −20 °C for 3 days. The enantiopurity of the remaining silane **1a** and product **4a** was continuously monitored during the course of the reaction (Figure 1A). Interestingly, the IDPi catalyst enables nearly complete control of the enantioselectivity of product **4a** from the starting point of the reaction, while the remaining silane **1a** undergoes a slow enantioenrichment throughout the course of the reaction, suggesting a kinetic resolution process. Meanwhile, racemization studies under IDPi catalysis were performed with enantiopure starting materials (*S*)-**1a** and (*R*)-**1a**, respectively, and no racemization was observed in both cases, excluding a DKR pathway (Figure 1B). Further, we investigated the activation of catalyst **3d** with *rac*-**1a** by ^31^P NMR analysis. Upon addition of racemic silane **1a** (40 equiv), the sharp singlet of IDPi **3d** (1.0 equiv) present at −4.5 ppm (Figure 1C, (i)) broadened due to the averaging of the acquired spectra during the drying of trace amounts of adventitious water (Figure 1C, (ii)). This signal sharpened again and shifted significantly to −6.3 ppm after complete drying (Figure 1C, (iii)). Finally, the catalyst was fully silylated, presumably mainly into diastereomers **Cat-Si1** and **Cat-Si2** and two additional minor species. The two main diastereomers feature two major doublets (*J*_pp_ = 97.4 Hz) at −2.0 and −7.0 ppm and two minor doublets (*J*_pp_ = 95.1 Hz) at −3.6 and −6.9 ppm, reflecting covalent bonding between the silicon atom and presumably an oxygen atom of the catalyst (Figure 1C, (iv)).^49^ It is interesting to note that there is interconversion between the epimers, which was confirmed by comparing the ^31^P NMR spectrum of the catalyst with those of (*R*)-**1a**, (*S*)-**1a** ,and *rac*-**1a** (see Figures S5–S10 in the Supporting Information). We also separately investigated the reaction of the three starting materials (*R*)-**1a**, (*S*)-**1a**, and *rac*-**1a** under the standard conditions. The same product enantiomer (*S*)-**4a** was obtained in all three cases, with similar results, showing that the IDPi catalyst largely controls the stereoselectivity, irrespective of enantiopurity and absolute configuration of the starting silane (see Figure S1 in the Supporting Information). This indicates the existence of similar intermediates, which likely are positively charged, as validated by a Hammett plot analysis (see Figure S12 in the Supporting Information). Furthermore, we monitored the reversion of the silylated catalyst into the protonated form and found the *Si*-(*R*) enantiomer to react much faster than its *Si*-(*S*) counterpart (Figure 1E,F).

**Figure 1:**
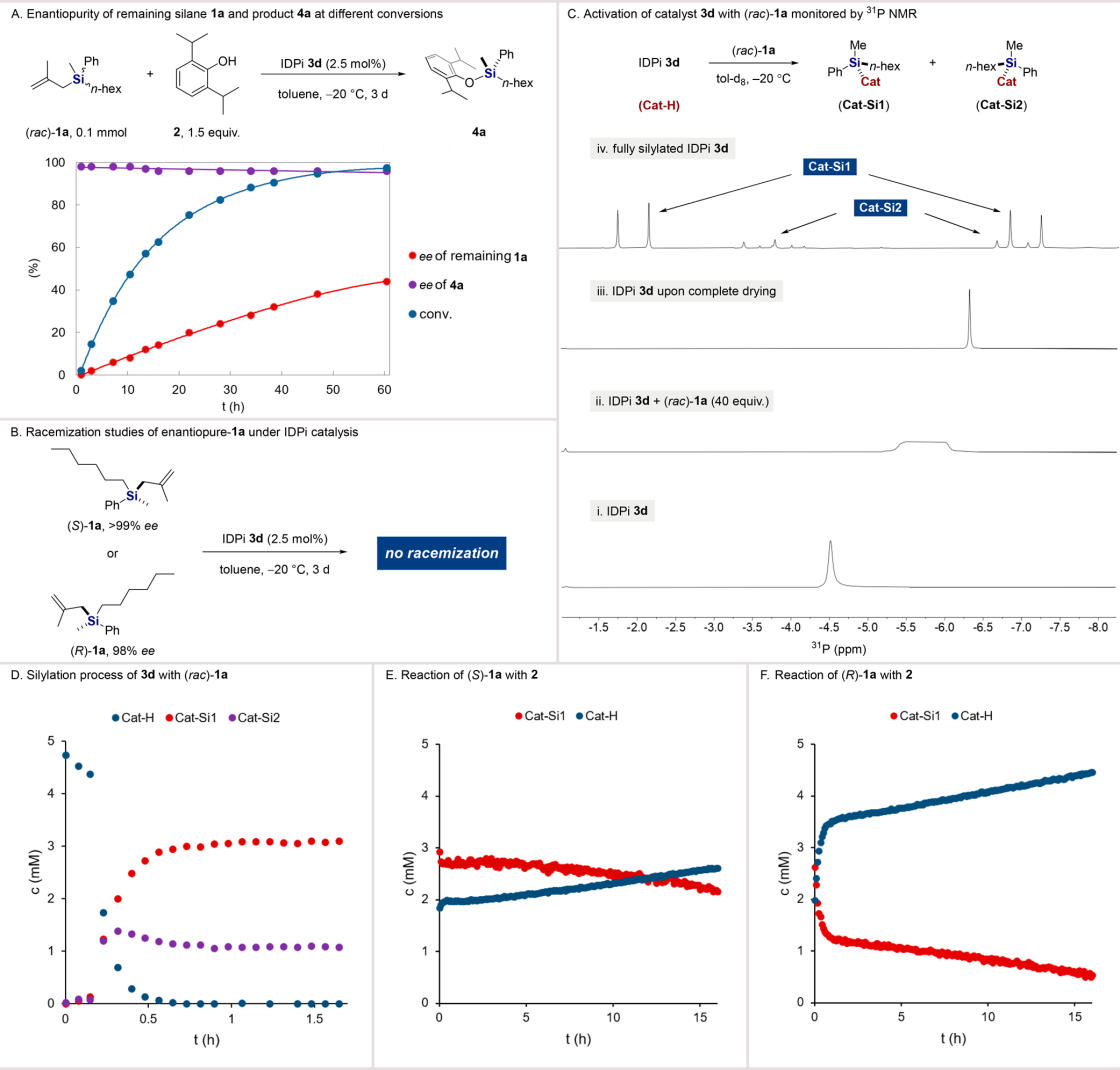
Mechanistic studies. (A) Time-course study with *rac*-**1a** (0.1 mmol, 1.0 equiv), **2** (0.15 mmol, 1.5 equiv), and IDPi **3d** (2.5 mol %) in toluene (0.1 M) at −20 °C under Ar for 3 days. Conversions were determined by GC, and enantiomeric ratios (e.r.) were determined by HPLC. (B) Racemization studies of either (*R*)-**1a** or (*S*)-**1a** (0.1 mmol) and IDPi **3d** (2.5 mol %) in toluene (0.2 M) at −20 °C for 3 days. (C) ^31^P NMR of the activation of catalyst with *rac*-**1a** (0.1 mmol, 1.0 equiv) and IDPi **3d** (2.5 mol %) in toluene-*d*_8_ (0.1 M) at −20 °C under Ar. (D) Silylation process performed under the same conditions as in (C), monitored by ^31^P NMR. (E) Reaction of (*S*)-**1a**, performed with (*S*)-**1a** (0.1 mmol, 1.0 equiv), **2** (0.15 mmol, 1.5 equiv), and IDPi **3d** (2.5 mol %) in toluene-*d*_8_ (0.1 M) at −20 °C under Ar. The variations of **Cat-Si1** and **Cat-H** were monitored by ^31^P NMR. (F) Reaction of (*R*)-**1a** under the same conditions as in (E).

Computational studies at the PBE-D3/def2-TZVP+CPCM (toluene) level of theory (see the Supporting Information for details) indicate that the diastereomers feature similar structures and a negligible energy difference (0.4 kcal/mol), which is within the expected computational error. Indeed, **Cat-Si1** and **Cat-Si2** show Si–O bond lengths of 1.81 and 1.82 Å, respectively. Nonetheless, they interconvert readily under the experimental conditions, with a reaction barrier of 19.2 kcal/mol, which is consistent with the experimental findings (Figure 2A, left). In order to explain the mode of interconversion, we located the transition state; this structure suggests the existence of a pentacoordinated silicon species, in which the Si atom simultaneously establishes two Si–O bonds with comparable lengths (2.22 and 2.16 Å). As a result, the silicon fragment and the two oxygen atoms of the IDPi’s sulfonyl groups arrange in space with a distorted-bipyramidal-trigonal geometry (Figure 2A, right).

**Figure 2:**
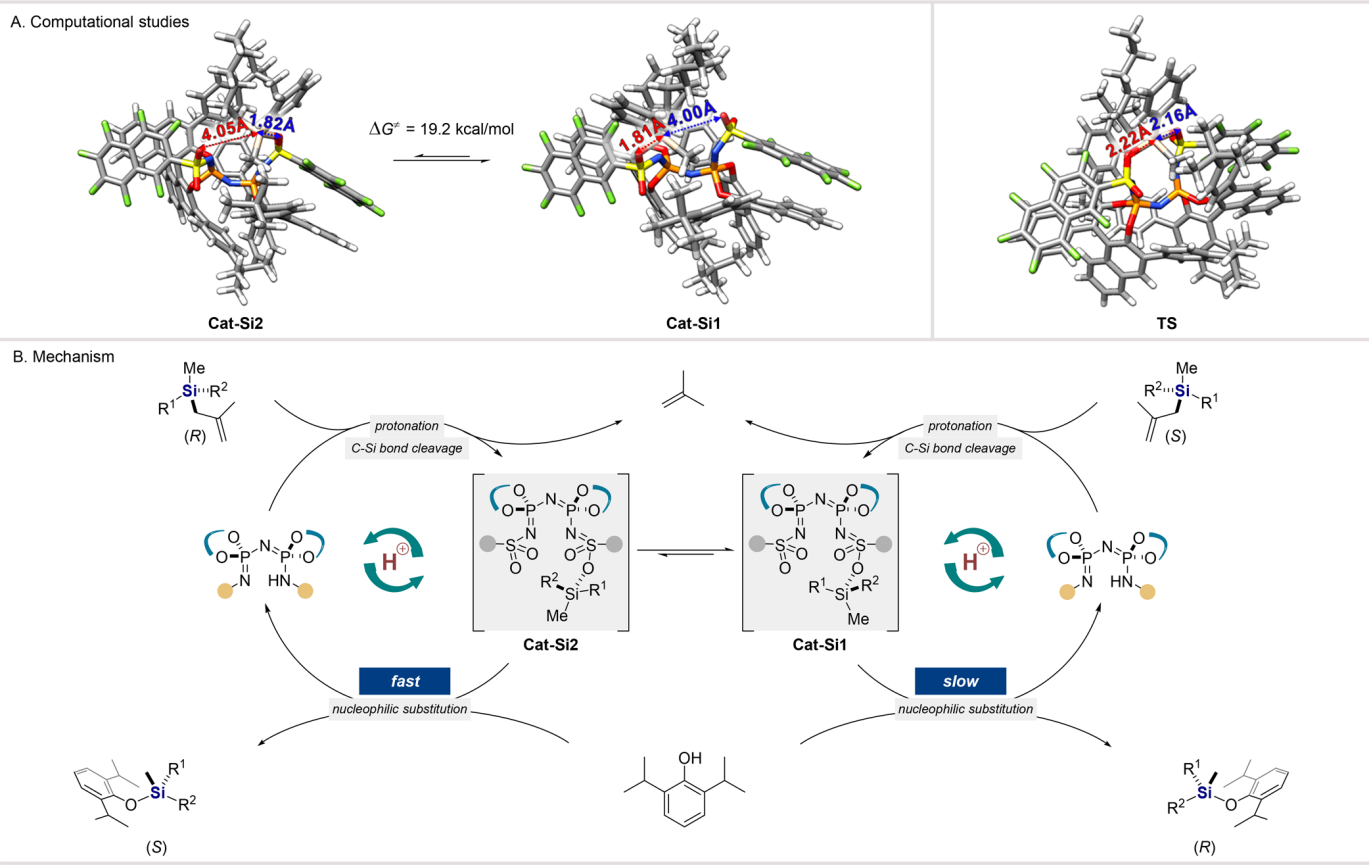
(A) Computational studies and (B) proposed mechanism.

Based on the accumulated experimental and computational results, a mechanism of IDPi-catalyzed DYKAT of racemic silane can be proposed (Figure 2B). First, the reaction sequence of protonation/C–Si bond cleavage of the silane enantiomers takes place, furnishing active silyl-catalyst species **Cat-Si1** and **Cat-Si2**, which interconvert as a result of the silyl group migration between the two diastereotopic oxygen atoms in the active site of the catalyst. The *S* enantiomer of the product is then generated through nucleophilic attack on **Cat-Si2** that is more favorable over the reaction of **Cat-Si1**.

In summary, we have developed an organocatalytic DYKAT of racemic allyl silanes using propofol as a nucleophile. A variety of functionalized silicon-stereogenic silyl ethers were constructed and readily derivatized to monohydrosilanes, potentially serving as general building blocks for chemical synthesis. Moreover, we elucidated a possible epimerization route triggered by the IDPi catalyst and supported a proposed mechanism by experimental and theoretical studies. By providing a general and direct transformation of racemic silanes into optically active *Si*-stereogenic compounds with high efficiency, this study paves a new avenue for organosilicon chemistry, which we believe will stimulate numerous applications in polymer and materials science, as well as in medicinal chemistry.

## Supporting Information Available

Experimental details and analytical data for all new compounds (PDF)

## References

[ref1] Hiyama T.; Shirakawa E. Organosilicon compounds. Cross-Coupling Reactions 2002, 219, 61–85. 10.1007/3-540-45313-X_3.

[ref2] Oestreich M. Silicon-Stereogenic Silanes in Asymmetric Catalysis. Synlett 2007, 2007, 1629–1643. 10.1055/s-2007-980385. PMC1810299 17288605

[ref3] Xu L. W.; Li L.; Lai G. Q.; Jiang J. X. The Recent Synthesis and Application of Silicon-Stereogenic Silanes: A Renewed and Significant Challenge in Asymmetric Synthesis. Chem. Soc. Rev. 2011, 40, 1777–1790. 10.1039/C0CS00037J. 21088772

[ref4] Xu L. W. Desymmetrization Catalyzed by Transition Metal Complex: Enantioselective Construction of Silicon-Stereogenic Silanes. Angew. Chem., Int. Ed. 2012, 51, 12932–12934. 10.1002/anie.201207932. 23255401

[ref5] Shintani R. Recent Advances in the Transition-Metal-Catalyzed Enantioselective Synthesis of Silicon-Stereogenic Organosilanes. Asian J. Org. Chem. 2015, 4, 510–514. 10.1002/ajoc.201500066.

[ref6] Cui Y. M.; Lin Y.; Xu L. W. Catalytic synthesis of chiral organoheteroatom compounds of silicon, phosphorus, and sulfur via asymmetric transition metal-catalyzed C-H functionalization. Coord. Chem. Rev. 2017, 330, 37–52. 10.1016/j.ccr.2016.09.011.

[ref7] Shintani R. Recent progress in catalytic enantioselective desymmetrization of prochiral organosilanes for the synthesis of silicon-stereogenic compounds. Synlett 2018, 29, 388–396. 10.1055/s-0036-1591839.

[ref8] Hiyama T.; Oestreich M. Organosilicon Chemistry: Novel Approaches and Reactions; Wiley: 2020.

[ref9] Zheng L.; Nie X. X.; Wu Y.; Wang P. Construction of Si-Stereogenic Silanes through C-H Activation Approach. Eur. J. Org. Chem. 2021, 2021, 6006–6014. 10.1002/ejoc.202101084.

[ref10] He C.; Yuan W. Enantioselective C-H Functionalization toward Silicon-Stereogenic Silanes. Synthesis 2022, 54, 1939–1950. 10.1055/a-1729-9664.

[ref11] Ahlrichs R.; Heinzmann R. Stability and reactivity of the silicon-carbon double bond. J. Am. Chem. Soc. 1977, 99, 7452–7456. 10.1021/ja00465a009.

[ref12] Morkin T. L.; Leigh W. J. Substituent Effects on the Reactivity of the Silicon-Carbon Double Bond. Acc. Chem. Res. 2001, 34, 129–136. 10.1021/ar960252y. 11263871

[ref13] Mori A.; Toriyama F.; Kajiro H.; Hirabayashi K.; Nishihara Y.; Hiyama T. Synthesis and optical resolution of novel chiral silanols. Chem. Lett. 1999, 28, 549–550. 10.1246/cl.1999.549.

[ref14] Sommer L.; Frye C.; Parker G.; Michael K.-W. Stereochemistry of Asymmetric Silicon. I. Relative and Absolute Configurations of Optically Active α-Naphthylphenylmethylsilanes. J. Am. Chem. Soc. 1964, 86, 3271–3276. 10.1021/ja01070a014.

[ref15] Bauer J. O.; Strohmann C. Recent progress in asymmetric synthesis and application of difunctionalized silicon-stereogenic silanes. Eur. J. Inorg. Chem. 2016, 2016, 2868–2881. 10.1002/ejic.201600100.

[ref16] Ye F.; Xu Z.; Xu L. W. The Discovery of Multifunctional Chiral P Ligands for the Catalytic Construction of Quaternary Carbon/Silicon and Multiple Stereogenic Centers. Acc. Chem. Res. 2021, 54, 452–470. 10.1021/acs.accounts.0c00740. 33375791

[ref17] Zhou H.; Han J. T.; Nöthling N.; Lindner M. M.; Jenniches J.; Kühn C.; Tsuji N.; Zhang L.; List B. Organocatalytic Asymmetric Synthesis of Si-Stereogenic Silyl Ethers. J. Am. Chem. Soc. 2022, 144, 10156–10161. 10.1021/jacs.2c04261. 35649270 PMC9490845

[ref18] Yamamoto K.; Kawanami Y.; Miyazawa M. Kinetic resolution of (±)-cyclohex-1-enylsilanols by the sharpless asymmetric epoxidation. J. Chem. Soc., Chem. 1993, 5, 436–437. 10.1039/C39930000436.

[ref19] Tacke R.; Heinrich T. Syntheses of enantiopure Si-centrochiral silicon-based muscarinic antagonists using an enantioselective enzymatic esterification as the key step. Silicon Chem. 2002, 1, 35–39. 10.1023/A:1016095507813.

[ref20] Rendler S.; Auer G.; Keller M.; Oestreich M. Preparation of a Privileged Silicon-Stereogenic Silane: Classical versus Kinetic Resolution. Adv. Synth. Catal. 2006, 348, 1171–1182. 10.1002/adsc.200606071.

[ref21] Guo Y.; Liu M. M.; Zhu X.; Zhu L.; He C. Catalytic Asymmetric Synthesis of Silicon-Stereogenic Dihydrodibenzosilines: Silicon Central-to-Axial Chirality Relay. Angew. Chem., Int. Ed. 2021, 133, 14006–14010. 10.1002/ange.202103748. 33830619

[ref22] Zeng Y.; Fang X. J.; Tang R. H.; Xie J. Y.; Zhang F. J.; Xu Z.; Nie Y. X.; Xu L. W. Rhodium-Catalyzed Dynamic Kinetic Asymmetric Hydrosilylation to Access Silicon-Stereogenic Center. Angew. Chem., Int. Ed. 2022, 61, e202214147 10.1002/anie.202214147. 36328976

[ref23] Bauer J. O.; Strohmann C. From an α-Functionalized Silicon-Stereogenic N, O-Silane to a Monomeric and Tetracoordinate *t*BuLi Adduct with Lithium-Centered Chirality. Angew. Chem., Int. Ed. 2014, 53, 8167–8171. 10.1002/anie.201404255. 24939015

[ref24] Igawa K.; Kokan N.; Tomooka K. Asymmetric Synthesis of Chiral Silacarboxylic Acids and Their Ester Derivatives. Angew. Chem., Int. Ed. 2010, 49, 728–731. 10.1002/anie.200904922. 20029860

[ref25] Shintani R.; Maciver E. E.; Tamakuni F.; Hayashi T. Rhodium-Catalyzed Asymmetric Synthesis of Silicon-Stereogenic Dibenzooxasilines via Enantioselective Transmetalation. J. Am. Chem. Soc. 2012, 134, 16955–16958. 10.1021/ja3076555. 22998336

[ref26] Ducos P.; Liautard V.; Robert F.; Landais Y. Chiral Memory in Silylium Ions. Chem. -Eur. J. 2015, 21, 11573–11578. 10.1002/chem.201501987. 26139434

[ref27] Mahlau M.; List B. Asymmetric Counteranion-Directed Catalysis: Concept, Definition, and Applications. Angew. Chem., Int. Ed. 2013, 52, 518–533. 10.1002/anie.201205343. 23280677

[ref28] Wang Q.; Leutzsch M.; van Gemmeren M.; List B. Disulfonimide-catalyzed asymmetric synthesis of β^3^-amino esters directly from N-Boc-Amino sulfones. J. Am. Chem. Soc. 2013, 135, 15334–15337. 10.1021/ja408747m. 24090068

[ref29] Wang Q.; van Gemmeren M.; List B. Asymmetric disulfonimide-catalyzed synthesis of δ-amino-β-ketoester derivatives by vinylogous Mukaiyama-Mannich reactions. Angew. Chem., Int. Ed. 2014, 53, 13592–13595. 10.1002/anie.201407532. 25348924

[ref30] Zhang Z.; Bae H. Y.; Guin J.; Rabalakos C.; van Gemmeren M.; Leutzsch M.; Klussmann M.; List B. Asymmetric Counteranion-Directed Lewis Acid Organocatalysis for the Scalable Cyanosilylation of Aldehydes. Nat. Commun. 2016, 7, 12478 10.1038/ncomms12478. 27530470 PMC4992067

[ref31] Kaib P. S.; Schreyer L.; Lee S.; Properzi R.; List B. Extremely active organocatalysts enable a highly enantioselective addition of allyltrimethylsilane to aldehydes. Angew. Chem., Int. Ed. 2016, 55, 13200–13203. 10.1002/anie.201607828. 27653018

[ref32] Lee S.; Kaib P. S.; List B. Asymmetric Catalysis via Cyclic, Aliphatic Oxocarbenium Ions. J. Am. Chem. Soc. 2017, 139, 2156–2159. 10.1021/jacs.6b11993. 28169541

[ref33] Bae H. Y.; Höfler D.; Kaib P. S.; Kasaplar P.; De C. K.; Döhring A.; Lee S.; Kaupmees K.; Leito I.; List B. Approaching Sub-PPM-Level Asymmetric Organocatalysis of a Highly Challenging and Scalable Carbon-Carbon Bond Forming Reaction. Nat. Chem. 2018, 10, 888 10.1038/s41557-018-0065-0. 29988150

[ref34] Gatzenmeier T.; Turberg M.; Yepes D.; Xie Y.; Neese F.; Bistoni G.; List B. Scalable and Highly Diastereo-and Enantioselective Catalytic Diels-Alder Reaction of α, β-Unsaturated Methyl Esters. J. Am. Chem. Soc. 2018, 140, 12671–12676. 10.1021/jacs.8b07092. 30277760

[ref35] Schreyer L.; Kaib P. S.; Wakchaure V. N.; Obradors C.; Properzi R.; Lee S.; List B. Confined Acids Catalyze Asymmetric Single Aldolizations of Acetaldehyde Enolates. Science 2018, 362, 216–219. 10.1126/science.aau0817. 30309951

[ref36] Bae H. Y.; List B. Triflimide: An Overlooked High-Performance Catalyst of the Mukaiyama Aldol Reaction of Silyl Ketene Acetals with Ketones. Eur. J. Chem. 2018, 24, 13767–13772. 10.1002/chem.201803142. 29943495

[ref37] Lee S.; Bae H. Y.; List B. Can a ketone be more reactive than an aldehyde? Catalytic asymmetric synthesis of substituted tetrahydrofurans. Angew. Chem., Int. Ed. 2018, 57, 12162–12166. 10.1002/anie.201806312. 30126072

[ref38] Schreyer L.; Properzi R.; List B. IDPi Catalysis. Angew. Chem., Int. Ed. 2019, 58, 12761–12777. 10.1002/anie.201900932. 30840780

[ref39] Mandrelli F.; Blond A.; James T.; Kim H.; List B. Deracemizing α-Branched Carboxylic Acids by Catalytic Asymmetric Protonation of Bis-Silyl Ketene Acetals with Water or Methanol. Angew. Chem., Int. Ed. 2019, 58, 11479–11482. 10.1002/anie.201905623. 31131975

[ref40] Zhang Z.; Klussmann M.; List B. Kinetic study of disulfonimide-catalyzed cyanosilylation of aldehydes by using a method of progress rates. Synlett 2020, 31, 1593–1597. 10.1055/s-0040-1707129.

[ref41] Zhou H.; Bae H. Y.; Leutzsch M.; Kennemur J. L.; Bécart D.; List B. The Silicon-Hydrogen Exchange Reaction: A Catalytic σ-Bond Metathesis Approach to the Enantioselective Synthesis of Enol Silanes. J. Am. Chem. Soc. 2020, 142, 13695–13700. 10.1021/jacs.0c06677. 32786813 PMC7426905

[ref42] Zhu C.; Mandrelli F.; Zhou H.; Maji R.; List B. Catalytic Asymmetric Synthesis of Unprotected β^2^-Amino Acids. J. Am. Chem. Soc. 2021, 143, 3312–3317. 10.1021/jacs.1c00249. 33645969 PMC7953379

[ref43] Amatov T.; Tsuji N.; Maji R.; Schreyer L.; Zhou H.; Leutzsch M.; List B. Confinement-Controlled, Either syn- or anti-Selective Catalytic Asymmetric Mukaiyama Aldolizations of Propionaldehyde Enolsilanes. J. Am. Chem. Soc. 2021, 143, 14475–14481. 10.1021/jacs.1c07447. 34436899 PMC8447262

[ref44] Zhou H.; Zhang P.; List B. The Silicon-Hydrogen Exchange Reaction: Catalytic Kinetic Resolution of 2-Substituted Cyclic Ketones. Synlett 2021, 32, 1953–1956. 10.1055/a-1670-5829.

[ref45] Das S.; Mitschke B.; De C. K.; Harden I.; Bistoni G.; List B. Harnessing the ambiphilicity of silyl nitronates in a catalytic asymmetric approach to aliphatic β^3^-amino acids. Nat. Catal. 2021, 4, 1043–1049. 10.1038/s41929-021-00714-x.

[ref46] Obradors C.; List B. Azine activation via silylium catalysis. J. Am. Chem. Soc. 2021, 143, 6817–6822. 10.1021/jacs.1c03257. 33908753 PMC8154516

[ref47] Ouyang J.; Bae H.; Jordi S.; Dao Q. M.; Dossenbach S.; Dehn S.; Lingnau J. B.; De C. K.; Kraft P.; List B. The smelling principle of vetiver oil, unveiled by chemical synthesis. Angew. Chem., Int. Ed. 2021, 60, 5666–5672. 10.1002/anie.202014609. PMC7986879 33315304

[ref48] Zhou H.; Zhou Y.; Bae H. Y.; Leutzsch M.; Li Y. H.; De C. K.; Cheng G.-J.; List B. Organocatalytic stereoselective cyanosilylation of small ketones. Nature 2022, 605, 84–89. 10.1038/s41586-022-04531-5. 35508776 PMC9068509

[ref49] Ouyang J.; Maji R.; Leutzsch M.; Mitschke B.; List B. Design of an Organocatalytic Asymmetric (4 + 3) Cycloaddition of 2-Indolylalcohols with Dienolsilanes. J. Am. Chem. Soc. 2022, 144, 8460–8466. 10.1021/jacs.2c02216. 35523203 PMC9121375

[ref50] Grossmann O.; Maji R.; Aukland M. H.; Lee S.; List B. Catalytic Asymmetric Additions of Enol Silanes to In Situ Generated Cyclic, Aliphatic N-Acyliminium Ions. Angew. Chem., Int. Ed. 2022, 61, e202115036 10.1002/anie.202115036. PMC9303265 34897932

[ref51] Smaller *o*-substituents (i.e., methyl) on the phenol gave lower enantioselectivity, while larger substituents (i.e., *t*-Bu) gave similar reactivity. Considering practicality, we chose readily available and inexpensive propofol throughout our studies.

[ref52] Phan T. B.; Breugst M.; Mayr H. Towards a General Scale of Nucleophilicity?. Angew. Chem., Int. Ed. 2006, 45, 3869–3874. 10.1002/anie.200600542. 16646102

[ref53] Sommer L.; McLick J.; Golino C. SNi-Si Mechanism. Reductive Displacement of Good Leaving Groups with Retention of Configuration by Diisobutylaluminum Hydride. Stereochemical and Mechanistic Crossover with the Etherate Complex of Diisobutylaluminum Hydride. J. Am. Chem. Soc. 1972, 94, 669–670. 10.1021/ja00757a078.

[ref54] Wu Y.; Wang P. Silicon-Stereogenic Monohydrosilane: Synthesis and Applications. Angew. Chem., Int. Ed. 2022, 61, e202205382 10.1002/anie.202205382. 35594056

