# Supplementary material for: Organocatalytic DYKAT of Si-Stereogenic Silanes
Source: J Am Chem Soc. 2023 Feb 24;145(9):4994–5000. doi: 10.1021/jacs.3c00858 (PMC9999423; doi:10.1021/jacs.3c00858)
Supplement: Supplementary file 1 — ja3c00858_si_001.pdf [file ja3c00858_si_001.pdf]

Supplementary Information

Organocatalytic DYKAT of *Si*-Stereogenic Silanes

Hui Zhou[†#], Roberta Properzi[†#], Markus Leutzsch[†], Paola Belanzoni[‡], Giovanni Bistoni[‡], Nobuya Tsuji[§], Jung Tae Han[†], Chendan Zhu[†], and Benjamin List[†§*]

[†] Max-Planck-Institut für Kohlenforschung, Kaiser-Wilhelm-Platz 1, 45470 Mülheim an der Ruhr, Germany

[‡] University of Perugia, Department of Chemistry, Biology and Biotechnology, 06122 Perugia, Italy

[§] Institute for Chemical Reaction Design and Discovery (WPI-ICReDD), Hokkaido University, Sapporo 001-0021, Japan

*Email: list@kofo.mpg.de

[#]H.Z. and R.P. contributed equally

Content

1. General considerations S2

2. Development of suitable reaction conditions and catalyst identification S4

3. The preparation and characterization of the silicon starting materials S7

4. Substrate scope for the DYKAT of racemic silanes S12

5. Gram scale synthesis and elaboration of **4a** S20

6. Mechanistic studies S22

7. Computational studies S32

8. Absolute configuration determination S54

9. References S66

10. Copies of NMR spectra S68

11. Copies of HPLC traces S129

1. General considerations

Chemicals

Unless otherwise indicated, starting materials were obtained from Sigma-Aldrich, ABCR-GmbH, TCI, or Acros Co. Ltd. Moreover, commercially available reagents were used without additional purification. Silane starting materials were synthesized according to literature procedure with minor modifications.[1] The chiral imidodiphosphorimidate acid (IDPis) **3b**[2] and **3d** were synthesized according to literature procedures.[3]

Solvents

Solvents (Et_2O, Cyclohexane, dichloromethane, THF and Toluene) were dried by distillation from an appropriate drying agent in the technical department of the Max-Planck-Institut für Kohlenforschung and received in Schlenk flasks under argon. In addition, CH_3CN was purchased from commercial suppliers and dried over molecular sieves.

Inert Gas

Dry argon was purchased from Air Liquide with >99.5% purity.

Thin Layer Chromatography

Thin-layer chromatography (TLC) was performed using silica gel pre-coated plastic sheets (Polygram SIL G/UV_{254}, 0.2 mm, with fluorescent indicator; Macherey-Nagel) which was visualized with a UV lamp (254 nm) and/or phosphomolybdic acid (PMA). PMA stain: PMA (20 g) in EtOH (200 mL).

Column Chromatography

Column chromatography (CC) was carried out using Merck silica gel (60 Å, 230‒400 mesh, particle size 0.040‒0.063 mm) using technical grade solvents. Elution was accelerated using compressed argon. All reported yields, unless otherwise specified, refer to spectroscopically and chromatographically pure compounds.

Nomenclature

Nomenclature follows the suggestions proposed by the computer program ChemDraw (20.1.1) of CBD/cambridgesoft.

Nuclear Magnetic Resonance Spectroscopy

^{1}H, ^{13}C, ^{19}F, ^{31}P Nuclear magnetic resonance (NMR) spectra for compound characterization were recorded on Bruker AVIII-500 MHz, NMR spectrometer in a suitable deuterated solvent. The solvent employed and the respective measuring frequency are indicated for each experiment. Chemical shifts are reported with tetramethylsilane (TMS) serving as a universal reference of all nuclides. The resonance multiplicity is described as s (singlet), d (doublet), t (triplet), q (quadruplet), m (multiplet), and b (broad). All spectra were recorded at 298 K, processed with MestReNova 14.2.3 suits of program, and coupling constants are reported as observed. The residual deuterated solvent signal relative to tetramethylsilane was used as the internal reference in ^{1}H and ^{13}C NMR spectra (e.g. CD_2Cl_2 = 5.32 ppm in ^{1}H NMR and 53.8 ppm in ^{13}C NMR).[4] Signals are reported as follows: chemical shift δ in ppm (multiplicity, coupling constant *J* in Hz, number of protons). All X-nuclei spectra were acquired proton decoupled unless otherwise noted. The temperature of low

temperature experiments was calibrated against a 4% MeOH in MeOD-d_4 sample.[5] Further details are mentioned in the corresponding section.

Mass Spectrometry

Electrospray ionization (ESI) mass spectrometry was conducted on a Bruker ESQ 3000 spectrometer. High resolution mass spectra were determined on a Bruker APEX III FTMS (7 T magnet). The ionization method and mode of detection employed is indicated for the respective experiment and all masses are reported in atomic units per elementary charge (*m/z*) with an intensity normalized to the most intense peak.

Specific Rotations

Specific rotations ($[\alpha]_D^T$) were measured with a Rudolph RA Autopol IV Automatic Polarimeter at the indicated temperature with a sodium lamp (sodium D line, λ = 589 nm). Measurements were performed in an acid resistant 1 mL cell (50 mm length) with concentrations (g/(100 mL)) reported in the corresponding solvent.

High Performance Liquid Chromatography

High performance liquid chromatography (HPLC) was performed on a Shimadzu LC-20AD liquid chromatograph SIL-20AC auto sampler, CMB-20A using Daicel columns with a chiral stationary phase. All solvents used were HPLC-grade solvents purchased from Sigma-Aldrich. The column employed and the respective solvent mixture are indicated for each experiment.

Abbreviations

e.r. = enantiomeric ratio, TLC = thin layer chromatography, CH_3CN = acetonitrile, Tf = SO_2CF_3, propofol = 2,6-diisopropylphenol.

2. Development of suitable reaction conditions and catalyst identification

We initiated our research with hexyl(methyl)(2-methylallyl)(phenyl)silane and 2,6-dimethylphenol as model substrates as shown below:

General procedure for the initial optimization of the organocatalytic asymmetric synthesis of chiral *Si*-stereogenic silyl ether from racemic silanes employing commonly used Brønsted acid catalysts.

To a 1.5 mL GC vial which was equipped with a teflon-coated magnetic stirring bar, 2,6-dimethylphenol (0.075 mmol, 1.5 equiv.), catalysts (2.5 mol%) and solvent (0.1 M, 0.5 mL) were added. The resultant solution was stirred for 5 min at rt. Racemic silane starting material **1a** (0.05 mmol, 1.0 equiv.) was added and the reaction mixture was stirred for an additional 24 h at the indicated temperature, then the reaction mixture was treated with triethylamine and volatiles were removed in vacuo and the yield of the corresponding product was determined by NMR analysis with dibromomethane as internal standard. The enantiomeric ratio was determined by HPLC after purification by prep. TLC using hexanes as eluent. (**Table S1**)

Table S1. Initial screening with commonly used Brønsted acid catalysts:[a]

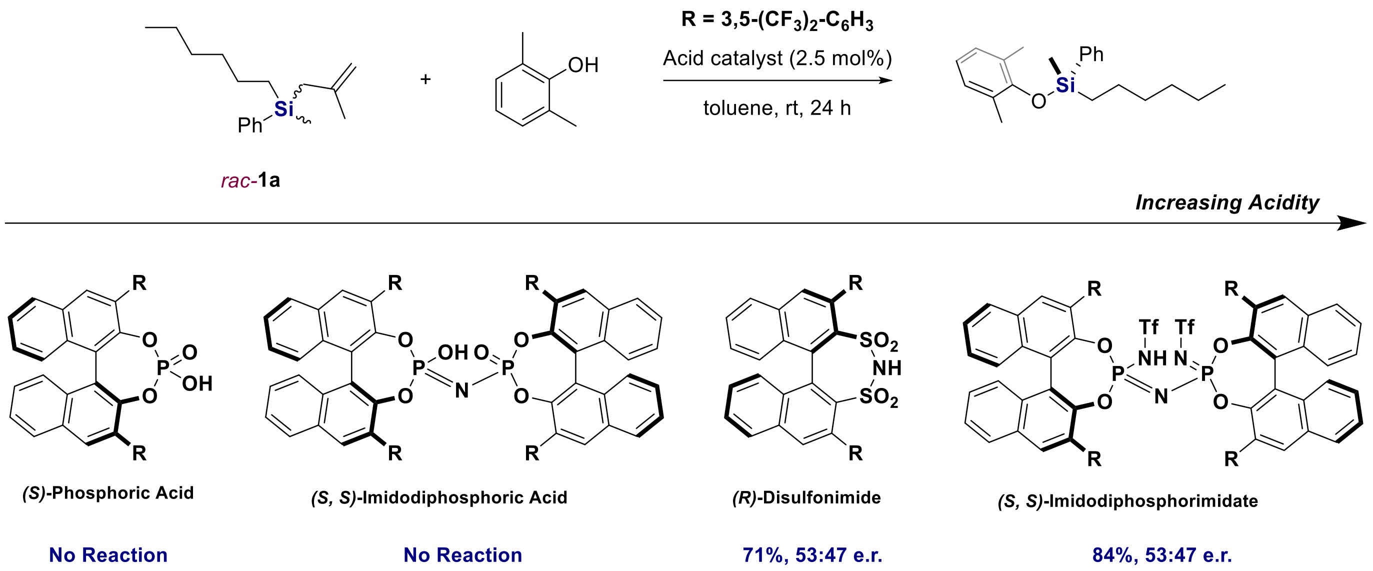

[a]Reactions were performed with 2,6-dimethylphenol (0.075 mmol, 1.5 equiv.), *rac*-**1a** (0.05mmol, 1.0 equiv.), and IDPi catalysts (2.5 mol%) in toluene (0.5 mL, 0.1 M), yields were determined by ^{1}H NMR using dibromomethane as internal standard and enantiomeric ratios (e.r.) were determined by HPLC.

From the screening results, we found the reactivity highly relied on the acidity of the catalysts. Based on our previous experience of silicon chemistry, we decide to explore the reaction catalyzed by the more confined IDPi catalysts.

Table S2. Screening of the different IDPi catalysts using 2,6-dimethylphenol as nucleophile:[a]

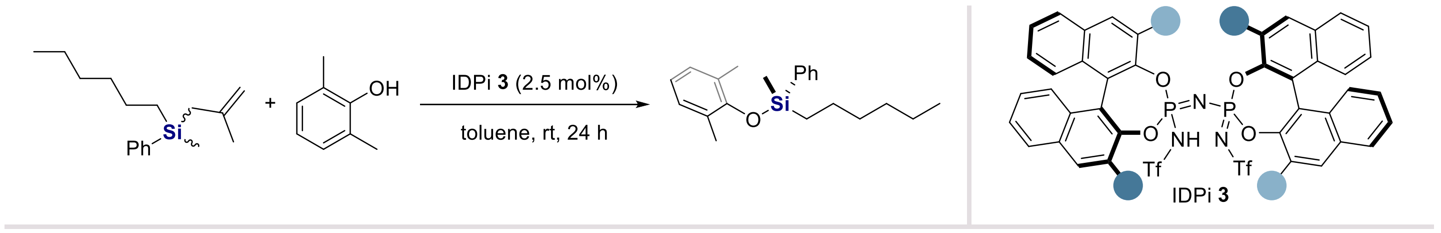

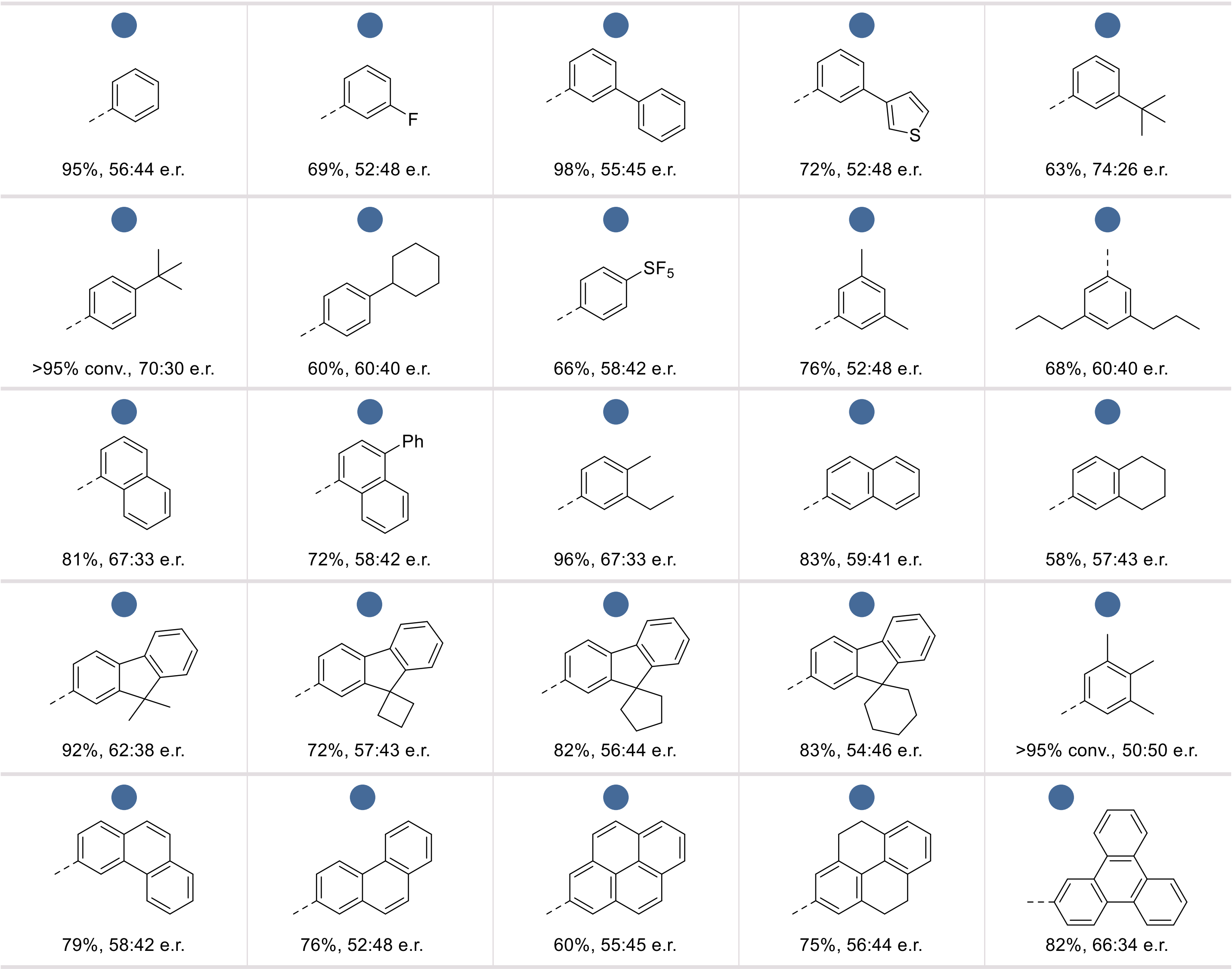

[a]Reactions were performed with 2,6-dimethylphenol (0.0375 mmol, 1.5 equiv.), *rac*-**1a** (0.025mmol, 1.0 equiv.), and IDPi catalysts (2.5 mol%) in toluene (0.25 mL, 0.1 M), yields were determined by ^{1}H NMR using dibromomethane as internal standard and enantiomeric ratios (e.r.) were determined by HPLC.

To a 1.5 mL GC vial which was equipped with a teflon-coated magnetic stirring bar, 2,6-dimethylphenol (0.0375 mmol, 1.5 equiv.), catalysts (2.5 mol%) and toluene (0.1 M, 0.25 mL) were added. The resultant solution was stirred for 5 min at rt. Racemic silane starting material **1a** (0.025 mmol, 1.0 equiv.) was added and the reaction mixture was stirred for an additional 24 h at the indicated temperature. After full conversion of **1a** was confirmed by TLC analysis, the reaction mixture was treated with triethylamine and volatiles were removed in vacuo and the yield of the corresponding product was determined by NMR analysis with dibromomethane as internal standard. The enantiomeric ratio was determined by HPLC after purification by prep. TLC using hexanes as eluent. (**Table S2**)

The reactions were catalyzed by IDPi catalysts bearing different alkyl and aryl groups; after we obtained the promising but moderate enantiomeric ratios summarized in **Table S2**, we realized that enlarging the group on the aryl ring of the phenol nucleophile was quite necessary, as we explained with DFT calculations in our previously reported work.[1]

Next, we sought to examine several more sterically-hindered nucleophiles, and the results were summarized in **Table S3**.

Table S3. Screening of different phenol nucleophiles:[a]

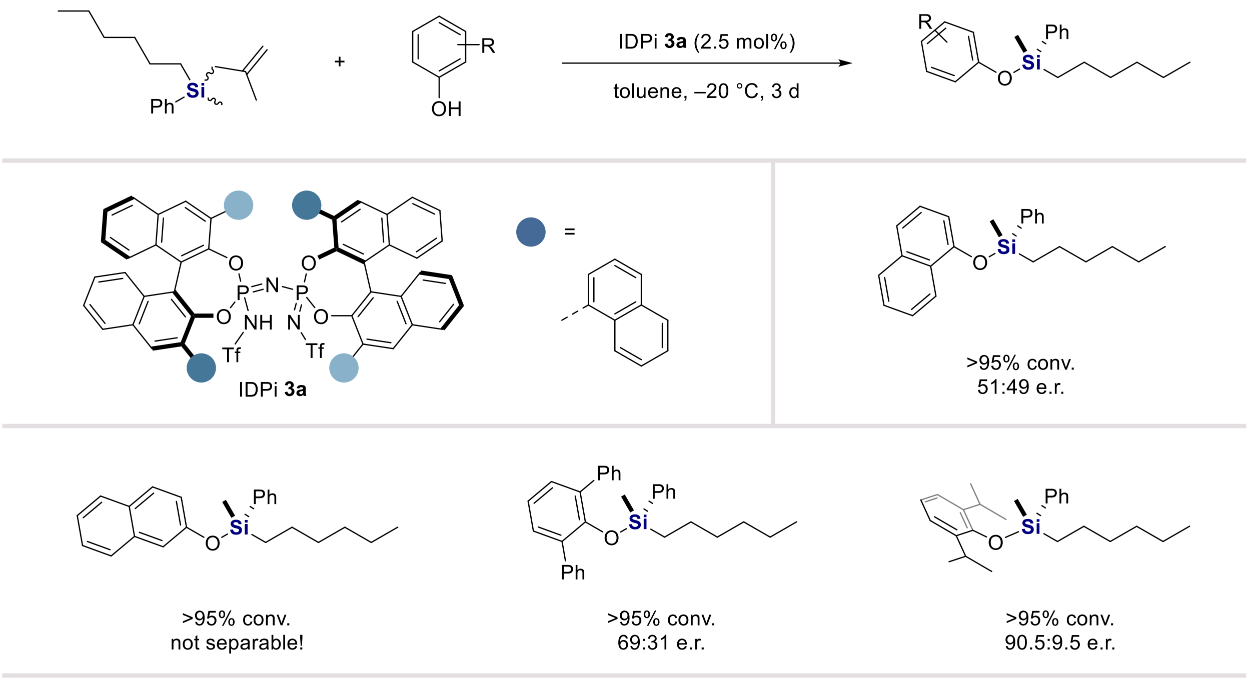

[a]Reactions were performed with different phenols (0.0375 mmol, 1.5 equiv.), *rac*-**1a** (0.025mmol, 1.0 equiv.), and IDPi **3a** (2.5 mol%) in toluene (0.25 mL, 0.1 M) at –20 °C, and enantiomeric ratios (e.r.) were determined by HPLC.

To a 1.5 mL GC vial which was equipped with a teflon-coated magnetic stirring bar, different phenols (0.0375 mmol, 1.5 equiv.), IDPi **3a** (2.5 mol%) and toluene (0.1 M, 0.25 mL) were added. The resultant solution was stirred for 10 min at –20 °C. Racemic silane starting material **1a** (0.025 mmol, 1.0 equiv.) was added and the reaction mixture was stirred for an additional 3 d at the indicated temperature. After full conversion of **1a** was confirmed by TLC analysis, the reaction mixture was treated with triethylamine and volatiles were removed in vacuo. The enantiomeric ratio of the corresponding product was determined by HPLC after purification by prep. TLC using hexanes as eluent. (**Table S3**)

These comparison experiments showed the vital role of the nucleophile for the high enantioselectivity, thus the following optimization of the reaction conditions was conducted with 2,6-diisopropylphenol (propofol) as nucleophile. (**Table S4**)

Table S4. Screening of the different IDPi catalysts using 2,6-diisopropylphenol as nucleophile:[a]

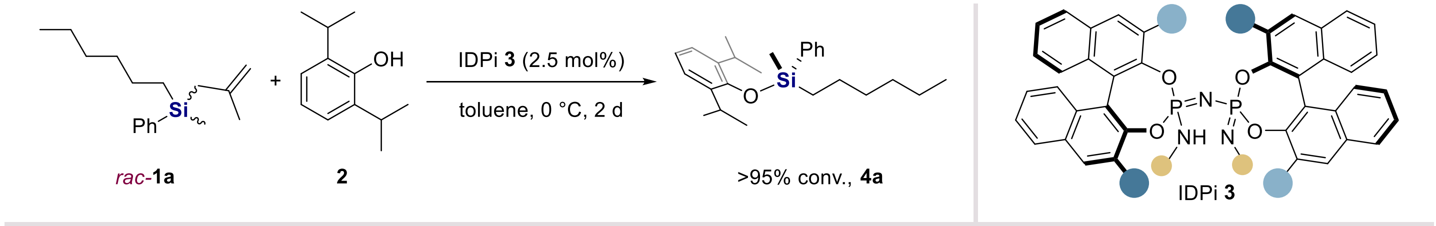

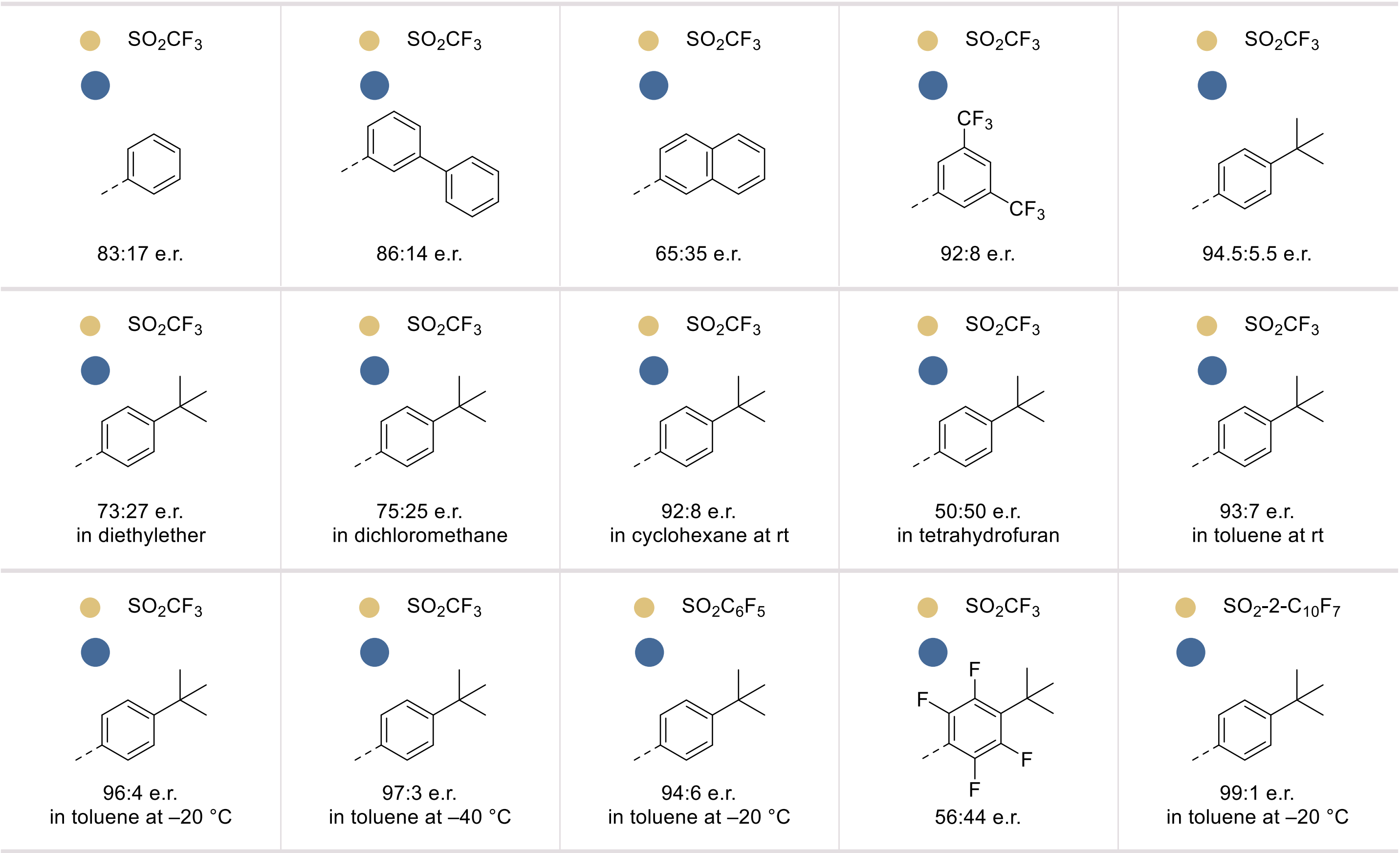

[a]Reactions were performed with propofol **2** (0.0375 mmol, 1.5 equiv.), *rac*-**1a** (0.025mmol, 1.0 equiv.), and IDPi catalysts (2.5 mol%) in toluene (0.25 mL, 0.1 M), and enantiomeric ratios (e.r.) were determined by HPLC.

To a 1.5 mL GC vial which was equipped with a teflon-coated magnetic stirring bar, propofol **2** (0.0375 mmol, 1.5 equiv.), IDPi catalysts **3** (2.5 mol%) and solvents (0.1 M, 0.25 mL) were added. The resultant solution was stirred for 5–10 min. Racemic silane starting material **1a** (0.025 mmol, 1.0 equiv.) was added and the reaction mixture was stirred for an additional 2 d at the indicated temperature. After full conversion of **1a** was confirmed by TLC analysis, the reaction mixture was treated with triethylamine and volatiles were removed in vacuo. The enantiomeric ratio of the corresponding product was determined by HPLC after purification by prep. TLC using hexanes as eluent. (**Table S4**)

3. The preparation and characterization of the silicon starting materials[1]

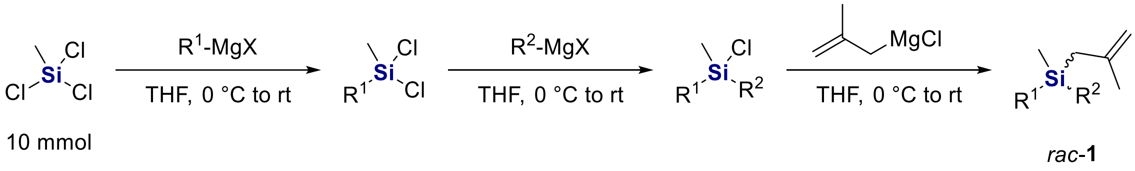

Procedure: To a solution of methyl trichlorosilane (10.0 mmol) in THF (20 mL), different Grignard reagents R^1-MgX (10.0 mmol) were added at 0 ºC under argon atmosphere. After the resulting mixture was stirred at rt for 2 h, Grignard reagents R^2-MgX (10.0 mmol) were added at 0 ºC under argon atmosphere and the resultant solution was stirred at rt for 2 h. A solution of (2-methylallyl)magnesium chloride (0.75 M in THF, 1.5 equivalent, 15 mmol) was added to the mixture at 0 °C. The reaction mixture was gradually warmed up to rt and stirred for 24 h. After the completion that was indicated by TLC, the reaction was quenched with sat. aq. NH_4Cl and extracted with

hexanes. The combined organic phases were dried over Na_2SO_4, filtrated and dried under reduced pressure. Purification of the crude by silica gel chromatography with hexanes afforded the desired products *rac*-**1**.

hexyl(methyl)(2-methylallyl)(phenyl)silane (1a)

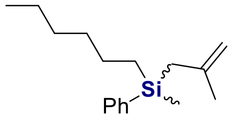

^{1}H NMR (501 MHz, CD_2Cl_2) δ 7.54–7.48 (m, 2H), 7.34 (dd, *J* = 4.8, 1.9 Hz, 3H), 4.57 (dd, *J* = 2.5, 1.4 Hz, 1H), 4.46 (dq, *J* = 2.0, 0.9 Hz, 1H), 1.79 (t, *J* = 0.9 Hz, 2H), 1.61–1.56 (m, 3H), 1.38–1.19 (m, 8H), 0.90–0.84 (m, 3H), 0.81 (ddd, *J* = 8.9, 6.6, 1.8 Hz, 2H), 0.30 (s, 3H).

^{13}C NMR (126 MHz, CD_2Cl_2) δ 143.9, 138.8, 134.3, 129.2, 128.0, 108.9, 33.7, 31.9, 26.5, 25.4, 24.1, 23.0, 14.5, 14.3, –5.0.

R$_f$ = 0.65 (hexanes).

ESI-HRMS (*m/z*): calculated for $C_{17}H_{28}Si_1Na_1$ ([M+Na]$^+$): 283.1852, found: 283.1852.

ethyl(methyl)(2-methylallyl)(phenyl)silane (1b)

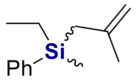

^{1}H NMR (501 MHz, CD_2Cl_2) δ 7.55–7.47 (m, 2H), 7.34 (dd, *J* = 4.9, 1.9 Hz, 3H), 4.57 (dd, *J* = 2.5, 1.4 Hz, 1H), 4.47 (dd, *J* = 2.3, 1.0 Hz, 1H), 1.80 (t, *J* = 1.2 Hz, 2H), 1.60 (t, *J* = 1.1 Hz, 3H), 1.00–0.93 (m, 3H), 0.90–0.73 (m, 2H), 0.30 (s, 3H).

^{13}C NMR (126 MHz, CD_2Cl_2) δ 143.4, 138.0, 133.9, 128.8, 127.6, 108.5, 25.7, 25.0, 7.1, 5.8, –5.9.

R$_f$ = 0.57 (hexanes).

EI-HRMS (*m/z*): calculated for $C_{13}H_{20}Si_1$ [M$^{+\bullet}$]: 204.1329, found: 204.1327.

methyl(2-methylallyl)(phenyl)(propyl)silane (1c)

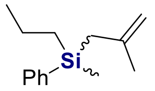

^{1}H NMR (501 MHz, CD_2Cl_2) δ 7.55–7.48 (m, 2H), 7.34 (dd, *J* = 4.9, 1.9 Hz, 3H), 4.57 (dd, *J* = 2.5, 1.4 Hz, 1H), 4.46 (t, *J* = 1.7 Hz, 1H), 1.79 (s, 2H), 1.63–1.56 (m, 3H), 1.43–1.29 (m, 2H), 0.95 (t, *J* = 7.2 Hz, 3H), 0.87–0.78 (m, 2H), 0.30 (s, 3H).

^{13}C NMR (126 MHz, CD_2Cl_2) δ 143.9, 138.8, 134.3, 129.2, 128.0, 109.0, 26.6, 25.4, 18.5, 17.7, 17.2, –4.9.

R$_f$ = 0.60 (hexanes).

CI-HRMS (*m/z*): calculated for $C_{14}H_{21}Si_1$ ([M-H]$^-$): 217.1407, found: 217.1405.

butyl(methyl)(2-methylallyl)(phenyl)silane (1d)

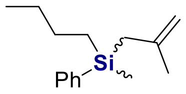

^{1}H NMR (501 MHz, CD_2Cl_2) δ 7.55–7.48 (m, 2H), 7.38–7.30 (m, 3H), 4.57 (dd, *J* = 2.5, 1.3 Hz, 1H), 4.46 (dq, *J* = 2.0, 0.9 Hz, 1H), 1.79 (t, *J* = 0.9 Hz, 2H), 1.59 (t, *J* = 1.1 Hz, 3H), 1.38–1.23 (m, 4H), 0.91–0.78 (m, 5H), 0.30 (s, 3H).

^{13}C NMR (126 MHz, CD_2Cl_2) δ 143.9, 138.8, 134.3, 129.2, 128.0, 108.9, 27.0, 26.5, 26.4, 25.4, 14.2, 13.9, –5.0.

R$_f$ = 0.62 (hexanes).

CI-HRMS (*m/z*): calculated for $C_{15}H_{23}Si_1$ ([M-H]$^-$): 231.1564, found: 231.1561.

methyl(2-methylallyl)(pentyl)(phenyl)silane (1e)

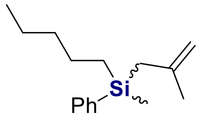

^{1}H NMR (501 MHz, CD_2Cl_2) δ 7.55–7.47 (m, 2H), 7.34 (dd, *J* = 4.9, 1.9 Hz, 3H), 4.57 (dd, *J* = 2.6, 1.3 Hz, 1H), 4.48–4.44 (m, 1H), 1.79 (d, *J* = 0.9 Hz, 2H), 1.59 (t, *J* = 1.1 Hz, 3H), 1.29 (qd, *J* = 6.9, 4.9, 2.5 Hz, 6H), 0.93–0.74 (m, 5H), 0.30 (s, 3H).

^{13}C NMR (126 MHz, CD_2Cl_2) δ 143.9, 138.8, 134.3, 129.2, 128.0, 108.9, 36.3, 26.5, 25.4, 23.8, 22.7, 14.4, 14.2, –4.9.

R$_f$ = 0.64 (hexanes).

CI-HRMS (*m/z*): calculated for $C_{16}H_{27}Si_1$ ([M+H]$^+$): 247.1877, found: 247.1874.

heptyl(methyl)(2-methylallyl)(phenyl)silane (1f)

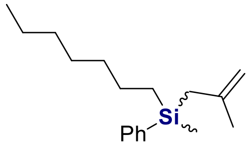

^{1}H NMR (501 MHz, CD_2Cl_2) δ 7.55–7.48 (m, 2H), 7.34 (dd, *J* = 4.9, 1.9 Hz, 3H), 4.57 (dd, *J* = 2.5, 1.3 Hz, 1H), 4.46 (dq, *J* = 1.8, 0.9 Hz, 1H), 1.79 (d, *J* = 1.0 Hz, 2H), 1.59 (t, *J* = 1.1 Hz, 3H), 1.37–1.18 (m, 10H), 0.87 (t, *J* = 7.0 Hz, 3H), 0.84–0.79 (m, 2H), 0.30 (s, 3H).

^{13}C NMR (126 MHz, CD_2Cl_2) δ 143.9, 138.8, 134.3, 129.2, 128.0, 108.9, 34.0, 32.2, 29.3, 26.5, 25.4, 24.1, 23.1, 14.5, 14.3, –4.9.

R_f = 0.61 (hexanes).

CI-HRMS (*m/z*): calculated for $C_{18}H_{29}Si_1$ ($[M-H]^-$): 273.2033, found: 273.2030.

isobutyl(methyl)(2-methylallyl)(phenyl)silane (1g)

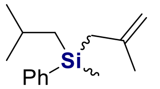

^{1}H NMR (501 MHz, CD_2Cl_2) δ 7.58–7.47 (m, 2H), 7.34 (dd, *J* = 4.7, 1.9 Hz, 3H), 4.56 (dt, *J* = 2.4, 1.3 Hz, 1H), 4.45 (dt, *J* = 2.6, 1.2 Hz, 1H), 1.79 (s, 2H), 1.78–1.72 (m, 1H), 1.56 (d, *J* = 1.3 Hz, 3H), 0.90 (d, *J* = 6.5 Hz, 3H), 0.86 (d, *J* = 6.6 Hz, 3H), 0.85–0.78 (m, 2H), 0.36 (d, *J* = 0.8 Hz, 3H).

^{13}C NMR (126 MHz, CD_2Cl_2) δ 143.9, 139.2, 134.3, 129.2, 128.0, 109.0, 27.4, 26.5, 26.5, 25.4, 25.2, 25.0, –4.2.

R_f = 0.60 (hexanes).

CI-HRMS (*m/z*): calculated for $C_{15}H_{23}Si_1$ ($[M-H]^-$): 231.1564, found: 231.1561.

hexyl(methyl)(2-methylallyl)(p-tolyl)silane (1h)

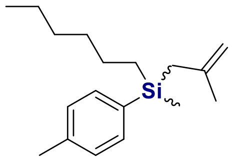

^{1}H NMR (501 MHz, CD_2Cl_2) δ 7.45–7.36 (m, 2H), 7.17 (dt, *J* = 7.4, 0.8 Hz, 2H), 4.56 (dd, *J* = 2.6, 1.4 Hz, 1H), 4.46 (dq, *J* = 2.3, 1.0 Hz, 1H), 2.33 (s, 3H), 1.77 (d, *J* = 1.0 Hz, 2H), 1.61–1.57 (m, 3H), 1.36–1.15 (m, 8H), 0.93–0.83 (m, 3H), 0.79 (ddd, *J* = 10.8, 6.3, 2.2 Hz, 2H), 0.28 (s, 3H).

^{13}C NMR (126 MHz, CD_2Cl_2) δ 144.0, 139.2, 135.0, 134.3, 128.8, 108.8, 33.8, 31.9, 26.6, 25.4, 24.1, 23.0, 21.5, 14.6, 14.3, –4.9.

R_f = 0.67 (hexanes).

CI-HRMS (*m/z*): calculated for $C_{18}H_{31}Si_1$ ($[M+H]^+$): 275.2190, found: 275.2187.

hexyl(4-methoxyphenyl)(methyl)(2-methylallyl)silane (1i)

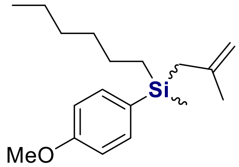

^{1}H NMR (501 MHz, CD_2Cl_2) δ 7.43 (d, *J* = 8.6 Hz, 2H), 6.93 – 6.85 (m, 2H), 4.56 (dd, *J* = 2.5, 1.4 Hz, 1H), 4.45 (dq, *J* = 2.3, 1.0 Hz, 1H), 3.79 (s, 3H), 1.76 (d, *J* = 0.8 Hz, 2H), 1.58 (t, *J* = 1.1 Hz, 3H), 1.36–1.21 (m, 8H), 0.90–0.84 (m, 3H), 0.81–0.76 (m, 2H), 0.27 (s, 3H).

^{13}C NMR (126 MHz, CD_2Cl_2) δ 160.8, 144.1, 135.7, 129.4, 113.8, 108.8, 55.3, 33.8, 31.9, 26.8, 25.4, 24.1, 23.0, 14.7, 14.3, –4.8.

R_f = 0.53 (Ethyl acetate/hexanes = 1:20).

ESI-HRMS (*m/z*): calculated for $C_{18}H_{30}O_1Na_1Si_1$ ($[M+Na]^+$): 313.1958, found: 313.1955.

(4-fluorophenyl)(hexyl)(methyl)(2-methylallyl)silane (1j)

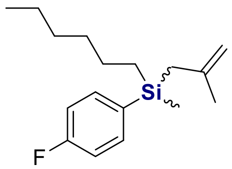

^{1}H NMR (501 MHz, CD_2Cl_2) δ 7.50 (dd, *J* = 8.5, 6.3 Hz, 2H), 7.09–7.02 (m, 2H), 4.57 (dd, *J* = 2.4, 1.4 Hz, 1H), 4.45 (dq, *J* = 2.0, 0.9 Hz, 1H), 1.77 (d, *J* = 0.9 Hz, 2H), 1.58 (t, *J* = 1.1 Hz, 3H), 1.36–1.22 (m, 8H), 0.90–0.84 (m, 3H), 0.83–0.78 (m, 2H), 0.30 (s, 3H).

^{13}C NMR (126 MHz, CD_2Cl_2) δ 164.0 (d, *J* = 248.2 Hz), 143.7, 136.2 (d, *J* = 8.4 Hz), 134.4 (d, *J* = 4.2 Hz), 115.0 (d, *J* = 19.6 Hz), 109.1, 33.7, 31.9, 26.6, 25.4, 24.1, 23.0, 14.5, 14.3, –4.8.

^{19}F NMR (471 MHz, CD_2Cl_2) δ –113.2.

R_f = 0.61 (hexanes).

APPI-HRMS (*m/z*): calculated for $C_{17}H_{28}F_1Si_1$ ($[M+H]^+$): 279.1939, found: 279.1938.

hexyl(methyl)(2-methylallyl)(4-(trifluoromethyl)phenyl)silane (1k)

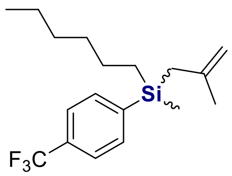

^{1}H NMR (501 MHz, CD_2Cl_2) δ 7.68–7.63 (m, 2H), 7.60 (s, 2H), 4.59 (dd, *J* = 2.4, 1.3 Hz, 1H), 4.46 (dd, *J* = 2.3, 1.0 Hz, 1H), 1.81 (s, 2H), 1.59 (t, *J* = 1.1 Hz, 3H), 1.34–1.20 (m, 8H), 1.03–0.73 (m, 5H), 0.34 (s, 3H).

^{13}C NMR (126 MHz, CD_2Cl_2) δ 144.2, 143.3, 134.7, 124.4 (q, *J* = 248.2 Hz), 109.4, 33.6, 31.9, 26.2, 25.4, 24.0, 23.0, 14.3, –5.1. (due to the low peak intensity, the splitting of carbon NMR is hard to distinguish)

^{19}F NMR (471 MHz, CD_2Cl_2) δ –63.2.

R_f = 0.77 (hexanes).

APPI-HRMS (*m/z*): calculated for $C_{18}H_{27}F_3Si_1$ $[M^{+\bullet}]$: 328.1829, found: 328.1824.

hexyl(3-methoxyphenyl)(methyl)(2-methylallyl)silane (1l)

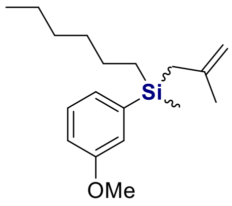

^{1}H NMR (501 MHz, CD_2Cl_2) δ 7.27 (dd, *J* = 8.2, 7.2 Hz, 1H), 7.08 (dt, *J* = 7.2, 1.1 Hz, 1H), 7.04 (dd, *J* = 2.8, 1.0 Hz, 1H), 6.88 (ddd, *J* = 8.3, 2.8, 1.1 Hz, 1H), 4.57 (dt, *J* = 2.8, 1.4 Hz, 1H), 4.47 (dt, *J* = 1.9, 0.9 Hz, 1H), 3.79 (s, 3H), 1.78 (d, *J* = 0.9 Hz, 2H), 1.60 (t, *J* = 1.1 Hz, 3H), 1.38–1.22 (m, 8H), 0.87 (d, *J* = 6.7 Hz, 3H), 0.81 (td, *J* = 5.8, 5.1, 2.1 Hz, 2H), 0.29 (s, 3H).

^{13}C NMR (126 MHz, CD_2Cl_2) δ 159.4, 143.9, 140.5, 129.2, 126.5, 119.8, 114.4, 109.0, 55.4, 33.7, 31.9, 26.5, 25.4, 24.1, 23.0, 14.5, 14.3, –4.9.

R_f = 0.66 (Ethyl acetate/hexanes = 1:20).

ESI-HRMS (*m/z*): calculated for $C_{18}H_{30}O_1Na_1Si_1$ ($[M+Na]^+$): 313.1958, found: 313.1958.

hexyl(methyl)(2-methylallyl)(o-tolyl)silane (1m)

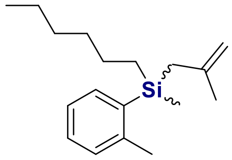

^{1}H NMR (501 MHz, CD_2Cl_2) δ 7.42 (dd, *J* = 7.2, 1.5 Hz, 1H), 7.25 (td, *J* = 7.4, 1.5 Hz, 1H), 7.17–7.10 (m, 2H), 4.55 (dq, *J* = 2.8, 1.4 Hz, 1H), 4.47 (dq, *J* = 2.1, 0.9 Hz, 1H), 2.46 (s, 3H), 1.85 (d, *J* = 1.1 Hz, 2H), 1.61–1.56 (m, 3H), 1.34–1.23 (m, 8H), 0.97–0.80 (m, 5H), 0.36 (s, 3H).

^{13}C NMR (126 MHz, CD_2Cl_2) δ 144.2, 144.1, 136.8, 135.4, 130.2, 129.5, 125.2, 109.0, 33.7, 31.9, 27.0, 25.2, 24.3, 23.4, 23.0, 15.2, 14.3, –3.5.

R_f = 0.59 (hexanes).

APPI-HRMS (*m/z*): calculated for $C_{18}H_{30}Si_1$ $[M^{+\bullet}]$: 274.2111, found: 274.2110.

hexyl(methyl)(2-methylallyl)(naphthalen-2-yl)silane (1n)

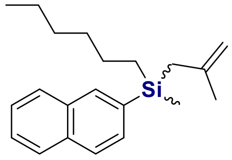

^{1}H NMR (501 MHz, CD_2Cl_2) δ 8.01 (s, 1H), 7.89–7.78 (m, 3H), 7.60 (dd, *J* = 8.2, 1.2 Hz, 1H), 7.52–7.45 (m, 2H), 4.57 (dd, *J* = 2.5, 1.3 Hz, 1H), 4.49 (dd, *J* = 2.4, 1.2 Hz, 1H), 1.87 (d, *J* = 1.0 Hz, 2H), 1.59 (t, *J* = 1.1 Hz, 3H), 1.39–1.18 (m, 8H), 0.95–0.79 (m, 5H), 0.40 (s, 3H).

^{13}C NMR (126 MHz, CD_2Cl_2) δ 143.9, 136.4, 134.9, 134.1, 133.3, 130.7, 128.4, 128.0, 127.1, 126.7, 126.2, 109.0, 33.7, 31.9, 26.6, 25.4, 24.2, 23.0, 14.6, 14.3, –4.9.

R_f = 0.56 (hexanes).

EI-HRMS (*m/z*): calculated for $C_{21}H_{30}Si_1$ $[M^{+\bullet}]$: 310.2111, found: 310.2107.

hexyl(methyl)(2-methylallyl)(thiophen-2-yl)silane (1o)

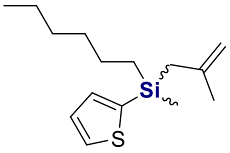

^{1}H NMR (501 MHz, CD_2Cl_2) δ 7.62 (dd, *J* = 4.7, 0.9 Hz, 1H), 7.29 (dd, *J* = 3.3, 0.9 Hz, 1H), 7.19 (dd, *J* = 4.6, 3.3 Hz, 1H), 4.60 (dd, *J* = 2.5, 1.4 Hz, 1H), 4.50 (q, *J* = 1.0 Hz, 1H), 1.82 (dd, *J* = 2.0, 1.0 Hz, 2H), 1.62 (t, *J* = 1.1 Hz, 3H), 1.41–1.21 (m, 8H), 0.93–0.77 (m, 5H), 0.34 (s, 3H).

^{13}C NMR (126 MHz, CD_2Cl_2) δ 143.4, 138.0, 135.2, 131.0, 128.4, 109.3, 33.6, 31.9, 27.4, 25.4, 24.0, 23.0, 15.5, 14.3, –3.6.

R_f = 0.77 (hexanes).

EI-HRMS (*m/z*): calculated for $C_{15}H_{26}S_1Si_1$ [$M^{+\bullet}$]: 266.1519, found: 266.1517.

allyl(methyl)(2-methylallyl)(phenyl)silane (1p)

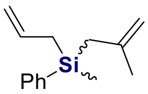

^{1}H NMR (501 MHz, CD_2Cl_2) δ 7.60–7.49 (m, 2H), 7.42–7.31 (m, 3H), 5.80 (ddt, *J* = 16.4, 10.0, 8.1 Hz, 1H), 4.94–4.83 (m, 2H), 4.61 (dd, *J* = 2.5, 1.4 Hz, 1H), 4.51 (dd, *J* = 2.2, 1.2 Hz, 1H), 1.92–1.81 (m, 4H), 1.61 (d, *J* = 1.3 Hz, 3H), 0.34 (s, 3H).

^{13}C NMR (126 MHz, CD_2Cl_2) δ 143.5, 137.7, 134.9, 134.4, 129.5, 128.1, 114.0, 109.5, 26.0, 25.5, 22.5, –5.3.

R_f = 0.65 (hexanes).

APPI-HRMS (*m/z*): calculated for $C_{14}H_{20}Si_1$ [$M^{+\bullet}$]: 216.1329, found: 216.1330.

methyl(2-methylallyl)(phenyl)(prop-1-en-2-yl)silane (1q)

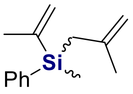

^{1}H NMR (501 MHz, CD_2Cl_2) δ 7.56–7.49 (m, 2H), 7.39–7.31 (m, 3H), 5.72 (dq, *J* = 3.3, 1.7 Hz, 1H), 5.38 (dq, *J* = 2.6, 1.3 Hz, 1H), 4.60 (dd, *J* = 2.5, 1.4 Hz, 1H), 4.51 (dd, *J* = 2.3, 1.1 Hz, 1H), 1.95 (dd, *J* = 2.9, 1.1 Hz, 2H), 1.83 (t, *J* = 1.5 Hz, 3H), 1.62 (t, *J* = 1.1 Hz, 3H), 0.40 (s, 3H).

^{13}C NMR (126 MHz, CD_2Cl_2) δ 145.6, 143.5, 137.3, 134.6, 129.5, 128.1, 127.6, 109.6, 25.3, 25.2, 23.0, –5.2.

R_f = 0.65 (hexanes).

APPI-HRMS (*m/z*): calculated for $C_{14}H_{20}Si_1$ [$M^{+\bullet}$]: 216.1329, found: 216.1330.

methyl(2-methylallyl)(4-methylpent-3-en-1-yl)(phenyl)silane (1r)

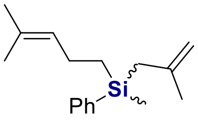

^{1}H NMR (501 MHz, CD_2Cl_2) δ 7.56–7.47 (m, 2H), 7.34 (dd, *J* = 4.7, 2.0 Hz, 3H), 5.16–5.08 (m, 1H), 4.57 (dq, *J* = 2.8, 1.5 Hz, 1H), 4.47 (dq, *J* = 1.8, 0.9 Hz, 1H), 2.00 (q, *J* = 5.6 Hz, 2H), 1.80 (d, *J* = 1.2 Hz, 2H), 1.64 (d, *J* = 1.4 Hz, 3H), 1.59 (t, *J* = 1.2 Hz, 3H), 1.53 (d, *J* = 1.5 Hz, 3H), 0.93–0.79 (m, 2H), 0.32 (s, 3H).

^{13}C NMR (126 MHz, CD_2Cl_2) δ 143.8, 138.6, 134.3, 130.4, 129.3, 128.0, 127.8, 109.0, 26.6, 25.7, 25.4, 22.5, 17.6, 14.9, –4.9.

R_f = 0.51 (hexanes).

CI-HRMS (*m/z*): calculated for $C_{17}H_{27}Si_1$ ($[M+H]^+$): 259.1877, found: 259.1874.

benzyl(4-methoxybutyl)(methyl)(2-methylallyl)silane (1s)

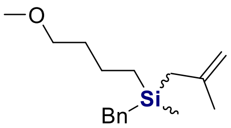

^{1}H NMR (501 MHz, CD_2Cl_2) δ 7.20 (dd, *J* = 8.3, 7.1 Hz, 2H), 7.06 (t, *J* = 7.4 Hz, 1H), 7.04–6.97 (m, 2H), 4.61 (dd, *J* = 2.5, 1.3 Hz, 1H), 4.49 (dq, *J* = 2.7, 0.9 Hz, 1H), 3.32 (s, 2H), 3.28 (s, 3H), 2.14 (s, 2H), 1.70 (t, *J* = 1.1 Hz, 3H), 1.60–1.47 (m, 4H), 1.41–1.21 (m, 2H), 0.54 (ddd, *J* = 10.8, 6.4, 2.9 Hz, 2H), –0.03 (s, 3H).

^{13}C NMR (126 MHz, CD_2Cl_2) δ 143.9, 140.6, 128.6, 128.5, 124.3, 108.9, 72.7, 58.6, 33.9, 25.5, 24.4, 20.6, 13.6, –4.9.

R_f = 0.25 (Ethyl acetate/hexanes = 1:19).

ESI-HRMS (*m/z*): calculated for $C_{17}H_{28}Na_1O_1Si_1$ ($[M+Na]^+$): 299.1802, found: 299.1780.

hexyl(methyl)(2-methylprop-1-en-1-yl)(phenyl)silane (1t)

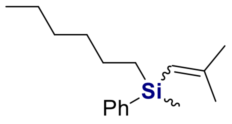

^{1}H NMR (501 MHz, CD_2Cl_2) δ 7.56–7.48 (m, 2H), 7.36–7.28 (m, 3H), 5.35 (hept, J = 0.9 Hz, 1H), 1.89 (d, J = 1.3 Hz, 3H), 1.69 (d, J = 0.8 Hz, 3H), 1.37–1.16 (m, 8H), 0.92–0.73 (m, 5H), 0.34 (s, 3H).

^{13}C NMR (126 MHz, CD_2Cl_2) δ 154.5, 140.0, 134.4, 128.9, 128.0, 121.1, 33.7, 32.0, 29.6, 24.3, 23.9, 23.0, 16.2, 14.3, –2.8.

R_f = 0.48 (hexanes).

CI-HRMS (*m/z*): calculated for $C_{17}H_{27}Si_1$ ($[M-H]^-$): 259.1877, found: 259.1873.

allyl(hexyl)(methyl)(phenyl)silane (1u)

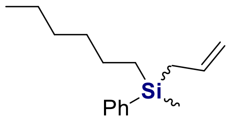

^{1}H NMR (501 MHz, CD_2Cl_2) δ 7.55–7.46 (m, 2H), 7.34 (dd, J = 4.8, 2.0 Hz, 3H), 5.86–5.69 (m, 1H), 4.90–4.77 (m, 2H), 1.79 (dt, J = 8.2, 1.3 Hz, 2H), 1.39–1.17 (m, 8H), 0.87 (t, J = 6.8 Hz, 3H), 0.83–0.73 (m, 2H), 0.27 (s, 3H).

^{13}C NMR (126 MHz, CD_2Cl_2) δ 138.4, 135.2, 134.3, 129.3, 128.0, 113.4, 33.7, 31.9, 24.0, 23.0, 22.5, 14.3, 14.0, –5.4.

R_f = 0.36 (hexanes).

CI-HRMS (*m/z*): calculated for $C_{16}H_{27}Si_1$ ($[M+H]^+$): 247.1877, found: 247.1874.

methyl(2-methylallyl)((S)-2-methylbutyl)(phenyl)silane (1v)

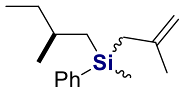

^{1}H NMR (501 MHz, CD_2Cl_2) δ 7.53 (td, J = 4.6, 2.6 Hz, 2H), 7.40–7.29 (m, 3H), 4.56 (dt, J = 2.7, 1.3 Hz, 1H), 4.46 (dq, J = 1.8, 0.9 Hz, 1H), 1.79 (d, J = 1.0 Hz, 2H), 1.57 (t, J = 1.1 Hz, 3H), 1.55–1.47 (m, 1H), 1.37–1.23 (m, 1H), 1.17 (ddt, J = 16.2, 13.4, 7.3 Hz, 1H), 0.97 (ddd, J = 21.1, 14.9, 5.1 Hz, 1H), 0.90–0.77 (m, 6H), 0.71 (ddd, J = 20.0, 14.8, 8.5 Hz, 1H), 0.37 (d, J = 2.2 Hz, 3H).

^{13}C NMR (126 MHz, CD_2Cl_2) δ 143.9, 139.3, 139.1, 134.4, 134.3, 129.2, 128.0, 128.0, 109.1, 35.2, 34.3, 33.6, 33.5, 31.4, 31.4, 30.1, 27.5, 27.4, 25.5, 22.6, 22.6, 22.5, 19.4, 11.6, 11.6, –4.0, –4.3.

R_f = 0.52 (hexanes).

CI-HRMS (*m/z*): calculated for $C_{16}H_{27}Si_1$ ($[M+H]^+$): 247.1877, found: 247.1874.

Note: the titled product was obtained as a mixture of diastereomers. For this substrate, d.r. was determined to be ~ 1:1 by nmr analysis.

4. Substrate scope for the DYKAT of racemic silanes

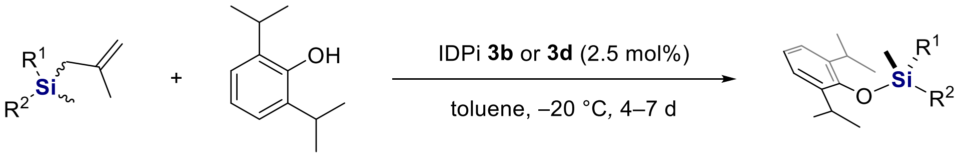

rac-**1** Propofol **2** **4**

General procedure for asymmetric synthesis of silyl ethers:

To a flame-dried Schlenk tube, IDPi **3b** or **3d** (2.5 mol%) in toluene (0.1–0.4 M) was added under Ar. The solution was lowered to –20 °C for 10 min, then propofol **2** (1.5 equiv., 56 μL, 0.3 mmol) and the *rac*-silane starting materials **1** (0.2 mmol) were added afterwards. The reaction mixture was stirred for an additional 4–7 d at the indicated temperature. After *rac*-**1** was fully consumed, as monitored by TLC, the reaction mixture was treated with triethylamine. All volatiles were removed in vacuo and the NMR yield was determined using CH_2Br_2 as internal standard. The residue was purified by column chromatography with silica gel, which was pre-neutralized by triethylamine, to afford the desired silyl ethers **4**.

Analytical data of products 4

(*S*)-(2,6-diisopropylphenoxy)(hexyl)(methyl)(phenyl)silane 4a

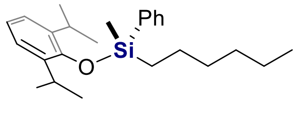

The titled product was purified by column chromatography with hexanes as eluent to afford **4a** as a colorless oil (53.6 mg, 70% yield).

^{1}H NMR (501 MHz, CD_2Cl_2) δ 7.62–7.55 (m, 2H), 7.42–7.33 (m, 3H), 7.03–6.97 (m, 2H), 6.92 (dd, *J* = 8.2, 6.9 Hz, 1H), 3.16 (p, *J* = 6.9 Hz, 2H), 1.43–1.29 (m, 4H), 1.25 (tt, *J* = 4.4, 1.8 Hz, 4H), 1.07 (s, 3H), 1.06 (s, 3H), 1.06 (s, 3H), 1.05 (s, 3H), 1.04–0.97 (m, 2H), 0.88–0.82 (m, 3H), 0.46 (s, 3H).

^{13}C NMR (126 MHz, CD_2Cl_2) δ 150.1, 139.5, 137.5, 133.9, 130.0, 128.1, 123.7, 122.4, 33.5, 31.8, 27.4, 23.5, 23.4, 22.9, 16.7, 14.3, –2.8.

R_f = 0.40 (hexanes).

EI-HRMS (*m/z*): calculated for $C_{25}H_{38}O_1Si_1$ [$M^{+\bullet}$]: 382.2686, found: 382.2685.

HPLC (OJ-3R, MeOH : H_2O = 80:20, 1 mL/min, 298 K, 220 nm): t_{R1} = 40.0 min, t_{R2} = 35.9 min, e.r. = 99:1.

$[\alpha]_D^{25}$ = 7.3 (*c* 0.88, $CHCl_3$).

(2,6-diisopropylphenoxy)(ethyl)(methyl)(phenyl)silane 4b

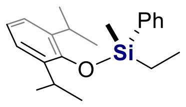

The titled product was purified by column chromatography with hexanes as eluent to afford **4b** as a colorless oil (43.9 mg, 67% yield).

^{1}H NMR (501 MHz, CD_2Cl_2) δ 7.63–7.57 (m, 2H), 7.44–7.32 (m, 3H), 7.04–6.98 (m, 2H), 6.92 (dd, *J* = 8.2, 6.9 Hz, 1H), 3.16 (p, *J* = 6.9 Hz, 2H), 1.06 (dd, *J* = 6.8, 3.7 Hz, 12H), 1.05–0.92 (m, 5H), 0.46 (s, 3H).

^{13}C NMR (126 MHz, CD_2Cl_2) δ 150.1, 139.5, 137.1, 133.9, 130.0, 128.1, 123.7, 122.4, 27.4, 23.5, 23.4, 8.5, 6.9, –3.4.

R_f = 0.34 (hexanes).

EI-HRMS (*m/z*): calculated for $C_{21}H_{30}O_1Si_1$ [$M^{+\bullet}$]: 326.2060, found: 326.2058.

HPLC (OJ-3R, Acetonitrile/Water = 70:30, 1 mL/min, 298 K, 220 nm): t_{R1} = 6.6 min, t_{R2} = 9.5 min, e.r. = 85:15.

$[\alpha]_D^{25}$ = 6.6 (*c* 1.0, $CHCl_3$).

(2,6-diisopropylphenoxy)(methyl)(phenyl)(propyl)silane 4c

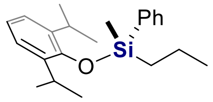

The titled product was purified by column chromatography with hexanes as eluent to afford **4c** as a colorless oil (49.7 mg, 73% yield).

^{1}H NMR (501 MHz, CD_2Cl_2) δ 7.63–7.57 (m, 2H), 7.44–7.32 (m, 3H), 7.04–6.98 (m, 2H), 6.92 (dd, *J* = 8.2, 6.9 Hz, 1H), 3.16 (p, *J* = 6.9 Hz, 2H), 1.47–1.36 (m, 2H), 1.08 (s, 3H), 1.06 (s, 6H), 1.05 (s, 4H), 1.04–1.00 (m, 1H), 0.97 (t, *J* = 7.3 Hz, 3H), 0.47 (s, 3H).

^{13}C NMR (126 MHz, CD_2Cl_2) δ 150.1, 139.5, 137.5, 133.9, 130.0, 128.1, 123.7, 122.4, 27.4, 23.5, 23.4, 19.3, 18.4, 17.1, –2.8.

R_f = 0.22 (hexanes).

ESI-HRMS (*m/z*): calculated for $C_{22}H_{32}O_1Si_1Na_1$ ($[M+Na]^+$): 363.2115, found: 363.2114.

HPLC (OJ-3R, MeOH : H_2O = 80:20, 1 mL/min, 298 K, 220 nm): t_{R1} = 16.5 min, t_{R2} = 19.2 min, e.r. = 92:8.

$[\alpha]_D^{25}$ = 11.2 (*c* 0.43, $CHCl_3$).

butyl(2,6-diisopropylphenoxy)(methyl)(phenyl)silane 4d

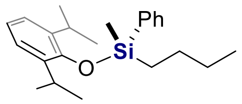

The titled product was purified by column chromatography with hexanes as eluent to afford **4d** as a colorless oil (53.2 mg, 75% yield).

^{1}H NMR (501 MHz, CD_2Cl_2) δ 7.59 (dd, J = 7.9, 1.6 Hz, 2H), 7.42–7.32 (m, 3H), 7.03–6.98 (m, 2H), 6.92 (dd, J = 8.2, 6.9 Hz, 1H), 3.16 (p, J = 6.9 Hz, 2H), 1.43–1.29 (m, 4H), 1.07 (s, 3H), 1.06 (s, 3H), 1.06 (s, 3H), 1.05 (s, 3H), 1.04–0.97 (m, 2H), 0.90–0.84 (m, 3H), 0.46 (s, 3H).

^{13}C NMR (126 MHz, CD_2Cl_2) δ 150.1, 139.5, 137.5, 133.9, 130.0, 128.1, 123.7, 122.4, 27.4, 26.8, 25.7, 23.5, 23.4, 16.4, 13.8, –2.9.

R_f = 0.19 (hexanes).

ESI-HRMS (*m/z*): calculated for $C_{23}H_{34}O_1Si_1Na_1$ ($[M+Na]^+$): 377.2271, found: 377.2270.

HPLC (OJ-3R, Acetonitrile/Water = 60:40, 1.0 mL/min, 298 K, 220 nm): t_{R1} = 17.4 min, t_{R2} = 20.3 min, e.r. = 96:4.

$[\alpha]_D^{25}$ = 8.6 (c 1.0, $CHCl_3$).

(2,6-diisopropylphenoxy)(methyl)(pentyl)(phenyl)silane 4e

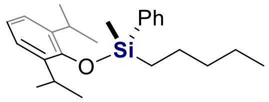

The titled product was purified by column chromatography with hexanes as eluent to afford **4e** as a colorless oil (53.2 mg, 72% yield).

^{1}H NMR (501 MHz, CD_2Cl_2) δ 7.62–7.56 (m, 2H), 7.42–7.33 (m, 3H), 7.03–6.98 (m, 2H), 6.94–6.88 (m, 1H), 3.16 (p, J = 6.9 Hz, 2H), 1.43–1.35 (m, 2H), 1.34–1.23 (m, 4H), 1.07 (s, 3H), 1.06 (s, 3H), 1.06 (s, 3H), 1.05 (s, 3H), 1.04–0.94 (m, 2H), 0.85 (t, J = 7.1 Hz, 3H), 0.46 (s, 3H).

^{13}C NMR (126 MHz, CD_2Cl_2) δ 150.1, 139.5, 137.5, 133.9, 130.0, 128.1, 123.7, 122.3, 36.1, 27.4, 23.5, 23.4, 23.1, 22.6, 16.6, 14.1, –2.8.

R_f = 0.26 (hexanes).

ESI-HRMS (*m/z*): calculated for $C_{24}H_{36}O_1Si_1Na_1$ ($[M+Na]^+$): 391.2428, found: 391.2426.

HPLC (OJ-3R, Acetonitrile/Water = 60:40, 1.0 mL/min, 298 K, 220 nm): t_{R1} = 20.9 min, t_{R2} = 23.9 min, e.r. = 98:2.

$[\alpha]_D^{25}$ = 6.2 (c 0.87, $CHCl_3$).

(2,6-diisopropylphenoxy)(heptyl)(methyl)(phenyl)silane 4f

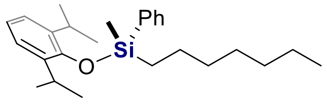

The titled product was purified by column chromatography with hexanes as eluent to afford **4f** as a colorless oil (67 mg, 84% yield).

^{1}H NMR (501 MHz, CD_2Cl_2) δ 7.65–7.57 (m, 2H), 7.44–7.34 (m, 3H), 7.02 (d, J = 7.5 Hz, 2H), 6.93 (dd, J = 8.3, 6.9 Hz, 1H), 3.18 (p, J = 6.9 Hz, 2H), 1.47–1.18 (m, 11H), 1.09 (s, 3H), 1.08 (s, 2H), 1.07 (s, 3H), 1.06 (s, 3H), 1.06–0.99 (m, 2H), 0.88 (t, J = 7.0 Hz, 3H), 0.48 (s, 3H).

^{13}C NMR (126 MHz, CD_2Cl_2) δ 150.1, 139.5, 137.5, 133.9, 130.0, 128.1, 123.7, 122.4, 33.8, 32.2, 29.3, 27.4, 23.5, 23.5, 23.4, 23.1, 16.7, 14.3, –2.8.

R_f = 0.24 (hexanes).

ESI-HRMS (*m/z*): calculated for $C_{26}H_{40}O_1Na_1Si_1$ ($[M+Na]^+$): 419.2741, found: 419.2743.

HPLC (OJ-3R, MeOH : H_2O = 80:20, 1 mL/min, 298 K, 220 nm): t_{R1} = 35.5 min, t_{R2} = 31.2 min, e.r. = 99:1.

$[\alpha]_D^{25}$ = 5.8 (c 1.73, $CHCl_3$).

(2,6-diisopropylphenoxy)(isobutyl)(methyl)(phenyl)silane 4g

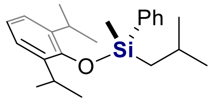

The titled product was purified by column chromatography with hexanes as eluent to afford **4g** as a colorless oil (67.2 mg, 95% yield).

^{1}H NMR (501 MHz, CD_2Cl_2) δ 7.64–7.56 (m, 2H), 7.43–7.33 (m, 3H), 7.04–6.99 (m, 2H), 6.92 (dd, *J* = 8.2, 6.9 Hz, 1H), 3.16 (p, *J* = 6.9 Hz, 2H), 1.84 (dq, *J* = 13.3, 6.6 Hz, 1H), 1.13 (dd, *J* = 15.0, 6.8 Hz, 2H), 1.08 (s, 3H), 1.06 (s, 3H), 1.05 (s, 3H), 1.03 (s, 3H), 0.95 (d, *J* = 6.5 Hz, 3H), 0.93 (d, *J* = 6.6 Hz, 3H), 0.50 (s, 3H).

^{13}C NMR (126 MHz, CD_2Cl_2) δ 150.1, 139.5, 137.8, 133.9, 130.0, 128.1, 123.7, 122.3, 27.4, 27.0, 26.5, 26.3, 24.6, 23.5, 23.4, –2.0.

R_f = 0.26 (hexanes).

ESI-HRMS (*m/z*): calculated for $C_{23}H_{34}O_1Si_1Na_1$ ($[M+Na]^+$): 377.2271, found: 377.2270.

HPLC (OJ-3R, Acetonitrile/Water = 60:40, 1.0 mL/min, 298 K, 220 nm): t_{R1} = 16.1 min, t_{R2} = 18.0 min, e.r. = 98:2.

$[\alpha]_D^{25}$ = 20.4 (*c* 1.54, $CHCl_3$).

(2,6-diisopropylphenoxy)(hexyl)(methyl)(p-tolyl)silane 4h

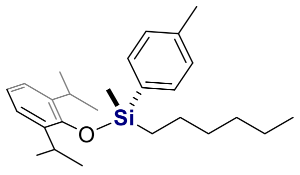

The titled product was purified by column chromatography with hexanes as eluent to afford **4h** as a colorless oil (66.8 mg, 84% yield).

^{1}H NMR (501 MHz, CD_2Cl_2) δ 7.51–7.45 (m, 2H), 7.19 (dt, *J* = 7.3, 0.8 Hz, 2H), 7.03–6.99 (m, 2H), 6.92 (dd, *J* = 8.2, 6.9 Hz, 1H), 3.19 (p, *J* = 6.9 Hz, 2H), 2.36 (s, 3H), 1.42–1.31 (m, 4H), 1.26 (tt, *J* = 5.5, 3.4 Hz, 4H), 1.09 (s, 3H), 1.08 (s, 3H), 1.07 (s, 3H), 1.07 (s, 3H), 1.02 (q, *J* = 8.0 Hz, 2H), 0.91–0.83 (m, 3H), 0.46 (s, 3H).

^{13}C NMR (126 MHz, CD_2Cl_2) δ 150.2, 140.0, 139.5, 134.0, 133.8, 128.9, 123.7, 122.3, 33.6, 31.9, 27.4, 23.5, 23.5, 23.4, 23.0, 21.7, 16.8, 14.3, –2.7.

R_f = 0.26 (hexanes).

EI-HRMS (*m/z*): calculated for $C_{26}H_{40}O_1Si_1$ $[M^{+\bullet}]$: 396.2843, found: 396.2842.

HPLC (OJ-3R, Acetonitrile/Water = 60:40, 1.0 mL/min, 298 K, 220 nm): t_{R1} = 29.2 min, t_{R2} = 31.8 min, e.r. = 98:2.

$[\alpha]_D^{25}$ = 7.8 (*c* 1.0, $CHCl_3$).

(2,6-diisopropylphenoxy)(hexyl)(4-methoxyphenyl)(methyl)silane 4i

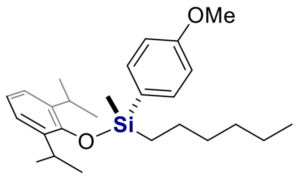

The titled product was purified by column chromatography with hexanes as eluent to afford **4i** as a colorless oil (71.7 mg, 87% yield).

^{1}H NMR (501 MHz, CD_2Cl_2) δ 7.55–7.49 (m, 2H), 7.02 (d, *J* = 7.6 Hz, 2H), 6.92 (t, *J* = 8.2 Hz, 3H), 3.81 (s, 3H), 3.19 (p, *J* = 6.9 Hz, 2H), 1.36 (dtd, *J* = 16.8, 8.7, 8.3, 4.3 Hz, 4H), 1.27 (tq, *J* = 5.1, 2.2 Hz, 4H), 1.10 (s, 3H), 1.09 (s, 3H), 1.08 (s, 3H), 1.07 (s, 3H), 1.03 (d, *J* = 8.4 Hz, 2H), 0.91–0.85 (m, 3H), 0.46 (s, 3H).

^{13}C NMR (126 MHz, CD_2Cl_2) δ 161.4, 150.2, 139.5, 135.5, 128.4, 123.7, 122.3, 113.8, 55.4, 33.6, 31.9, 27.4, 23.6, 23.5, 23.5, 23.0, 16.8, 14.3, –2.7.

R_f = 0.24 (Ethyl acetate/hexanes = 1:19).

ESI-HRMS (*m/z*): calculated for $C_{26}H_{40}O_2Na_1Si_1$ ($[M+Na]^+$): 435.2690, found: 435.2689.

HPLC (OJ-3R, Acetonitrile/Water = 60:40, 1.0 mL/min, 298 K, 220 nm): t_{R1} = 27.8 min, t_{R2} = 31.5 min, e.r. = 99:1.

$[\alpha]_D^{25}$ = 8.8 (*c* 0.93, $CHCl_3$).

(2,6-diisopropylphenoxy)(4-fluorophenyl)(hexyl)(methyl)silane 4j

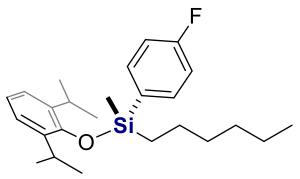

The titled product was purified by column chromatography with hexanes as eluent to afford **4j** as a colorless oil (61.7 mg, 77% yield).

^{1}H NMR (501 MHz, CD_2Cl_2) δ 7.63–7.56 (m, 2H), 7.12–7.05 (m, 2H), 7.04–7.00 (m, 2H), 6.93 (dd, *J* = 8.2, 6.9 Hz, 1H), 3.13 (p, *J* = 6.9 Hz, 2H), 1.40–1.30 (m, 4H), 1.28–1.21 (m, 4H), 1.08 (s, 3H), 1.07 (s, 3H), 1.07 (s, 3H), 1.06 (s, 3H), 1.04–0.97 (m, 2H), 0.87 (t, *J* = 6.8 Hz, 3H), 0.46 (s, 3H).

^{13}C NMR (126 MHz, CD_2Cl_2) δ 164.4 (d, *J* = 248.9 Hz), 150.0, 139.4, 136.0 (d, *J* = 7.6 Hz), 133.4 (d, *J* = 4.0 Hz), 123.8, 122.5, 115.2 (d, *J* = 19.8 Hz), 33.5, 31.8, 27.4, 23.5, 23.4, 23.4, 22.9, 16.7, 14.3, –2.8.

^{19}F NMR (471 MHz, CD_2Cl_2) δ –111.7.

R_f = 0.43 (hexanes).

ESI-HRMS (*m/z*): calculated for $C_{25}H_{37}O_1Na_1F_1Si_1$ ($[M+Na]^+$): 423.2490, found: 423.2487.

HPLC (OJ-3R, MeOH : H_2O = 80:20, 1 mL/min, 298 K, 220 nm): t_{R1} = 34.6 min, t_{R2} = 30.6 min, e.r. = 96.5:3.5.

$[\alpha]_D^{25}$ = 7.0 (*c* 1.0, $CHCl_3$).

(2,6-diisopropylphenoxy)(hexyl)(methyl)(4-(trifluoromethyl)phenyl)silane 4k

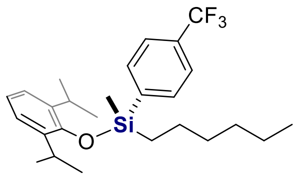

The titled product was purified by column chromatography with hexanes as eluent to afford **4k** as a colorless oil (70.5 mg, 78% yield).

^{1}H NMR (501 MHz, CD_2Cl_2) δ 7.74 (d, *J* = 7.7 Hz, 2H), 7.63 (d, *J* = 7.7 Hz, 2H), 7.04–7.00 (m, 2H), 6.94 (dd, *J* = 8.3, 6.9 Hz, 1H), 3.09 (p, *J* = 6.8 Hz, 2H), 1.40–1.14 (m, 9H), 1.06 (dd, *J* = 6.9, 4.8 Hz, 13H), 0.90–0.81 (m, 3H), 0.47 (s, 3H).

^{13}C NMR (126 MHz, CD_2Cl_2) δ 149.8, 142.7, 139.4, 134.2, 131.7, 125.8, 124.6 (q, *J* = 3.8 Hz), 123.8, 122.7, 33.4, 31.8, 27.5, 23.5, 23.4, 23.2, 22.9, 16.6, 14.2, –2.9.

^{19}F NMR (471 MHz, CD_2Cl_2) δ –63.3.

R_f = 0.43 (hexanes).

EI-HRMS (*m/z*): calculated for $C_{26}H_{37}O_1Si_1F_3$ $[M^{+\bullet}]$: 450.2560, found: 450.2560.

HPLC (OJ-3R, MeOH : H_2O = 80:20, 1 mL/min, 298 K, 220 nm): t_{R1} = 35.5 min, t_{R2} = 31.3 min, e.r. = 98:2.

$[\alpha]_D^{25}$ = 7.8 (*c* 1.0, $CHCl_3$).

(2,6-diisopropylphenoxy)(hexyl)(3-methoxyphenyl)(methyl)silane 4l

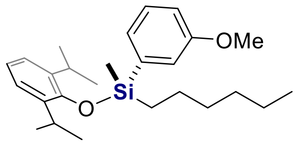

The titled product was purified by column chromatography with hexanes as eluent to afford **4l** as a colorless oil (68.4 mg, 83% yield).

^{1}H NMR (501 MHz, CD_2Cl_2) δ 7.29 (dd, *J* = 8.2, 7.2 Hz, 1H), 7.15 (dt, *J* = 7.2, 1.0 Hz, 1H), 7.07 (dd, *J* = 2.8, 1.0 Hz, 1H), 7.01 (d, *J* = 7.5 Hz, 2H), 6.95–6.90 (m, 2H), 3.74 (s, 3H), 3.17 (p, *J* = 6.9 Hz, 2H), 1.43–1.30 (m, 4H), 1.30–1.21 (m, 4H), 1.08 (s, 3H), 1.08 (s, 3H), 1.07 (s, 3H), 1.06 (s, 3H), 1.05–0.97 (m, 2H), 0.88–0.84 (m, 3H), 0.46 (s, 3H).

^{13}C NMR (126 MHz, CD_2Cl_2) δ 159.4, 150.1, 139.5, 139.0, 129.3, 126.1, 123.7, 122.4, 118.8, 115.7, 55.4, 33.5, 31.8, 27.4, 23.5, 23.4, 23.4, 22.9, 16.8, 14.3, –2.7.

R_f = 0.47 (Ethyl acetate/hexanes = 1:19).

ESI-HRMS (*m/z*): calculated for $C_{26}H_{40}O_2Si_1Na_1$ ($[M+Na]^+$): 435.2690, found: 435.2692.

HPLC (IB-N3, CO_2, BP, 150 bar, 50 °C, 2.0 mL / min, 313 K, 19.0 MPa UV, 220 nm 0.5 mL/min 10mM NH_4AC in MeOH): t_{R1} = 4.8 min, t_{R2} = 4.2 min, e.r. = 98:2.

$[\alpha]_D^{25}$ = 5.8 (*c* 2.2, $CHCl_3$).

(2,6-diisopropylphenoxy)(hexyl)(methyl)(o-tolyl)silane 4m

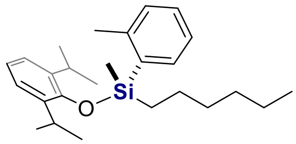

The titled product was purified by column chromatography with hexanes as eluent to afford **4m** as a colorless oil (59.3 mg, 75% yield).

^1H NMR (501 MHz, CD_2Cl_2) δ 7.54 (dd, *J* = 7.3, 1.5 Hz, 1H), 7.31 (td, *J* = 7.5, 1.5 Hz, 1H), 7.22–7.15 (m, 2H), 7.02 (d, *J* = 7.8 Hz, 2H), 6.93 (dd, *J* = 8.2, 7.0 Hz, 1H), 3.12 (p, *J* = 6.9 Hz, 2H), 2.53 (s, 3H), 1.35–1.18 (m, 10H), 1.06 (s, 3H), 1.04 (s, 6H), 1.03 (s, 3H), 0.86 (t, *J* = 6.9 Hz, 3H), 0.43 (s, 3H).

^{13}C NMR (126 MHz, CD_2Cl_2) δ 150.3, 144.0, 139.5, 136.5, 134.6, 130.3, 130.1, 125.1, 123.7, 122.3, 33.5, 31.8, 27.5, 23.6, 23.5, 23.4, 23.0, 22.9, 17.3, 14.3, –2.2.

R_f = 0.25 (hexanes).

ESI-HRMS (*m/z*): calculated for $C_{26}H_{40}O_1Na_1Si_1$ ($[M+Na]^+$): 419.2741, found: 419.2739.

HPLC (OJ-3R, MeOH : H_2O = 80:20, 1 mL/min, 298 K, 220 nm): t_{R1} = 26.4 min, t_{R2} = 24.2 min, e.r. = 71:29.

$[\alpha]_D^{25}$ = 3.0 (*c* 1.2, $CHCl_3$).

(2,6-diisopropylphenoxy)(hexyl)(methyl)(naphthalen-2-yl)silane 4n

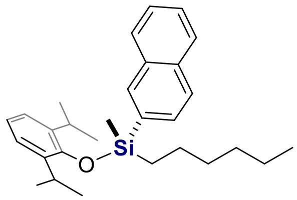

The titled product was purified by column chromatography with hexanes as eluent to afford **4n** as a colorless oil (74.9 mg, 87% yield).

^1H NMR (501 MHz, CD_2Cl_2) δ 8.12 (s, 1H), 7.88–7.80 (m, 3H), 7.67 (dd, *J* = 8.2, 1.2 Hz, 1H), 7.50 (ddd, *J* = 7.1, 4.8, 1.5 Hz, 2H), 7.01 (d, *J* = 7.5 Hz, 2H), 6.92 (dd, *J* = 8.3, 6.9 Hz, 1H), 3.21 (p, *J* = 6.9 Hz, 2H), 1.44–1.10 (m, 10H), 1.06 (t, *J* = 6.6 Hz, 12H), 0.87–0.80 (m, 3H), 0.53 (s, 3H).

^{13}C NMR (126 MHz, CD_2Cl_2) δ 150.1, 139.5, 135.1, 134.8, 134.4, 133.3, 130.0, 128.6, 128.0, 127.2, 127.0, 126.3, 123.8, 122.4, 33.5, 31.8, 27.5, 23.6, 23.4, 22.9, 16.8, 14.2, –2.8.

R_f = 0.29 (hexanes).

EI-HRMS (*m/z*): calculated for $C_{29}H_{40}O_1Si_1$ $[M^{+\bullet}]$: 432.2843, found: 432.2841.

HPLC (OJ-3R, Acetonitrile/Water = 70:30, 1.0 mL/min, 298 K, 220 nm): t_{R1} = 16.3 min, t_{R2} = 19.9 min, e.r. = 98:2.

$[\alpha]_D^{25}$ = 9.6 (*c* 1.0, $CHCl_3$).

(2,6-diisopropylphenoxy)(hexyl)(methyl)(thiophen-2-yl)silane 4o

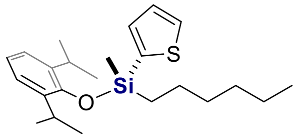

The titled product was purified by column chromatography with hexanes as eluent to afford **4o** as a colorless oil (64.4 mg, 83% yield).

^1H NMR (501 MHz, CD_2Cl_2) δ 7.64 (dd, *J* = 4.7, 0.9 Hz, 1H), 7.35 (dd, *J* = 3.4, 0.9 Hz, 1H), 7.19 (dd, *J* = 4.7, 3.4 Hz, 1H), 7.06–6.99 (m, 2H), 6.93 (dd, *J* = 8.3, 6.9 Hz, 1H), 3.20 (p, *J* = 6.8 Hz, 2H), 1.49–1.17 (m, 10H), 1.10 (dd, *J* = 6.9, 5.2 Hz, 12H), 0.90–0.83 (m, 3H), 0.56 (s, 3H).

^{13}C NMR (126 MHz, CD_2Cl_2) δ 149.8, 139.5, 136.4, 135.9, 131.7, 128.3, 123.8, 122.6, 33.4, 31.9, 27.5, 23.5, 23.5, 23.4, 22.9, 17.5, 14.3, –1.4.

R_f = 0.35 (hexanes).

EI-HRMS (*m/z*): calculated for $C_{23}H_{36}O_1S_1Si_1$ $[M^{+\bullet}]$: 388.2251, found: 388.2250.

HPLC (OJ-3R, MeOH : H_2O = 80:20, 1 mL/min, 298 K, 220 nm): t_{R1} = 25.6 min, t_{R2} = 23.6 min, e.r. = 98:2.

$[\alpha]_D^{25}$ = 6.4 (*c* 1.0, $CHCl_3$).

allyl(2,6-diisopropylphenoxy)(methyl)(phenyl)silane 4p

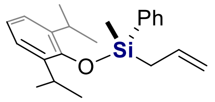

The titled product was purified by column chromatography with hexanes as eluent to afford **4p** as a colorless oil (51.4 mg, 76% yield).

^{1}H NMR (501 MHz, CD_2Cl_2) δ 7.64–7.57 (m, 2H), 7.45–7.34 (m, 3H), 7.06–6.98 (m, 2H), 6.94 (dd, *J* = 8.3, 6.9 Hz, 1H), 5.77 (ddt, *J* = 16.9, 10.1, 8.0 Hz, 1H), 4.96–4.83 (m, 2H), 3.17 (p, *J* = 6.9 Hz, 2H), 2.16–2.01 (m, 2H), 1.08 (d, *J* = 2.8 Hz, 6H), 1.07 (d, *J* = 2.8 Hz, 6H), 0.50 (s, 3H).

^{13}C NMR (126 MHz, CD_2Cl_2) δ 149.9, 139.4, 136.6, 134.0, 133.5, 130.2, 128.1, 123.8, 122.6, 115.0, 27.4, 24.5, 23.5, 23.4, −3.3.

R_f = 0.28 (hexanes).

ESI-HRMS (*m/z*): calculated for $C_{22}H_{30}O_1Si_1Na_1$ ([M+Na]$^+$): 361.1958, found: 361.1959.

HPLC (OJ-3R, MeOH : H_2O = 80:20, 1 mL/min, 298 K, 220 nm): t_{R1} = 15.3 min, t_{R2} = 17.4 min, e.r. = 95:5.

$[\alpha]_D^{25}$ = –12.9 (*c* 0.9, $CHCl_3$).

(2,6-diisopropylphenoxy)(methyl)(phenyl)(prop-1-en-2-yl)silane 4q

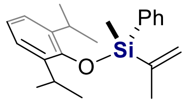

The titled product was purified by column chromatography with hexanes as eluent to afford **4q** as a colorless oil (41.2 mg, 61% yield).

^{1}H NMR (501 MHz, CD_2Cl_2) δ 7.65–7.61 (m, 2H), 7.44–7.36 (m, 3H), 7.06–7.00 (m, 2H), 6.94 (dd, *J* = 8.3, 6.9 Hz, 1H), 5.79 (dd, *J* = 3.1, 1.6 Hz, 1H), 5.53 (dd, *J* = 3.0, 1.4 Hz, 1H), 3.17 (p, *J* = 6.8 Hz, 2H), 1.87 (t, *J* = 1.5 Hz, 3H), 1.07 (s, 3H), 1.06 (s, 6H), 1.05 (s, 3H), 0.51 (s, 3H).

^{13}C NMR (126 MHz, CD_2Cl_2) δ 150.1, 145.0, 139.6, 136.5, 134.2, 130.2, 128.8, 128.2, 123.8, 122.6, 27.4, 23.5, 23.5, 22.1, −3.5.

R_f = 0.22 (hexanes).

EI-HRMS (*m/z*): calculated for $C_{22}H_{30}O_1Si_1$ [$M^{+\bullet}$]: 338.2060, found: 338.2058.

HPLC (OJ-3R, MeOH : H_2O = 80:20, 1 mL/min, 298 K, 220 nm): t_{R1} = 15.8 min, t_{R2} = 19.6 min, e.r. = 86:14.

$[\alpha]_D^{25}$ = –2.3 (*c* 1.0, $CHCl_3$).

(2,6-diisopropylphenoxy)(methyl)(4-methylpent-3-en-1-yl)(phenyl)silane 4r

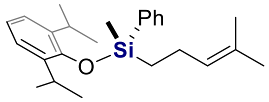

The titled product was purified by column chromatography with hexanes as eluent to afford **4r** as a colorless oil (55.7 mg, 73% yield).

^{1}H NMR (501 MHz, CD_2Cl_2) δ 7.59 (dt, *J* = 6.5, 1.6 Hz, 2H), 7.37 (ddd, *J* = 12.2, 7.5, 5.8 Hz, 3H), 7.01 (d, *J* = 7.5 Hz, 2H), 6.92 (dd, *J* = 8.2, 6.9 Hz, 1H), 5.12 (ddt, *J* = 7.0, 5.7, 1.4 Hz, 1H), 3.16 (p, *J* = 6.8 Hz, 2H), 2.11–1.98 (m, 2H), 1.64 (d, *J* = 1.5 Hz, 3H), 1.50 (d, *J* = 1.3 Hz, 3H), 1.06 (dd, *J* = 6.9, 5.6 Hz, 14H), 0.48 (s, 3H).

^{13}C NMR (126 MHz, CD_2Cl_2) δ 150.1, 139.5, 137.3, 133.9, 130.8, 130.0, 128.1, 127.3, 123.7, 122.4, 30.1, 27.4, 25.7, 23.5, 23.4, 21.9, 17.6, 17.1, –2.7.

R_f = 0.24 (hexanes).

EI-HRMS (*m/z*): calculated for $C_{25}H_{36}O_1Si_1$ [$M^{+\bullet}$]: 380.2530, found: 380.2529.

HPLC (OJ-3R, Acetonitrile/Water = 70:30, 1.0 mL/min, 298 K, 220 nm): t_{R1} = 8.0 min, t_{R2} = 9.5 min, e.r. = 97:3.

$[\alpha]_D^{25}$ = 4.6 (*c* 1.0, $CHCl_3$).

benzyl(2,6-diisopropylphenoxy)(4-methoxybutyl)(methyl)silane 4s

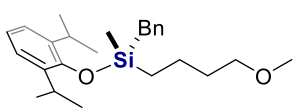

The titled product was purified by column chromatography with ethyl acetate and hexanes as eluent to afford **4s** as a colorless oil (56.0 mg, 70% yield).

^{1}H NMR (501 MHz, CD_2Cl_2) δ 7.18 (dd, J = 8.2, 7.1 Hz, 2H), 7.06 (dd, J = 10.3, 7.5 Hz, 3H), 7.01–6.92 (m, 3H), 3.29 (t, J = 6.5 Hz, 2H), 3.26 (s, 3H), 3.23–3.15 (m, 2H), 2.33 (s, 2H), 1.58–1.48 (m, 2H), 1.46–1.31 (m, 2H), 1.16 (dd, J = 6.8, 4.0 Hz, 12H), 0.74 (dt, J = 10.7, 5.8 Hz, 2H), 0.21 (s, 3H).

^{13}C NMR (126 MHz, CD_2Cl_2) δ 150.0, 139.4, 139.0, 128.9, 128.7, 124.7, 123.8, 122.5, 72.6, 58.6, 54.3, 54.1, 53.6, 53.4, 33.6, 27.4, 26.0, 23.6, 23.6, 20.1, 15.7, –2.6.

R_f = 0.40 (Acetone/pentane = 1:19).

ESI-HRMS (m/z): calculated for $C_{25}H_{38}Na_1O_2Si_1$ ($[M+Na]^+$): 421.2533, found: 421.2531.

HPLC (OJ-3R, MeOH : H_2O = 80:20, 1 mL/min, 298 K, 220 nm): t_{R1} = 28.3 min, t_{R2} = 25.4 min, e.r. = 75:25.

$[\alpha]_D^{25}$ = 0.6 (c 1.0, $CHCl_3$).

Procedures for asymmetric synthesis of silyl ethers bearing a carbon stereocenter:

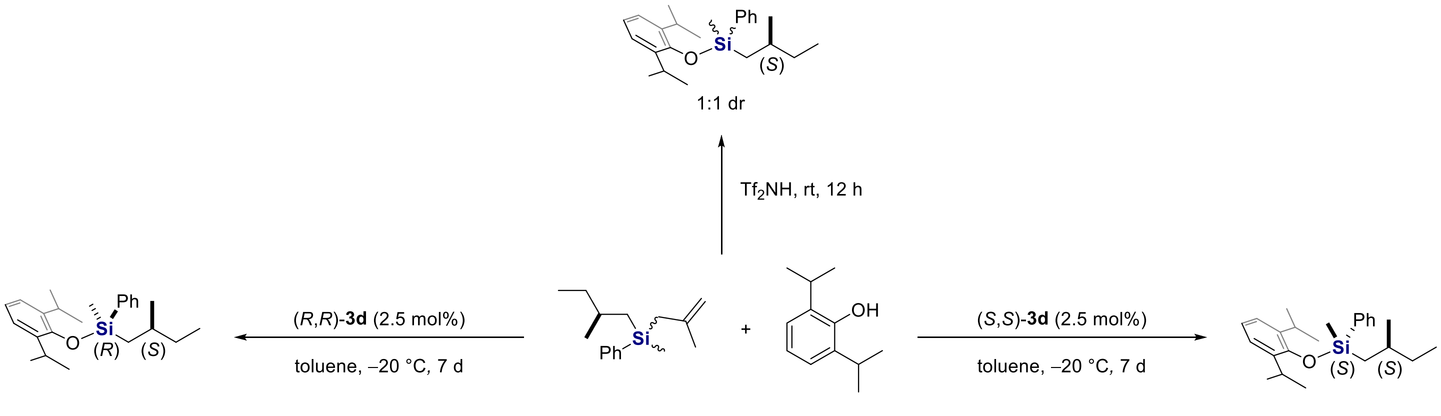

4t, 76%, 18:1 d.r. **1v** **2** **4u**, 66%, >20:1 d.r.

Procedure I: To a 1.5 mL GC vial, Tf_2NH (1.0 mol%) in toluene (0.2 M, 1 mL) was added under air. Propofol **2** (1.5 equiv., 56 μL, 0.3 mmol) and the *rac*-silane starting materials **1** (0.2 mmol) were added afterwards. The reaction mixture was stirred for an additional 12 h at the indicated temperature. After *rac*-**1** was fully consumed, as monitored by TLC, the reaction mixture was treated with triethylamine. All volatiles were removed in vacuo and the residue was purified by column chromatography with silica gel, which was pre-neutralized by triethylamine, to afford the desired silyl ether.

Procedure II: To a flame-dried Schlenk tube, (*S*, *S*)-IDPi **3d** or (*R*, *R*)-IDPi **3d** (2.5 mol%) in toluene (0.1 M, 1.0 mL) was added under Ar. The solution was lowered to –20 °C for 10 min, then propofol **2** (1.5 equiv., 28 μL, 0.15 mmol) and the *rac*-silane starting materials **1** (0.1 mmol) were added afterwards. The reaction mixture was stirred for an additional 7 d at the indicated temperature. After *rac*-**1** was fully consumed, as monitored by TLC, the reaction mixture was treated with triethylamine. All volatiles were removed in vacuo and the NMR yield was determined using CH_2Br_2 as internal standard. The residue was purified by column chromatography with silica gel, which was pre-neutralized by triethylamine, to afford the desired silyl ethers **4t** and **4u** (76% and 66% isolated yield, respectively).

(*R*)-(2,6-diisopropylphenoxy)(methyl)((*S*)-2-methylbutyl)(phenyl)silane 4t

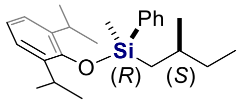

The titled product was purified by column chromatography with hexanes as eluent to afford **4t** as a colorless oil (28 mg, 76% yield). d.r. = 18:1

^1H NMR (501 MHz, CD_2Cl_2) δ 7.64–7.54 (m, 2H), 7.42–7.30 (m, 3H), 7.04–6.96 (m, 2H), 6.91 (dd, *J* = 8.3, 6.9 Hz, 1H), 3.15 (p, *J* = 6.9 Hz, 2H), 1.57 (dd, *J* = 6.9, 1.8 Hz, 1H), 1.34 (dd, *J* = 7.5, 5.6 Hz, 1H), 1.28–1.14 (m, 2H), 1.05 (dd, *J* = 15.6, 6.9 Hz, 12H), 0.96–0.91 (m, 1H), 0.88 (d, *J* = 6.6 Hz, 3H), 0.83 (t, *J* = 7.4 Hz, 3H), 0.49 (s, 3H).

^{13}C NMR (126 MHz, CD_2Cl_2) δ 150.1, 139.5, 137.8, 133.9, 129.9, 128.1, 123.7, 122.3, 33.5, 30.7, 27.4, 24.5, 23.5, 23.4, 22.5, 11.7, –1.9.

R_f = 0.43 (hexanes).

EI-HRMS (*m/z*): calculated for $C_{24}H_{36}O_1Si_1$ [$M^{+•}$]: 368.2530, found: 368.2528.

$[\alpha]_D^{25}$ = –4.4 (*c* 0.54, $CHCl_3$).

(*S*)-(2,6-diisopropylphenoxy)(methyl)((*S*)-2-methylbutyl)(phenyl)silane 4u

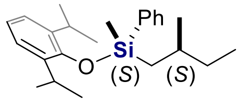

The titled product was purified by column chromatography with hexanes as eluent to afford **4u** as a colorless oil (24.5 mg, 66% yield). d.r. > 20:1

^1H NMR (501 MHz, CD_2Cl_2) δ 7.64–7.56 (m, 2H), 7.42–7.31 (m, 3H), 7.05–6.96 (m, 2H), 6.91 (dd, *J* = 8.3, 6.9 Hz, 1H), 3.16 (p, *J* = 6.9 Hz, 2H), 1.60 (td, *J* = 6.8, 1.7 Hz, 1H), 1.31 (dd, *J* = 7.5, 5.6 Hz, 1H), 1.25–1.13 (m, 2H), 1.05 (dd, *J* = 16.2, 6.8 Hz, 12H), 0.98 (dd, *J* = 15.1, 8.6 Hz, 1H), 0.89 (d, *J* = 6.6 Hz, 3H), 0.82 (t, *J* = 7.4 Hz, 3H), 0.50 (s, 3H).

^{13}C NMR (126 MHz, CD_2Cl_2) δ 150.1, 139.5, 138.0, 133.9, 129.9, 128.0, 123.7, 122.3, 33.4, 30.8, 27.4, 24.4, 23.5, 23.4, 22.6, 11.6, –2.1.

R_f = 0.43 (hexanes).

EI-HRMS (*m/z*): calculated for $C_{24}H_{36}O_1Si_1$ [$M^{+•}$]: 368.2530, found: 368.2528.

$[\alpha]_D^{25}$ = 32.4 (*c* 0.5, $CHCl_3$).

5. Gram-scale synthesis and elaboration of 4a

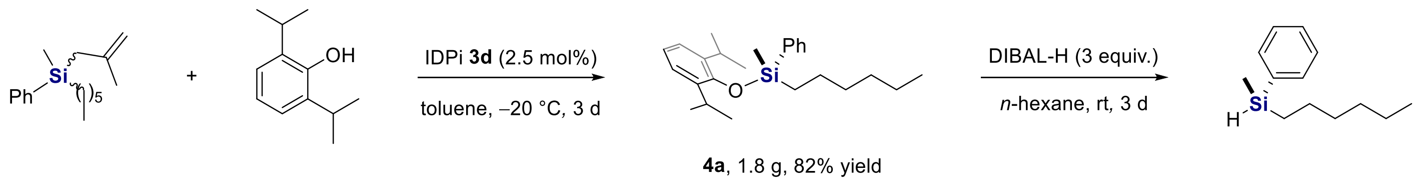

I. Gram scale catalytic reaction of silane 1a with Propofol 2.

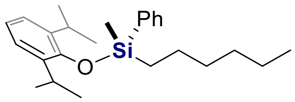

Propofol **2** (1.5 g, 8.6 mmol, 1.5 equiv.) was placed in a round-bottom flask, which was equipped with a teflon-coated magnetic stirring bar. IDPi **3d** (264 mg, 0.14 mmol, 2.5 mol%) and toluene (0.1 M, 58.0 mL) were added under argon, and the resultant solution was stirred at –20 °C for 10 min before the silane starting material **1a** (1.8 mL, 5.8 mmol, 1.0 equiv.) was slowly added. The reaction mixture was stirred for an additional 3 d at the indicated temperature and then treated with 5 drops of triethylamine via pipet. All volatiles were removed in vacuo and the crude residue was purified by column chromatography with silica gel, which was pre-neutralized by triethylamine, to afford the desired silane product **4a** (1.8 g, 82% yield, 99:1 e.r.) and catalyst **3d** (233 mg, 88% yield).

II. Preparation of hexyl(methyl)(phenyl)silane 5

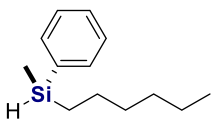

According to a known procedure with minor modification[6]

To a solution of silane **4a** (115 mg, 0.3 mmol) in *n*-hexane (2 mL), DIBAL-H (1 M in DCM, 0.9 mL, 0.9 mmol, 3 equiv.) was added dropwise at 0 °C and stirred for 3 d at room temperature. After the reaction completed (monitored by TLC), the reaction was quenched with aqueous NaCl (sat.) and extracted with diethylether (3 × 3 mL). The organic layers were combined, dried over anhydrous Na_2SO_4. The solvent was removed under reduced pressure and purified by column chromatography with pentane as eluent, affording product **5** as a colorless oil (53.2 mg, 86% yield, 99:1 e.r.).

^{1}H NMR (501 MHz, CD_2Cl_2) δ 7.60–7.48 (m, 2H), 7.46–7.31 (m, 3H), 4.34 (q, *J* = 3.6 Hz, 1H), 1.40–1.25 (m, 8H), 0.90–0.81 (m, 5H), 0.33 (d, *J* = 3.8 Hz, 3H).

^{13}C NMR (126 MHz, CD_2Cl_2) δ 137.3, 134.7, 129.5, 128.2, 33.3, 32.0, 24.7, 23.0, 14.3, 13.7, –5.5.

R_f = 0.75 (Hexanes).

CI-HRMS (*m/z*): calculated for $C_{13}H_{21}Si_1$ ([M–H]$^-$): 205.1407, found: 205.1406.

HPLC (IG-3R, MeOH/Water = 80:20, 1.0 mL/min, 298 K, 220 nm): t_{R1} = 13.0 min, t_{R2} = 13.9 min, e.r. = 99:1.

$[\alpha]_D^{25}$ = 6.0 (*c* 1.0, $CHCl_3$).

1,3-dihexyl-1,3-dimethyl-1,3-diphenyldisiloxane 6

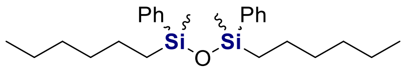

^{1}H NMR (501 MHz, CD_2Cl_2) δ 7.55 (ddq, *J* = 9.4, 5.8, 1.9 Hz, 4H), 7.44–7.28 (m, 6H), 1.43–1.18 (m, 16H), 0.99–0.70 (m, 10H), 0.33 (t, *J* = 3.4 Hz, 6H).

^{13}C NMR (126 MHz, CD_2Cl_2) δ 139.7, 133.7, 129.6, 128.0, 33.5, 32.0, 23.6, 23.0, 17.8, 17.8, 14.3, –0.8.

R_f = 0.60 (hexanes).

ESI-HRMS (*m/z*): calculated for $C_{26}H_{42}Na_1O_1Si_2$ ([M+Na]$^+$): 449.2666, found: 449.2667.

hexyl(methyl)(phenyl)silanol

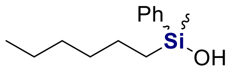

^{1}H NMR (501 MHz, CD_2Cl_2) δ 7.61–7.54 (m, 2H), 7.42–7.33 (m, 3H), 1.90 (s, 1H), 1.38–1.22 (m, 8H), 0.91–0.81 (m, 5H), 0.35 (s, 3H).

^{13}C NMR (126 MHz, CD_2Cl_2) δ 139.2, 133.6, 129.8, 128.2, 33.5, 31.9, 23.4, 23.0, 16.9, 14.3, –1.5.

R_f = 0.21 (Ethyl acetate/hexanes = 1:9).

CI-HRMS (*m/z*): calculated for $C_{13}H_{26}N_1O_1Si_1$ ([M+NH$_4$]$^+$): 240.1778, found: 240.1779.

(*S*, *S*)-IDPi-3d

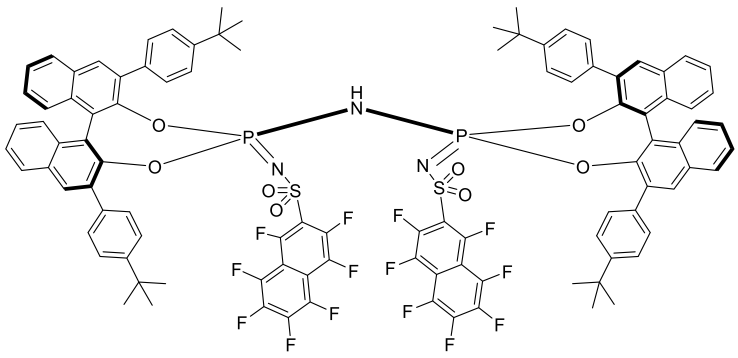

^{1}H NMR (501 MHz, CD_2Cl_2) δ 8.21 (d, *J* = 8.2 Hz, 2H), 8.00 (d, *J* = 10.0 Hz, 4H), 7.78 (d, *J* = 8.3 Hz, 2H), 7.70 (ddd, *J* = 8.1, 6.7, 1.1 Hz, 2H), 7.58 (q, *J* = 8.7 Hz, 8H), 7.44 (ddd, *J* = 8.4, 6.8, 1.3 Hz, 2H), 7.37 (ddd, *J* = 8.2, 5.9, 2.0 Hz, 2H), 7.14 (d, *J* = 8.6 Hz, 2H), 7.12–7.04 (m, 4H), 6.89–6.81 (m, 4H), 6.52–6.45 (m, 4H), 4.27 (s, 1H), 1.31 (s, 18H), 0.86 (s, 18H).

^{13}C NMR (126 MHz, CD_2Cl_2) δ 151.7, 150.5, 143.9, 143.7, 134.6, 133.2, 133.1, 132.9, 132.5, 131.9, 131.7, 131.6, 131.2, 130.5, 129.9, 129.4, 129.3, 128.3, 127.5, 127.1, 127.0, 126.6, 126.5, 126.5, 124.9, 123.3, 123.0, 35.0, 34.6, 31.3, 31.3.

^{31}P NMR (203 MHz, CD_2Cl_2) δ –6.1.

^{19}F NMR (471 MHz, CD_2Cl_2) δ –111.7 (dd, *J* = 76.7, 18.4 Hz), –133.5 (d, *J* = 18.7 Hz), –142.1 (dd, *J* = 76.6, 16.9 Hz), –144.7 (dd, *J* = 58.2, 16.9 Hz), –147.2 – –147.5 (m), –150.5 (t, *J* = 19.1 Hz), –155.2 (d, *J* = 19.6 Hz).

R_f = 0.45 (Ethyl acetate/hexanes = 1:4).

ESI-HRMS (*m/z*): calculated for $C_{100}H_{72}F_{14}N_3O_8P_2S_2$ ([M-H]$^-$): 1834.4018, found: 1834.4023.

m.p. = 220–256 °C.

$[\boldsymbol{\alpha}]_{\mathbf{D}}^{\mathbf{25}}$ = 120.9 (*c* 0.44, $CHCl_3$).

Note: the catalysts employed in this work were synthesized according to the procedures described in our recently reported work.[3,7] *IDPi* ***3b*** *was a known compound and the spectra were consistent with the reported ones.*[2]

6. Mechanistic studies

At first, comparison reactions starting from *rac*-**1a**, (*S*)-**1a**, and (*R*)-**1a** were conducted under standard reaction conditions to get a good understanding of the possible mechanisms:

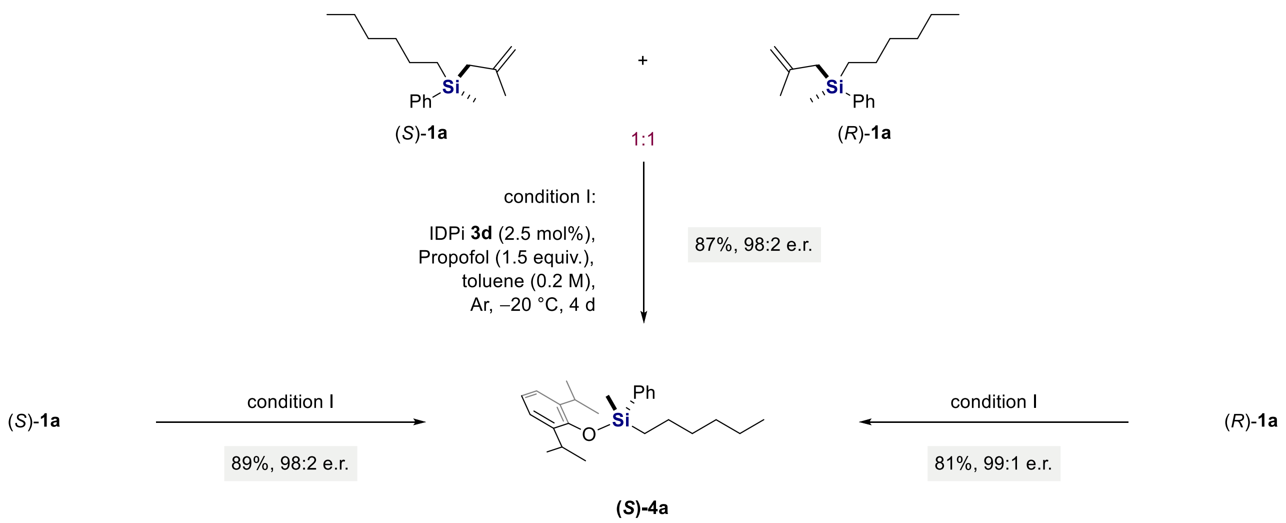

Figure S1. Comparison experiments. Reactions were performed with *rac*- or enantiopure-**1a** (0.1 mmol), **2** (0.15 mmol), and IDPi **3d** (2.5 mol%) in toluene (0.5 mL, 0.2 M) at –20 °C for 4 d.

Procedure: To a flame-dried Schlenk tube, IDPi **3d** (2.5 mol%) in toluene (0.2 M) was added under Ar. The solution was lowered to –20 °C for 10 min, then propofol **2** (1.5 equiv., 28 µL, 0.15 mmol) and the *rac*- or enantiopure silane starting materials **1a** (0.1 mmol) were added afterwards. The reaction mixture was stirred for an additional 4 d at the indicated temperature. After the silicon starting materials were fully consumed, as monitored by TLC, the reaction mixture was treated with triethylamine. All volatiles were removed in vacuo and the NMR yields were determined using CH_2Br_2 as internal standard. After purification by prep TLC, the e.r. of the desired product was determined by HPLC analysis. From the comparison experiments, we obtained *the same enantiomer* as major product with comparable results, which supports the common intermediacy.

Next, to get a better insight into the transformation, we monitored the kinetic profile of the following reaction:

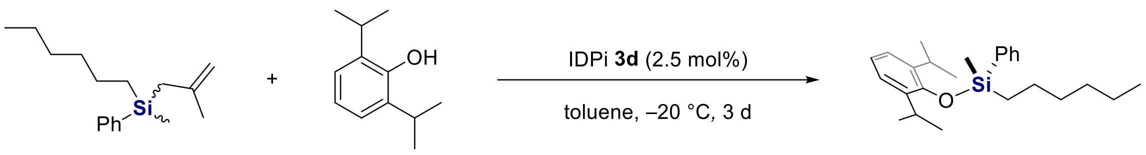

1a, 0.1 mmol **2**, 1.5 equiv. **4a**

Procedure: To a flame-dried Schlenk tube, IDPi **3d** (2.5 mol%) in toluene (0.2 M) was added under Ar. The solution was lowered to –20 °C for 10 min, then propofol **2** (1.5 equiv., 28 µL, 0.15 mmol) and the *rac*-starting materials **1a** (0.1 mmol) were added afterwards. The resultant mixture was kept at this temperature for an additional 3 d, from which samples were taken at different time points and treated with triethylamine. All volatiles were removed in vacuo and the conversions were determined with prior calibration method using TMB (1, 3, 5-trimethoxybenzene) as internal standard. After purification by prep TLC, the e.r. of the desired product **4a** and the remaining starting material was determined by HPLC analysis.

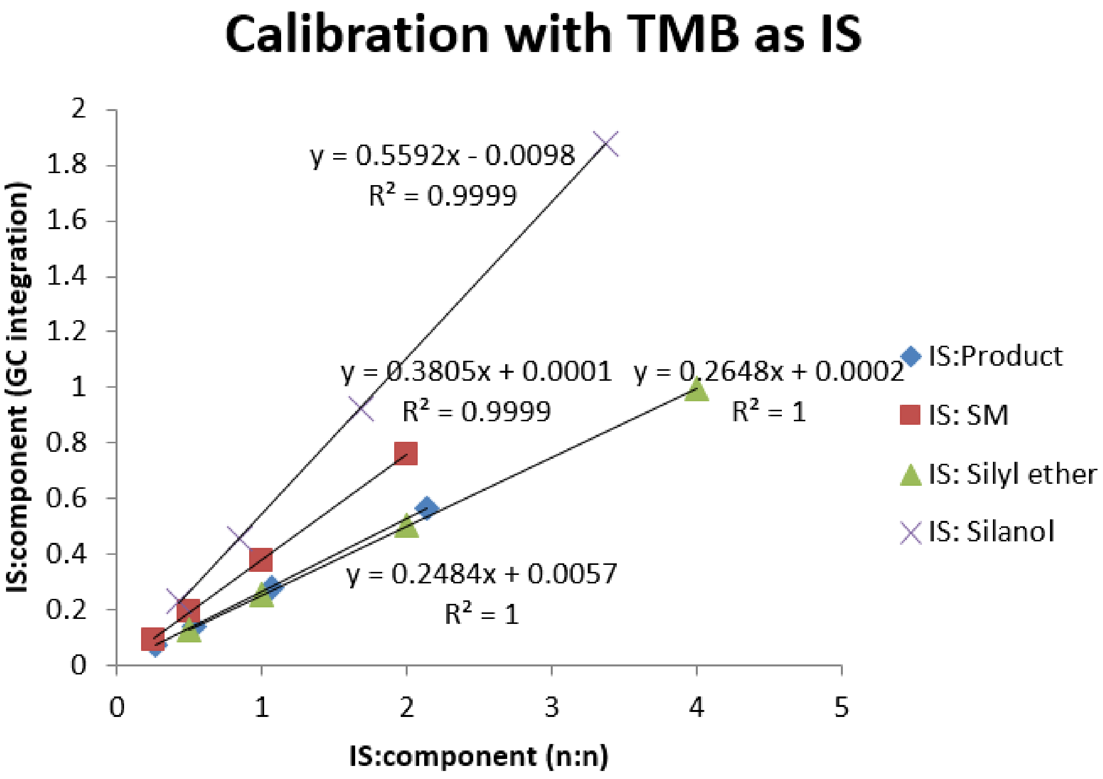

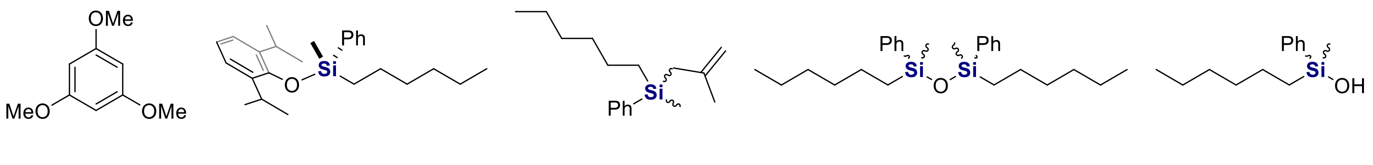

IS **Product** **SM** **Silyl ether** **Silanol**

Figure S2. GC calibrations of different components.

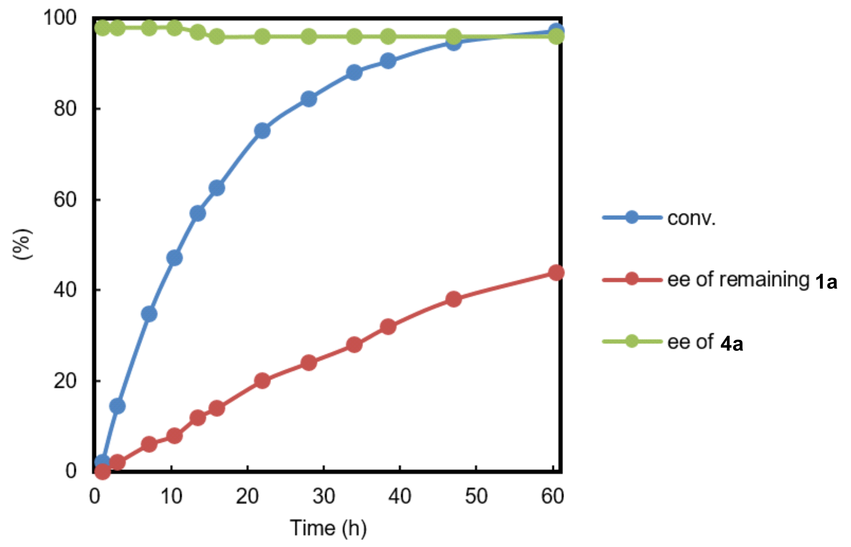

Figure S3. Control experiments. Reaction was performed with *rac*-**1a** (0.1 mmol), **2** (0.15 mmol), and IDPi **3d** (2.5 mol%) in toluene (0.5 mL, 0.2 M) at –20 °C for ~3 d.

From the plots, we confirmed that one enantiomer of **1a** reacted preferentially, thus led to the kinetic resolution. Interestingly, the IDPi catalyst allows complete control of enantioselectivity from the starting point of the reaction.

In the meantime, we conducted several control experiments to examine the racemization possibility of optically pure starting materials (*R*)-**1a**, (*S*)-**1a** and the stability of product **4a**. From the results shown in figure S4, we can see no decomposition of **4a** was detected, and no racemization of silicon starting materials was obtained, which excludes the dynamic kinetic resolution route.

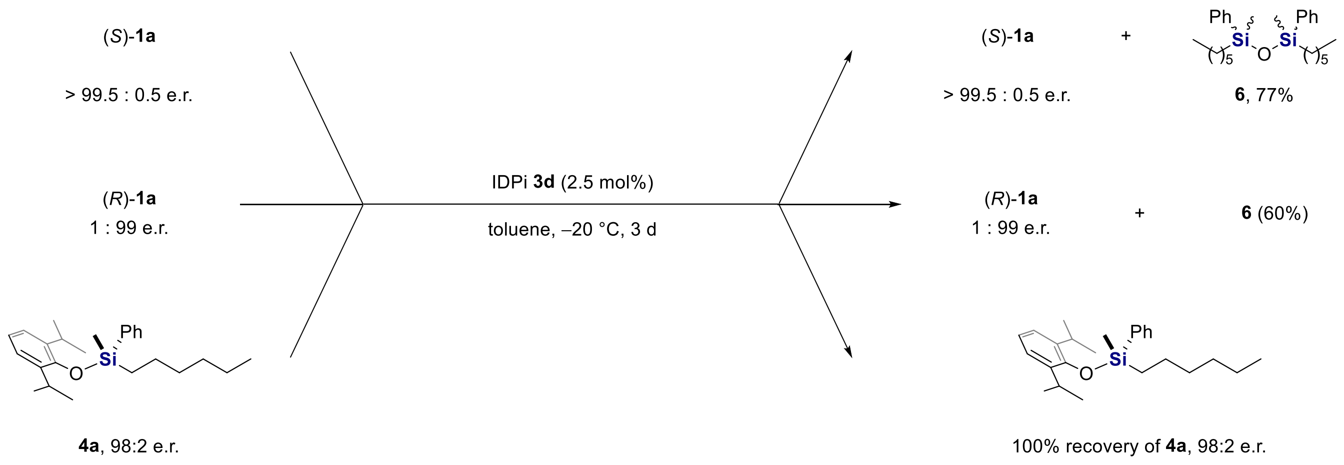

Figure S4. Control experiments. Reactions were performed with enantiopure-**1a** (0.1 mmol) or **4a**, and IDPi **3d** (2.5 mol%) in toluene (0.5 mL, 0.2 M) at –20 °C for 3 d.

Procedure: To a flame-dried Schlenk tube, enantiopure-**1a** or **4a** and IDPi **3d** (2.5 mol%) in toluene (0.2 M) was added under Ar. The solution was lowered to –20 °C and stirred at this temperature for 3 d then it was treated with triethylamine. All volatiles were removed in vacuo and the yields were determined by crude ^{1}H NMR analysis. After purification by prep TLC, the e.r. of **1a** and **4a** were determined by HPLC analysis.

In order to gain a better understanding of the reaction mechanism, the silylation of IDPi **3d** was studied by ^{31}P NMR. Upon addition of excess silane to IDPi **3d**, the sharp singlet signal corresponding to the catalyst started to broaden, and significantly shifted as a result of the consumption of water in the reaction system. Therefore, the formation of two major species, which are indicated as Cat-Si1 and Cat-Si2, was subsequently observed; nonetheless, other species (minor Cat-Si1 and minor Cat-Si2) (Figure S5) were detected as a result of the silylation process, and we observed their exchange with the two major ones through 2D NMR spectroscopy. Indeed, ^{31}P-^{31}P COSY and ^{31}P-^{31}P EXSY experiments display an existing exchange between Cat-Si1 (major and minor) and Cat-Si2 (major and minor) species.

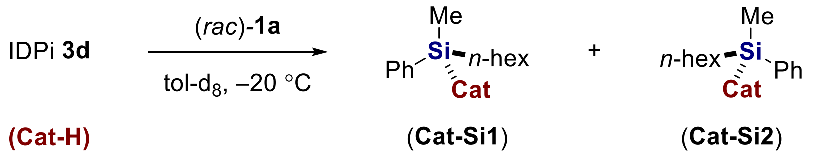

More specifically, Figure S5 shows that the species reached a steady state, which we perturbed by adding propofol (**2**). This resulted in the fast consumption of one of the two species (Cat-Si2), leading us to hypothesize that Cat-Si1 and Cat-Si2 are two covalently-bound species resulting from the reaction between the (*S*,*S*)-IDPi catalyst and each enantiomer of the starting material. Moreover, we speculated that while Cat-Si2 reacts rapidly, Cat-Si1 slowly converts into the reactive species through an epimerization process.

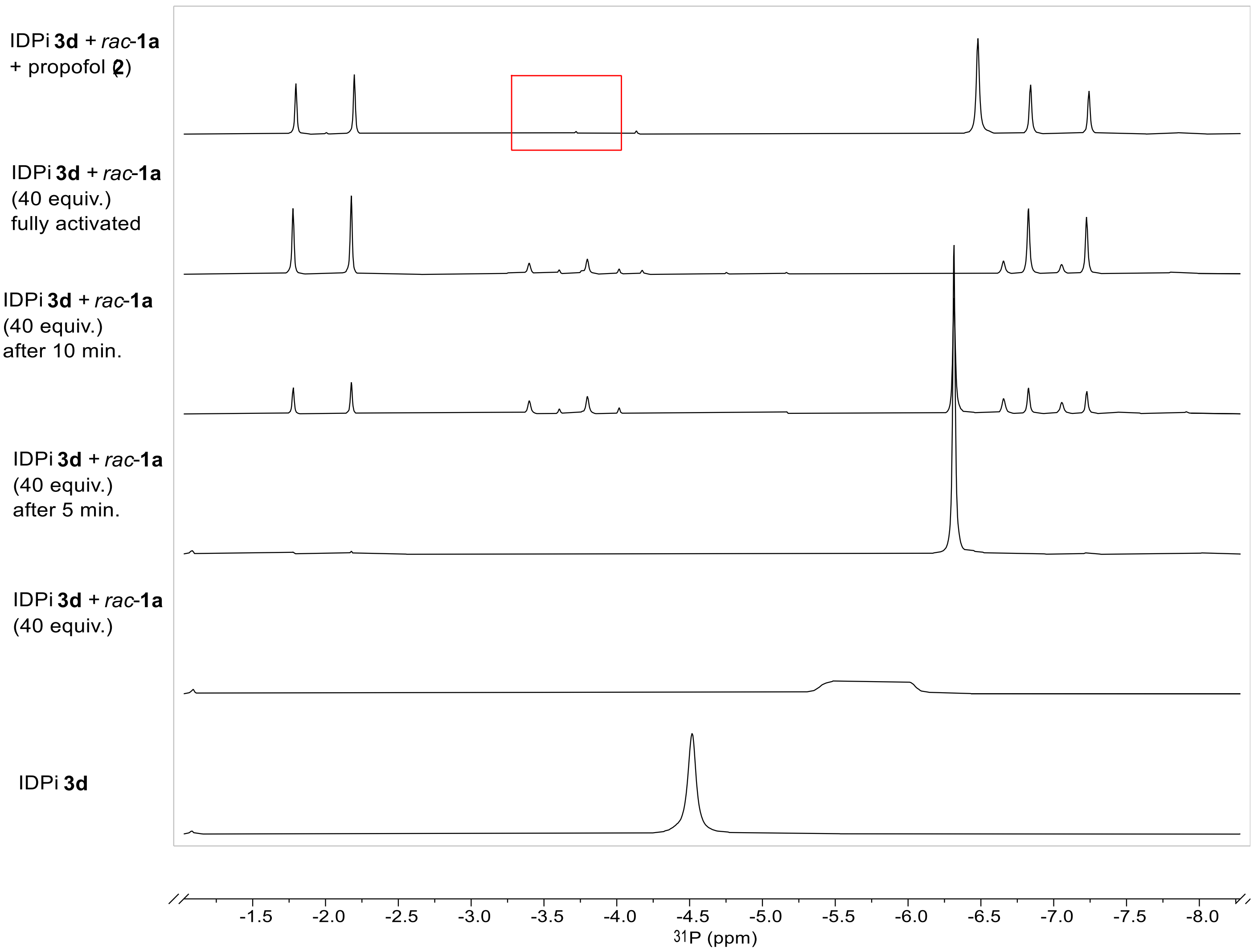

Figure S5. Activation of catalyst 3d. NMR studies were performed with *rac*-**1a** (0.1 mmol, 1.0 equiv.), and IDPi **3d** (2.5 mol%) in toluene-d_8 (0.1 M) at –20 °C under Ar, and ^{31}P NMR data were given.

Procedure: To an oven-dried Young NMR tube, *rac*-**1a** (40 equiv.) and IDPi **3d** (2.5 mol%) in toluene-d_8 (0.1 M) was added under Ar. The solution was cooled to –20 °C and kept at this temperature in the NMR spectrometer (600 MHz) while acquiring ^{31}P NMR data at different time points.

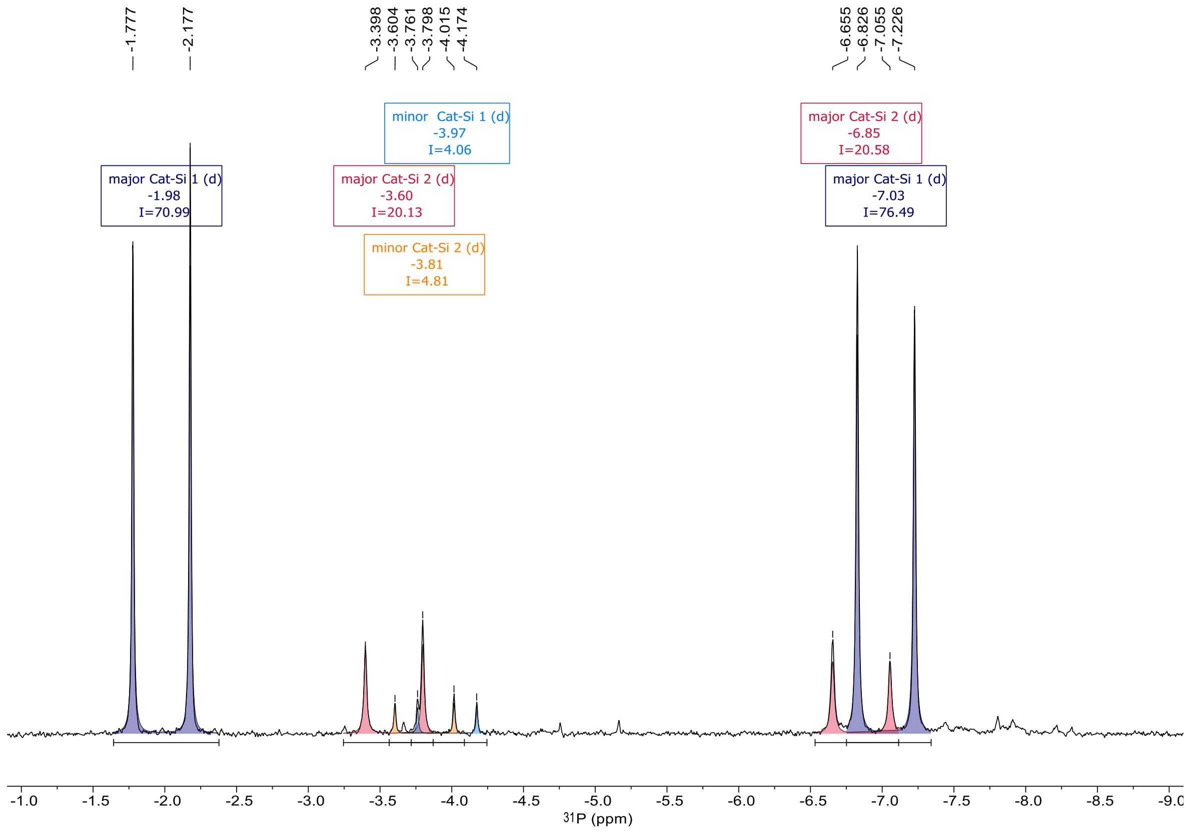

Figure S6. Species resulting from the silylation of catalyst 3d. NMR studies were performed with *rac*-**1a** (0.1 mmol, 1.0 equiv.), and IDPi **3d** (2.5 mol%) in toluene-d_8 (0.1 M) at –20 °C under Ar, and ^{31}P NMR data were given.

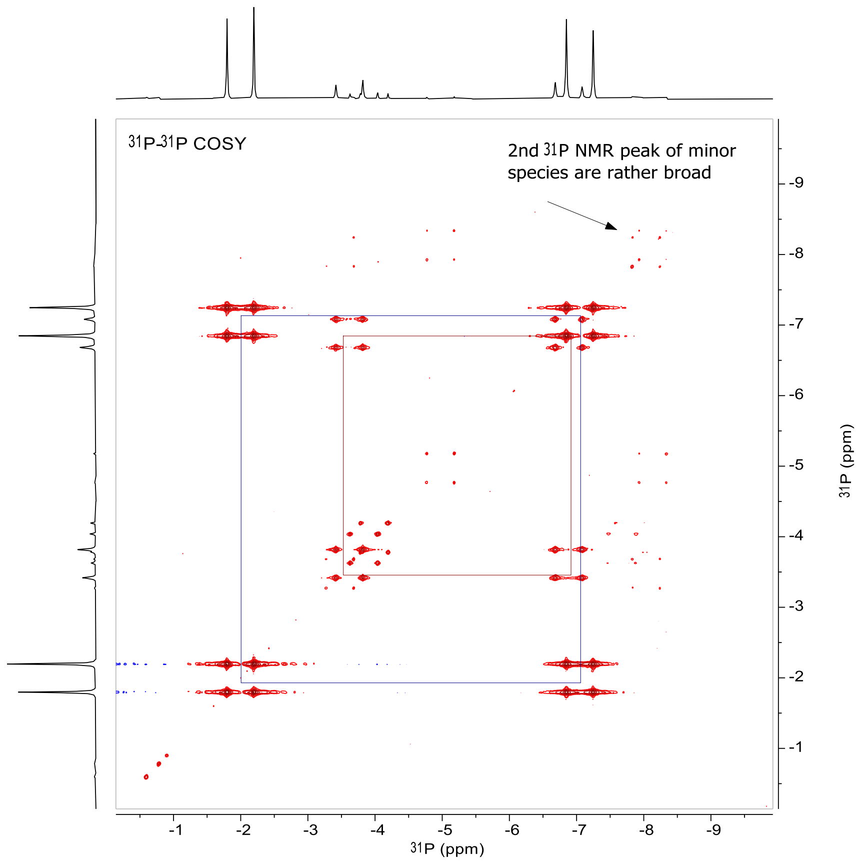
31P-31P COSY
2nd 31P NMR peak of minor species are rather broad
31P (ppm)
31P (ppm)

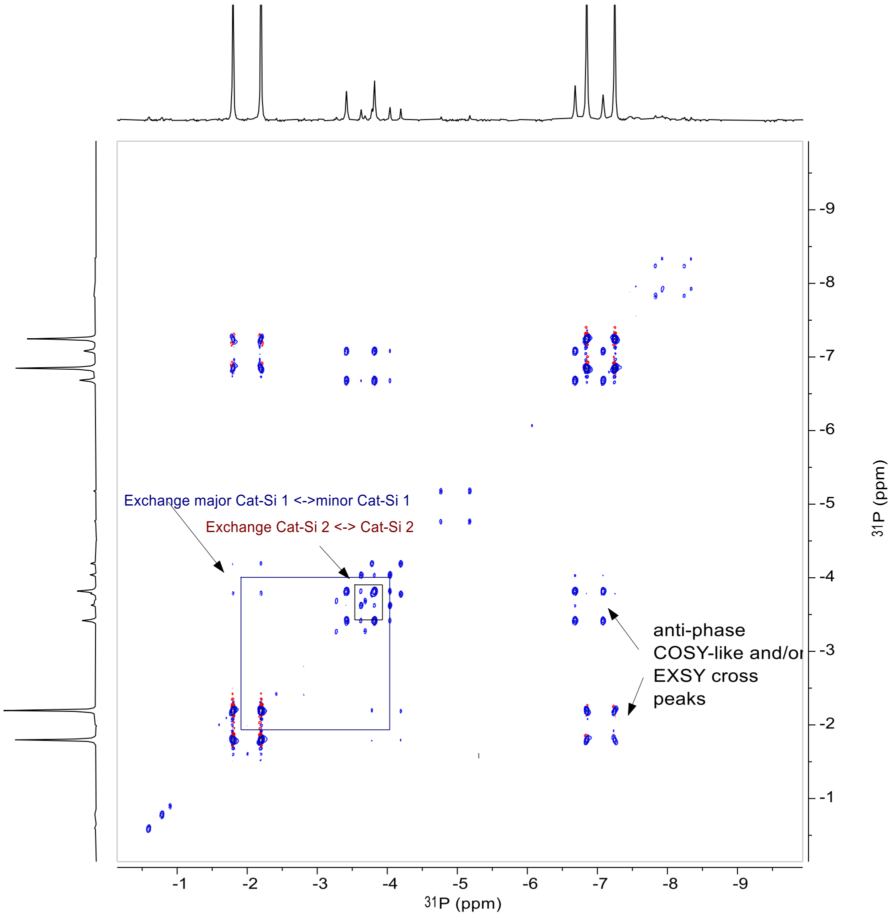
Exchange major Cat-Si 1 <->minor Cat-Si 1
Exchange Cat-Si 2 <-> Cat-Si 2
anti-phase COSY-like and/or EXSY cross peaks
31P (ppm)
31P (ppm)

Figure S7. 2D NMR experiments. ^{31}P-^{31}P COSY and ^{31}P-^{31}P EXSY displaying exchange between Cat-Si1 and Cat-Si2 species.

Further indication pointing at the existence of two diasteromeric species came from the parallel experiment that we run with a non-chiral silicon substrate (Figure S8). Indeed, in this case only one species was obtained, confirming our assumption for the c ovalent interaction between IDPi **3d** and *rac*-**1a**.

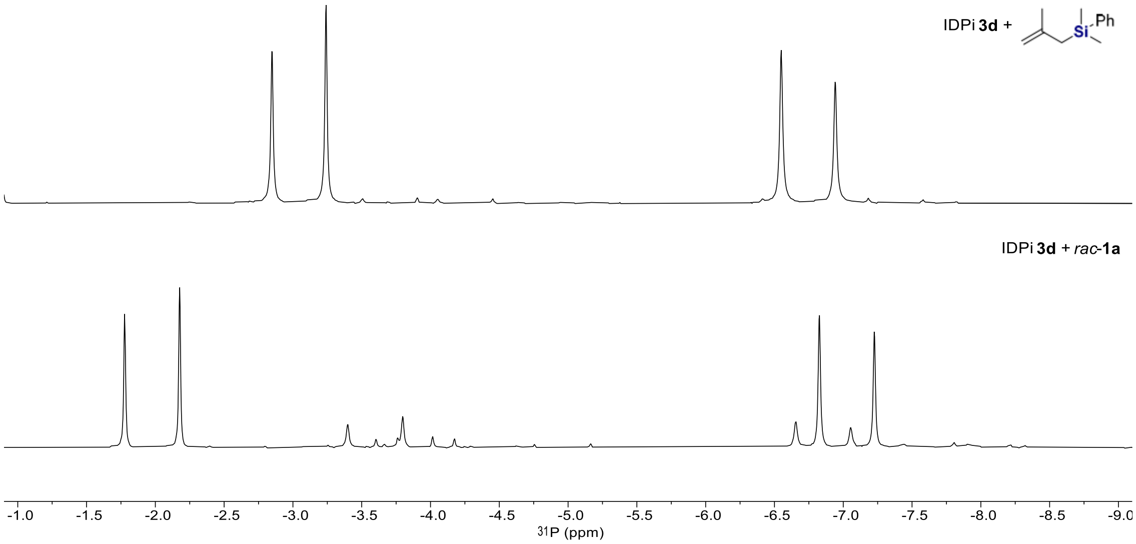

Figure S8. ^{31}P NMR comparison. NMR studies were performed with an achiral substrate (top) (0.1 mmol, 1.0 equiv.) or *rac*-**1a** (bottom) (0.1 mmol, 1.0 equiv.), and IDPi **3d** (2.5 mol%) in toluene-d_8 (0.1 M) at –20 °C under Ar, and ^{31}P NMR data were given.

To gain a deeper understanding on the nature of the silylated species, we run three experiments under the same conditions utilizing IDPi **3d** in the presence of the racemic silane starting material (*rac*-**1a**) as well as the single enantiomers, namely *R*-**1a** and *S*-**1a**. In figure S9, we report a comparison between ^{31}P NMR measured for the three starting materials at the beginning of the silylation process. While the interaction between *rac*-**1a** and IDPi **3a** resulted in the initial formation of Cat-Si1 and Cat-Si2 in a 1:1 ratio, the enantiomers showed the two species forming in different ratios.

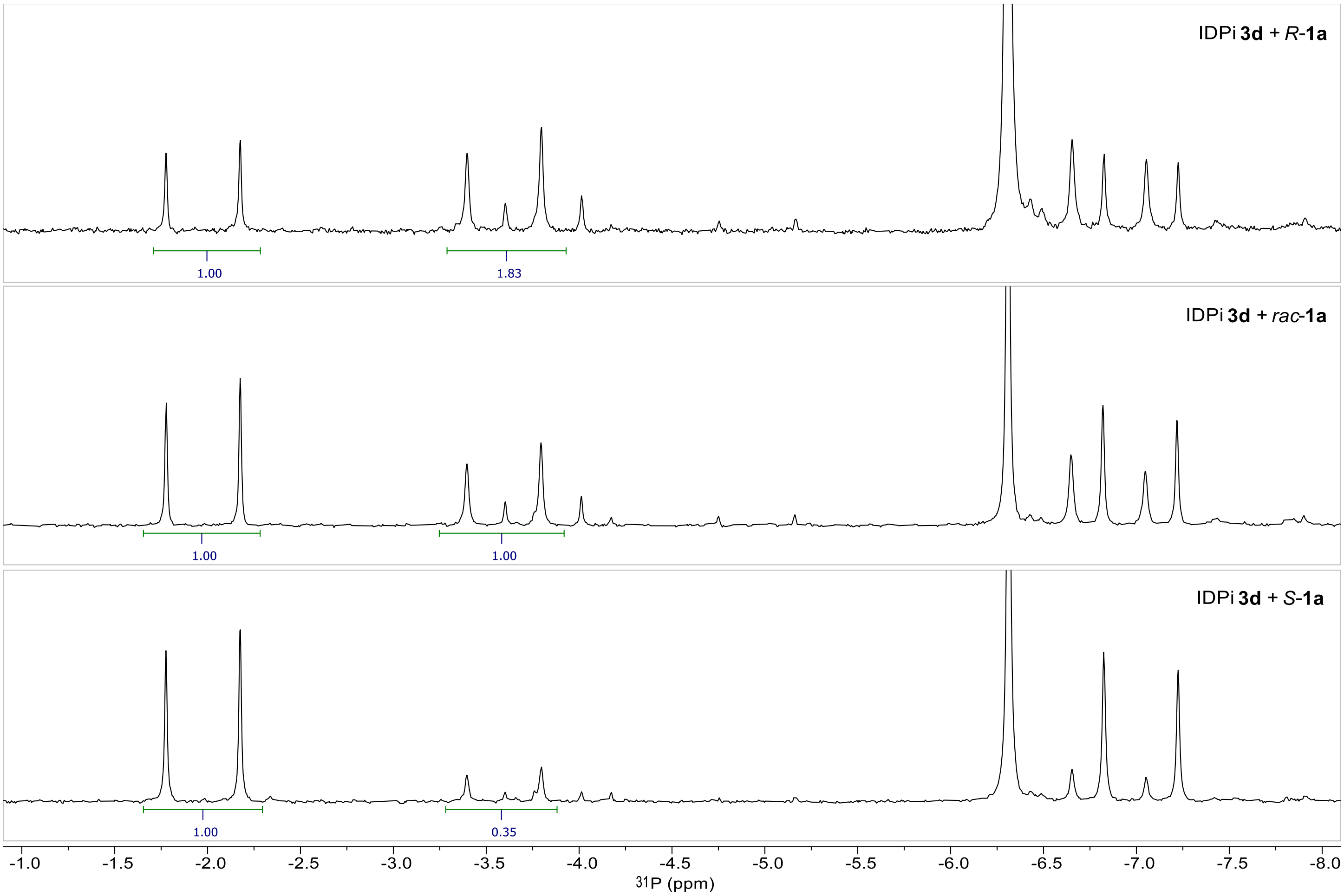

Figure S9. Comparison experiments of silylation with enantiopure and racemic silanes. NMR studies were performed with **1a** (0.1 mmol, 1.0 equiv.), and IDPi **3d** (2.5 mol%) in toluene-d_8 (0.1 M) at –20 °C under Ar, and ^{31}P NMR data were given.

However, a more exhaustive kinetic analysis realized by ^{31}P NMR reaction monitoring, showed that the systems reached an equilibrium after approximately one hour. The curves in Figure S10 show the activation for *rac*-**1a**, as well as for the single enantiomers *S*-**1a** and *R*-**1a** and suggest that the equilibrium is reached after a comparable time regardless of the chiral information carried by the starting material.

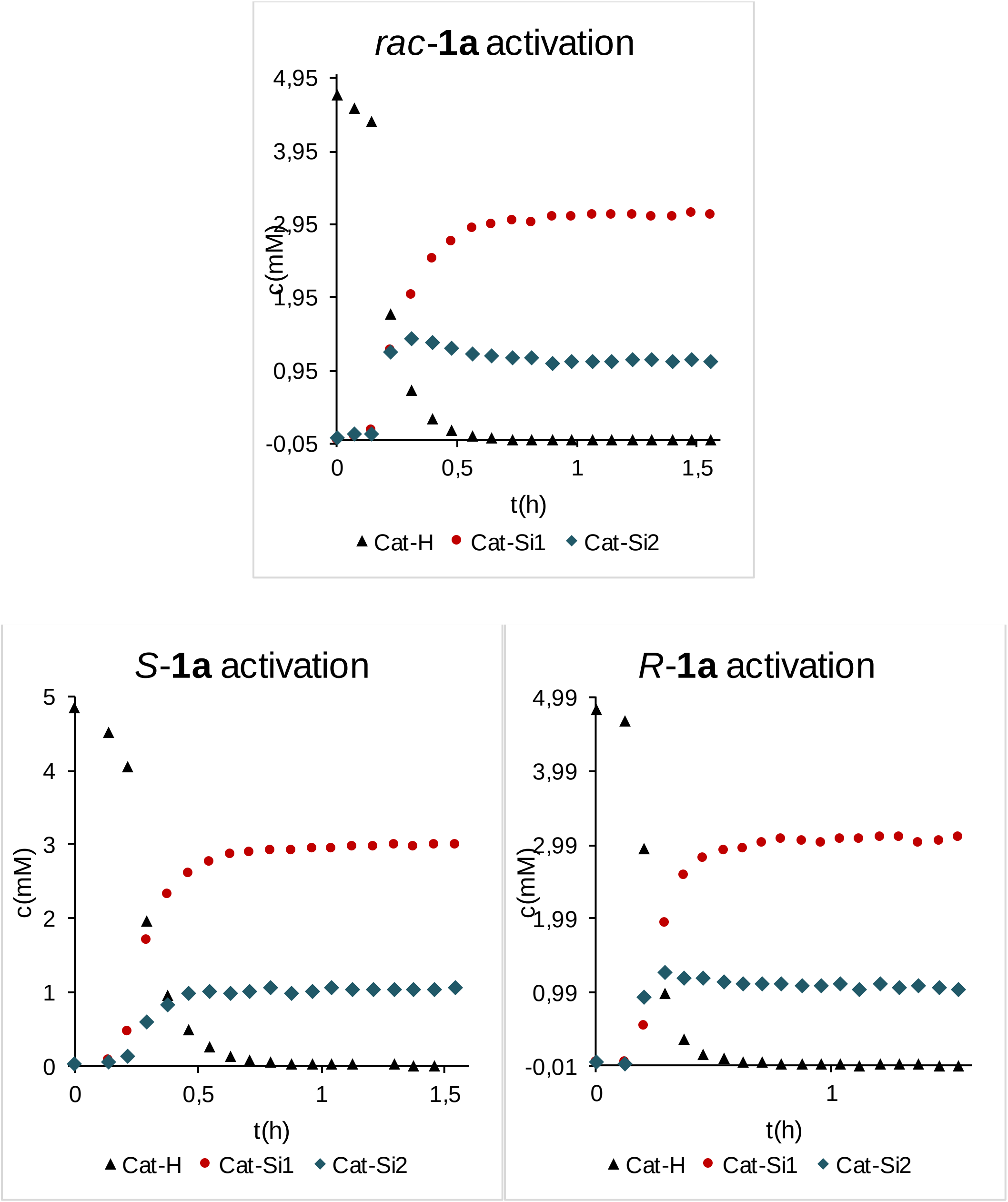

Figure S10. Activation process. NMR studies were performed with **1a** (0.1 mmol, 1.0 equiv.), and IDPi **3d** (2.5 mol%) in toluene-d_8 (0.1 M) at –20 °C under Ar; the values were obtained from ^{31}P NMR reaction monitoring.

Lastly, we monitored the overall reaction of the racemic (*rac*-**1a**) and enantiopure silanes (*R*- and *S*-**1a**) with propofol (**2**) catalyzed by IDPi **3d** via NMR spectroscopy. We analyzed initial rates as well as the overall reaction by ^{31}P NMR, and we also evaluated the reactions by ^{1}H NMR spectroscopy. This analysis revealed that *R*-**1a** reacts faster than *S*-**1a**, and that the values for the racemate can be found in between those of the two enantiopure compounds as illustrated in Figure S11. Moreover, these results are consistent with the time-course study (Figure S4) that disclosed how kinetic resolution occurs in the reaction system.

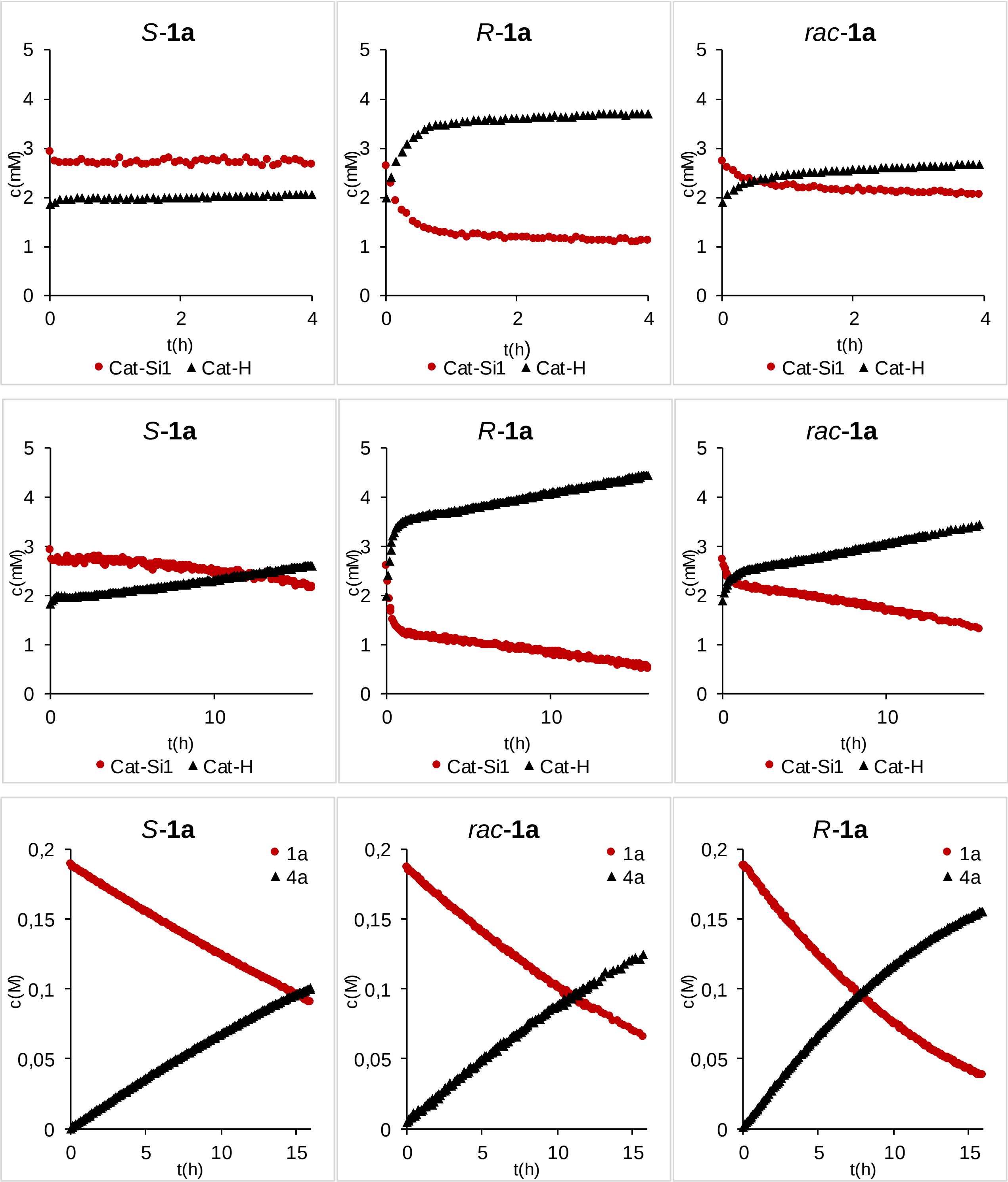

Figure S11. Reaction monitoring by NMR spectroscopy. NMR studies were performed with *S*-**1a**, *rac*-**1a** and *R*-**1a** (0.1 mmol, 1.0 equiv.), propofol (0.15 mmol, 1.5 equiv.) and IDPi **3d** (2.5 mol%) in toluene-d_8 (0.1 M) at –20 °C under Ar. From the top: initial rates obtained from ^{31}P NMR reaction monitoring; overall profile obtained from ^{31}P NMR reaction monitoring; overall profile obtained from ^{1}H NMR reaction monitoring.

In order to understand the substituents effect with the aim of have more insights into the reaction mechanism and its intermediates, we also performed the Hammett plot analysis that allowed us to postulate the existence of a positively charged reaction intermediate (Figure S12).

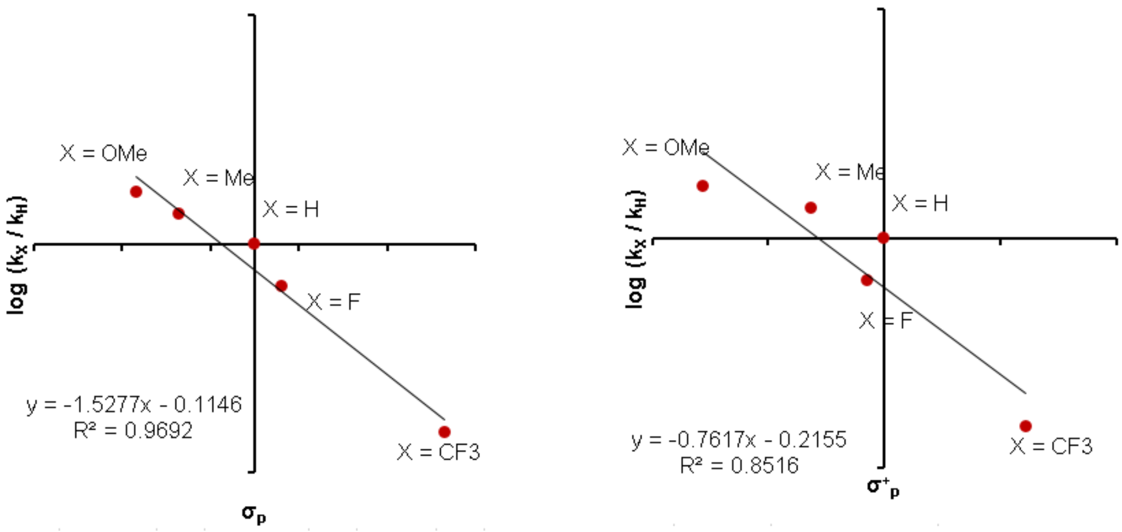

Figure S12. Hammett plot analysis. NMR studies were performed with *rac*-silanes (0.1 mmol, 1.0 equiv.), propofol (0.15 mmol, 1.5 equiv.) and IDPi **3d** (2.5 mol%) in toluene-d_8 (0.1 M) at –20 °C under Ar; the values were obtained from ^{1}H NMR reaction monitoring.

7. Computational studies

Computational details

A preliminary exploration of the conformational space for reaction intermediates (Cat-Si1 and Cat-Si2) and transition states was carried out at the GFN-xtb level of theory,[8] as implemented in ORCA version 5.0.3.[9] The conformers found were optimized using the Amsterdam Density Functional (ADF) code[10,11] in combination with the related Quantum-regions Interconnected by Local Description (QUILD) program.[12] The PBE[13] GGA exchange-correlation (XC) functional, the DZP basis set with a large frozen core approximation for all atoms, and the Grimme's D3-BJ dispersion correction were used.[14,15] Frequency calculations were carried out at the same level of theory to verify the nature of the stationary points along the section of potential energy surface investigated in this work. Thermal corrections were included at 298K. Final single point energies were refined at the PBE-D3/def2-TZVP +CPCM(Toluene) level of theory. This combination of functional and basis set has been successfully used in a number of different organocatalyzed transformations.[16]

Calculated coordinates

Cat-Si1

C	-3.02293000000000	-6.00977900000000	-1.11258600000000
C	-1.67768300000000	-5.60654500000000	-1.12868700000000
C	-0.69972200000000	-6.56755300000000	-1.42860800000000
C	-1.04787800000000	-7.89211000000000	-1.68706800000000
C	-2.38731000000000	-8.27713600000000	-1.65466500000000

C	-3.37576800000000	-7.33272200000000	-1.37464600000000
Si	-1.17060100000000	-3.87934600000000	-0.58823300000000
C	-1.51932200000000	-3.59622800000000	1.23657400000000
C	-1.64967800000000	-4.92947700000000	1.98725400000000
C	-0.38033800000000	-5.78810300000000	1.92231800000000
C	-0.66315000000000	-7.28187900000000	2.03200500000000
C	0.58518900000000	-8.14352000000000	1.86294100000000
C	0.27724200000000	-9.63877300000000	1.87503200000000
O	1.63846000000000	-3.05377600000000	2.20246200000000
S	2.08127900000000	-1.66922600000000	2.15250300000000
O	1.91502200000000	-0.83760900000000	3.33668400000000
C	3.87275700000000	-1.74345100000000	1.77525400000000
C	4.35531100000000	-2.75447500000000	0.91259700000000
C	5.68592500000000	-2.85249200000000	0.58704300000000
C	6.63687700000000	-1.93447700000000	1.08034600000000
C	6.17441600000000	-0.89110200000000	1.96154700000000
C	4.79137500000000	-0.84147700000000	2.28064900000000
C	7.12480900000000	0.02800200000000	2.47324700000000
C	8.45501600000000	-0.06620700000000	2.13673600000000
C	8.89605500000000	-1.07018200000000	1.26043500000000
C	8.00763000000000	-1.98047100000000	0.74088300000000
F	8.47656900000000	-2.88396500000000	-0.14878000000000
F	6.76307800000000	1.03303600000000	3.29181100000000
F	9.34770300000000	0.82777100000000	2.60120400000000
F	10.18871100000000	-1.08427200000000	0.88619400000000
F	6.07098100000000	-3.85239000000000	-0.24188500000000
F	3.51666700000000	-3.65246600000000	0.37050000000000
F	4.38640400000000	0.13773900000000	3.10574900000000
N	1.49373800000000	-1.01144600000000	0.81431300000000
P	1.50775000000000	0.50125100000000	0.35684300000000
N	0.12492400000000	1.16302400000000	-0.03627100000000
P	-1.34186300000000	0.68444500000000	-0.17602700000000
N	-1.69854300000000	-0.87811600000000	-0.44908000000000

S	-2.35202700000000	-1.41345500000000	-1.74827000000000
O	-2.39551200000000	-2.94972400000000	-1.56174100000000
O	2.31487800000000	1.40871000000000	1.44008100000000
C	2.60279200000000	2.71367000000000	1.03644500000000
C	3.67341000000000	2.91278500000000	0.18256400000000
C	3.93827500000000	4.24469000000000	-0.27533700000000
C	3.11691900000000	5.31388100000000	0.21382200000000
C	2.05502000000000	5.03562100000000	1.10345600000000
C	1.75616900000000	3.74809600000000	1.50809400000000
C	3.35160900000000	6.63464400000000	-0.24532500000000
C	4.33247600000000	6.89888000000000	-1.17288100000000
C	5.11998300000000	5.84351600000000	-1.67998300000000
C	4.92921800000000	4.55171300000000	-1.24202100000000
C	4.42862700000000	1.75277900000000	-0.34557200000000
C	3.74301900000000	0.72695000000000	-0.98051100000000
C	4.37858200000000	-0.31381600000000	-1.71137700000000
C	5.76277400000000	-0.29139700000000	-1.75508500000000
C	6.52251900000000	0.68917800000000	-1.07872500000000
C	5.85565200000000	1.70207400000000	-0.31594100000000
C	6.63552100000000	2.63265800000000	0.41324100000000
C	8.00940400000000	2.62669400000000	0.31634700000000
C	8.66597300000000	1.68179500000000	-0.50241500000000
C	7.93774100000000	0.72134300000000	-1.16770700000000
C	0.59130700000000	3.47966800000000	2.37193100000000
C	0.66588400000000	2.63602100000000	3.48672800000000
C	-0.42451700000000	2.49819000000000	4.34385200000000
C	-1.62894300000000	3.17449900000000	4.11610000000000
C	-1.70872400000000	3.97499400000000	2.96733200000000
C	-0.62641700000000	4.12460700000000	2.11092900000000
O	2.35750400000000	0.77238100000000	-1.01032500000000
C	3.61122400000000	-1.29527700000000	-2.50764900000000
C	2.45603800000000	-0.93346100000000	-3.21716000000000
C	1.82042500000000	-1.83765900000000	-4.06282100000000

C	2.29253300000000	-3.14346500000000	-4.23030700000000
C	3.43651300000000	-3.50599200000000	-3.50403900000000
C	4.08316600000000	-2.60743500000000	-2.66499700000000
C	1.62599300000000	-4.15180300000000	-5.16828600000000
C	0.34425100000000	-3.59120600000000	-5.79838200000000
C	-2.80590200000000	3.12673700000000	5.09309700000000
C	-2.55795400000000	2.15773700000000	6.25685000000000
C	-4.09320900000000	2.68699800000000	4.37281900000000
C	-2.99694700000000	4.54907100000000	5.66016900000000
C	2.62207100000000	-4.49377200000000	-6.29426900000000
C	1.25855200000000	-5.43554500000000	-4.39872500000000
O	-2.13722400000000	1.48490700000000	-1.34725400000000
C	-2.36995500000000	2.83771900000000	-1.08391000000000
C	-3.41124200000000	3.14883700000000	-0.23103000000000
C	-3.62595300000000	4.52271000000000	0.10193400000000
C	-2.76813200000000	5.50928400000000	-0.48965900000000
C	-1.72699600000000	5.11201200000000	-1.36018500000000
C	-1.48705000000000	3.78302800000000	-1.65843800000000
C	-2.94706400000000	6.87248200000000	-0.14429900000000
C	-3.90834900000000	7.25290500000000	0.76408900000000
C	-4.73326500000000	6.27905700000000	1.36619700000000
C	-4.59771600000000	4.94723400000000	1.04182300000000
C	-4.17268600000000	2.06274400000000	0.43191000000000
C	-3.48968800000000	1.11910200000000	1.18397600000000
C	-4.11870800000000	0.14979000000000	2.01349800000000
C	-5.50577600000000	0.16507700000000	2.02336200000000
C	-6.26245400000000	1.03989700000000	1.21536400000000
C	-5.59477800000000	1.98978300000000	0.37635600000000
C	-6.36704000000000	2.82089800000000	-0.47142100000000
C	-7.74311600000000	2.76306100000000	-0.44324700000000
C	-8.40610100000000	1.86477600000000	0.42298200000000
C	-7.68088500000000	1.01295200000000	1.22424700000000
C	-0.33184000000000	3.37379500000000	-2.48012100000000

C	-0.43732200000000	2.43857500000000	-3.51608500000000
C	0.66377300000000	2.14028500000000	-4.31871400000000
C	1.90660200000000	2.75235200000000	-4.11460100000000
C	2.01215300000000	3.64648800000000	-3.03957200000000
C	0.92188500000000	3.95170200000000	-2.23748800000000
O	-2.09762200000000	1.17204700000000	1.17236900000000
C	-3.36437700000000	-0.75865700000000	2.90088000000000
C	-2.10278200000000	-0.42714700000000	3.42615900000000
C	-1.45582300000000	-1.26472900000000	4.32139100000000
C	-2.03528800000000	-2.45842700000000	4.76817300000000
C	-3.28992500000000	-2.79223400000000	4.24275400000000
C	-3.93295100000000	-1.97190100000000	3.31927600000000
C	-1.30186300000000	-3.30818900000000	5.80469800000000
C	0.10505300000000	-3.66024200000000	5.28673200000000
C	3.10367600000000	2.55211700000000	-5.04796000000000
C	2.82158300000000	1.51963600000000	-6.14681800000000
C	4.34190900000000	2.08937400000000	-4.25908000000000
C	3.40368600000000	3.91250700000000	-5.71240700000000
C	-1.17999500000000	-2.48066800000000	7.10133600000000
C	-2.04986700000000	-4.60807400000000	6.12440500000000
O	-1.81806100000000	-1.02878000000000	-3.03715600000000
C	-4.11678300000000	-1.02366500000000	-1.72447300000000
C	-4.99033700000000	-1.69180900000000	-0.83369900000000
C	-6.34579600000000	-1.47616000000000	-0.86183900000000
C	-6.92572000000000	-0.54423200000000	-1.75151600000000
C	-6.04698500000000	0.21408400000000	-2.60701400000000
C	-4.65726300000000	-0.06800100000000	-2.57095300000000
C	-6.61411200000000	1.18815900000000	-3.46571500000000
C	-7.97399300000000	1.39038500000000	-3.50609400000000
C	-8.82856300000000	0.63237900000000	-2.68958100000000
C	-8.31705000000000	-0.31579600000000	-1.83373000000000
F	-4.51522200000000	-2.56945200000000	0.06544800000000
F	-7.11220100000000	-2.16703400000000	0.00727300000000

F	-3.86712300000000	0.61292200000000	-3.41089900000000
F	-9.17937400000000	-0.99339700000000	-1.05220900000000
F	-10.14782600000000	0.88680500000000	-2.71317400000000
F	-8.50057200000000	2.34667800000000	-4.29127400000000
F	-5.84456300000000	1.97005300000000	-4.24510000000000
C	0.51229700000000	-3.42019100000000	-1.21100300000000
H	3.71105900000000	1.42218800000000	-6.78979200000000
H	2.96302100000000	4.13130500000000	-2.82422000000000
H	1.04642800000000	4.63468600000000	-1.39675600000000
H	-0.72869900000000	4.73930600000000	1.21547700000000
H	-2.63140100000000	4.50463500000000	2.73154900000000
H	-3.43749000000000	2.15427000000000	6.92033300000000
H	-1.68615800000000	2.45944200000000	6.85871200000000
H	-2.40441900000000	1.12790900000000	5.89781700000000
H	-4.95602700000000	2.78982900000000	5.05170400000000
H	-4.02537500000000	1.63485300000000	4.06861100000000
H	-4.29131900000000	3.29189800000000	3.47658700000000
H	-3.81044700000000	4.55497000000000	6.40431400000000
H	-3.25247300000000	5.26362700000000	4.86237900000000
H	-2.07158000000000	4.89834700000000	6.14478000000000
H	-0.31744900000000	1.84635800000000	5.21037200000000
H	1.58600800000000	2.09172400000000	3.70049900000000
H	1.44582000000000	5.86205600000000	1.47521600000000
H	2.72470700000000	7.43999200000000	0.14461000000000
H	4.49755800000000	7.91914400000000	-1.52377900000000
H	5.88474800000000	6.04906700000000	-2.43141100000000
H	5.54456900000000	3.75462300000000	-1.65369300000000
H	6.13205000000000	3.36205200000000	1.04877400000000
H	8.59633400000000	3.34870800000000	0.88711400000000
H	9.75548900000000	1.68945000000000	-0.57378900000000
H	8.44257800000000	-0.04201400000000	-1.76528600000000
H	6.28735700000000	-1.02594000000000	-2.37004300000000
H	4.96166600000000	-2.93912000000000	-2.11218700000000

H	3.83803700000000	-4.51746100000000	-3.58776100000000
H	-0.39576900000000	-3.30968600000000	-5.03207000000000
H	-0.11287100000000	-4.35971600000000	-6.44143600000000
H	0.55235100000000	-2.70953500000000	-6.42525600000000
H	3.53661500000000	-4.95723100000000	-5.89327600000000
H	2.91477500000000	-3.58208000000000	-6.83847200000000
H	2.16310400000000	-5.19813000000000	-7.00693000000000
H	2.13008900000000	-5.87427900000000	-3.88986500000000
H	0.85741200000000	-6.18902100000000	-5.09631200000000
H	0.48513500000000	-5.23464100000000	-3.64470600000000
H	0.92307200000000	-1.50393200000000	-4.58281000000000
H	2.04900400000000	0.07110700000000	-3.12977900000000
H	-4.88577800000000	-2.29955700000000	2.89910300000000
H	-3.77728500000000	-3.72225800000000	4.53509800000000
H	0.04719800000000	-4.23676500000000	4.35422400000000
H	0.63798400000000	-4.26470600000000	6.03899700000000
H	0.70784500000000	-2.76527100000000	5.07610400000000
H	-2.17614900000000	-2.19332000000000	7.47410700000000
H	-0.59885500000000	-1.56129800000000	6.93360200000000
H	-0.66886900000000	-3.07111700000000	7.87923300000000
H	-1.48232200000000	-5.17927400000000	6.87609500000000
H	-2.15556500000000	-5.24526500000000	5.23188300000000
H	-3.05182300000000	-4.41161700000000	6.53873000000000
H	-0.46674400000000	-0.96794400000000	4.67216400000000
H	-1.62045300000000	0.50706900000000	3.16076200000000
H	-6.04091900000000	-0.50240200000000	2.70027100000000
H	-8.18755200000000	0.29066900000000	1.86800900000000
H	-9.49759000000000	1.82771800000000	0.43206600000000
H	-8.32666500000000	3.41029400000000	-1.10203600000000
H	-5.85670500000000	3.51229200000000	-1.14420400000000
H	-5.23828000000000	4.20919000000000	1.52186900000000
H	-5.48257000000000	6.58160000000000	2.10040200000000
H	-4.02886300000000	8.30540500000000	1.02702200000000

H	-2.29421900000000	7.61671200000000	-0.60593200000000
H	-1.08336700000000	5.87667100000000	-1.80001300000000
H	-1.39116700000000	1.94979600000000	-3.71511000000000
H	0.53184700000000	1.42252600000000	-5.12815500000000
H	4.20422500000000	1.07088200000000	-3.87558700000000
H	5.22598900000000	2.08613300000000	-4.91775100000000
H	4.55537100000000	2.74916400000000	-3.40736800000000
H	2.51816300000000	4.28207900000000	-6.25309200000000
H	4.23678300000000	3.80822700000000	-6.42668200000000
H	3.68424900000000	4.66809900000000	-4.96246000000000
H	2.60753700000000	0.52633300000000	-5.72255300000000
H	1.97823500000000	1.82621200000000	-6.78586200000000
H	0.55442300000000	-3.38415900000000	-2.30678000000000
H	0.83181800000000	-2.45194500000000	-0.80026700000000
H	1.24897700000000	-4.16039500000000	-0.85808400000000
H	0.35508500000000	-6.28573700000000	-1.44648400000000
H	-0.27074600000000	-8.62687900000000	-1.90759100000000
H	-2.66332200000000	-9.31596900000000	-1.84858000000000
H	-4.42615300000000	-7.63130800000000	-1.35289800000000
H	-3.80725500000000	-5.28273100000000	-0.88575800000000
H	-0.67783200000000	-3.01128700000000	1.64256800000000
H	-2.42032200000000	-2.97662800000000	1.36091500000000
H	-1.90618500000000	-4.72625900000000	3.03874300000000
H	-2.49490400000000	-5.50891300000000	1.57394500000000
H	0.15191500000000	-5.60942100000000	0.96974100000000
H	0.33764900000000	-5.46652400000000	2.69591000000000
H	-1.13940000000000	-7.50266900000000	3.00707300000000
H	-1.39922500000000	-7.56000400000000	1.25423200000000
H	1.07271600000000	-7.87520200000000	0.90711300000000
H	1.31278200000000	-7.89992300000000	2.65874800000000
H	1.18862000000000	-10.24435200000000	1.75104700000000
H	-0.19817400000000	-9.93531500000000	2.82437700000000
H	-0.41856200000000	-9.89726700000000	1.05965500000000

Cat-Si2

C	8.26288200000000	0.60407900000000	-0.90431900000000
C	6.87986100000000	0.84349300000000	-1.06552300000000
C	6.11951300000000	-0.04457800000000	-1.90889300000000
C	6.78961600000000	-1.11980300000000	-2.54415200000000
C	8.14112700000000	-1.30750900000000	-2.37505500000000
C	8.87753300000000	-0.44488500000000	-1.54720300000000
C	6.19821200000000	1.90946400000000	-0.43887000000000
C	4.84738900000000	2.10272100000000	-0.60358300000000
C	4.08425800000000	1.25323400000000	-1.44164800000000
C	4.73650000000000	0.20893800000000	-2.07855000000000
S	2.29199900000000	1.42186100000000	-1.60598600000000
O	2.10600500000000	2.94099400000000	-1.41205600000000
F	4.05040200000000	-0.60430800000000	-2.89302700000000
F	6.86757700000000	2.76522300000000	0.36298200000000
F	4.28059000000000	3.12713200000000	0.04910500000000
F	6.13022700000000	-1.99829000000000	-3.31797700000000
F	8.76959800000000	-2.33782900000000	-2.96450200000000
F	10.18184700000000	-0.69435700000000	-1.34883200000000
F	9.01441900000000	1.36710700000000	-0.08380300000000
N	1.66132600000000	0.76476900000000	-0.36452000000000
P	1.36593600000000	-0.79653900000000	-0.05911800000000
O	2.03910000000000	-1.63233800000000	-1.28203700000000
C	2.12179800000000	-3.01437100000000	-1.05592700000000
C	3.13446000000000	-3.47624700000000	-0.23468200000000
C	3.17278100000000	-4.87122800000000	0.08024200000000
C	2.20066000000000	-5.73470300000000	-0.52431000000000
C	1.22133100000000	-5.19572000000000	-1.38731700000000
C	1.14079000000000	-3.84109400000000	-1.65436600000000
C	4.08223700000000	-5.42981500000000	1.01295800000000
C	4.05509800000000	-6.77470500000000	1.30991400000000
C	3.11993900000000	-7.62995500000000	0.68815000000000
C	2.21219900000000	-7.11709400000000	-0.20946600000000

C	4.08603100000000	-2.51802800000000	0.37058700000000
C	3.60564100000000	-1.44497700000000	1.10440000000000
C	4.43499600000000	-0.54149500000000	1.82483600000000
C	5.79933200000000	-0.78498300000000	1.77897100000000
C	6.35746900000000	-1.83633300000000	1.01986200000000
C	5.49907000000000	-2.69575800000000	0.26009400000000
C	7.75492800000000	-2.08216700000000	1.01091900000000
C	8.28641300000000	-3.10646000000000	0.26045500000000
C	7.44425000000000	-3.90397100000000	-0.54635600000000
C	6.08244900000000	-3.69770100000000	-0.55371200000000
O	2.22554700000000	-1.29373700000000	1.21983300000000
C	3.88878500000000	0.55165700000000	2.65459700000000
C	2.66823700000000	0.44852400000000	3.34064800000000
C	2.20740200000000	1.48346300000000	4.14740400000000
C	2.93763600000000	2.66376700000000	4.31641900000000
C	4.16906700000000	2.75395700000000	3.65053800000000
C	4.63222600000000	1.72967000000000	2.83555300000000
C	0.07390000000000	-3.31107900000000	-2.52217800000000
C	0.32955300000000	-2.39001800000000	-3.54529900000000
C	-0.67976700000000	-2.01898300000000	-4.43227700000000
C	-1.97768600000000	-2.53347800000000	-4.32371400000000
C	-2.23190200000000	-3.41483000000000	-3.26407500000000
C	-1.23239500000000	-3.79691800000000	-2.38068200000000
C	-3.08221000000000	-2.23067000000000	-5.33855500000000
C	-4.34578500000000	-1.70998200000000	-4.62943500000000
C	2.44859900000000	3.81540800000000	5.19329400000000
C	3.33969300000000	3.86999900000000	6.45026700000000
N	-0.09201100000000	-1.20953000000000	0.25436700000000
P	-1.51626600000000	-0.53451200000000	0.35784400000000
O	-2.41090600000000	-1.41812000000000	1.39233100000000
C	-2.68678700000000	-2.72297100000000	0.99214500000000
C	-3.69969300000000	-2.90448400000000	0.06757200000000
C	-3.99096400000000	-4.23379300000000	-0.37284000000000

C	-3.23952400000000	-5.31830100000000	0.19166500000000
C	-2.21105000000000	-5.05420600000000	1.12365600000000
C	-1.88793500000000	-3.76833700000000	1.52079500000000
C	-4.93962400000000	-4.52716000000000	-1.38443900000000
C	-5.15902800000000	-5.82330200000000	-1.79596800000000
C	-4.44198100000000	-6.89310800000000	-1.21745500000000
C	-3.50266100000000	-6.64189100000000	-0.24449000000000
C	-4.35716200000000	-1.71953200000000	-0.53437100000000
C	-3.57977800000000	-0.74664200000000	-1.14779400000000
C	-4.11213100000000	0.35230200000000	-1.87533900000000
C	-5.49385000000000	0.42631800000000	-1.95635300000000
C	-6.34200100000000	-0.49651900000000	-1.30791600000000
C	-5.77442400000000	-1.56807300000000	-0.54535400000000
C	-7.75390800000000	-0.39532900000000	-1.39909900000000
C	-8.56848100000000	-1.29318100000000	-0.74897200000000
C	-8.00479300000000	-2.30943300000000	0.05500500000000
C	-6.63835400000000	-2.43852900000000	0.16378000000000
O	-2.19468700000000	-0.88626200000000	-1.08135600000000
C	-3.26390200000000	1.30842100000000	-2.61798800000000
C	-2.05319600000000	0.92049900000000	-3.21690700000000
C	-1.34744400000000	1.79206000000000	-4.03334900000000
C	-1.80735100000000	3.08727500000000	-4.30685900000000
C	-2.99793700000000	3.48261000000000	-3.68500000000000
C	-3.70350400000000	2.61933900000000	-2.84889200000000
C	-0.72898000000000	-3.51490600000000	2.39838200000000
C	-0.74703000000000	-2.58198800000000	3.44243400000000
C	0.35697200000000	-2.43586700000000	4.28274300000000
C	1.51450200000000	-3.20563900000000	4.11821800000000
C	1.53905700000000	-4.10053900000000	3.03898100000000
C	0.44850600000000	-4.25042300000000	2.19513800000000
C	2.69035900000000	-3.17336300000000	5.09776600000000
C	4.01620600000000	-2.90939600000000	4.35991300000000
C	-1.00505600000000	3.99280000000000	-5.24313100000000

C	-0.81559400000000	3.26446700000000	-6.58925500000000
N	-1.69040100000000	1.00248500000000	0.70184100000000
S	-2.21895600000000	1.57615800000000	2.10831300000000
O	-1.65139200000000	0.88645300000000	3.26016900000000
C	-4.00622100000000	1.15678300000000	2.03443700000000
C	-4.83922500000000	1.88639400000000	1.15547600000000
C	-6.19611200000000	1.68200700000000	1.09901400000000
C	-6.82027800000000	0.68242700000000	1.87594200000000
C	-5.98346100000000	-0.14007700000000	2.71553700000000
C	-4.59579800000000	0.15918600000000	2.78794400000000
C	-8.21400400000000	0.44909200000000	1.86576200000000
C	-8.76359000000000	-0.57765500000000	2.59634100000000
C	-7.94825500000000	-1.41220500000000	3.37641800000000
C	-6.59117300000000	-1.19412000000000	3.44228300000000
F	-4.31671200000000	2.81807200000000	0.34039100000000
F	-3.85849600000000	-0.56875300000000	3.64384000000000
F	-6.92259700000000	2.44636400000000	0.25172000000000
F	-5.86182800000000	-2.03928100000000	4.19781000000000
F	-8.51315000000000	-2.44739700000000	4.02920200000000
F	-10.08402300000000	-0.83366000000000	2.52908700000000
F	-9.04275100000000	1.20710100000000	1.11882600000000
O	-2.15636900000000	3.02860200000000	2.05945500000000
C	-1.71435000000000	5.32594900000000	-5.51054700000000
C	0.37536100000000	4.28847200000000	-4.62045100000000
C	2.75834200000000	-4.55464800000000	5.78347600000000
C	2.52142900000000	-2.09806700000000	6.17888800000000
C	2.55830300000000	5.15016300000000	4.43020600000000
C	0.98799600000000	3.62437300000000	5.62295800000000
C	-3.40908600000000	-3.54768400000000	-6.07274000000000
C	-2.65180800000000	-1.18106500000000	-6.37190000000000
O	1.91065400000000	0.99896300000000	-2.93596600000000
C	1.17220900000000	3.21947600000000	1.44491000000000
Si	0.98093900000000	3.81950600000000	-0.30096100000000

H	3.39491300000000	-2.11702600000000	6.85014100000000
H	2.42333700000000	-4.70824700000000	2.85133500000000
H	0.51429600000000	-4.93712900000000	1.35094400000000
H	-1.46767800000000	-4.47360200000000	-1.55861900000000
H	-3.22932100000000	-3.82806200000000	-3.12272400000000
H	-3.48629900000000	-0.99102400000000	-7.06582300000000
H	-1.79311300000000	-1.52564000000000	-6.96991200000000
H	-2.39235800000000	-0.22563600000000	-5.88948600000000
H	-5.17606700000000	-1.63740000000000	-5.35120800000000
H	-4.16911900000000	-0.71042200000000	-4.21271600000000
H	-4.66684400000000	-2.36788400000000	-3.80876500000000
H	-4.16773600000000	-3.36820700000000	-6.85180000000000
H	-3.80124400000000	-4.30477500000000	-5.37597200000000
H	-2.50455700000000	-3.95964700000000	-6.54781900000000
H	-0.43547600000000	-1.31458100000000	-5.22705300000000
H	1.33055400000000	-1.97607600000000	-3.66993800000000
H	0.50014200000000	-5.86879500000000	-1.85495500000000
H	1.47274100000000	-7.76569900000000	-0.68452100000000
H	3.11289100000000	-8.69473700000000	0.92897500000000
H	4.75914200000000	-7.18081200000000	2.03890100000000
H	4.80540100000000	-4.78469800000000	1.50960000000000
H	5.43845200000000	-4.31157600000000	-1.18412400000000
H	7.87826200000000	-4.68007300000000	-1.17925100000000
H	9.36410100000000	-3.28282500000000	0.26009900000000
H	8.40488500000000	-1.43443600000000	1.60489100000000
H	6.46928300000000	-0.17037300000000	2.38362700000000
H	5.57884700000000	1.86461100000000	2.31162800000000
H	4.77706400000000	3.65499300000000	3.74438100000000
H	0.31792600000000	3.51815900000000	4.75487500000000
H	0.65982200000000	4.50045600000000	6.20396600000000
H	0.86472800000000	2.73767700000000	6.26428100000000
H	4.39428300000000	4.03965400000000	6.18275800000000
H	3.27828800000000	2.92170800000000	7.00693700000000

H	3.01365500000000	4.68854000000000	7.11251600000000
H	3.59564500000000	5.38419800000000	4.14818400000000
H	2.19104100000000	5.97224000000000	5.06556100000000
H	1.95525400000000	5.12559400000000	3.51175100000000
H	1.23542500000000	1.35932200000000	4.62402700000000
H	2.06120400000000	-0.44921200000000	3.25788400000000
H	-4.60866200000000	2.97901200000000	-2.35637400000000
H	-3.38902400000000	4.48814500000000	-3.83733600000000
H	0.26756900000000	4.89247300000000	-3.70782800000000
H	0.99297300000000	4.85890100000000	-5.33449400000000
H	0.91210000000000	3.36288100000000	-4.36126300000000
H	-1.79189100000000	2.99755200000000	-7.02397200000000
H	-0.23071900000000	2.34013500000000	-6.47171800000000
H	-0.28076000000000	3.91614100000000	-7.29914800000000
H	-1.11576900000000	5.92383200000000	-6.21629200000000
H	-1.82722600000000	5.91739300000000	-4.58913800000000
H	-2.70960600000000	5.17065900000000	-5.95716000000000
H	-0.40743900000000	1.44168500000000	-4.46243700000000
H	-1.66860500000000	-0.08416800000000	-3.07336600000000
H	-5.95052400000000	1.20733700000000	-2.56652900000000
H	-8.18370600000000	0.42156800000000	-1.98264700000000
H	-9.65370800000000	-1.19867400000000	-0.81915400000000
H	-8.65968400000000	-2.98809800000000	0.60616600000000
H	-6.20636100000000	-3.21770500000000	0.79338400000000
H	-5.50102400000000	-3.71536300000000	-1.84483500000000
H	-5.89154500000000	-6.02328300000000	-2.58028800000000
H	-4.62978500000000	-7.91596500000000	-1.54906800000000
H	-2.93092200000000	-7.45952900000000	0.20061300000000
H	-1.64488000000000	-5.89273500000000	1.53376300000000
H	-1.62882400000000	-1.96704200000000	3.61587900000000
H	0.28898200000000	-1.70824800000000	5.09156500000000
H	4.05168600000000	-1.88327000000000	3.97281300000000
H	4.86239400000000	-3.03637600000000	5.05532600000000

H	4.16436600000000	-3.59858000000000	3.51612900000000
H	1.80439700000000	-4.78325800000000	6.28444600000000
H	3.56501800000000	-4.56565900000000	6.53502600000000
H	2.95632100000000	-5.35271500000000	5.05141300000000
H	2.45954400000000	-1.08973900000000	5.74110100000000
H	1.62359300000000	-2.27601100000000	6.79169500000000
H	0.58096400000000	3.87756400000000	2.10208500000000
H	0.77271000000000	2.20626700000000	1.56785500000000
H	2.22176000000000	3.23607200000000	1.76851400000000
C	-0.73867700000000	3.67144800000000	-1.00199700000000
C	1.70027600000000	5.53933700000000	-0.51060500000000
C	2.35896400000000	5.94622300000000	-1.68262400000000
C	2.73366900000000	7.27570200000000	-1.86546700000000
C	2.45381900000000	8.22378700000000	-0.88057000000000
C	1.81757700000000	7.83392000000000	0.29688100000000
C	1.45162300000000	6.50170000000000	0.48199900000000
H	2.56918800000000	5.21861500000000	-2.46901700000000
H	3.24077500000000	7.57677700000000	-2.78476000000000
H	2.73605000000000	9.26814400000000	-1.03000500000000
H	1.60364100000000	8.57087400000000	1.07383400000000
H	0.94876900000000	6.21683600000000	1.40899100000000
C	-1.61014000000000	4.86435700000000	-0.55210300000000
H	-0.70923500000000	3.61107600000000	-2.09904900000000
H	-1.16389200000000	2.72110100000000	-0.63977100000000
H	-2.66715200000000	4.55235600000000	-0.54970700000000
H	-1.39605600000000	5.12103700000000	0.50205800000000
C	-1.42938700000000	6.10463500000000	-1.42998600000000
C	-1.84925000000000	7.40539700000000	-0.75273000000000
H	-0.37159400000000	6.20600200000000	-1.72328100000000
H	-1.99181200000000	5.96891300000000	-2.37048600000000
C	-1.50051200000000	8.64169600000000	-1.57876300000000
H	-2.93275700000000	7.39041000000000	-0.52733600000000
H	-1.33143900000000	7.47542400000000	0.22262000000000

C	-1.81117400000000	9.94860500000000	-0.85349700000000
H	-0.42277300000000	8.60422500000000	-1.82331000000000
H	-2.04177600000000	8.60802700000000	-2.54253300000000
H	-1.54893700000000	10.82603900000000	-1.46496800000000
H	-2.88220300000000	10.01888900000000	-0.60214000000000
H	-1.24367100000000	10.01221400000000	0.08957200000000

TS

C	-8.22924300000000	-0.95225600000000	-0.98430000000000
C	-6.84156700000000	-1.21174100000000	-1.05967300000000
C	-6.02744900000000	-0.35970400000000	-1.89021700000000
C	-6.65132900000000	0.68441200000000	-2.61885900000000
C	-8.00872200000000	0.88490400000000	-2.53925900000000
C	-8.79677600000000	0.06823200000000	-1.70965600000000
C	-6.20836900000000	-2.26915000000000	-0.37298200000000
C	-4.85213200000000	-2.49117000000000	-0.46539400000000
C	-4.03313600000000	-1.65941100000000	-1.26046200000000
C	-4.64244400000000	-0.63111000000000	-1.96063900000000
S	-2.25439000000000	-1.92857400000000	-1.51948200000000
O	-1.96223400000000	-3.22560900000000	-0.83697500000000
F	-3.89029800000000	0.14246000000000	-2.75815400000000
F	-6.93159100000000	-3.09580700000000	0.41667400000000
F	-4.34741300000000	-3.52692700000000	0.22218700000000
F	-5.93923600000000	1.50882900000000	-3.40666100000000
F	-8.60217100000000	1.87657600000000	-3.22506000000000
F	-10.11029500000000	0.33215600000000	-1.60351400000000
F	-9.03173900000000	-1.67835200000000	-0.17716600000000
N	-1.47448200000000	-0.82268300000000	-0.75244000000000
P	-1.36475500000000	0.68651100000000	-0.32380400000000
O	-1.96744900000000	1.62070500000000	-1.51255800000000
C	-2.09605100000000	2.97892500000000	-1.20039800000000
C	-3.17043700000000	3.37142600000000	-0.42379500000000
C	-3.27548300000000	4.75068400000000	-0.04866400000000
C	-2.30076800000000	5.67306900000000	-0.55249600000000

C	-1.25040200000000	5.20487100000000	-1.37172300000000
C	-1.10731700000000	3.86778800000000	-1.69111200000000
C	-4.25983200000000	5.24035100000000	0.84674100000000
C	-4.30323900000000	6.57255300000000	1.19487100000000
C	-3.36649500000000	7.48612000000000	0.66594200000000
C	-2.38378600000000	7.04035900000000	-0.18738100000000
C	-4.12683700000000	2.37251500000000	0.10098500000000
C	-3.66606200000000	1.29688300000000	0.84597000000000
C	-4.52633200000000	0.45801200000000	1.61303400000000
C	-5.88552300000000	0.70917200000000	1.53668900000000
C	-6.42255100000000	1.72263600000000	0.71398100000000
C	-5.53775700000000	2.54956700000000	-0.05007200000000
C	-7.81762300000000	1.97116100000000	0.65660100000000
C	-8.32385700000000	2.96612700000000	-0.14924700000000
C	-7.45442500000000	3.73108700000000	-0.95800000000000
C	-6.09308100000000	3.52616100000000	-0.91231800000000
O	-2.29822500000000	1.08793400000000	0.95720900000000
C	-4.00984900000000	-0.56579300000000	2.54211800000000
C	-2.86036400000000	-0.35748400000000	3.31647800000000
C	-2.41396400000000	-1.32680500000000	4.20923000000000
C	-3.06790800000000	-2.55479800000000	4.34745100000000
C	-4.24406300000000	-2.73835800000000	3.60639100000000
C	-4.71222100000000	-1.76524700000000	2.73445200000000
C	0.03035600000000	3.42559000000000	-2.51760500000000
C	-0.13543500000000	2.59007600000000	-3.62826300000000
C	0.93600500000000	2.31815300000000	-4.47766500000000
C	2.20987400000000	2.84902000000000	-4.24193400000000
C	2.37361500000000	3.64224600000000	-3.09810000000000
C	1.31130400000000	3.92687300000000	-2.25189100000000
C	3.38451700000000	2.65788100000000	-5.20337900000000
C	4.60521800000000	2.08294200000000	-4.46220200000000
C	-2.54394100000000	-3.68319200000000	5.23443300000000
C	-3.53728100000000	-3.90148500000000	6.39218500000000

N	0.05432100000000	1.17827900000000	0.13865000000000
P	1.49076300000000	0.57234700000000	0.27097700000000
O	2.33315300000000	1.35347400000000	1.42609700000000
C	2.64280900000000	2.68584300000000	1.12726100000000
C	3.71103100000000	2.92647700000000	0.27917300000000
C	4.01248900000000	4.28194000000000	-0.07145900000000
C	3.22239600000000	5.32931100000000	0.50789400000000
C	2.14463300000000	5.00625200000000	1.36086500000000
C	1.80855600000000	3.69797100000000	1.66128600000000
C	5.00491000000000	4.63809700000000	-1.01981600000000
C	5.23191000000000	5.95636700000000	-1.34866900000000
C	4.47993200000000	6.98835500000000	-0.74722100000000
C	3.49472600000000	6.67733500000000	0.16076400000000
C	4.40754200000000	1.79248900000000	-0.37203000000000
C	3.66048400000000	0.87134400000000	-1.09354000000000
C	4.23126100000000	-0.15281000000000	-1.89787600000000
C	5.61592600000000	-0.19439000000000	-1.95541300000000
C	6.43335300000000	0.68735000000000	-1.21877800000000
C	5.82911200000000	1.66622500000000	-0.36494400000000
C	7.84746400000000	0.64949000000000	-1.32779000000000
C	8.63344100000000	1.51037700000000	-0.59667400000000
C	8.03703000000000	2.41821100000000	0.30764200000000
C	6.66660200000000	2.48935300000000	0.42794200000000
O	2.27384500000000	1.01023600000000	-1.08606400000000
C	3.42571500000000	-1.05765500000000	-2.74073900000000
C	2.21763500000000	-0.65366400000000	-3.33421900000000
C	1.54277500000000	-1.48512000000000	-4.21499400000000
C	2.03300900000000	-2.75146100000000	-4.56042300000000
C	3.22987300000000	-3.15609100000000	-3.95819900000000
C	3.90700700000000	-2.33457900000000	-3.06098200000000
C	0.59418300000000	3.40875000000000	2.44784300000000
C	0.56575900000000	2.50720100000000	3.51749100000000
C	-0.58016300000000	2.37902200000000	4.30425800000000

C	-1.73559100000000	3.12890000000000	4.04858600000000
C	-1.71675400000000	3.97174200000000	2.92862000000000
C	-0.58176400000000	4.10938300000000	2.14388300000000
C	-2.95011000000000	3.14627600000000	4.98074500000000
C	-4.25401700000000	2.88385400000000	4.20345500000000
C	1.29016300000000	-3.59917500000000	-5.59245300000000
C	1.36281000000000	-2.86345800000000	-6.94695800000000
N	1.68760300000000	-0.99390200000000	0.48660400000000
S	2.11644800000000	-1.82007200000000	1.74687400000000
O	1.68648500000000	-1.33980300000000	3.04673900000000
C	3.92295100000000	-1.72300400000000	1.70551200000000
C	4.67181900000000	-2.45008200000000	0.75314800000000
C	6.02809400000000	-2.27902100000000	0.62175300000000
C	6.73595600000000	-1.35413700000000	1.42149200000000
C	5.98796600000000	-0.56684800000000	2.36947500000000
C	4.59432800000000	-0.79761800000000	2.48952800000000
C	8.13243600000000	-1.15832900000000	1.33335500000000
C	8.76408800000000	-0.21186400000000	2.10521900000000
C	8.03195200000000	0.58401200000000	2.99962300000000
C	6.67520900000000	0.40977400000000	3.13191000000000
F	4.06422800000000	-3.32479000000000	-0.06249800000000
F	3.92972200000000	-0.06945500000000	3.39710700000000
F	6.67028400000000	-3.00084400000000	-0.32325500000000
F	6.02189400000000	1.23509500000000	3.97124200000000
F	8.66853600000000	1.55820900000000	3.67501700000000
F	10.08332700000000	0.00695700000000	1.97011500000000
F	8.88165100000000	-1.86327900000000	0.46208800000000
O	1.76294200000000	-3.24577500000000	1.42842100000000
C	1.91857600000000	-4.98887000000000	-5.75817900000000
C	-0.18342300000000	-3.77127100000000	-5.17930200000000
C	-3.01469500000000	4.55405500000000	5.61218000000000
C	-2.84091100000000	2.11148000000000	6.10793600000000
C	-2.42140400000000	-4.97581500000000	4.40189700000000

C	-1.16431100000000	-3.35677800000000	5.82156400000000
C	3.73940200000000	4.04294800000000	-5.78347100000000
C	3.04173000000000	1.70587500000000	-6.35696500000000
O	-2.05795800000000	-1.91940400000000	-2.95830200000000
C	-0.94559100000000	-2.37800300000000	1.68568700000000
Si	-0.12573900000000	-3.45766500000000	0.38924800000000
H	-3.73808100000000	2.17217800000000	6.74440900000000
H	-2.59802300000000	4.55543900000000	2.66740200000000
H	-0.60452700000000	4.76523700000000	1.27347300000000
H	1.47506600000000	4.53952200000000	-1.36436000000000
H	3.34894000000000	4.06433300000000	-2.86075500000000
H	3.92195000000000	1.59407900000000	-7.01022300000000
H	2.21520400000000	2.09298400000000	-6.97391000000000
H	2.77129500000000	0.70544600000000	-5.98287100000000
H	5.48477700000000	2.08995000000000	-5.12691600000000
H	4.41482600000000	1.04530000000000	-4.16025300000000
H	4.85721700000000	2.65947800000000	-3.56026700000000
H	4.55638000000000	3.94876700000000	-6.51741900000000
H	4.06522400000000	4.73366600000000	-4.99028600000000
H	2.86444300000000	4.48854200000000	-6.28274400000000
H	0.76113700000000	1.68088700000000	-5.34417100000000
H	-1.11862700000000	2.17381300000000	-3.85084800000000
H	-0.52651400000000	5.92073100000000	-1.76650400000000
H	-1.64095300000000	7.73242100000000	-0.59080400000000
H	-3.41740500000000	8.54058300000000	0.94349200000000
H	-5.06769800000000	6.92211300000000	1.89173300000000
H	-4.98693000000000	4.55288300000000	1.27484100000000
H	-5.42626700000000	4.11856800000000	-1.53999100000000
H	-7.86668300000000	4.48318400000000	-1.63307200000000
H	-9.40049400000000	3.14285700000000	-0.19121200000000
H	-8.48644200000000	1.34889900000000	1.25683500000000
H	-6.56615800000000	0.13381800000000	2.16793600000000
H	-5.62460200000000	-1.95990200000000	2.17181900000000

H	-4.79969300000000	-3.67370700000000	3.68582700000000
H	-0.41810100000000	-3.17467600000000	5.03201700000000
H	-0.81447500000000	-4.20909900000000	6.42495000000000
H	-1.19933100000000	-2.47399400000000	6.47972000000000
H	-4.53693300000000	-4.16799000000000	6.01601600000000
H	-3.63409700000000	-2.98533700000000	6.99621300000000
H	-3.18643200000000	-4.71769300000000	7.04459700000000
H	-3.39402400000000	-5.30843300000000	4.01032300000000
H	-2.01567600000000	-5.78725300000000	5.02731600000000
H	-1.74606700000000	-4.83723900000000	3.54574100000000
H	-1.49663300000000	-1.12662400000000	4.76243400000000
H	-2.29253700000000	0.56531300000000	3.21656000000000
H	4.81560500000000	-2.70724600000000	-2.58468200000000
H	3.64839600000000	-4.13852100000000	-4.17531800000000
H	-0.26363400000000	-4.30180200000000	-4.22019500000000
H	-0.71723600000000	-4.35914300000000	-5.94400200000000
H	-0.70192000000000	-2.80920400000000	-5.06600900000000
H	2.41143400000000	-2.70355000000000	-7.24440400000000
H	0.86994000000000	-1.88066600000000	-6.89270000000000
H	0.86062300000000	-3.45681800000000	-7.72851100000000
H	1.33858100000000	-5.56666700000000	-6.49501800000000
H	1.91306200000000	-5.55013100000000	-4.81101200000000
H	2.95588800000000	-4.92602100000000	-6.12415100000000
H	0.60536500000000	-1.12634900000000	-4.64320200000000
H	1.81070900000000	0.33134000000000	-3.13198300000000
H	6.09776400000000	-0.90446800000000	-2.62957500000000
H	8.30200500000000	-0.08821500000000	-1.99271600000000
H	9.72082000000000	1.46877000000000	-0.68438000000000
H	8.66888600000000	3.05890400000000	0.92693800000000
H	6.21058000000000	3.19034600000000	1.12894500000000
H	5.59283800000000	3.85948800000000	-1.50237600000000
H	5.99744700000000	6.20251000000000	-2.08706000000000
H	4.67453300000000	8.02922800000000	-1.01255300000000

H	2.89200100000000	7.46453500000000	0.61929900000000
H	1.54343600000000	5.81410900000000	1.78254000000000
H	1.45193800000000	1.91959100000000	3.75984000000000
H	-0.55038400000000	1.68876200000000	5.14744500000000
H	-4.29393200000000	1.85181100000000	3.83260100000000
H	-5.11951100000000	3.03733300000000	4.86896900000000
H	-4.36511100000000	3.56078500000000	3.34459700000000
H	-2.07542200000000	4.78362400000000	6.13993900000000
H	-3.84807000000000	4.60805800000000	6.33195400000000
H	-3.16962400000000	5.32671200000000	4.84344600000000
H	-2.78223200000000	1.08582200000000	5.71368600000000
H	-1.96460200000000	2.29975300000000	6.74831000000000
H	-0.61840200000000	-2.78510100000000	2.65297200000000
H	-0.72135400000000	-1.30948100000000	1.67619000000000
H	-2.03267000000000	-2.51911800000000	1.60764400000000
C	0.88942600000000	-3.46479500000000	-1.21819700000000
C	-0.50716300000000	-5.19918400000000	1.02866400000000
C	-1.70680100000000	-5.85019400000000	0.69299400000000
C	-2.02059800000000	-7.09709200000000	1.23114300000000
C	-1.14893800000000	-7.71718800000000	2.12467400000000
C	0.03961400000000	-7.08179900000000	2.47795400000000
C	0.35667000000000	-5.83605200000000	1.93646900000000
H	-2.40824200000000	-5.37970500000000	0.00231400000000
H	-2.95605300000000	-7.58693800000000	0.95047700000000
H	-1.39788900000000	-8.69302400000000	2.54762500000000
H	0.72910200000000	-7.55787300000000	3.17905100000000
H	1.28926400000000	-5.35308300000000	2.23245000000000
C	1.53607300000000	-4.85354600000000	-1.38856700000000
H	0.25490200000000	-3.23304800000000	-2.08502600000000
H	1.65144200000000	-2.67702500000000	-1.16179500000000
H	2.40186800000000	-4.75484400000000	-2.06386900000000
H	1.95410300000000	-5.19751700000000	-0.42600100000000
C	0.56606900000000	-5.91270300000000	-1.93371000000000

C	0.83915300000000	-7.32051000000000	-1.41395800000000
H	-0.47055200000000	-5.64182300000000	-1.66962400000000
H	0.59520500000000	-5.91064700000000	-3.03719000000000
C	-0.17781000000000	-8.34919900000000	-1.90260200000000
H	1.86138700000000	-7.63795700000000	-1.69818400000000
H	0.81538600000000	-7.29668300000000	-0.31009600000000
C	-0.00028800000000	-9.71066000000000	-1.23457500000000
H	-1.19298200000000	-7.96692700000000	-1.68884100000000
H	-0.10679900000000	-8.45237400000000	-3.00144200000000
H	-0.73036600000000	-10.44651100000000	-1.60713700000000
H	1.00962000000000	-10.11315700000000	-1.41825000000000
H	-0.13152000000000	-9.62022100000000	-0.14384000000000

8. Absolute configuration determination

i. CD experiments for determination of the absolute configuration of compounds 1a and 4a

Method:

The CD-spectra of (*R*)-**1a** (c = 8.0 x 10^{-4} M), (*S*)-**1a** (c = 8.0 x 10^{-4} M) and (*S*)-**4a** (c = 6.0 x 10^{-4} M) were recorded in hexane (HPLC grade) at 20 °C and compared with the corresponding TD-DFT calculated CD spectra of the possible conformers. After a UV correction of −24 nm to −30 nm and a correction of the σ-value of 0.3 eV, the CD characteristics of the calculated spectra (blue curves) were in good agreement with the experimental spectra (red curves), thus allowing the assignment of the absolute configuration of both enantiomers of chiral silicon compounds **1a**, and the absolute configuration of **4a** that was generated from the catalytic reaction could be assigned to be *S*, using (*S*, *S*)-IDPi as catalyst.

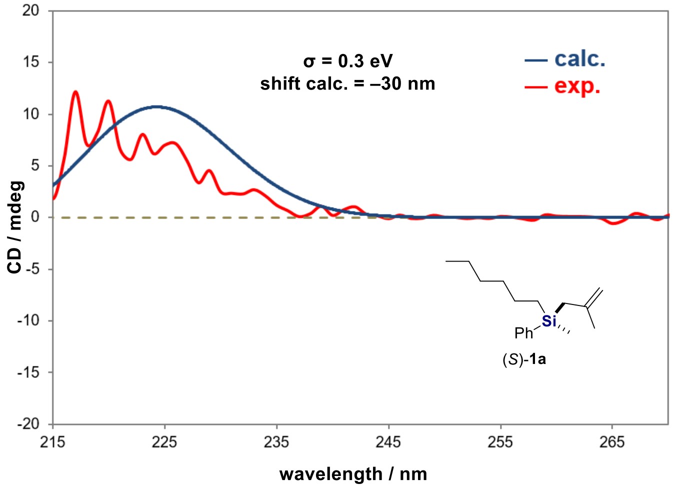

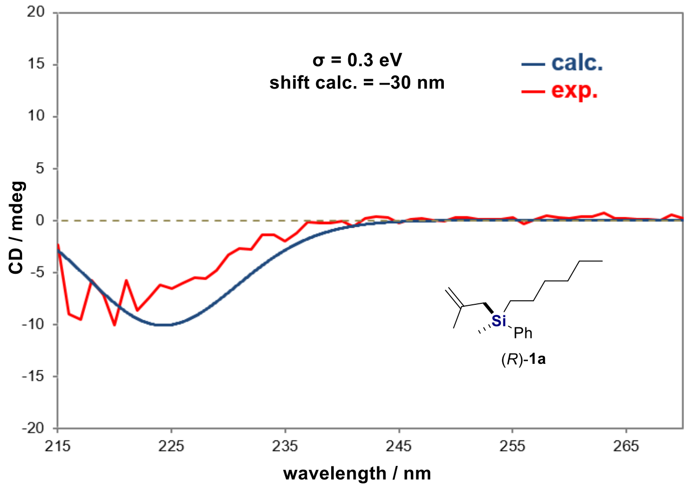

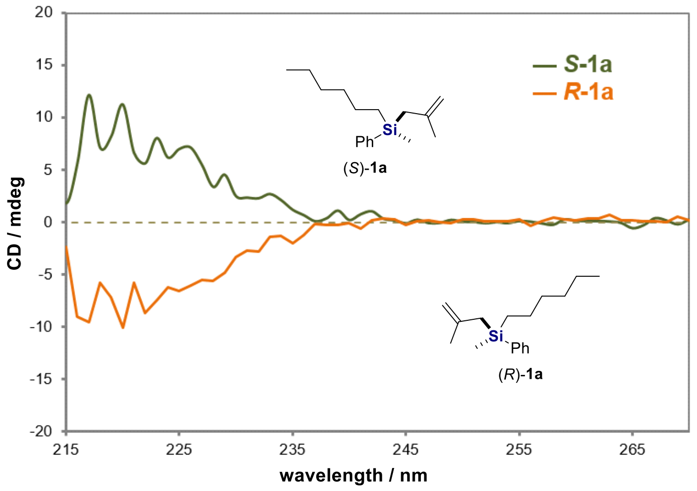

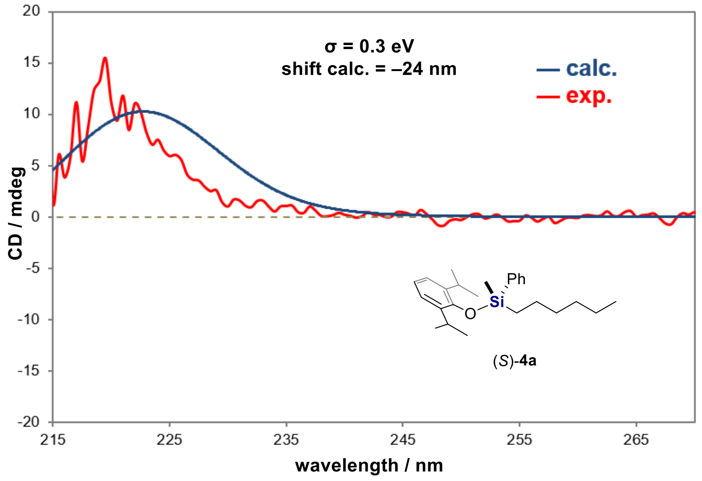

CD spectra calculation

Method 1: : Staring from the initial guess structures, molecular dynamics (MD) simulations with xTB[17] employing GFN2-xTB method were performed to sample the conformers. Density Functional Theory (DFT) calculations of compounds (*R*)-**1a** and (*S*)-**1a** were performed on the Max-Planck-Institut für Kohlenforschung computer cluster using the ORCA program package (Version 5.0-Stable).[18] Structural optimizations and frequency calculations to identify all of the stationary points as minima (zero imaginary frequencies) and to obtain thermal and entropic correction were performed with the B3LYP functional[19,20] with D3 dispersion correction[14] and Becke-Johnson damping (BJ)[15] along with RI approximation, utilizing the def2/J auxiliary basis set[21] and the def2-SVP basis set[22] on all atoms. The libint2 library was used for the computation of 2-el integrals.[23] Tight SCF convergence and geometry optimization criterions were chosen.

The CD spectrum was computed by time-dependent density functional theory (TD-DFT, NROOTS = 25) at B3LYP/TZVP level, and solvent effects of hexane were taken into account using the conductor-like polarized continuum model (CPCM).[24] The CD spectrum was created using Multiwfn[25] with a 0.3 eV half-width at half-height.

Method 2: Staring from the initial guess structures, molecular dynamics (MD) simulations with xTB employing GFN2-xTB method were performed to sample the conformers. Density Functional Theory (DFT) calculations of compound **4a** were carried out using the Gaussian 16C01 program package.[26] Structural optimizations and frequency calculations to identify all of the stationary points as minima (zero imaginary frequencies) and to obtain thermal and entropic correction were performed with the apfd functional with D3 dispersion

correction and Becke-Johnson damping (BJ) along with RI approximation, utilizing the 6-311+g(2d,p) basis set on all atoms. The libint2 library was used for the computation of 2-el integrals. Tight SCF convergence and geometry optimization criterions were chosen.

The CD spectrum was computed by time-dependent density functional theory (TD-DFT, NROOTS = 25) at 6-311+g(2d,p) level, and solvent effects of hexane were taken into account using the conductor-like polarized continuum model (IEFPCM). The CD spectrum was created using Multiwfn with a 0.3 eV half-width at half-height.

Calculated coordinates:

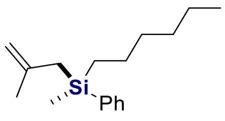

(*R*)-**1a**

C	-1.697743000000	1.568277000000	-1.711374000000
Si	-1.143815000000	0.950225000000	-0.015126000000
C	-0.118309000000	-0.637523000000	-0.199478000000
C	0.108743000000	-1.243645000000	-1.455262000000
C	0.872528000000	-2.416827000000	-1.571203000000
C	1.427673000000	-3.010161000000	-0.426783000000
C	1.210513000000	-2.427688000000	0.833103000000
C	0.444914000000	-1.257117000000	0.940618000000
C	-2.696436000000	0.575283000000	1.046563000000
C	-3.622416000000	-0.444054000000	0.432493000000
C	-3.299610000000	-1.892188000000	0.701576000000
C	-4.666337000000	-0.083028000000	-0.347847000000
C	-0.147574000000	2.294540000000	0.900236000000
C	0.931855000000	3.020800000000	0.073190000000
C	1.992790000000	2.109284000000	-0.560708000000
C	2.861754000000	1.339667000000	0.437713000000
C	3.764454000000	0.292803000000	-0.221388000000
C	4.619031000000	-0.488247000000	0.776541000000
H	-0.839561000000	1.747074000000	-2.390007000000
H	-2.381873000000	0.838080000000	-2.189027000000
H	-2.250325000000	2.524559000000	-1.605551000000
H	-0.316142000000	-0.790803000000	-2.365221000000
H	1.036842000000	-2.869561000000	-2.561316000000
H	2.030087000000	-3.927197000000	-0.515146000000

H	1.641651000000	-2.888796000000	1.735222000000
H	0.283636000000	-0.817807000000	1.939575000000
H	-2.351922000000	0.250898000000	2.052992000000
H	-3.213548000000	1.549693000000	1.179353000000
H	-4.019071000000	-2.573560000000	0.208206000000
H	-2.277182000000	-2.146633000000	0.350976000000
H	-3.312683000000	-2.101479000000	1.793517000000
H	-4.902776000000	0.976194000000	-0.538848000000
H	-5.318237000000	-0.837371000000	-0.815576000000
H	-0.886183000000	3.035785000000	1.278206000000
H	0.293061000000	1.834514000000	1.812250000000
H	0.433272000000	3.598034000000	-0.735608000000
H	1.438651000000	3.779519000000	0.711536000000
H	2.649159000000	2.719024000000	-1.219905000000
H	1.494553000000	1.376991000000	-1.234959000000
H	3.478316000000	2.058075000000	1.025029000000
H	2.213590000000	0.822294000000	1.177126000000
H	4.418469000000	0.788764000000	-0.973492000000
H	3.124346000000	-0.415082000000	-0.792900000000
H	5.257762000000	-1.241093000000	0.271567000000
H	5.286971000000	0.184114000000	1.355320000000
H	3.980524000000	-1.029696000000	1.505792000000

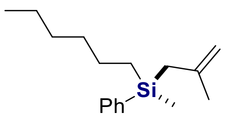

(*S*)-**1a**

C	1.461742000000	-1.257624000000	1.879852000000
Si	1.156517000000	-0.535948000000	0.160498000000
C	0.683577000000	1.293141000000	0.310506000000
C	-0.044742000000	1.942551000000	-0.713184000000
C	-0.344075000000	3.311755000000	-0.638929000000
C	0.082460000000	4.063722000000	0.467753000000
C	0.802806000000	3.438509000000	1.497830000000
C	1.096345000000	2.067965000000	1.418623000000

C	2.766494000000	-0.701052000000	-0.869385000000
C	3.879572000000	0.178089000000	-0.357239000000
C	3.958592000000	1.560547000000	-0.953526000000
C	4.739320000000	-0.233120000000	0.602779000000
C	-0.229318000000	-1.477873000000	-0.734249000000
C	-1.592976000000	-1.439624000000	-0.029124000000
C	-2.701931000000	-2.188135000000	-0.776460000000
C	-4.060358000000	-2.147370000000	-0.072353000000
C	-5.172098000000	-2.890209000000	-0.819694000000
C	-6.524182000000	-2.843414000000	-0.107003000000
H	1.625855000000	-2.353080000000	1.816724000000
H	0.601589000000	-1.076236000000	2.555264000000
H	2.365859000000	-0.807939000000	2.338162000000
H	-0.385611000000	1.369290000000	-1.591209000000
H	-0.913252000000	3.795634000000	-1.447878000000
H	-0.149596000000	5.138215000000	0.528343000000
H	1.137739000000	4.022849000000	2.368942000000
H	1.666103000000	1.594750000000	2.234004000000
H	2.523990000000	-0.464825000000	-1.927715000000
H	3.049644000000	-1.774662000000	-0.828646000000
H	4.753009000000	2.170459000000	-0.482069000000
H	2.993204000000	2.098221000000	-0.845685000000
H	4.165171000000	1.501418000000	-2.044634000000
H	4.689463000000	-1.250330000000	1.023612000000
H	5.527261000000	0.432986000000	0.987635000000
H	0.106981000000	-2.531441000000	-0.865704000000
H	-0.322109000000	-1.071399000000	-1.767117000000
H	-1.904349000000	-0.381468000000	0.120218000000
H	-1.496269000000	-1.864190000000	0.995395000000
H	-2.803135000000	-1.761498000000	-1.800498000000
H	-2.393175000000	-3.247904000000	-0.925685000000
H	-4.364333000000	-1.086378000000	0.078775000000
H	-3.957126000000	-2.574518000000	0.951367000000

H	-5.272170000000	-2.461496000000	-1.841966000000
H	-4.864273000000	-3.949131000000	-0.970603000000
H	-6.873935000000	-1.798008000000	0.025281000000
H	-6.462754000000	-3.298431000000	0.903892000000
H	-7.307390000000	-3.388661000000	-0.671488000000

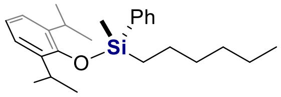

(*S*)-**4a**

C	-1.083696000000	-1.396011000000	2.944640000000
H	-1.781263000000	-0.939502000000	3.652628000000
H	-0.098551000000	-1.434648000000	3.416137000000
H	-1.419077000000	-2.419342000000	2.756856000000
Si	-1.063769000000	-0.414035000000	1.358870000000
O	-0.326910000000	-1.353916000000	0.185759000000
C	0.775082000000	-1.061070000000	-0.572362000000
C	2.050118000000	-1.398081000000	-0.104926000000
C	3.142337000000	-1.148120000000	-0.933530000000
C	2.981292000000	-0.574386000000	-2.181676000000
C	1.708083000000	-0.241065000000	-2.622698000000
C	0.587329000000	-0.479920000000	-1.836650000000
C	-0.811244000000	-0.185027000000	-2.324846000000
C	-1.426087000000	-1.454248000000	-2.921717000000
H	-1.375224000000	-2.285246000000	-2.215063000000
H	-2.474622000000	-1.292005000000	-3.188483000000
H	-0.884449000000	-1.749413000000	-3.825753000000
H	-1.414275000000	0.090146000000	-1.454963000000
C	-0.891488000000	0.971894000000	-3.314994000000
H	-0.413722000000	1.873367000000	-2.921743000000
H	-1.937597000000	1.208504000000	-3.526754000000

H	-0.416794000000	0.727538000000	-4.269851000000
H	1.584854000000	0.215024000000	-3.598854000000
H	3.844489000000	-0.378510000000	-2.809505000000
H	4.138517000000	-1.403405000000	-0.585245000000
C	2.251739000000	-2.041787000000	1.246416000000
C	2.792995000000	-3.465808000000	1.100502000000
H	2.889165000000	-3.945893000000	2.078976000000
H	2.130400000000	-4.077038000000	0.482883000000
H	3.780883000000	-3.465939000000	0.630583000000
C	3.160304000000	-1.203311000000	2.146792000000
H	2.784897000000	-0.183509000000	2.261912000000
H	4.171739000000	-1.134085000000	1.736447000000
H	3.237847000000	-1.651473000000	3.141810000000
H	1.273412000000	-2.116271000000	1.724290000000
C	-2.819257000000	-0.133036000000	0.778888000000
C	-3.536911000000	0.996518000000	1.181102000000
C	-4.847705000000	1.199863000000	0.766194000000
C	-5.464580000000	0.269082000000	-0.061295000000
C	-4.768195000000	-0.863729000000	-0.467417000000
C	-3.458655000000	-1.059937000000	-0.048554000000
H	-2.918847000000	-1.940952000000	-0.381812000000
H	-5.246376000000	-1.592852000000	-1.114415000000
H	-6.486800000000	0.427213000000	-0.390756000000
H	-5.387120000000	2.086963000000	1.083723000000
H	-3.067856000000	1.737859000000	1.823539000000
C	-0.140261000000	1.191165000000	1.582457000000
C	-0.196033000000	2.175718000000	0.413988000000
C	0.626236000000	3.444718000000	0.644854000000

C	2.110358000000	3.197502000000	0.916187000000
C	2.808268000000	2.382254000000	-0.165493000000
C	4.293155000000	2.194352000000	0.109351000000
H	4.771694000000	1.598535000000	-0.672266000000
H	4.452064000000	1.676085000000	1.060506000000
H	4.814951000000	3.155197000000	0.167785000000
H	2.338091000000	1.398981000000	-0.258114000000
H	2.667072000000	2.872115000000	-1.137487000000
H	2.616965000000	4.164665000000	1.026504000000
H	2.234816000000	2.692387000000	1.882858000000
H	0.527824000000	4.090331000000	-0.237399000000
H	0.195353000000	4.006522000000	1.483732000000
H	0.157645000000	1.682399000000	-0.494992000000
H	-1.236471000000	2.453442000000	0.213333000000
H	0.897602000000	0.908380000000	1.793534000000
H	-0.502315000000	1.667077000000	2.503891000000

ii. Determination of absolute configuration by the comparison with a known compound

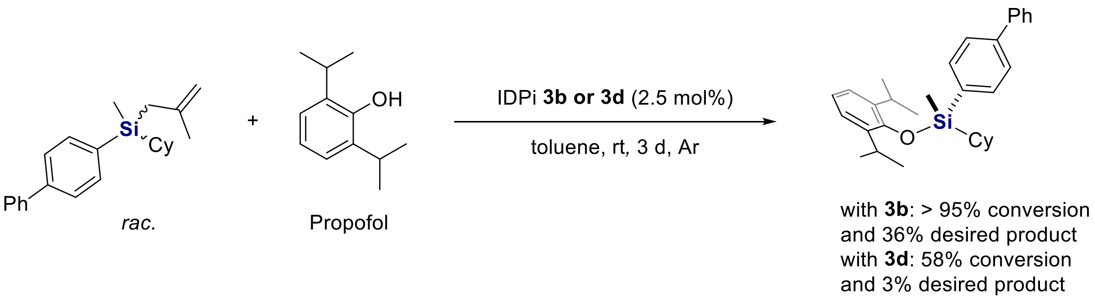

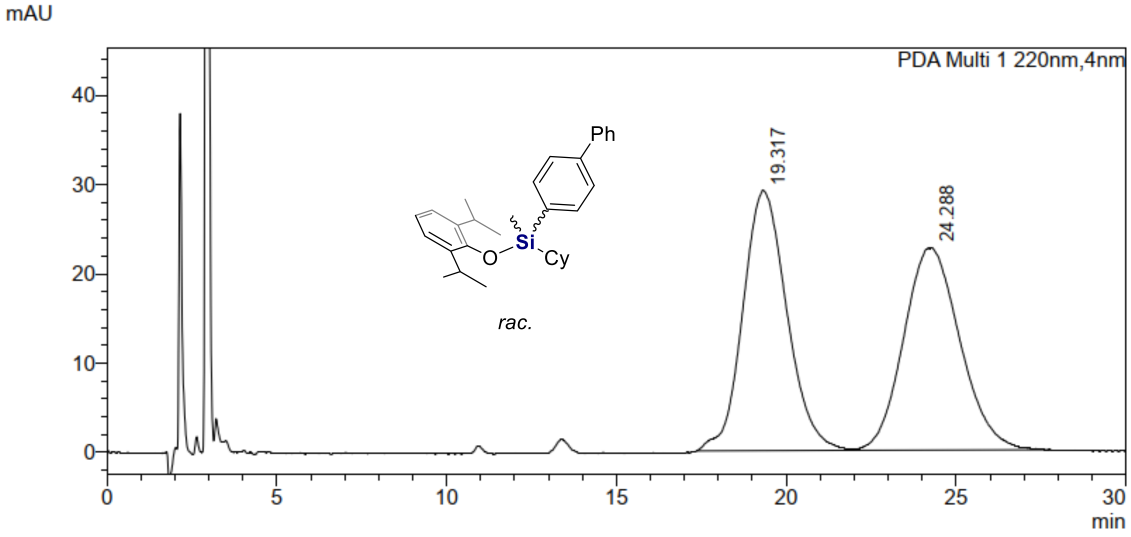

Peak #	t_R/min	% peak area
1	19.3	50.11
2	24.3	49.89
Total		100

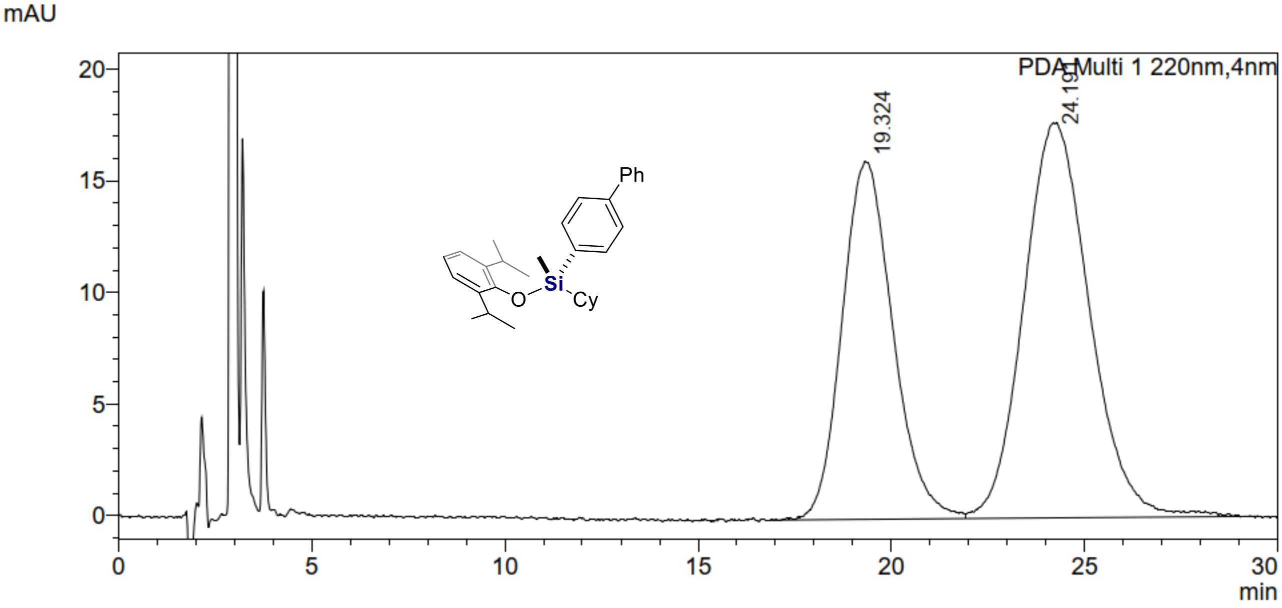

Peak #	t_R/min	% peak area
1	19.3	41.24
2	24.2	58.76
Total		100

After prep LC separation of the above-mentioned product, we obtained the enantiopure product with 99.5:0.5 e.r..

Separation method: 250 mm OJ-H, 20 mm i.D., 5 μm, MeOH/ H_2O = 90:10, flow rate: 20 mL/min, UV 220 nm, 298 K, 15.0 MPa.

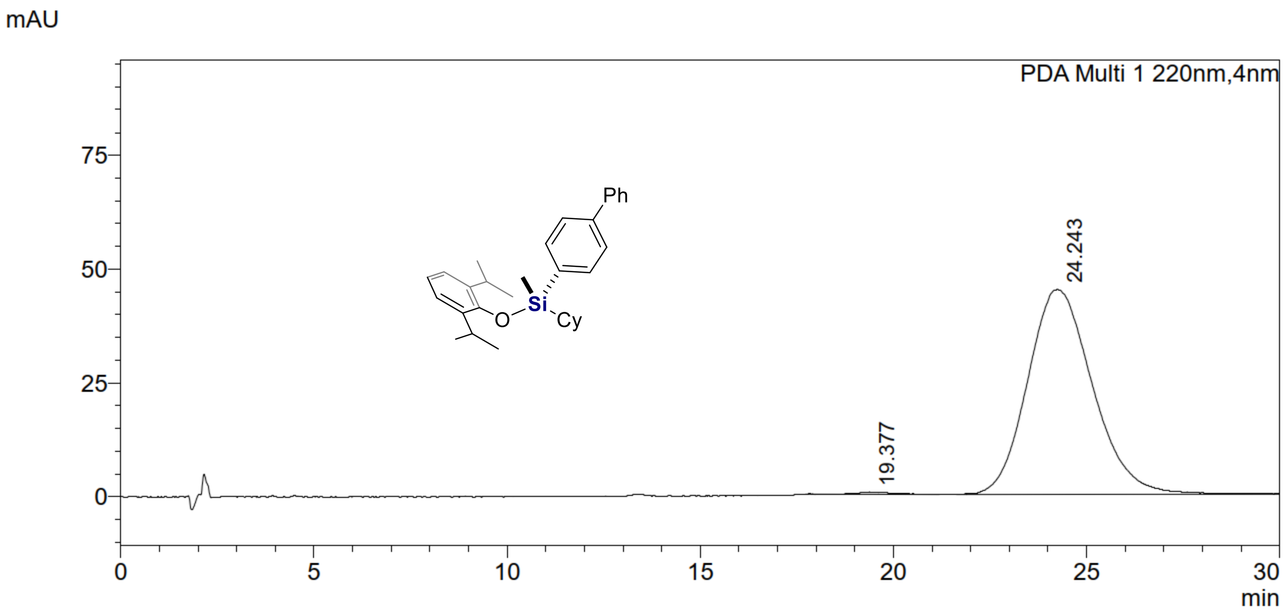

Peak #	t_R/min	% peak area
1	19.4	0.50
2	24.2	99.50
Total		100

We performed the following reduction:

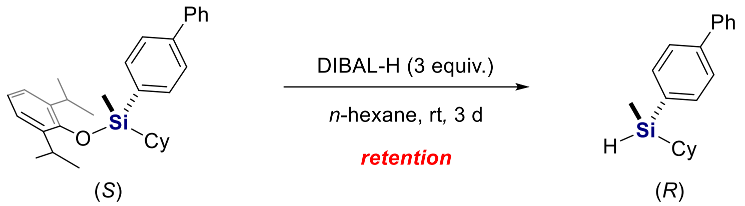

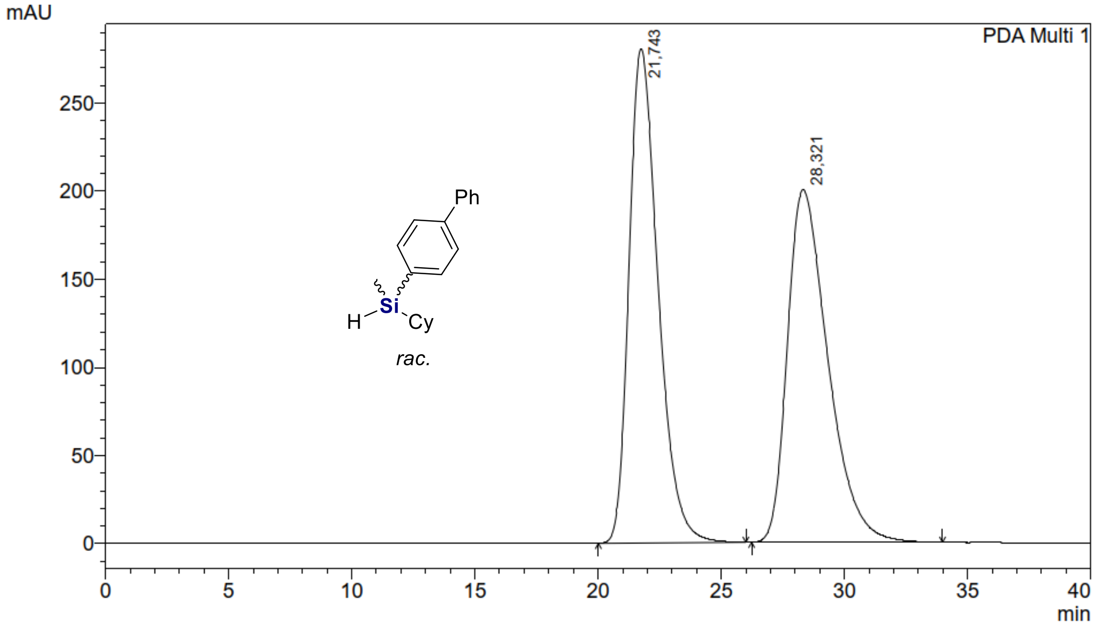

Peak #	t_R/min	% peak area
1	21.7	50.17
2	28.3	49.83
Total		100

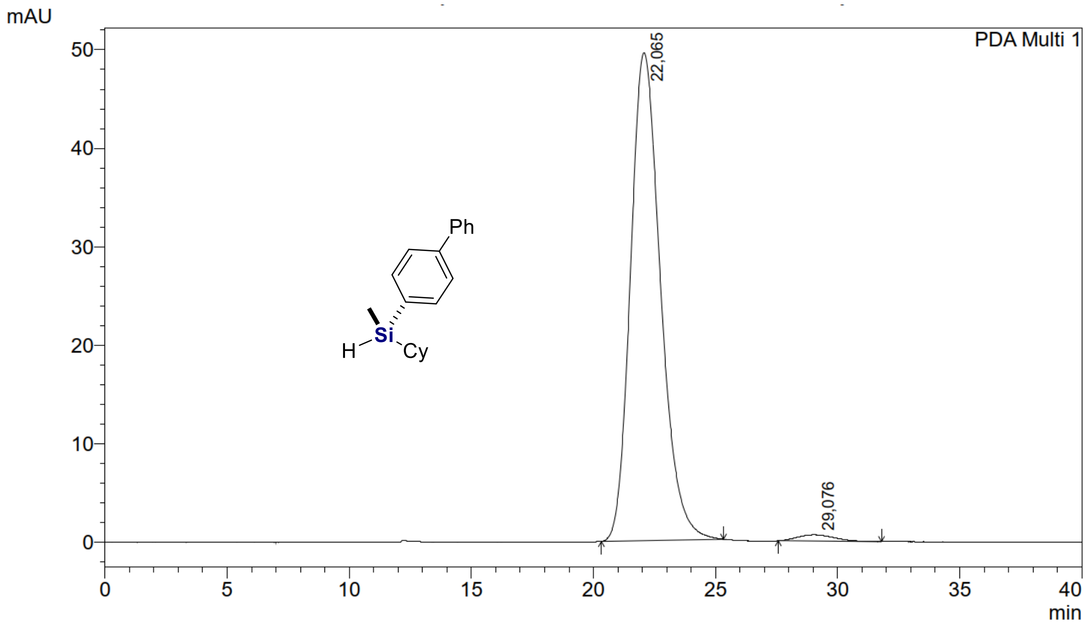

Peak #	t_R/min	% peak area
1	22.1	98.49
2	29.1	1.51
Total		100

Compared to a known procedure,[27] we confirmed that the absolute configuration of monohydrosilane product is *R*. The reduction took place with complete retention of configuration that was reported previously;[28] therefore, we verified that the absolute configuration of the corresponding silyl ether product bearing a phenolic substituent is *S*.

[1,1'-biphenyl]-4-yl(cyclohexyl)(methyl)(2-methylallyl)silane

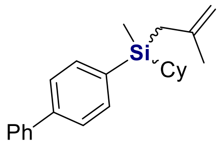

^{1}H NMR (501 MHz, CD_2Cl_2) δ 7.66–7.56 (m, 6H), 7.45 (dd, *J* = 8.4, 7.0 Hz, 2H), 7.38–7.33 (m, 1H), 4.58 (dd, *J* = 2.5, 1.4 Hz, 1H), 4.50 (dq, *J* = 1.8, 0.9 Hz, 1H), 1.87 (d, *J* = 1.1 Hz, 2H), 1.82–1.64 (m, 5H), 1.60 (t, *J* = 1.1 Hz, 3H), 1.32–1.07 (m, 5H), 0.98–0.87 (m, 1H), 0.32 (s, 3H).

^{13}C NMR (126 MHz, CD_2Cl_2) δ 144.0, 141.8, 141.4, 136.5, 135.3, 129.2, 127.8, 127.4, 126.5, 109.3, 28.5, 28.0, 27.9, 27.3, 25.7, 25.6, 24.2, –7.2.

R_f = 0.20 (hexanes).

EI-HRMS (*m/z*): calculated for $C_{23}H_{30}Si_1$ [$M^{+\bullet}$]: 334.2111, found: 334.2110.

(*S*)-[1,1'-biphenyl]-4-yl(cyclohexyl)(2,6-diisopropylphenoxy)(methyl)silane

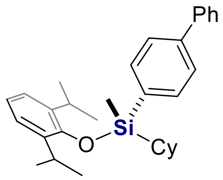

^{1}H NMR (501 MHz, CD_2Cl_2) δ 7.70–7.58 (m, 6H), 7.46 (dd, *J* = 8.4, 7.0 Hz, 2H), 7.40–7.34 (m, 1H), 7.03 (d, *J* = 7.6 Hz, 2H), 6.94 (dd, *J* = 8.2, 7.0 Hz, 1H), 3.24 (p, *J* = 6.8 Hz, 2H), 1.90 (ddt, *J* = 12.4, 5.8, 3.0 Hz, 2H), 1.83–1.68 (m, 3H), 1.28 (dtd, *J* = 21.0, 10.3, 6.1 Hz, 6H), 1.10 (dd, *J* = 13.2, 6.8 Hz, 12H), 0.46 (s, 3H).

^{13}C NMR (126 MHz, CD_2Cl_2) δ 150.2, 142.4, 141.3, 139.5, 135.2, 134.9, 129.2, 127.9, 127.4, 126.5, 123.8, 122.4, 28.4, 28.0, 27.4, 27.4, 27.3, 27.3, 23.6, 23.5, –5.2.

R_f = 0.28 (Ethyl acetate/hexanes = 1:49).

ESI-HRMS (*m/z*): calculated for $C_{31}H_{40}Na_1O_1Si_1$ ([M+Na]$^+$): 479.2741, found: 479.2742.

(*R*)-[1,1'-biphenyl]-4-yl(cyclohexyl)(methyl)silane

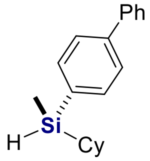

^{1}H NMR (501 MHz, CD_2Cl_2) δ 7.64–7.57 (m, 6H), 7.45 (dd, J = 8.4, 6.9 Hz, 2H), 7.36 (d, J = 7.4 Hz, 1H), 4.21 (dd, J = 3.8, 2.7 Hz, 1H), 1.81–1.64 (m, 5H), 1.33–1.13 (m, 6H), 0.34 (d, J = 3.8 Hz, 3H).

^{13}C NMR (126 MHz, CD_2Cl_2) δ 142.2, 141.4, 135.6, 135.1, 129.2, 127.8, 127.4, 126.7, 28.6, 28.3, 28.3, 27.2, 24.7, –7.6.

R_f = 0.26 (hexanes).

EI-HRMS (m/z): calculated for $C_{19}H_{24}Si_1$ [$M^{+\bullet}$]: 280.1642, found: 280.1644.

9. References

1. Zhou, H.; Han, J. T.; Nöthling, N.; Lindner, M. M.; Jenniches, J.; Kühn, C.; Tsuji, N.; Zhang, L.; List, B., Organocatalytic Asymmetric Synthesis of Si-Stereogenic Silyl Ethers. *J. Am. Chem. Soc.* **2022**, *144*, 10156–10161.

2. Lee, S.; Bae, H. Y.; List, B., Can a ketone be more reactive than an aldehyde? Catalytic asymmetric synthesis of substituted tetrahydrofurans. *Angew. Chem. Int. Ed.* **2018**, *57*, 12162–12166.

3. Kaib, P. S.; Schreyer, L.; Lee, S.; Properzi, R.; List, B., Extremely active organocatalysts enable a highly enantioselective addition of allyltrimethylsilane to aldehydes. *Angew. Chem. Int. Ed.* **2016**, *55*, 13200–13203.

4. Fulmer, G. R.; Miller, A. J.; Sherden, N. H.; Gottlieb, H. E.; Nudelman, A.; Stoltz, B. M.; Bercaw, J. E.; Goldberg, K. I., NMR Chemical Shifts of Trace Impurities: Common Laboratory Solvents, Organics, and Gases in Deuterated Solvents Relevant to the Organometallic Chemist. *Organometallics* **2010**, *29*, 2176–2179.

5. Hoffman, R. E., Standardization of Chemical Shifts of TMS and Solvent Signals in NMR Solvents. *Magn. Reson. Chem.* **2006**, *44*, 606–616.

6. Rendler, S.; Oestreich, M., Conclusive Evidence for an S_N2-Si Mechanism in the $B(C_6F_5)_3$-Catalyzed Hydrosilylation of Carbonyl Compounds: Implications for the Related Hydrogenation. *Angew. Chem. Int. Ed.* **2008**, *47*, 5997–6000.

7. Schwengers, S. A.; De, C. K.; Grossmann, O.; Grimm, J. A.; Sadlowski, N. R.; Gerosa, G. G.; List, B., Unified Approach to Imidodiphosphate-Type Brønsted Acids with Tunable Confinement and Acidity. *J. Am. Chem. Soc.* **2021**, *143*, 14835–14844.

8. Grimme, S.; Bannwarth, C.; Shushkov, P., A robust and accurate tight-binding quantum chemical method for structures, vibrational frequencies, and noncovalent interactions of large molecular systems parametrized for all spd-block elements (Z= 1–86). *J. Chem. Theory Comput.* **2017**, *13*, 1989–2009.

9. Neese, F., Software update: The ORCA program system—Version 5.0. WIREs Comput Mol Sci. 2022; 12:e1606.

10. Te Velde, G. t.; Bickelhaupt, F. M.; Baerends, E. J.; Fonseca Guerra, C.; van Gisbergen, S. J.; Snijders, J. G.; Ziegler, T., Chemistry with ADF. *J. Comput. Chem.* **2001**, *22*, 931–967.

11. Manual, A., ADF Program System Release 2014, **1993**.

12. Swart, M.; Bickelhaupt, F. M., QUILD: QUantum-regions interconnected by local descriptions. *J. Comput. Chem.* **2008**, *29*, 724–734.

13. Perdew, J. P.; Burke, K.; Ernzerhof, M., Generalized gradient approximation made simple. *Phys. Rev. Lett.* **1996**, *77*, 3865–3868.

14. Grimme, S.; Antony, J.; Ehrlich, S; Krieg, H., A consistent and accurate ab initio parametrization of density functional dispersion correction (DFT-D) for the 94 elements H–Pu. *J. Chem. Phys.* **2010**, *132*, 154104.

15. Grimme, S.; Ehrlich, S.; Goerigk, L., Effect of the damping function in dispersion corrected density functional theory. *J. Comput. Chem.* **2011**, *32*, 1456–1465.

16. Yepes, D.; Neese, F.; List, B.; Bistoni, G., Unveiling the delicate balance of steric and dispersion interactions in organocatalysis using high-level computational methods. *J. Am. Chem. Soc.* **2020** *142*, 3613–3625.

17. Xtb, Version 6.3; University Bonn: 2020; please refer to xtb@thch.uni-bonn.de.

18. Neese, F., The ORCA program system. *WIREs Comput. Mol. Sci.* **2012**, *2*, 73–78.

19. Becke, A. D., Density-functional thermochemistry. III. The role of exact exchange. *J. Chem. Phys.* **1993**, *98*, 5648–5652.

20. Lee, C.; Yang, W.; Parr, R. G., Development of the Colle-Salvetti correlation-energy formula into a functional of the electron density. *Phys. Rev. B* **1988**, *37*, 785–789.

21. Weigend, F., Accurate Coulomb-fitting basis sets for H to Rn. *Phys. Chem. Chem. Phys.* **2006**, *8*, 1057–1065.

22. Weigend, F; Ahlrichs, R., Balanced basis sets of split valence, triple zeta valence and quadruple zeta valence quality for H to Rn: Design and assessment of accuracy. *Phys. Chem. Chem. Phys.* **2005**, *7*, 3297–3305.

23. Valeev, E. F., Libint: A library for the evaluation of molecular integrals of many-body operators over Gaussian functions, *http://libint.valeyev.net/*

24. Barone, V.; Cossi, M., Quantum calculation of molecular energies and energy gradients in solution by a conductor solvent model. *J. Phys. Chem. A* **1998**, *102*, 1995–2001.

25. Lu, T.; Chen, F., Multiwfn: A multifunctional wavefunction analyzer. *J. Comput. Chem.* **2012**, *33*, 580–592.

26. Frisch, M.; Trucks, G.; Schlegel, H.; Scuseria, G.; Robb, M.; Cheeseman, J.; Scalmani, G.; Barone, V.; Mennucci, B.; Petersson, G., Gaussian 09 (Gaussian, Inc., Wallingford, CT, 2009).

27. Wang, X.; Feng, C.; Kubota, K.; Ito, H., Stereospecific Synthesis of Silicon-Stereogenic Optically Active Silylboranes and their Application to Synthesis of Chiral Organosilanes. ChemRxiv. Cambridge: Cambridge Open Engage; 2022; This content is a preprint and has not been peer-reviewed.

28. Sommer, L.; McLick, J.; Golino, C., SNi-Si Mechanism. Reductive Displacement of Good Leaving Groups with Retention of Configuration by Diisobutylaluminum Hydride. Stereochemical and Mechanistic Crossover with the Etherate Complex of Diisobutylaluminum Hydride. *J. Am. Chem. Soc.* **1972**, *94*, 669–670.

10. Copies of NMR spectra

hexyl(methyl)(2-methylallyl)(phenyl)silane 1a:

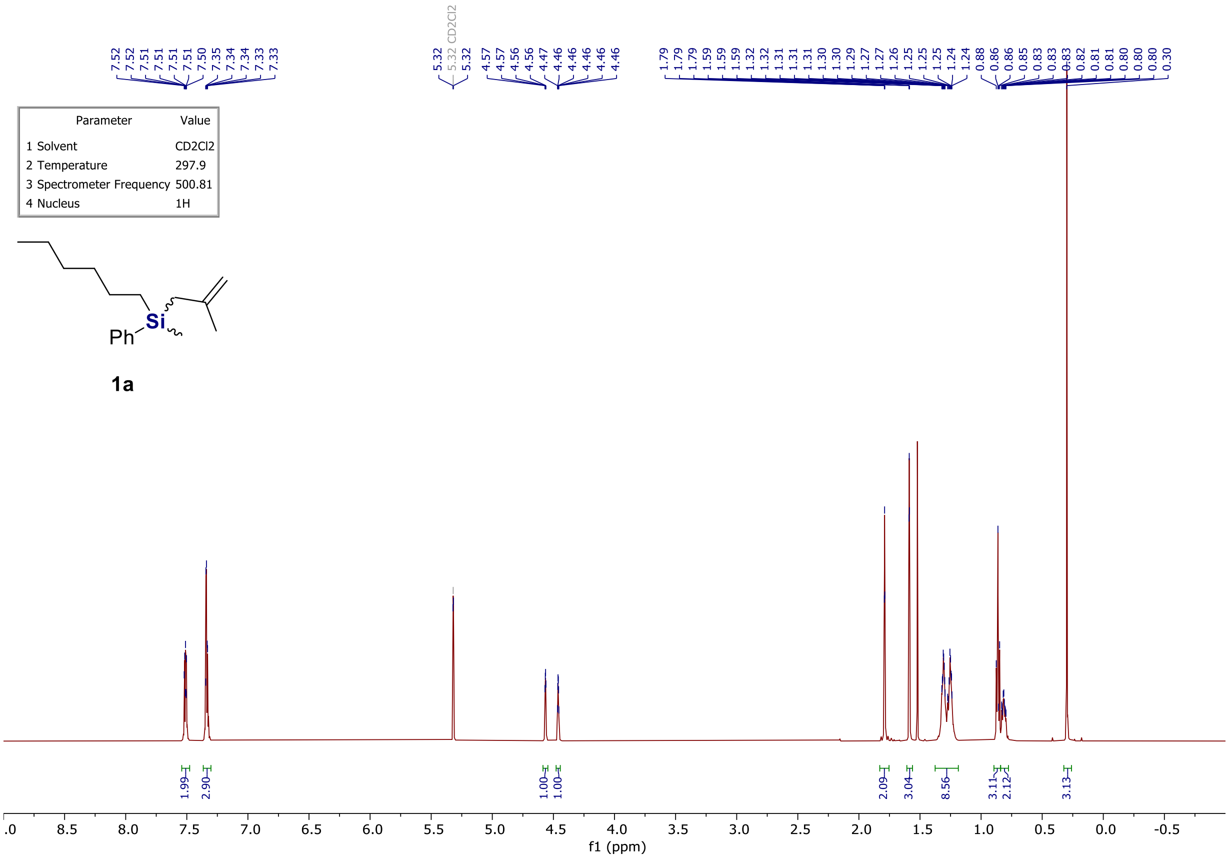

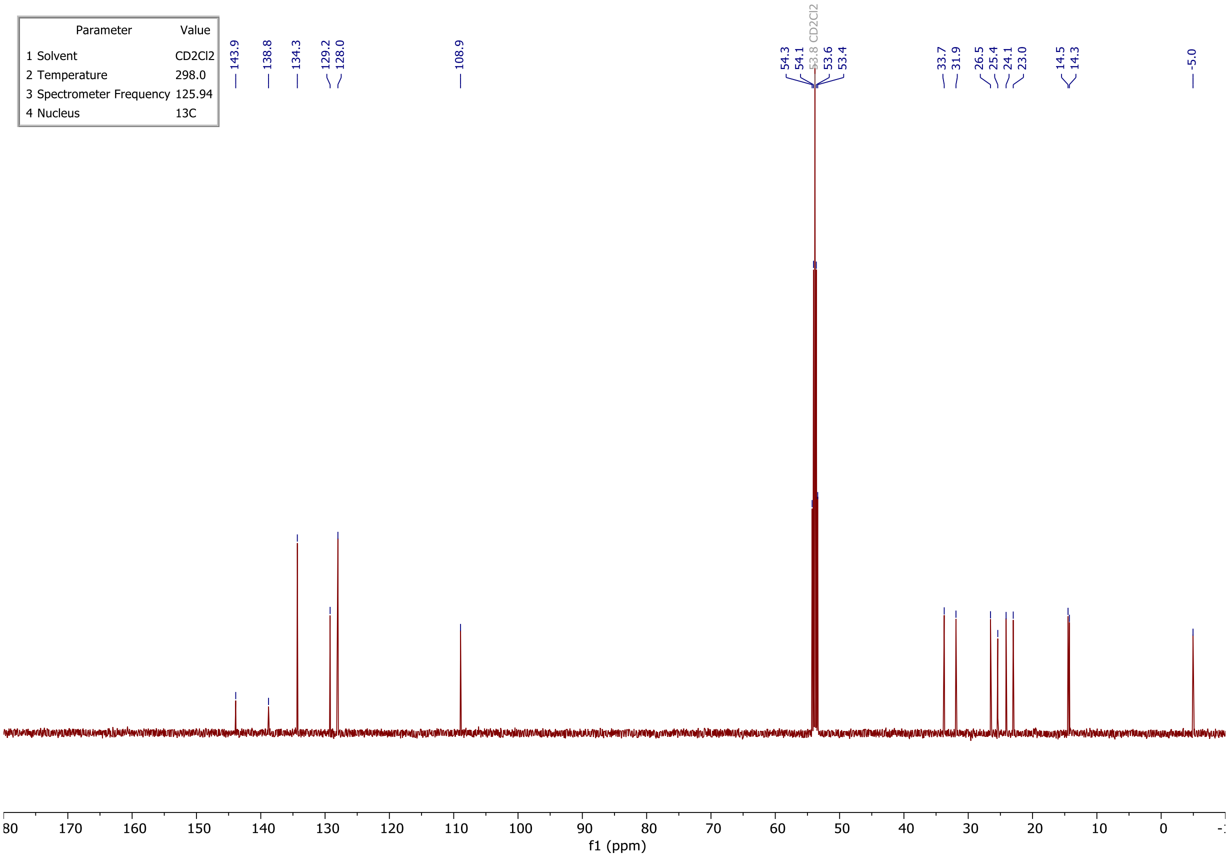

ethyl(methyl)(2-methylallyl)(phenyl)silane 1b

Parameter	Value
1 Solvent	CD2Cl2
2 Temperature	297.9
3 Spectrometer Frequency	500.81
4 Nucleus	1H

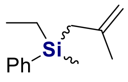

1b

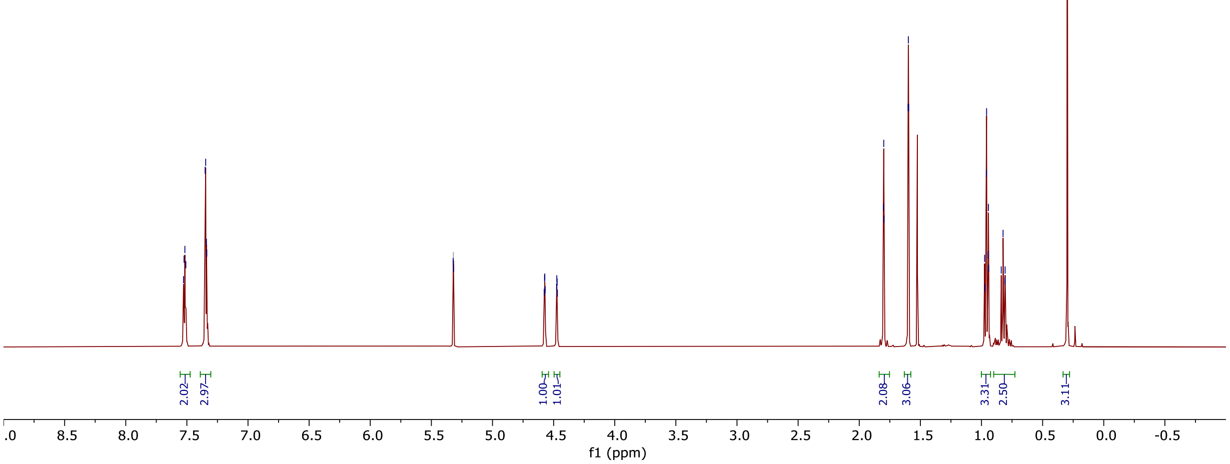

Parameter	Value
1 Solvent	CD2Cl2
2 Temperature	298.0
3 Spectrometer Frequency	125.94
4 Nucleus	13C

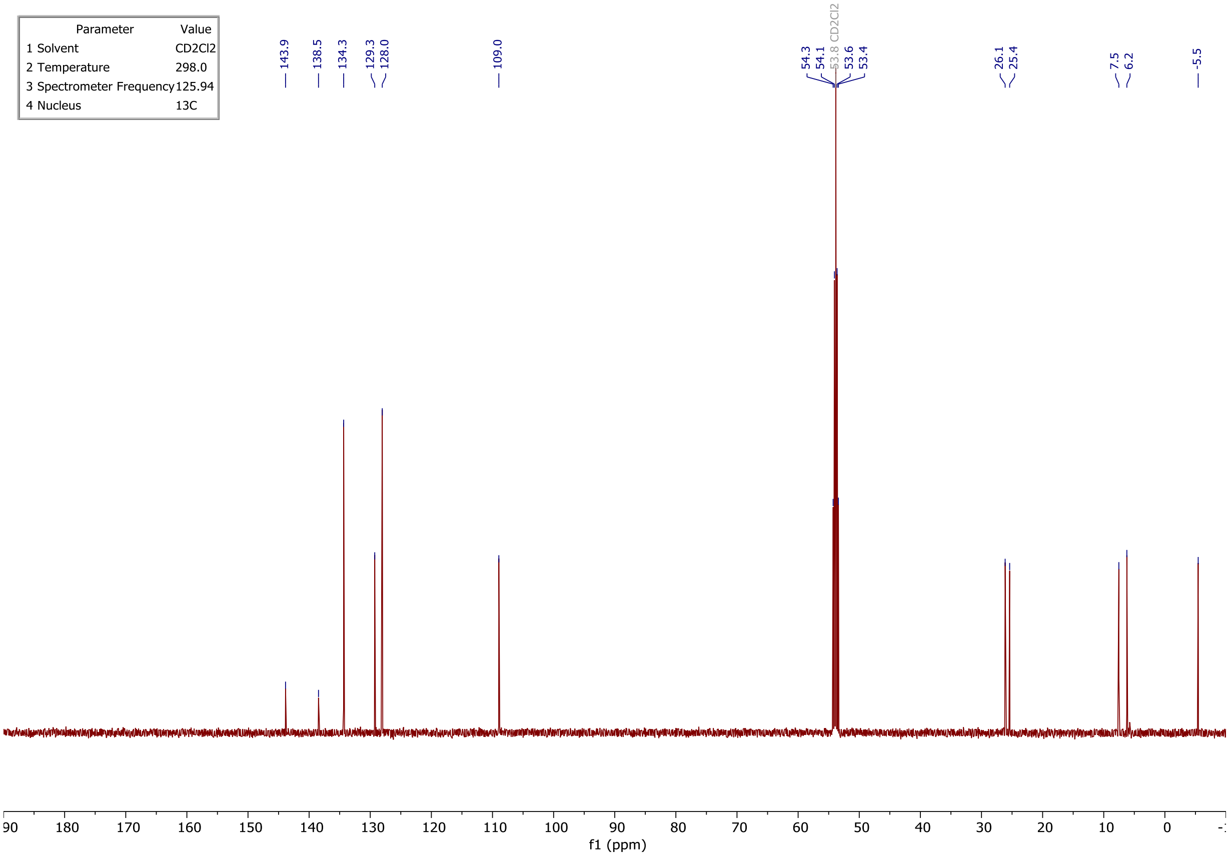

methyl(2-methylallyl)(phenyl)(propyl)silane 1c

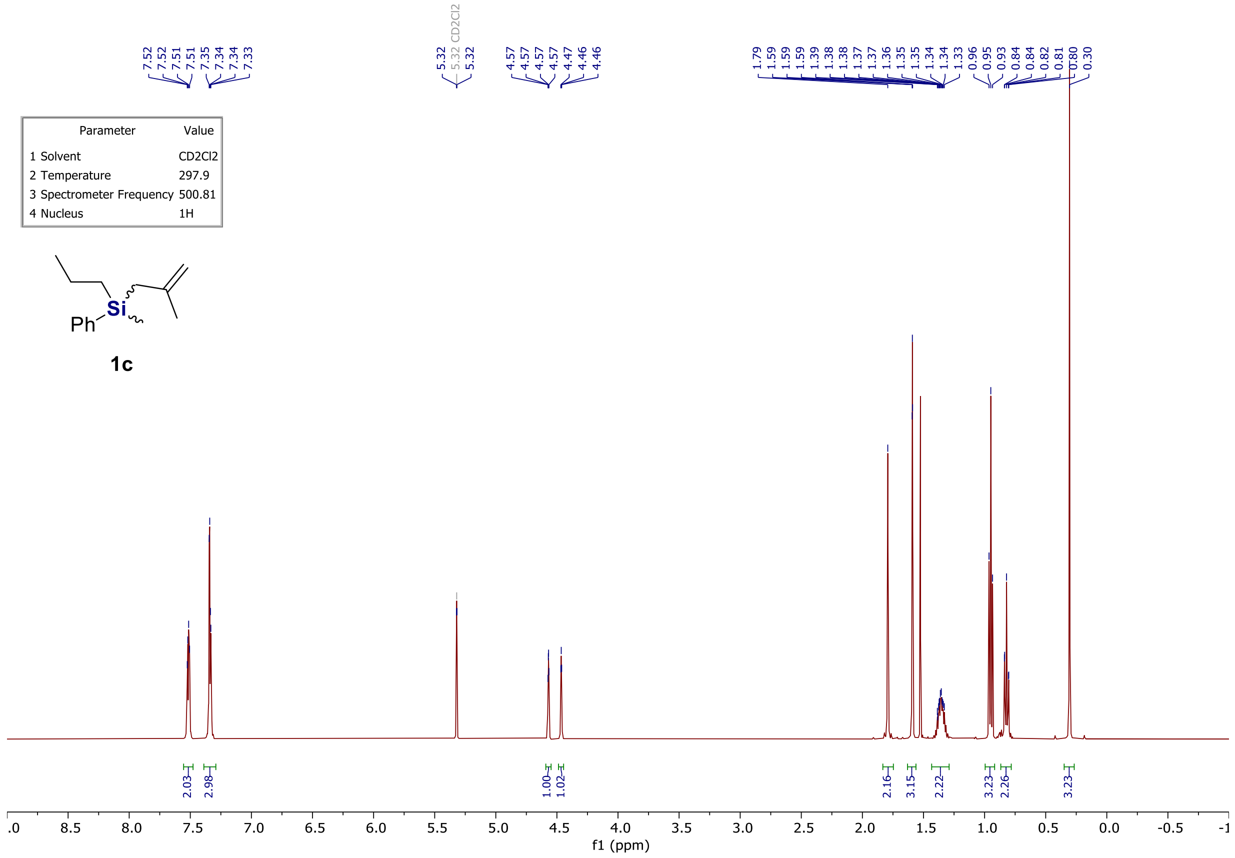

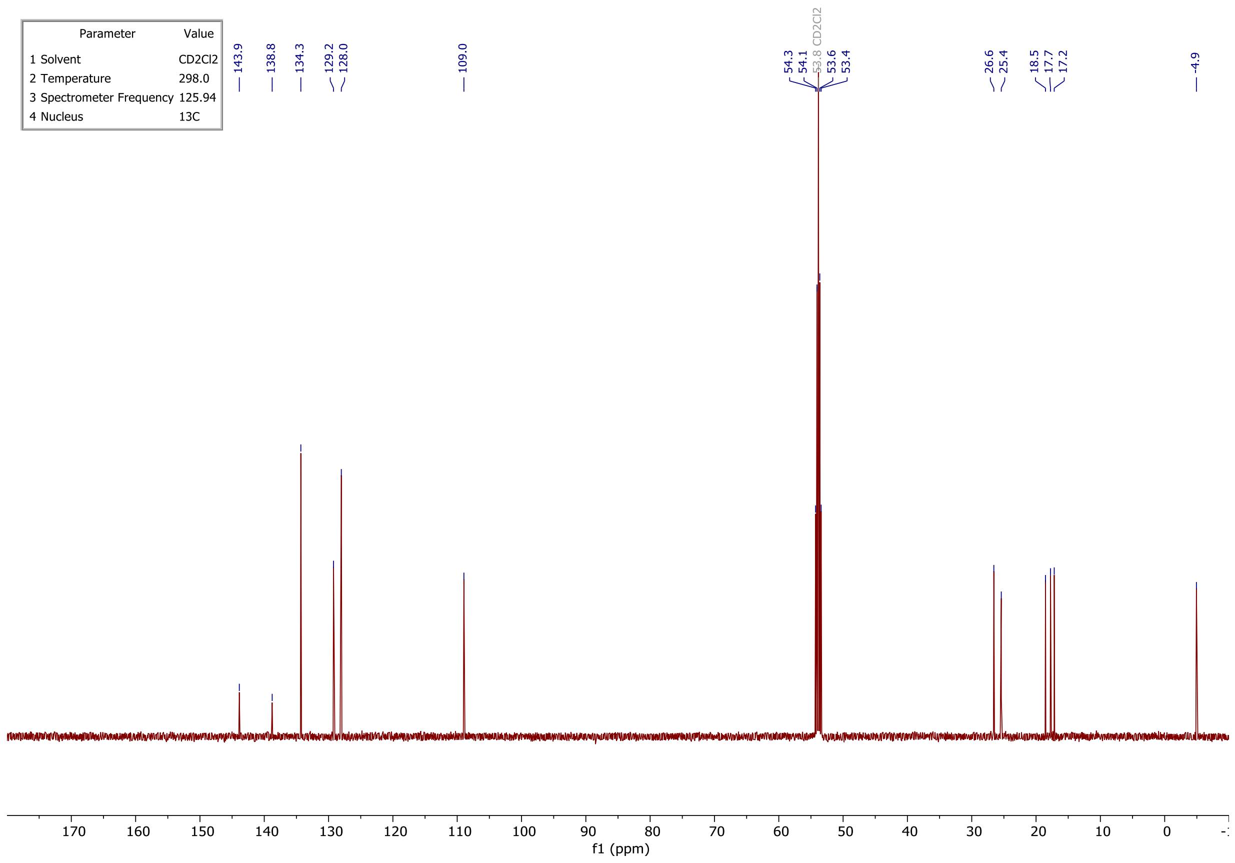

butyl(methyl)(2-methylallyl)(phenyl)silane 1d

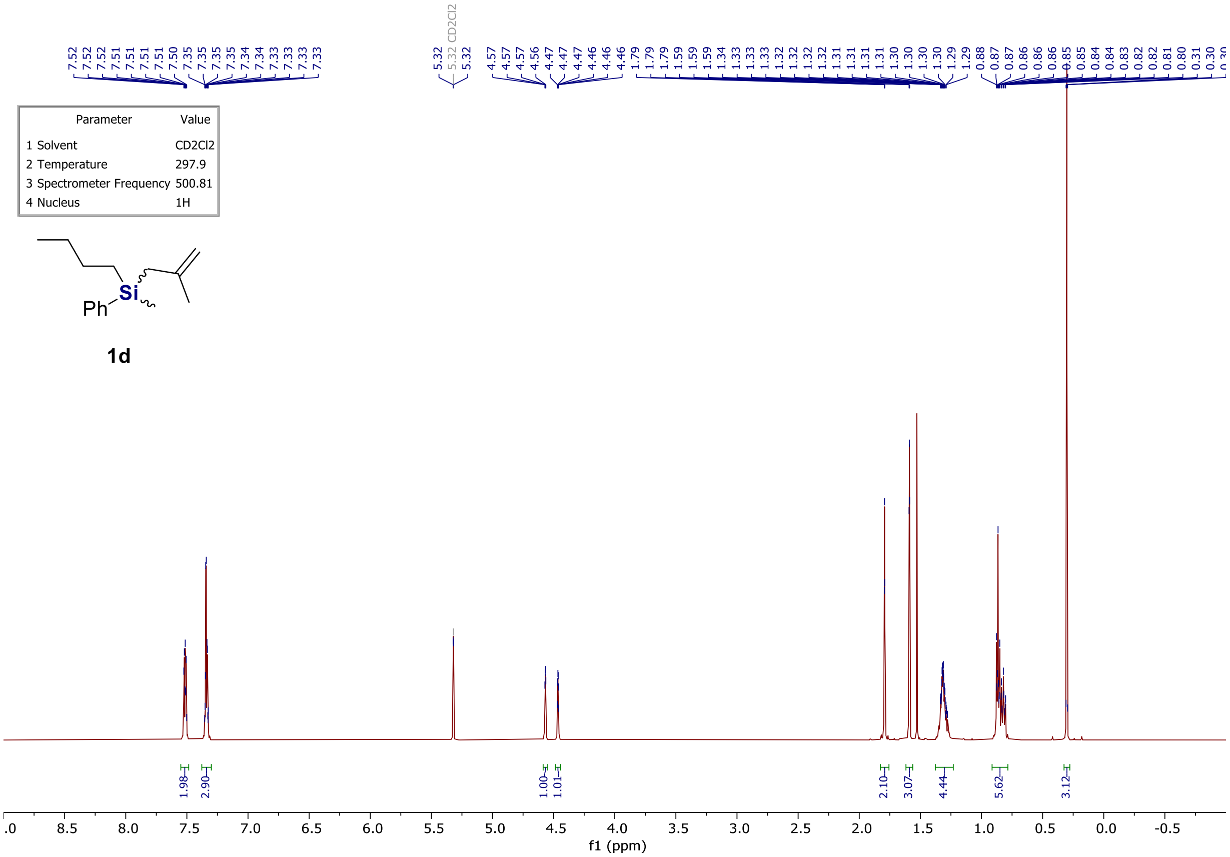

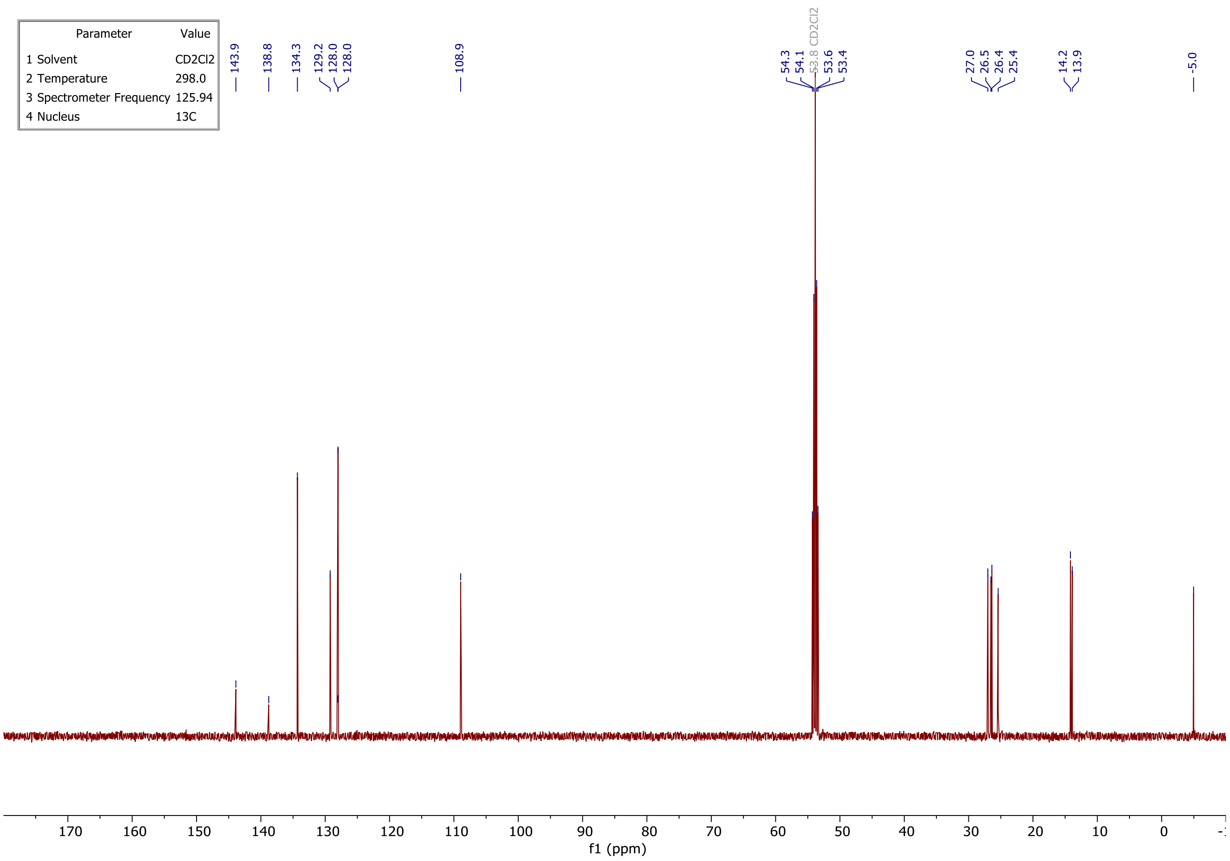

methyl(2-methylallyl)(pentyl)(phenyl)silane 1e

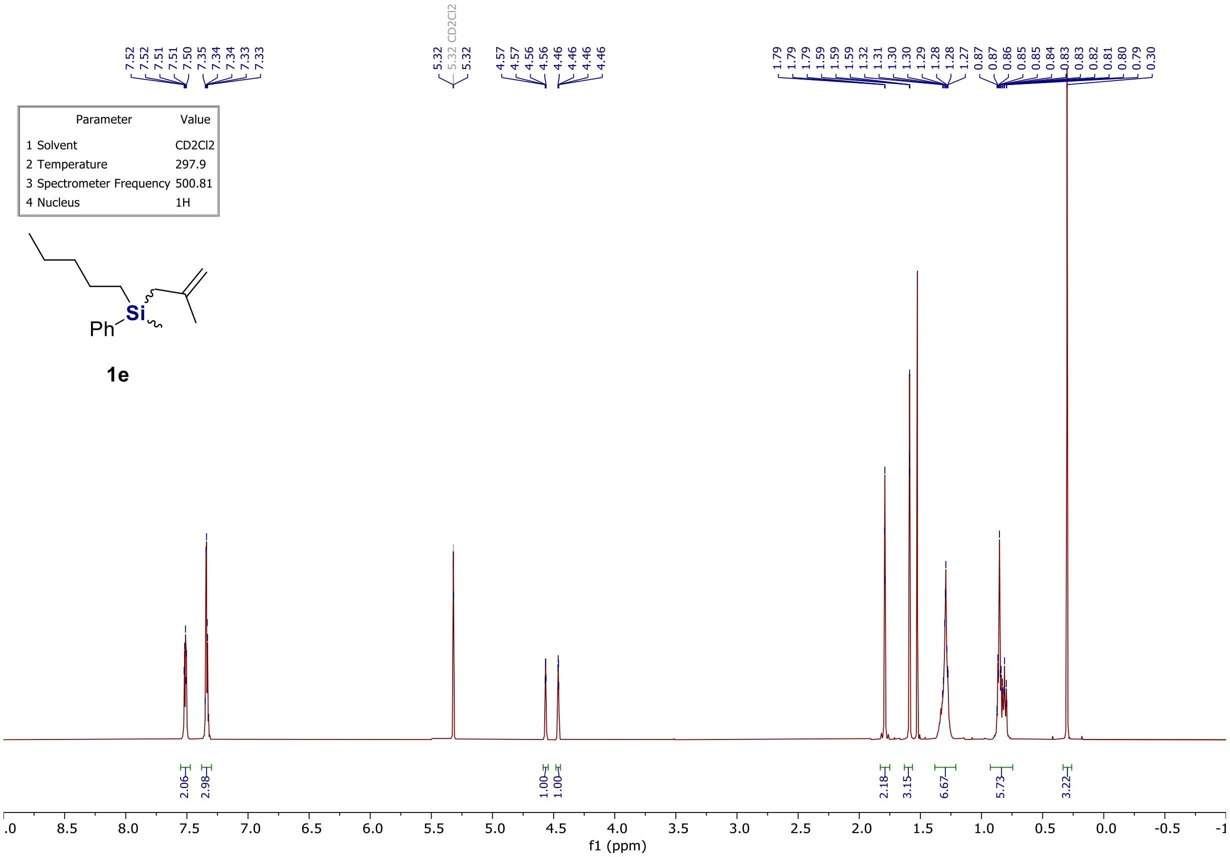

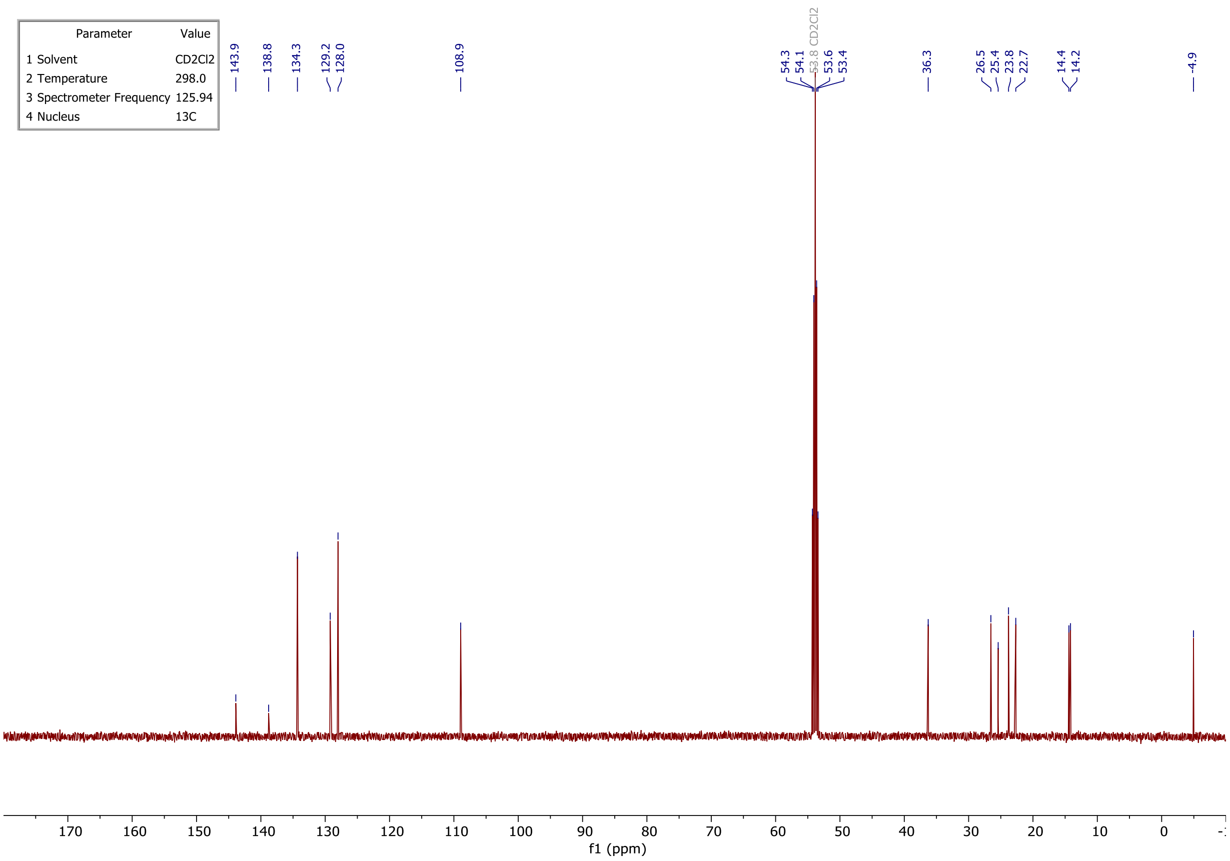

heptyl(methyl)(2-methylallyl)(phenyl)silane 1f

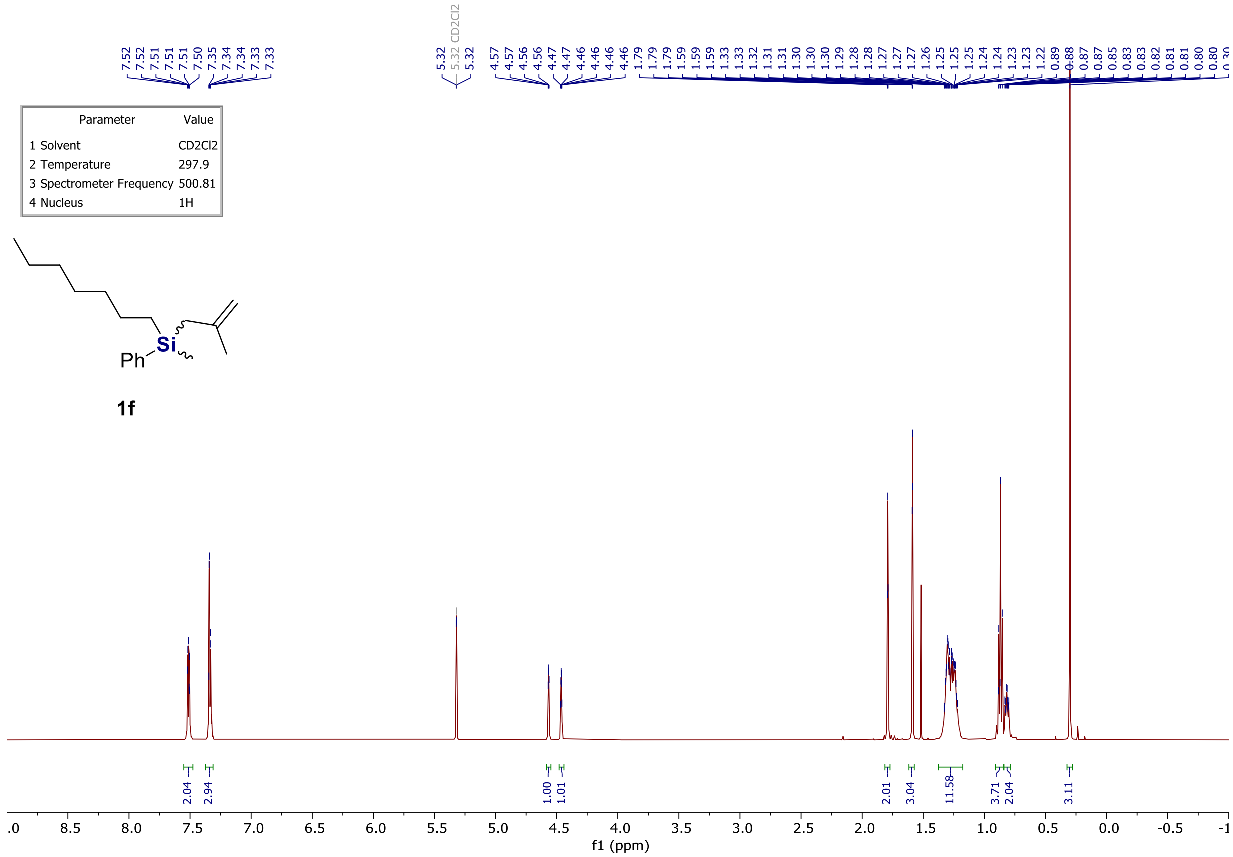

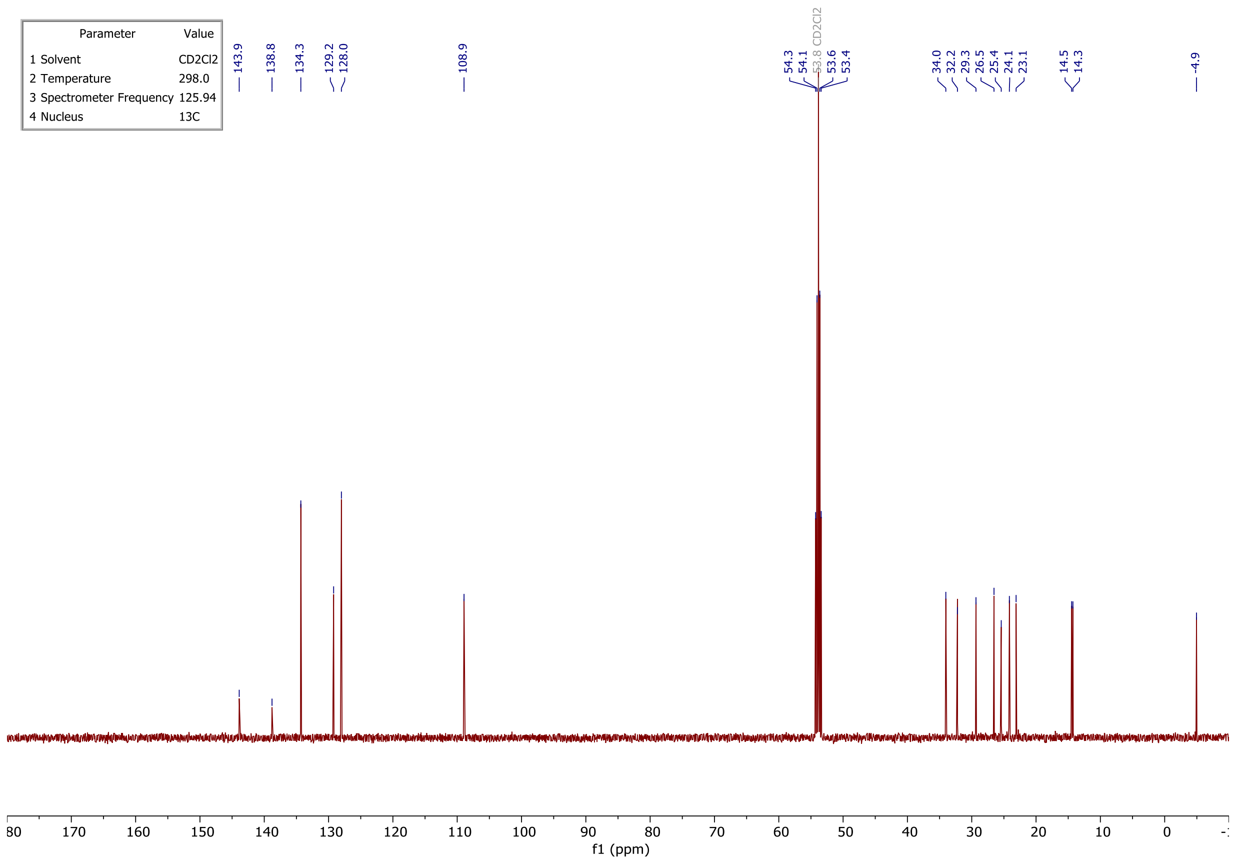

isobutyl(methyl)(2-methylallyl)(phenyl)silane 1g

Parameter	Value
1 Solvent	CD2Cl2
2 Temperature	297.9
3 Spectrometer Frequency	500.81
4 Nucleus	1H

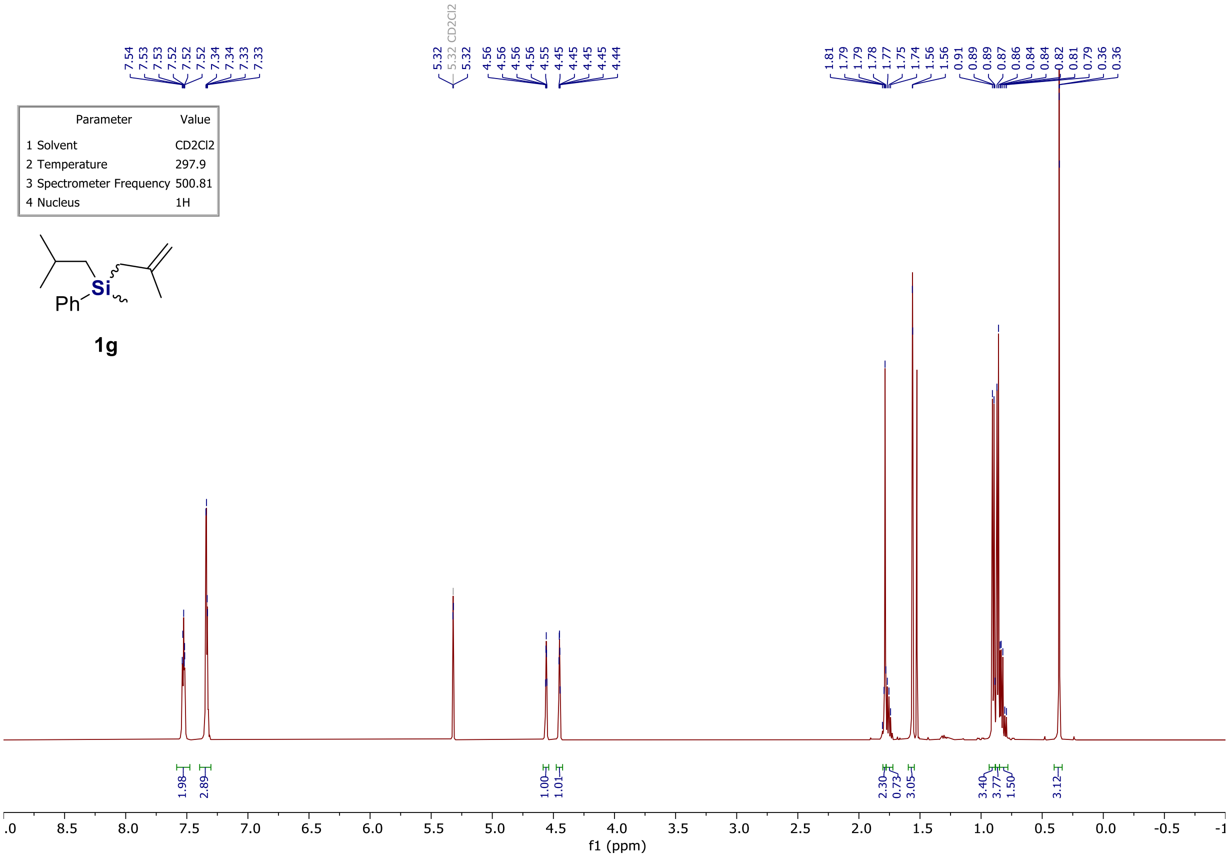

Parameter	Value
1 Solvent	CD2Cl2
2 Temperature	298.0
3 Spectrometer Frequency	125.94
4 Nucleus	13C

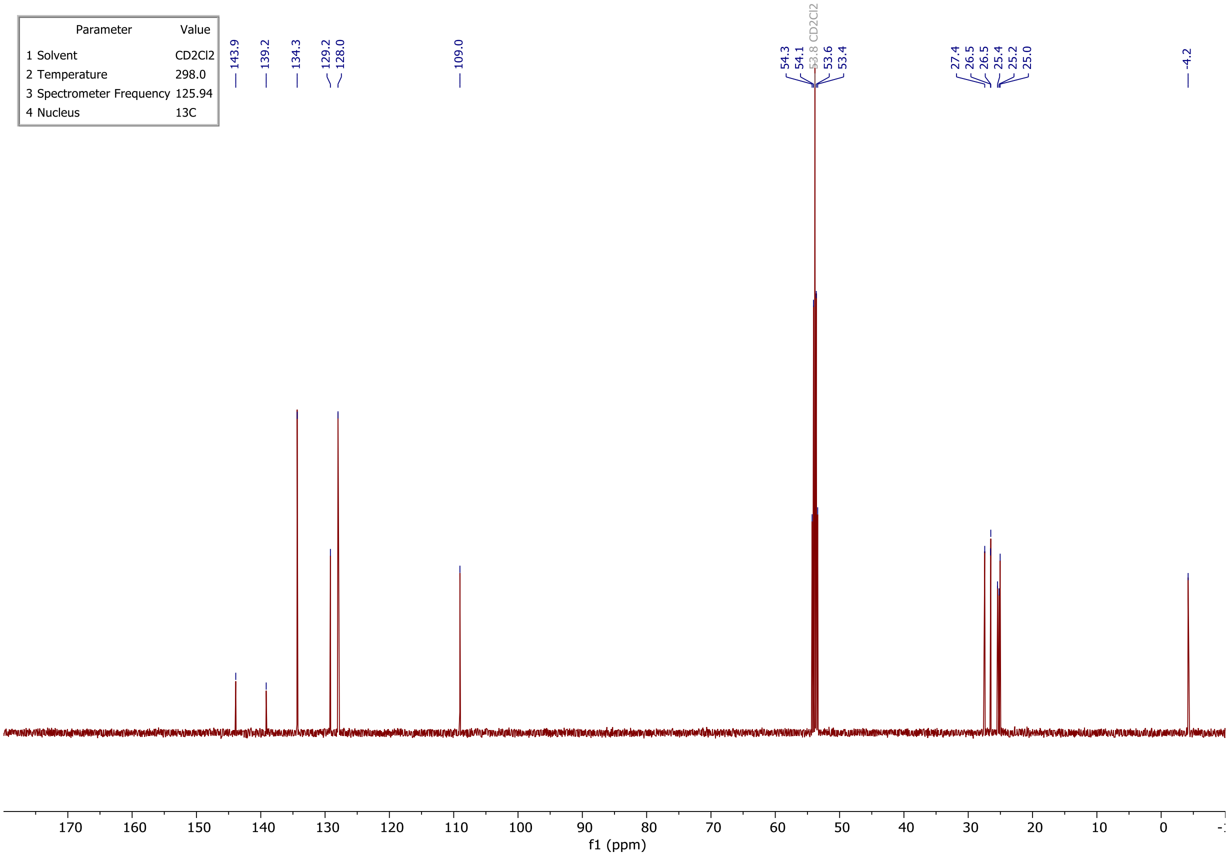

hexyl(methyl)(2-methylallyl)(p-tolyl)silane 1h

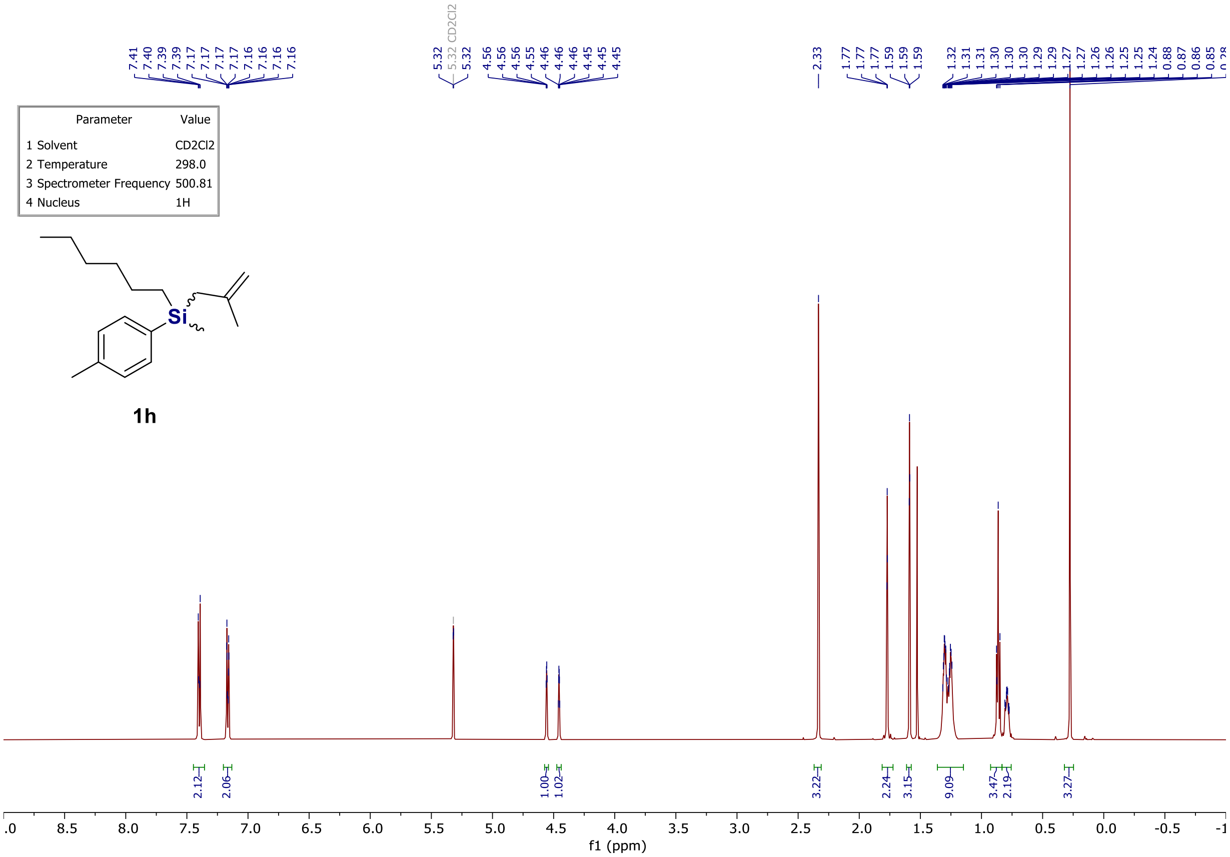

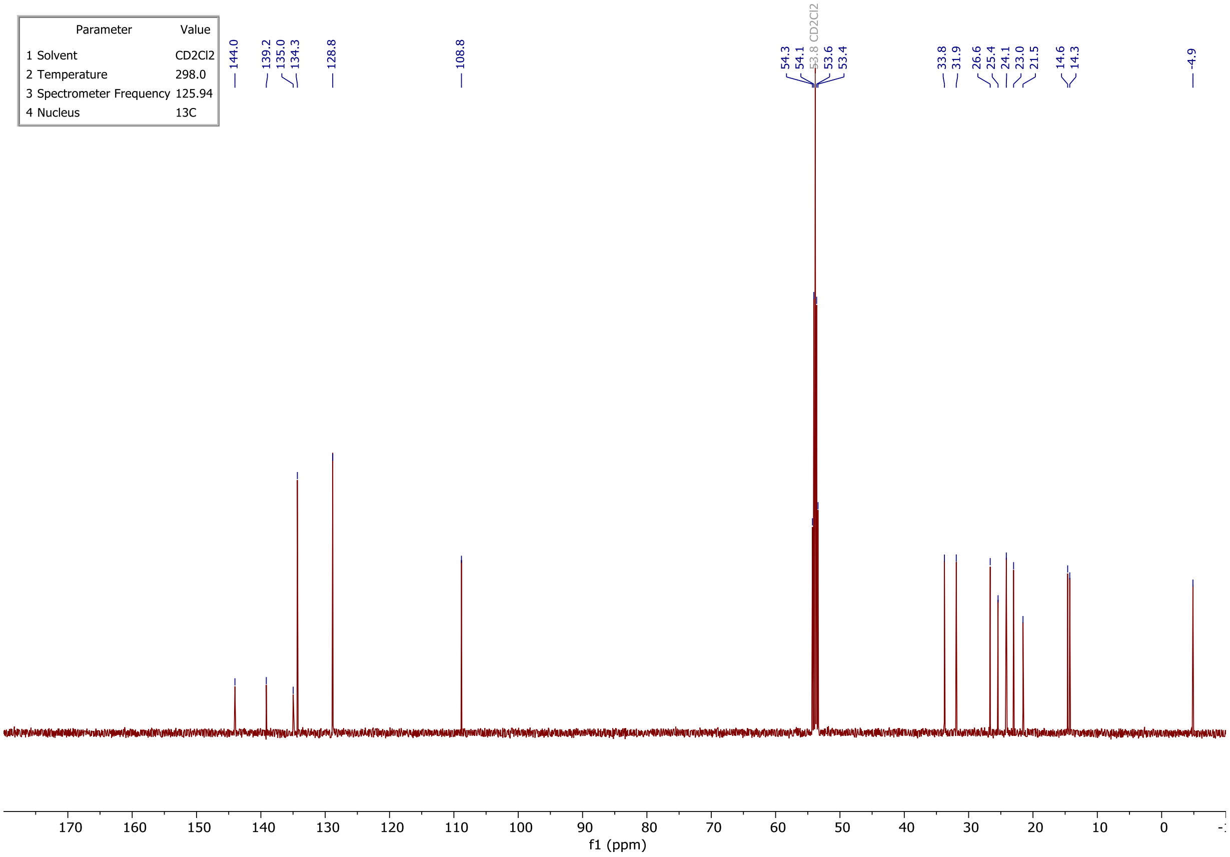

hexyl(4-methoxyphenyl)(methyl)(2-methylallyl)silane 1i

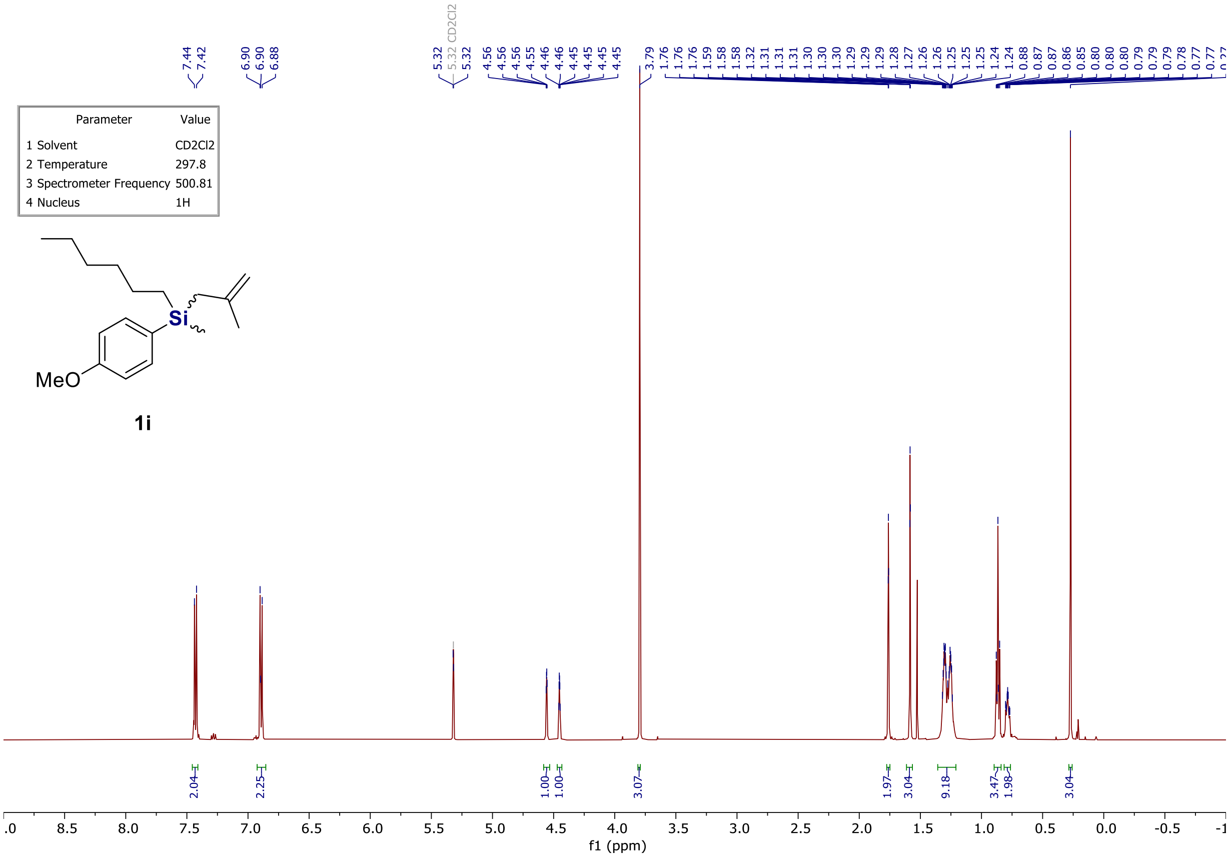

Parameter	Value
1 Solvent	CD2Cl2
2 Temperature	297.8
3 Spectrometer Frequency	500.81
4 Nucleus	1H

1i

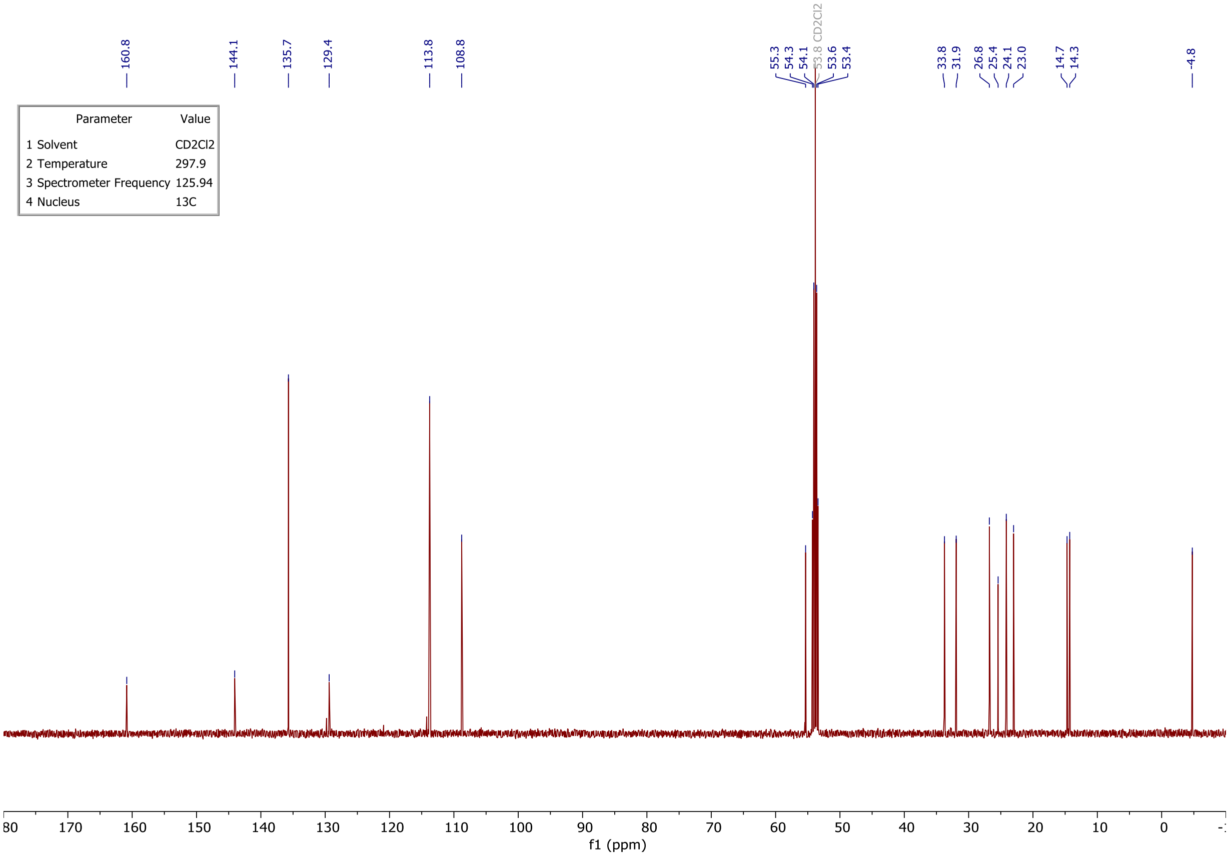

Parameter	Value
1 Solvent	CD2Cl2
2 Temperature	297.9
3 Spectrometer Frequency	125.94
4 Nucleus	13C

(4-fluorophenyl)(hexyl)(methyl)(2-methylallyl)silane 1j

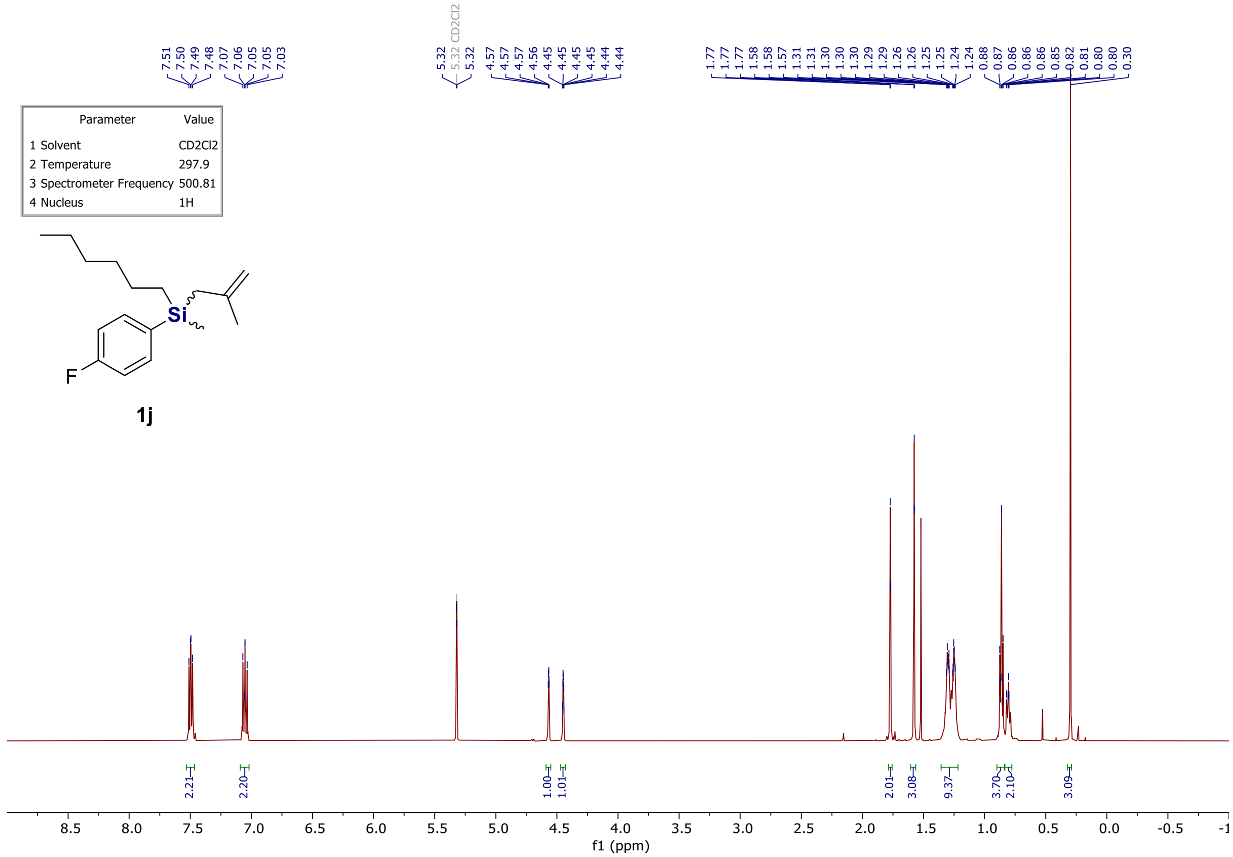

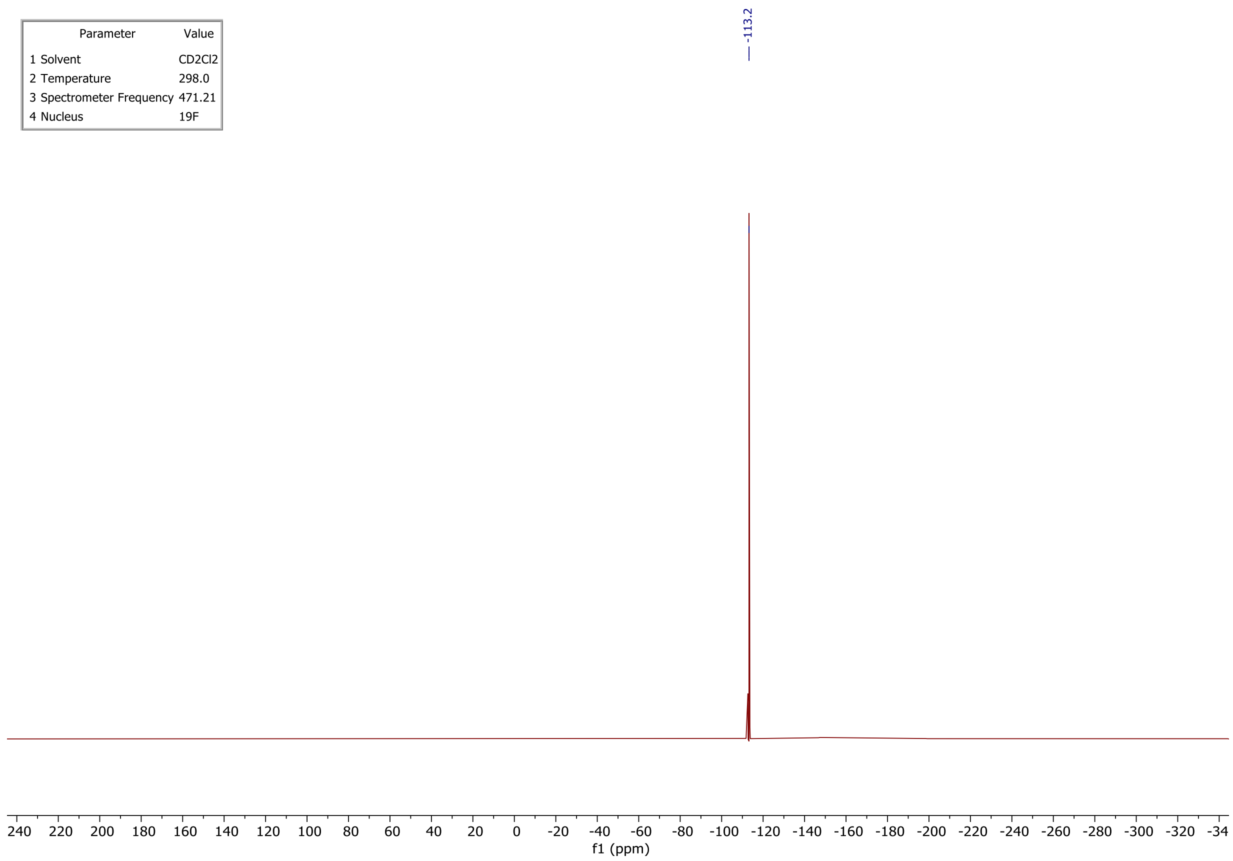

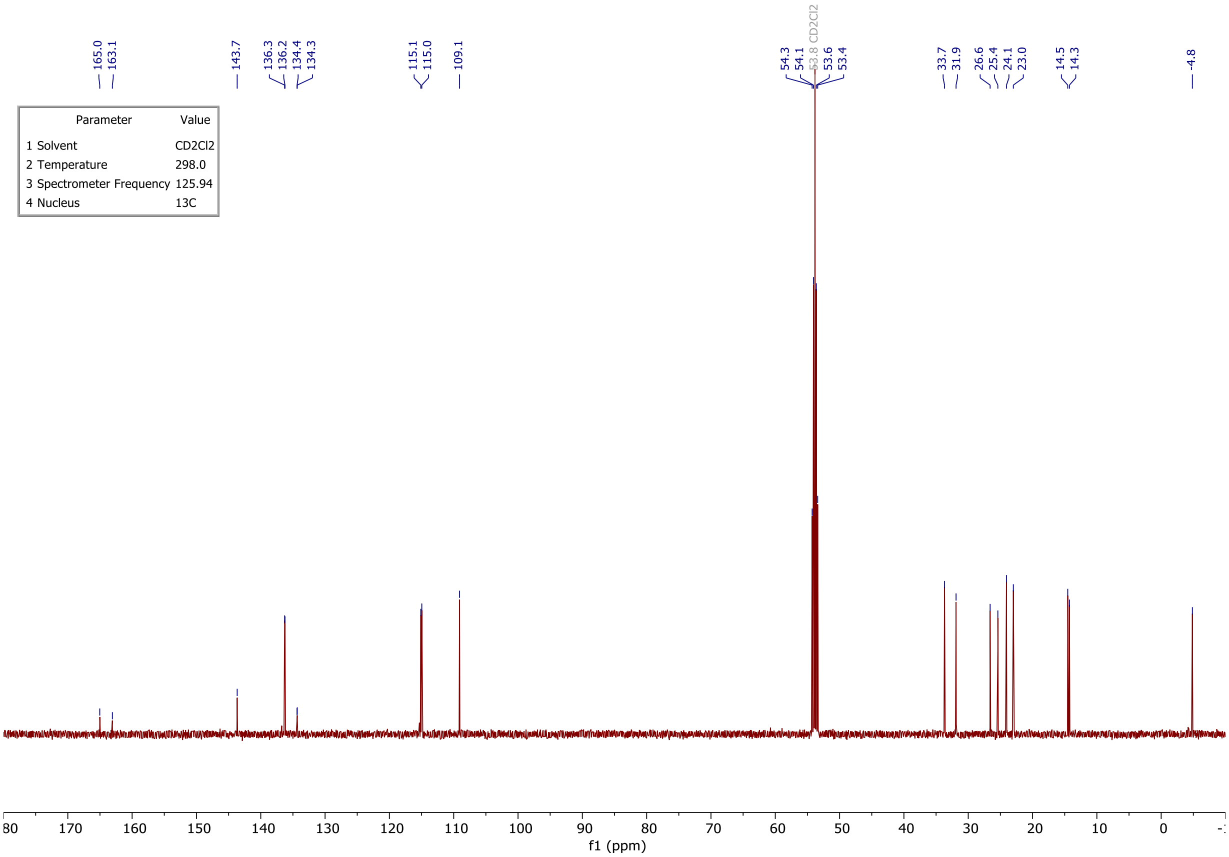

hexyl(methyl)(2-methylallyl)(4-(trifluoromethyl)phenyl)silane 1k

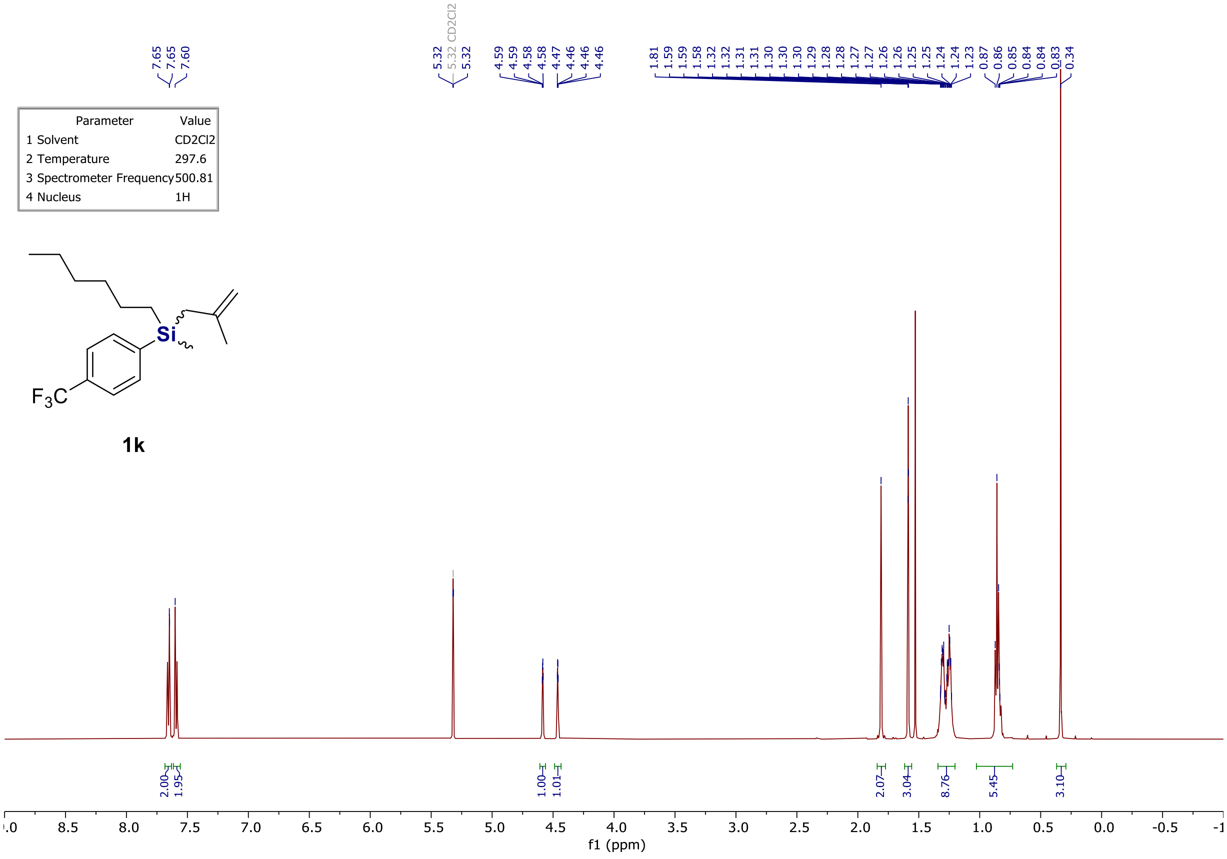

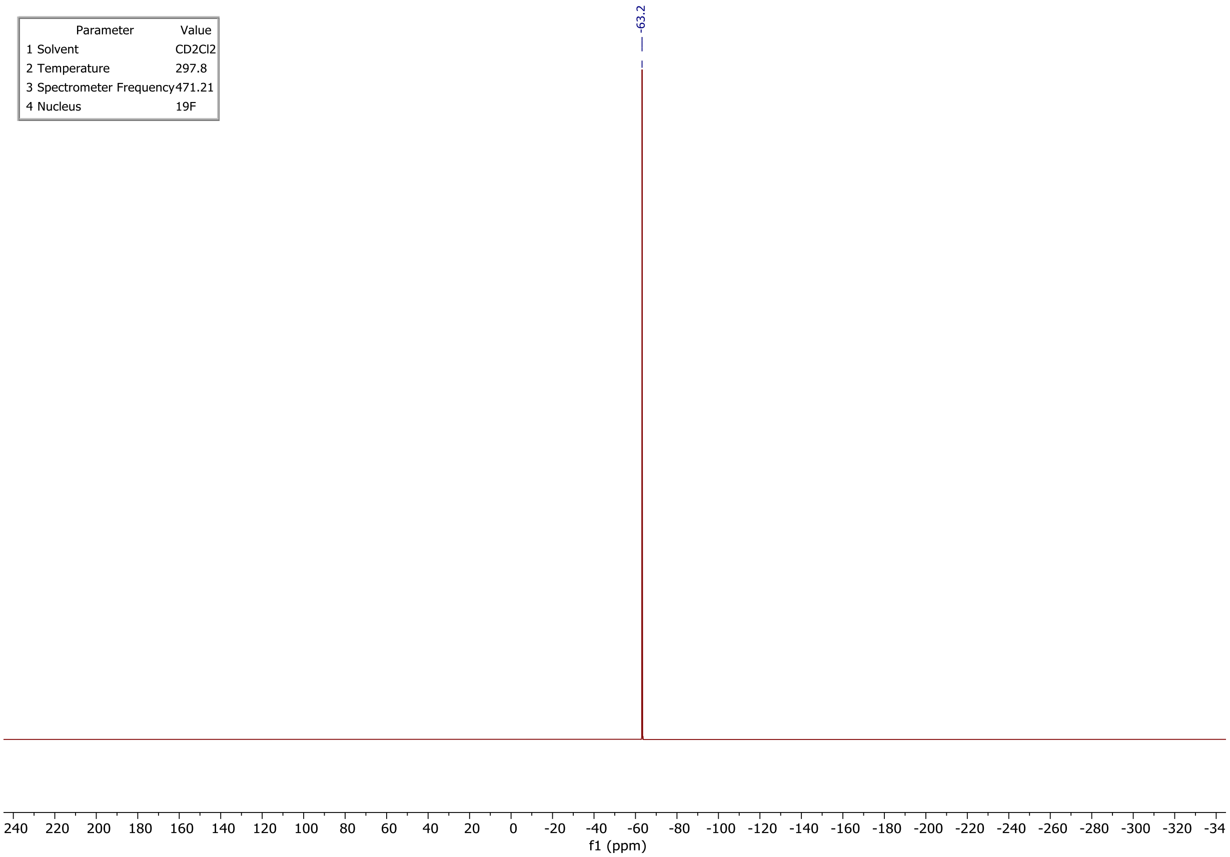

Parameter Value
1 Solvent CD2Cl2
2 Temperature 297.8
3 Spectrometer Frequency 471.21
4 Nucleus 19F
-63.2
f1 (ppm)

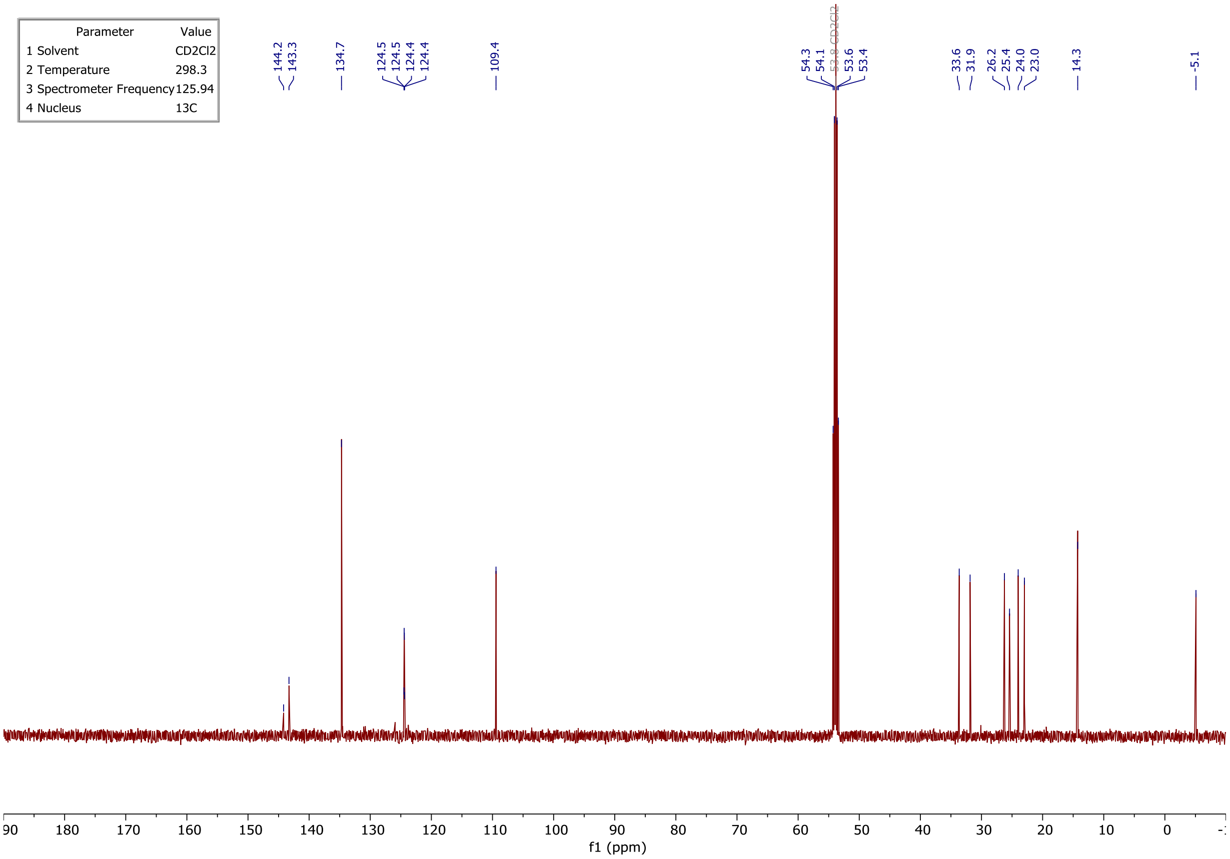

Parameter Value
1 Solvent CD2Cl2
2 Temperature 298.3
3 Spectrometer Frequency 125.94
4 Nucleus 13C
144.2
143.3
134.7
124.5
124.5
124.4
124.4
109.4
54.3
54.1
53.8 CD2Cl2
53.6
53.4
33.6
31.9
26.2
25.4
24.0
23.0
14.3
-5.1
f1 (ppm)

hexyl(3-methoxyphenyl)(methyl)(2-methylallyl)silane 1l

Parameter	Value
1 Solvent	CD2Cl2
2 Temperature	297.9
3 Spectrometer Frequency	500.81
4 Nucleus	1H

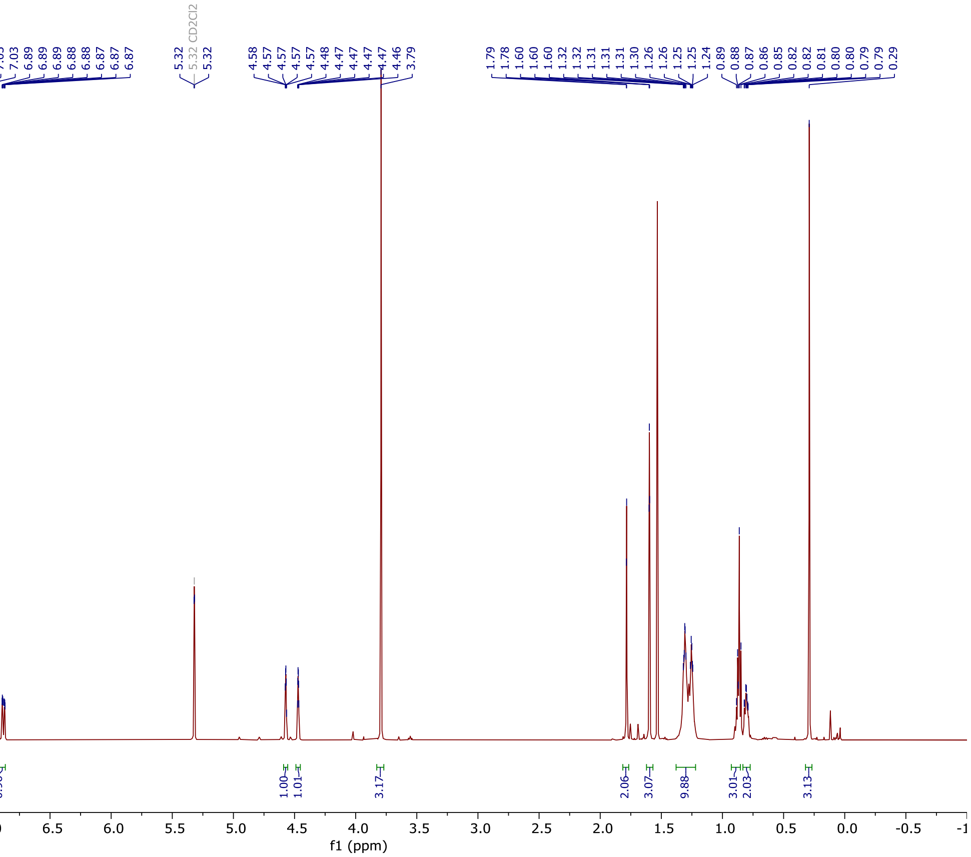

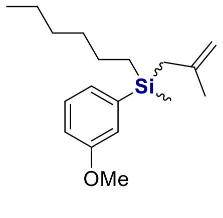

1l

Parameter	Value
1 Solvent	CD2Cl2
2 Temperature	298.0
3 Spectrometer Frequency	125.94
4 Nucleus	13C

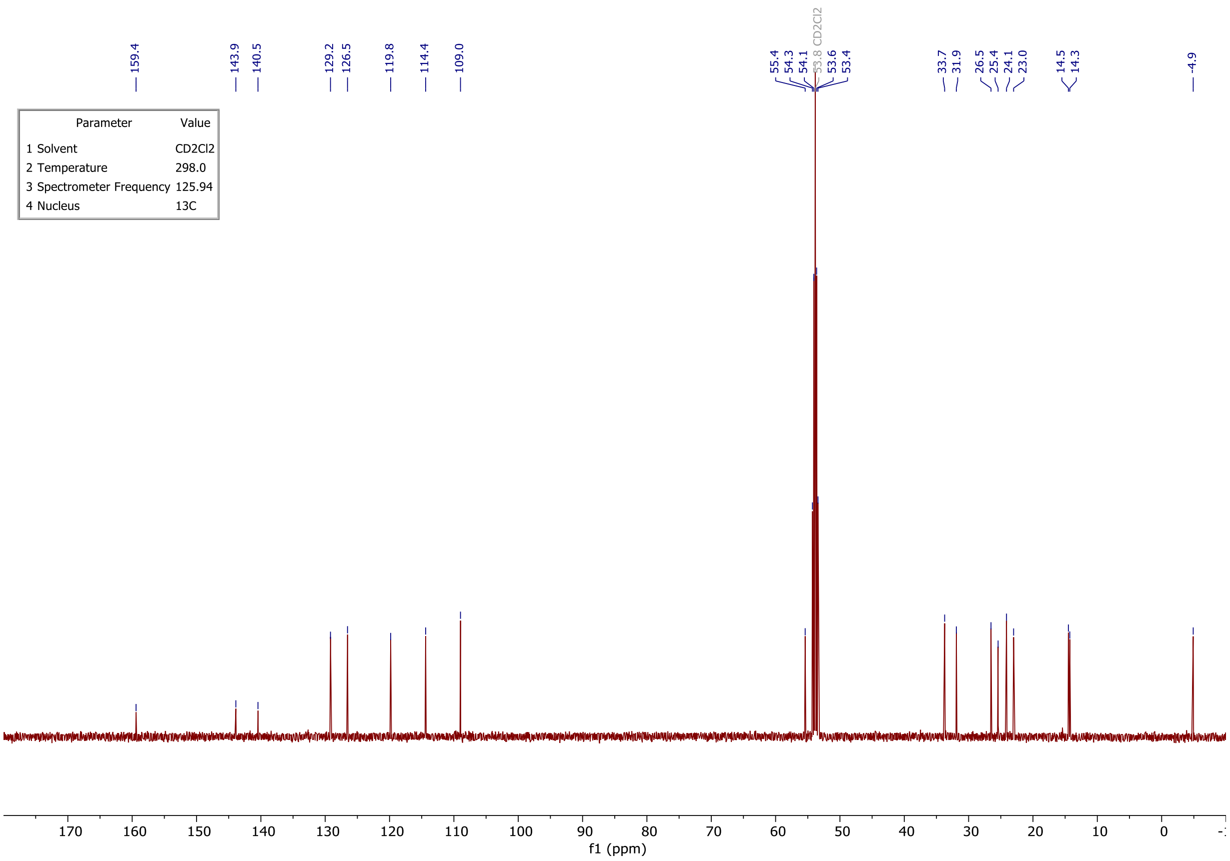

hexyl(methyl)(2-methylallyl)(o-tolyl)silane 1m

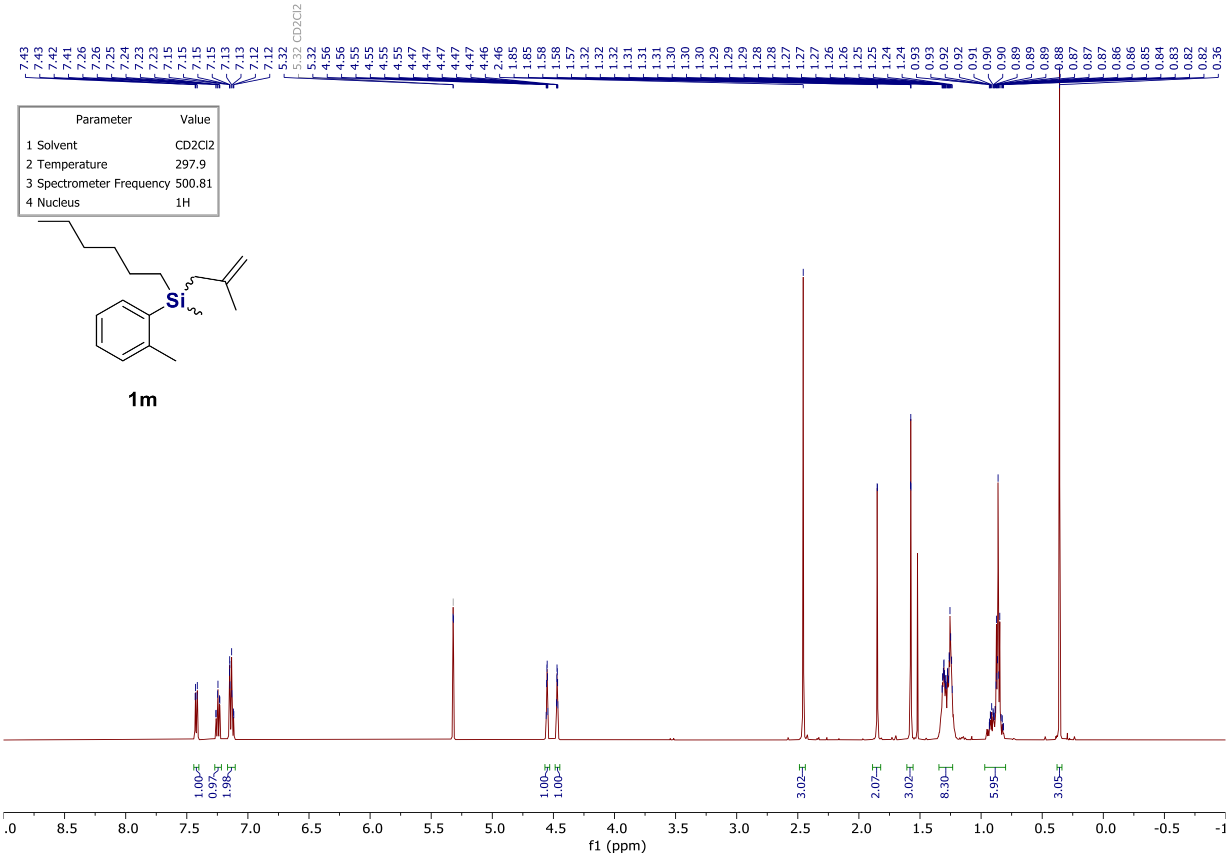

Parameter	Value
1 Solvent	CD2Cl2
2 Temperature	297.9
3 Spectrometer Frequency	500.81
4 Nucleus	1H

1m

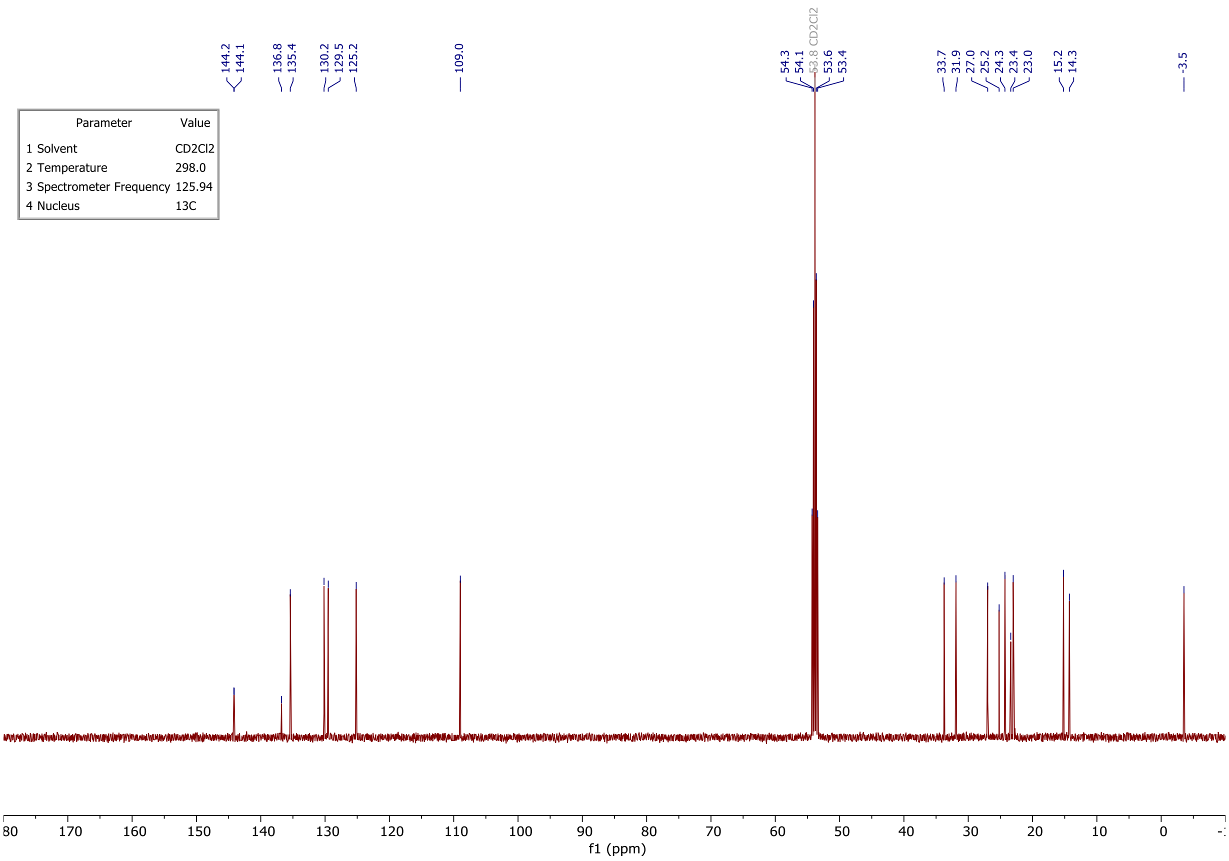

Parameter	Value
1 Solvent	CD2Cl2
2 Temperature	298.0
3 Spectrometer Frequency	125.94
4 Nucleus	13C

hexyl(methyl)(2-methylallyl)(naphthalen-2-yl)silane 1n

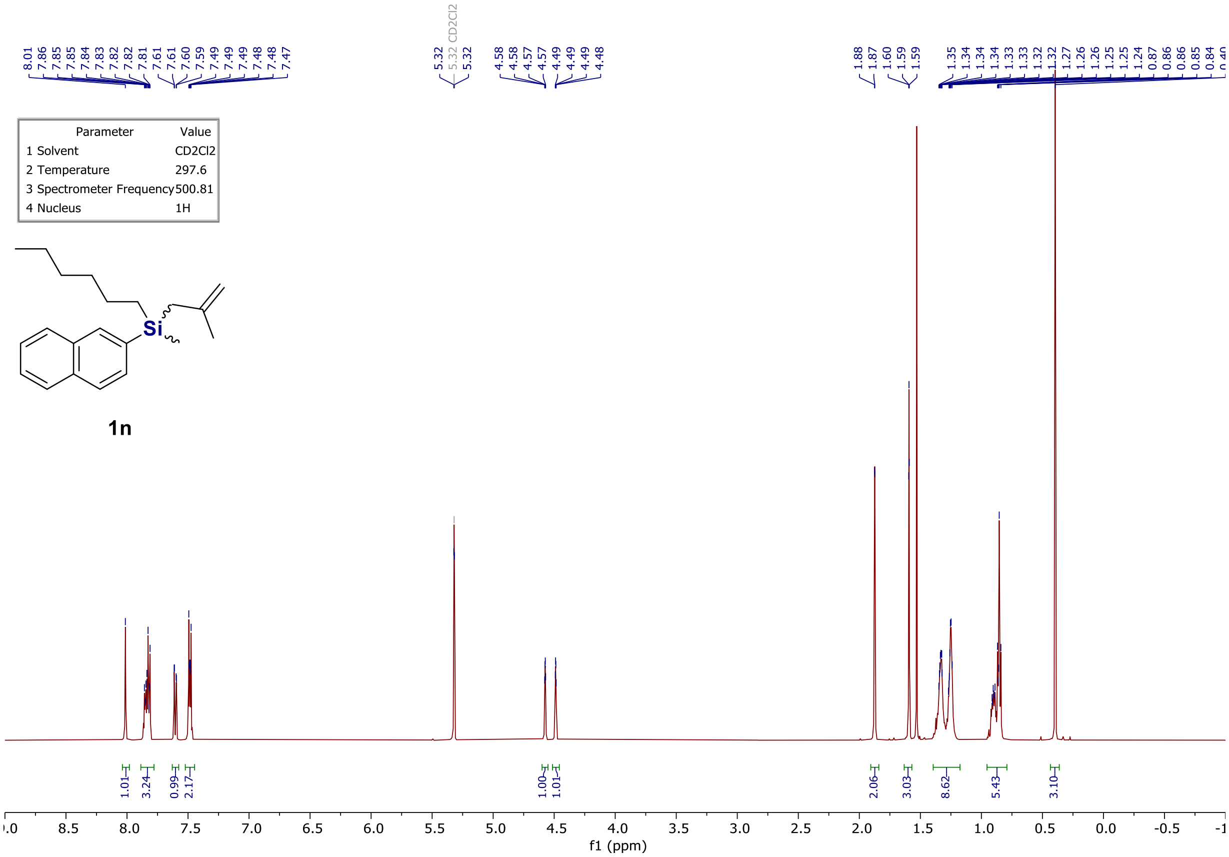

1n

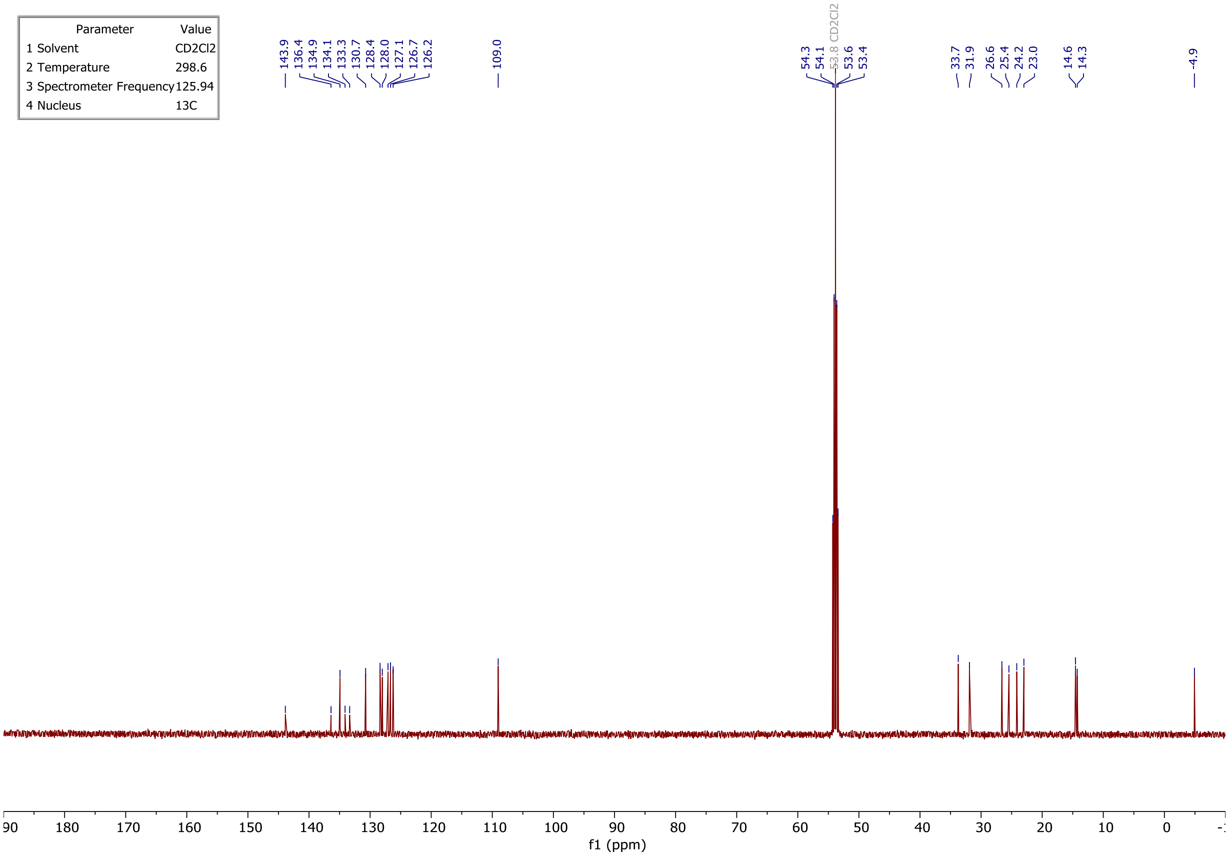

hexyl(methyl)(2-methylallyl)(thiophen-2-yl)silane 1o

Parameter	Value
1 Solvent	CD2Cl2
2 Temperature	297.3
3 Spectrometer Frequency	500.81
4 Nucleus	1H

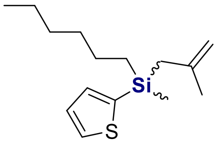

1o

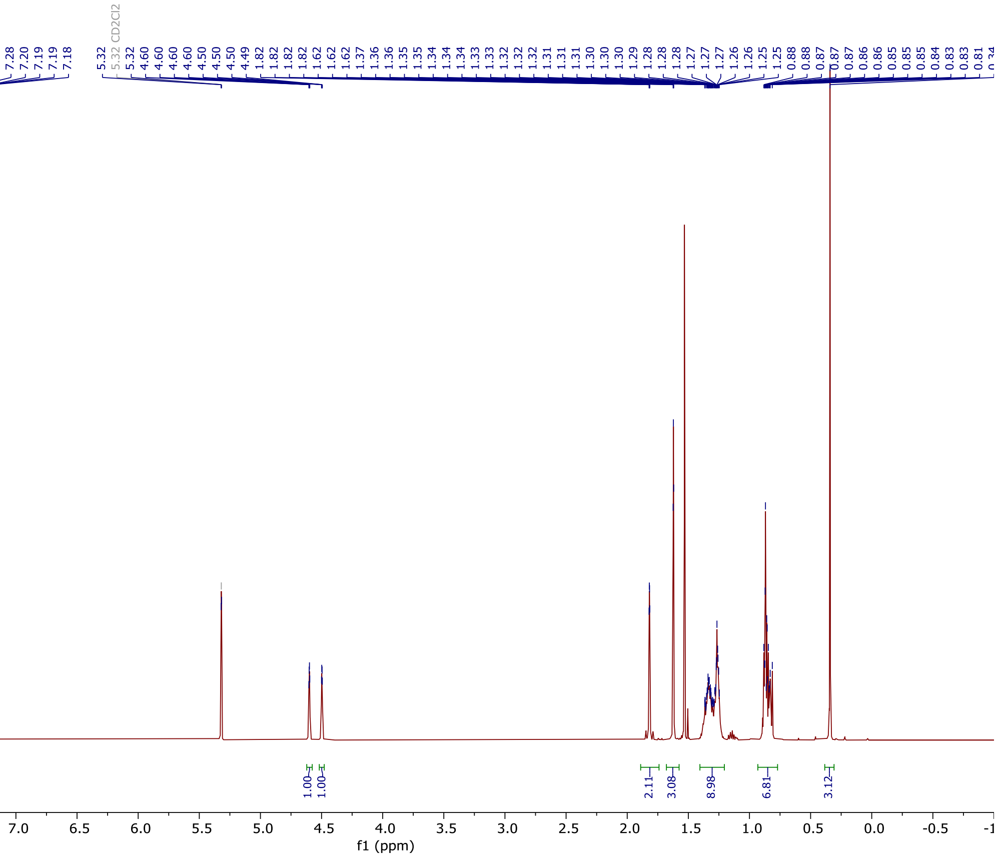

Parameter	Value
1 Solvent	CD2Cl2
2 Temperature	298.5
3 Spectrometer Frequency	125.94
4 Nucleus	13C

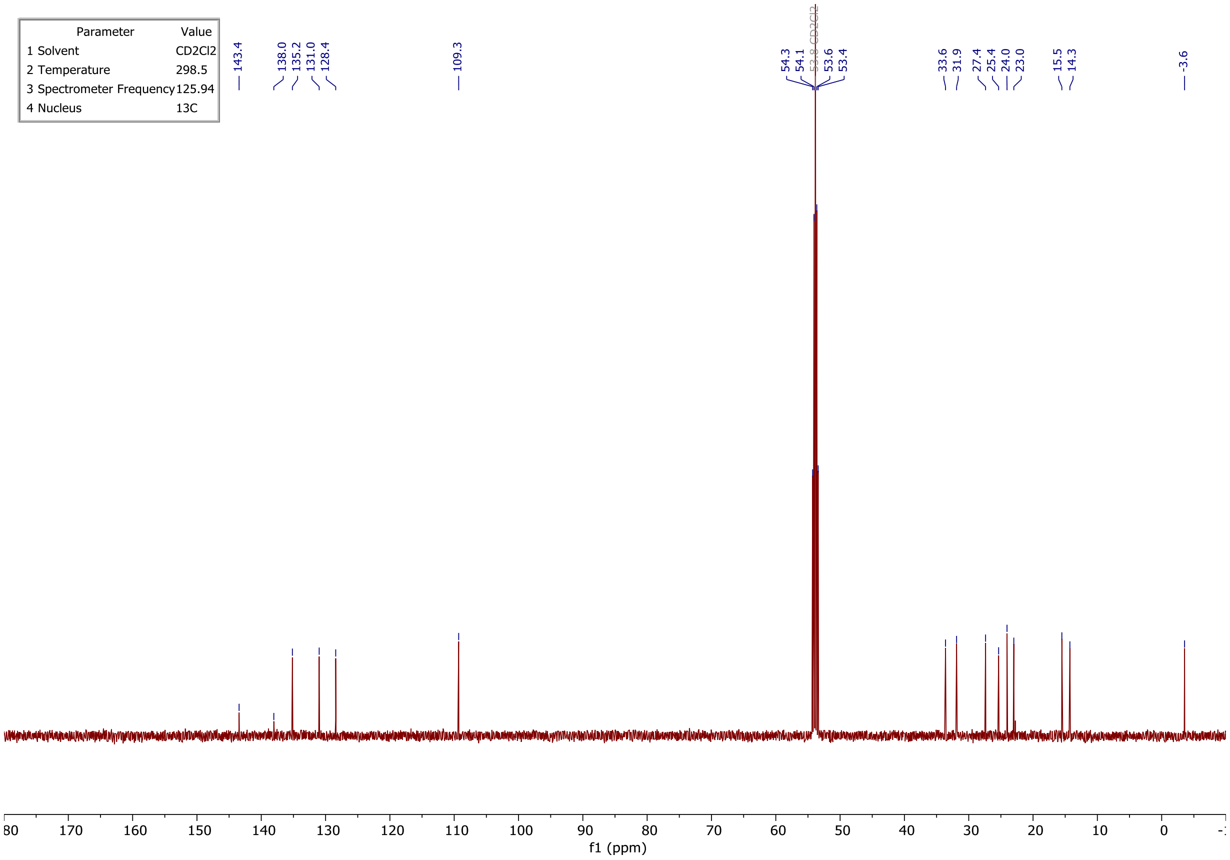

allyl(methyl)(2-methylallyl)(phenyl)silane 1p

Parameter	Value
1 Solvent	CD2Cl2
2 Temperature	297.9
3 Spectrometer Frequency	500.81
4 Nucleus	1H

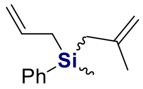

1p

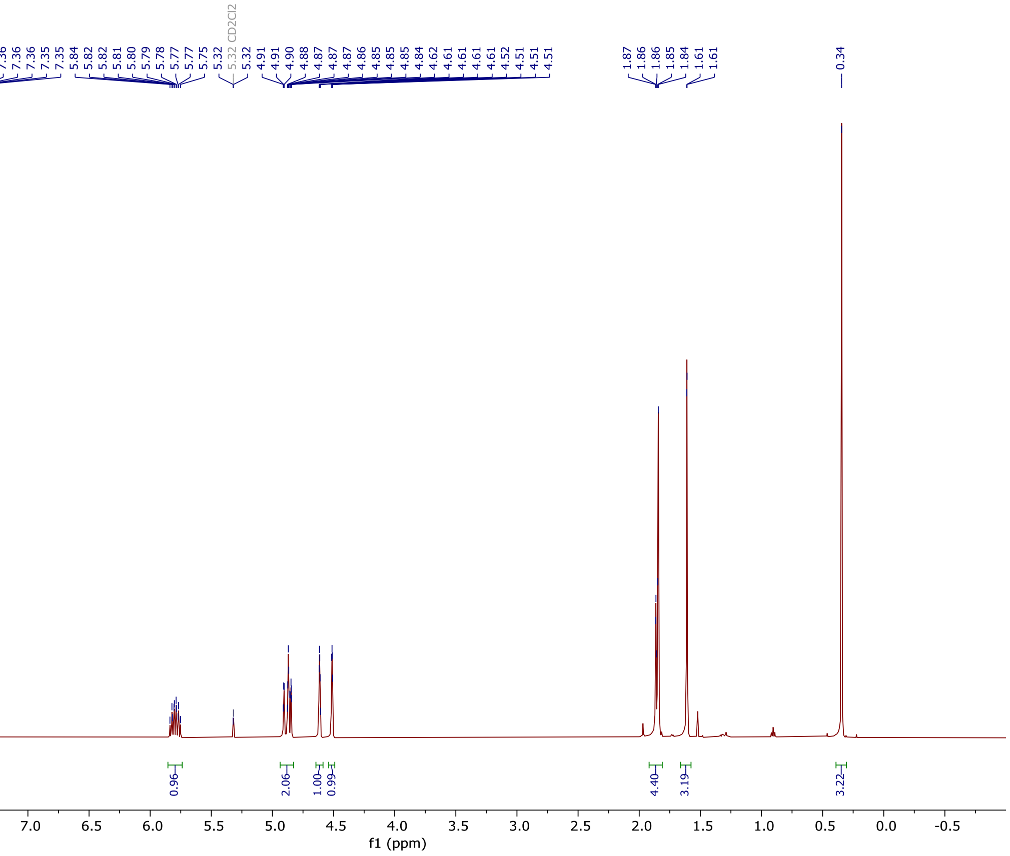

Parameter	Value
1 Solvent	CD2Cl2
2 Temperature	298.0
3 Spectrometer Frequency	125.94
4 Nucleus	13C

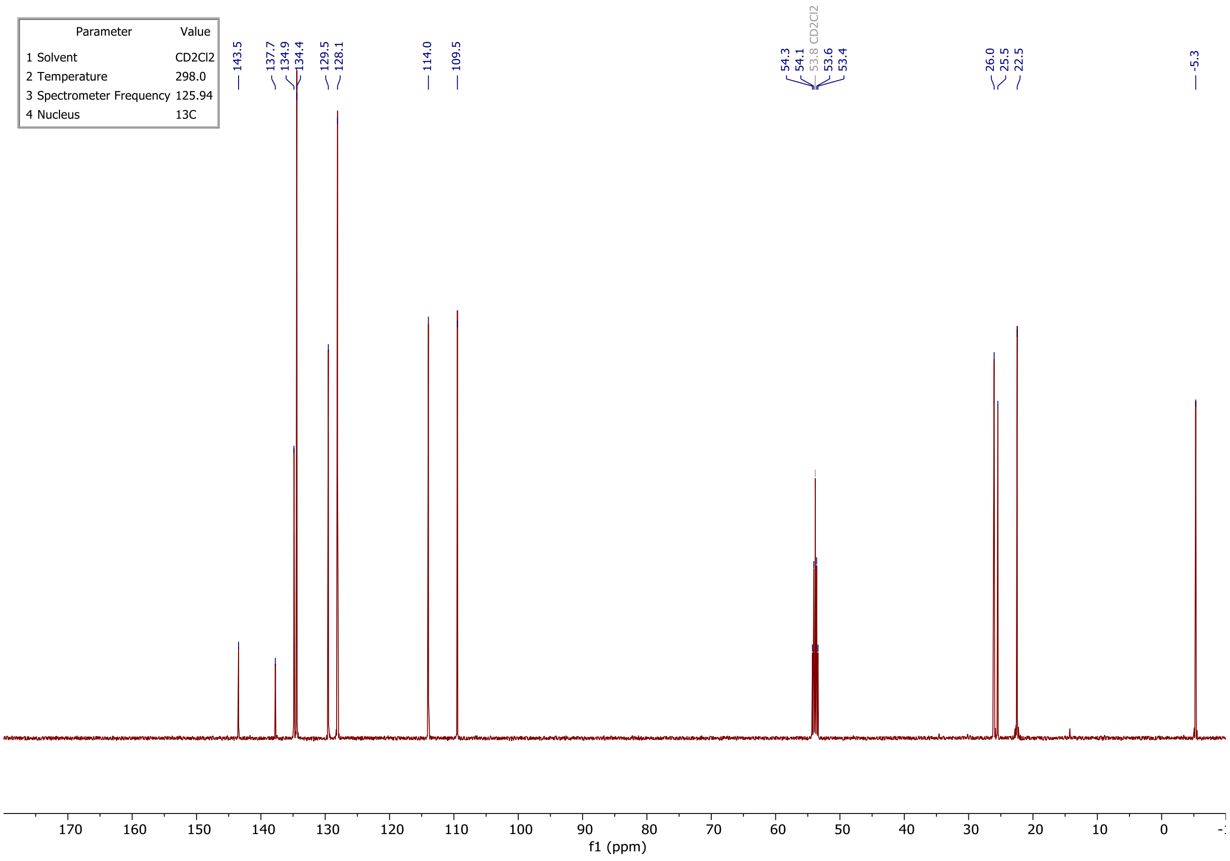

methyl(2-methylallyl)(phenyl)(prop-1-en-2-yl)silane 1q

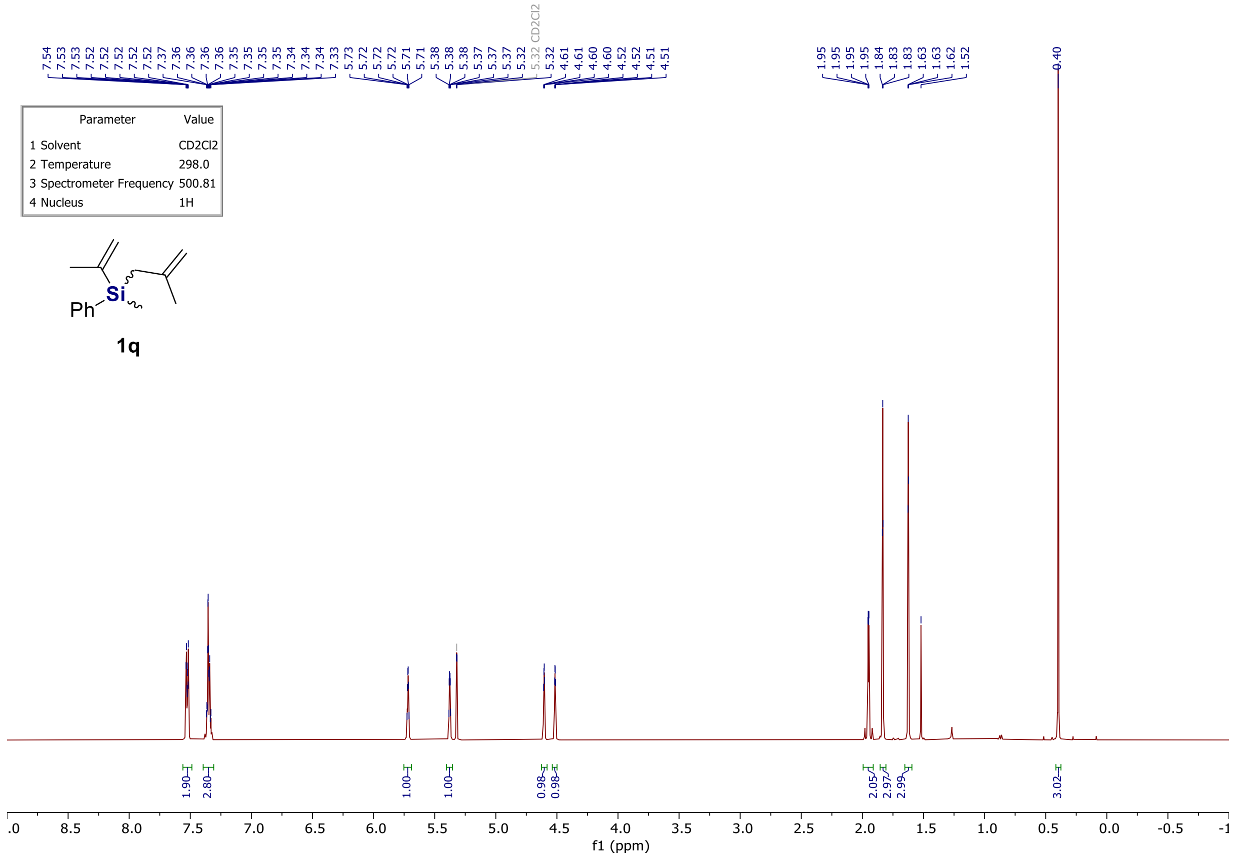

Parameter	Value
1 Solvent	CD2Cl2
2 Temperature	298.0
3 Spectrometer Frequency	500.81
4 Nucleus	1H

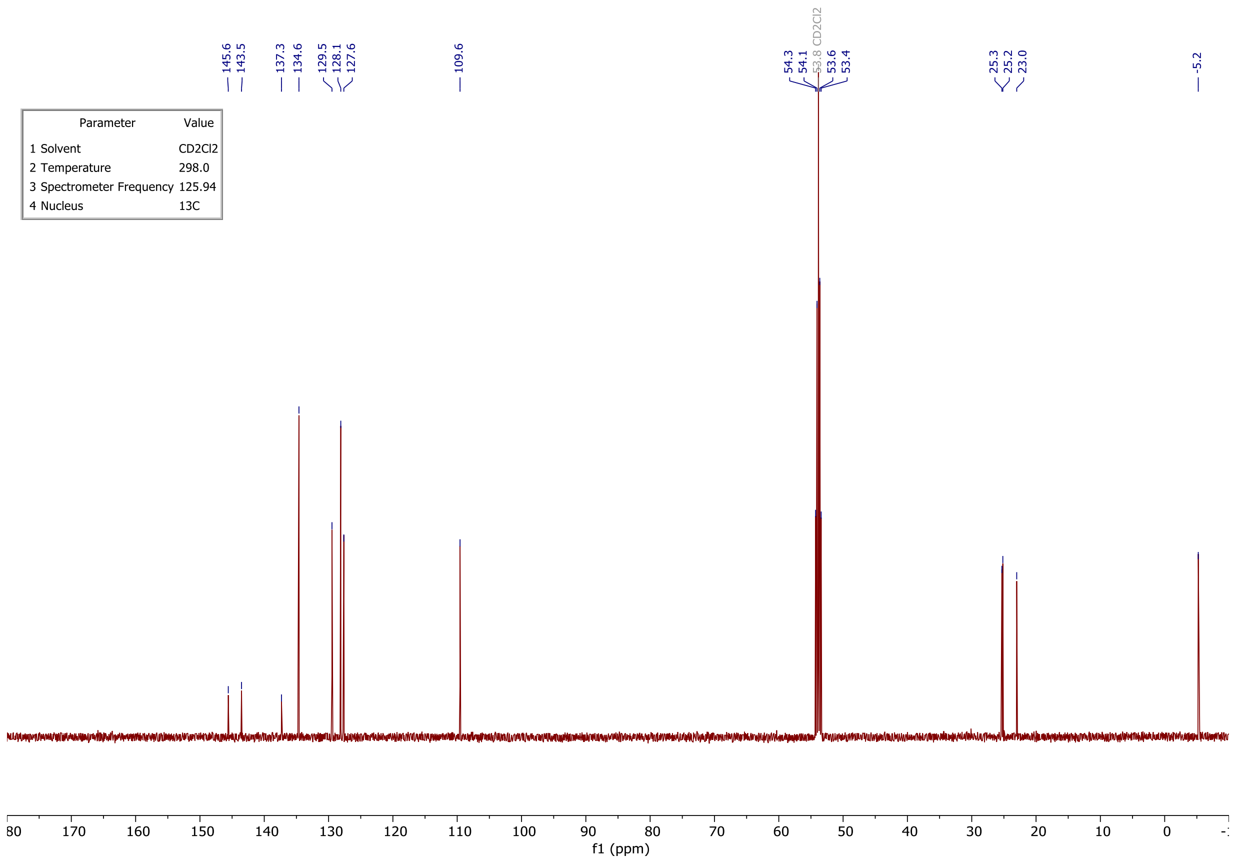

Parameter	Value
1 Solvent	CD2Cl2
2 Temperature	298.0
3 Spectrometer Frequency	125.94
4 Nucleus	13C

methyl(2-methylallyl)(4-methylpent-3-en-1-yl)(phenyl)silane 1r

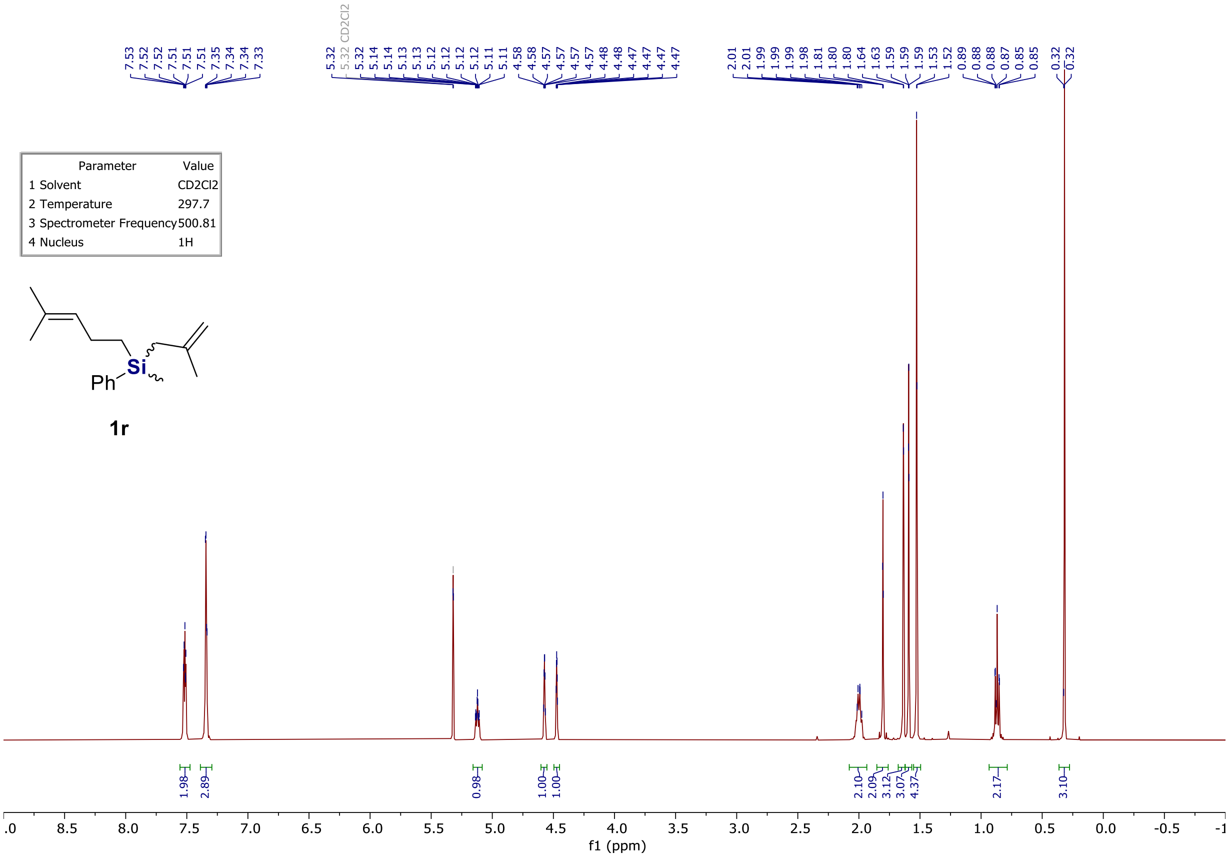

Parameter	Value
1 Solvent	CD2Cl2
2 Temperature	297.7
3 Spectrometer Frequency	500.81
4 Nucleus	1H

1r

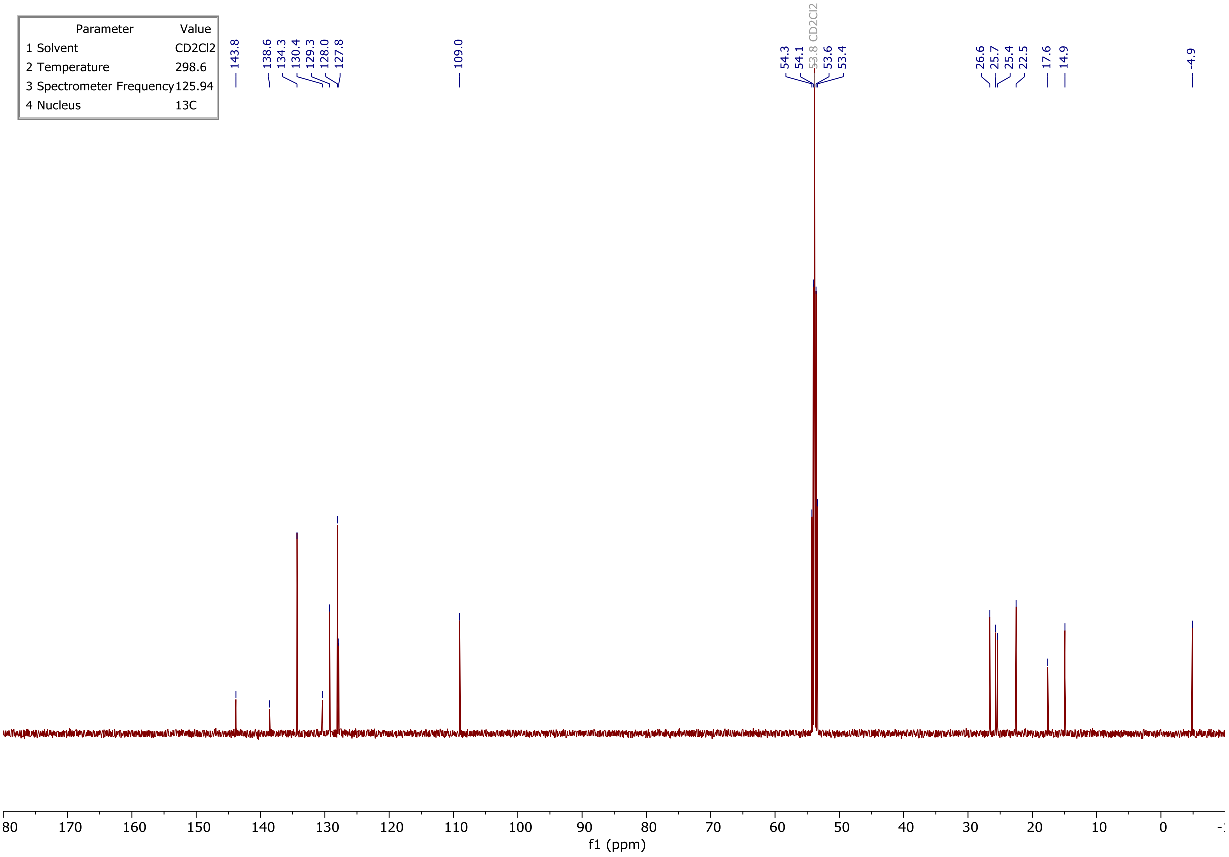

Parameter	Value
1 Solvent	CD2Cl2
2 Temperature	298.6
3 Spectrometer Frequency	125.94
4 Nucleus	13C

benzyl(4-methoxybutyl)(methyl)(2-methylallyl)silane 1s

Parameter	Value
1 Solvent	CD2Cl2
2 Temperature	298.0
3 Spectrometer Frequency	500.81
4 Nucleus	1H

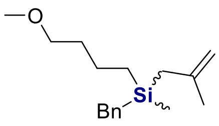

1s

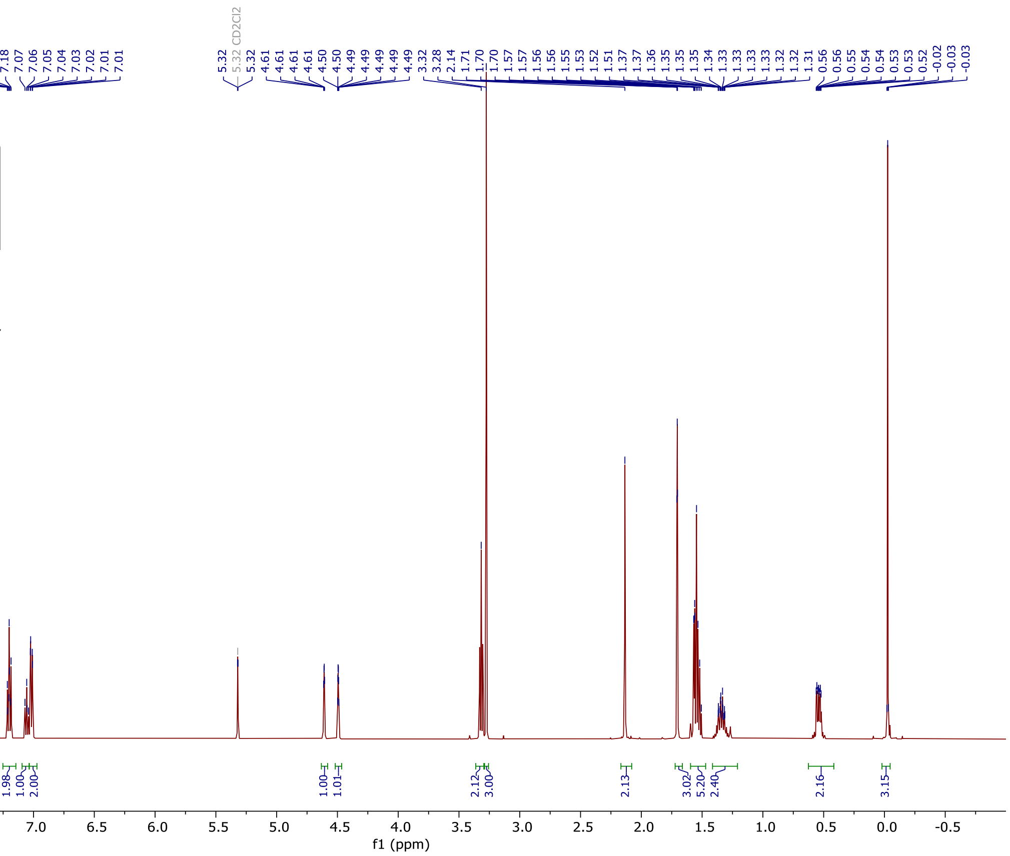

Parameter	Value
1 Solvent	CD2Cl2
2 Temperature	298.0
3 Spectrometer Frequency	125.94
4 Nucleus	13C

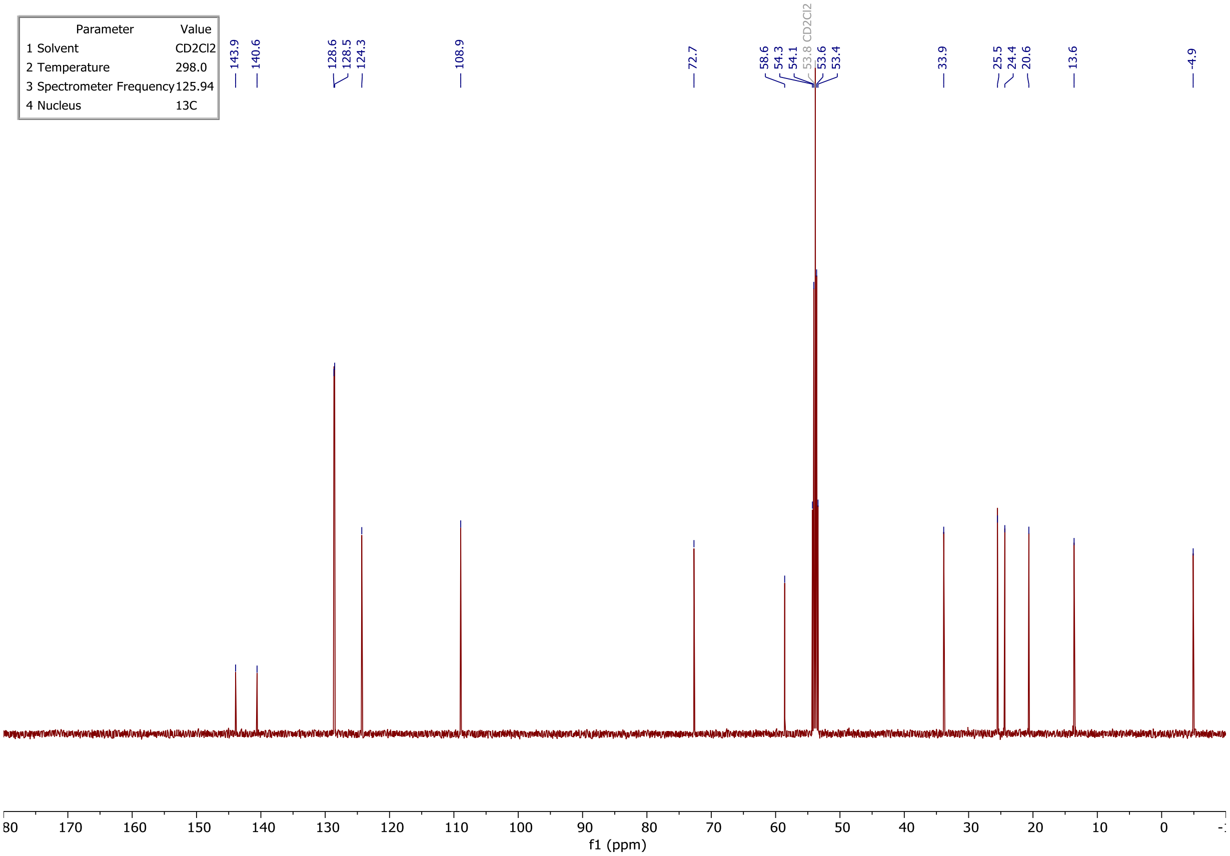

hexyl(methyl)(2-methylprop-1-en-1-yl)(phenyl)silane 1t

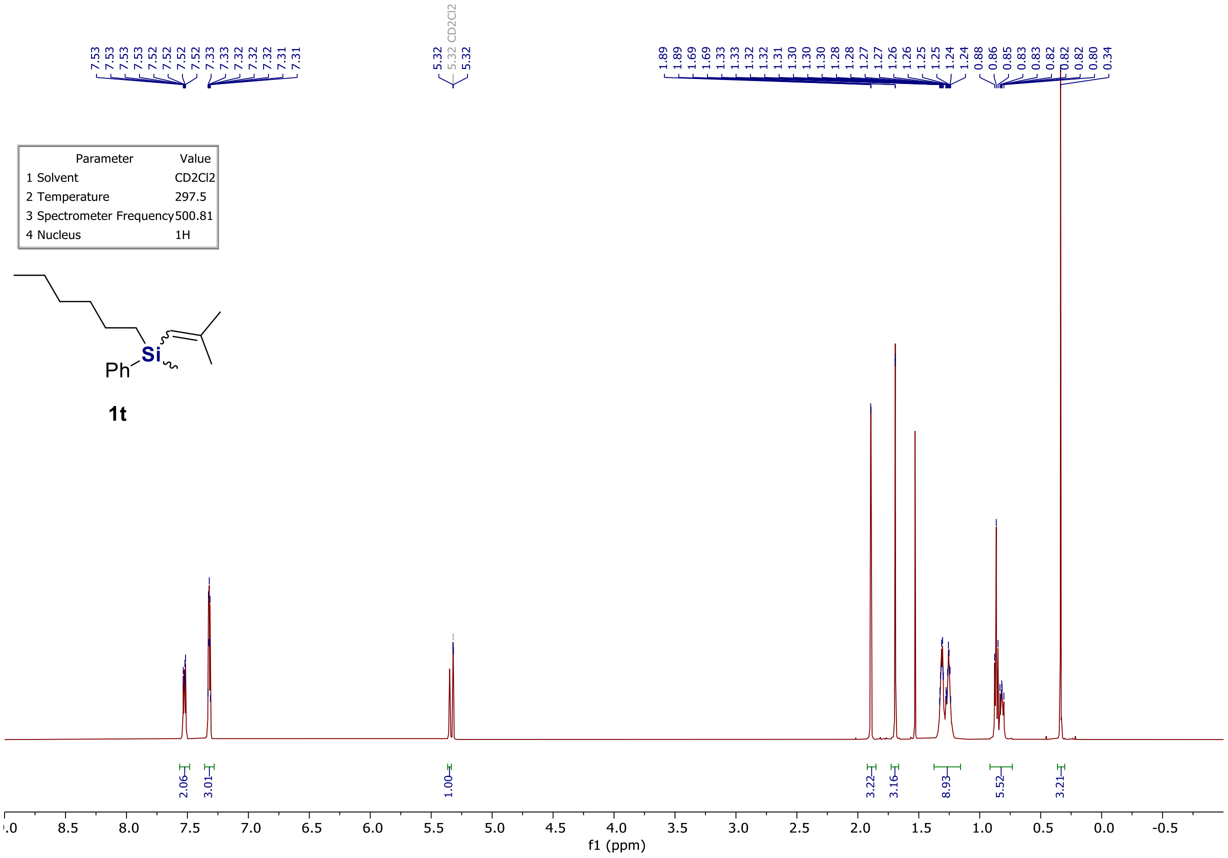

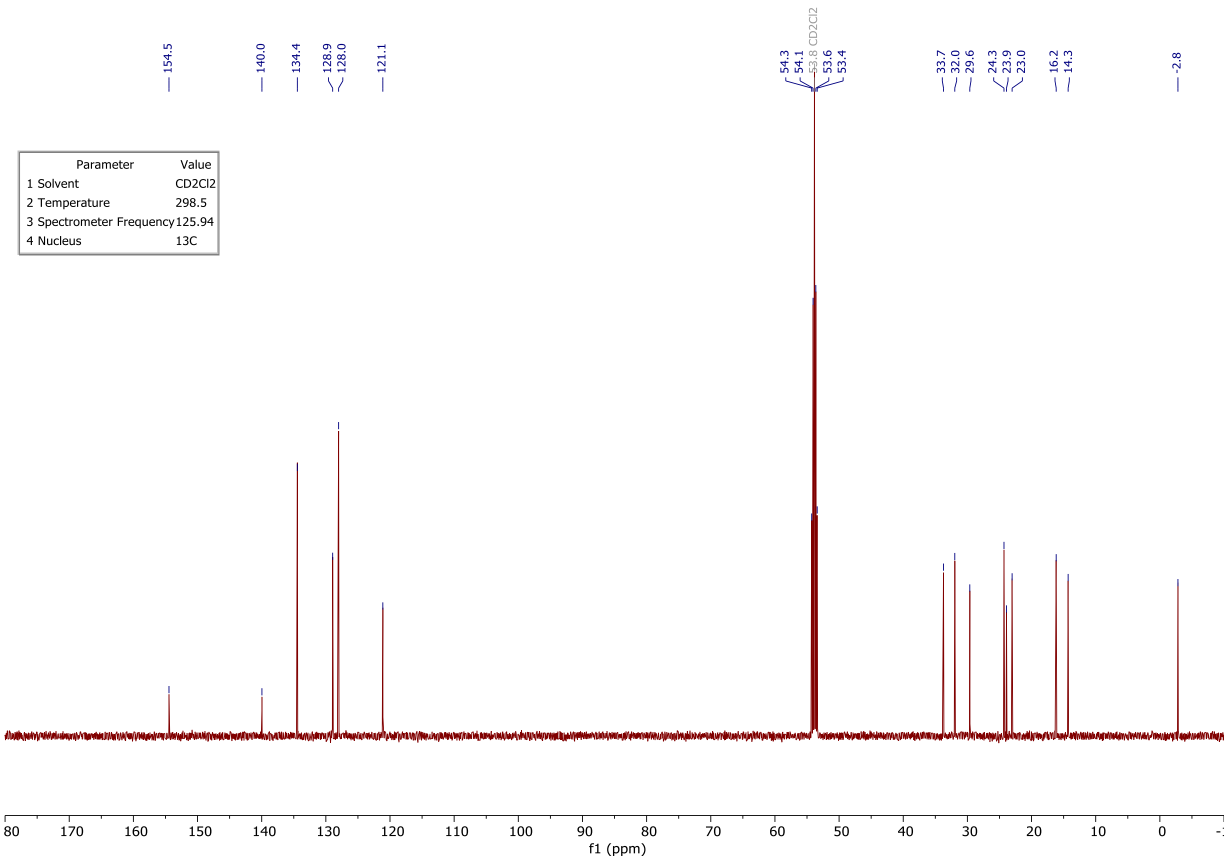

allyl(hexyl)(methyl)(phenyl)silane 1u

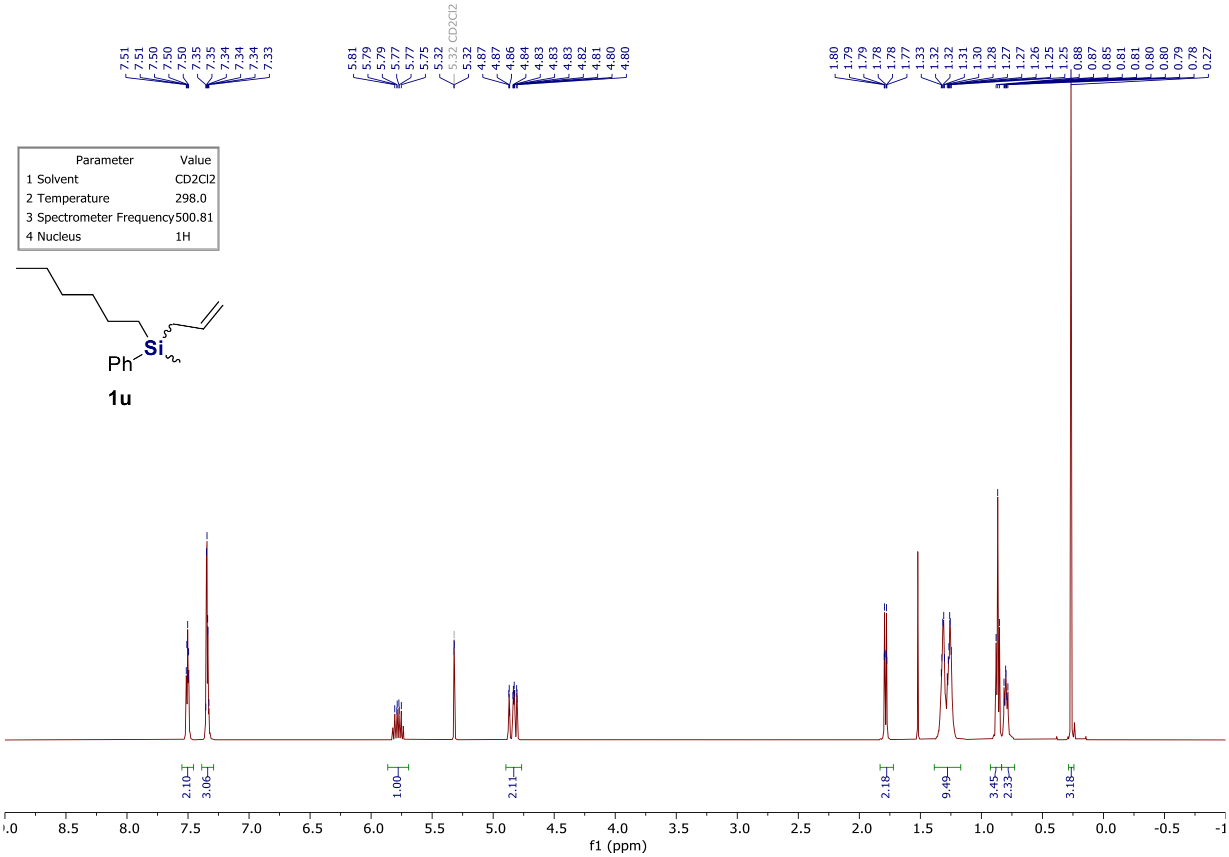

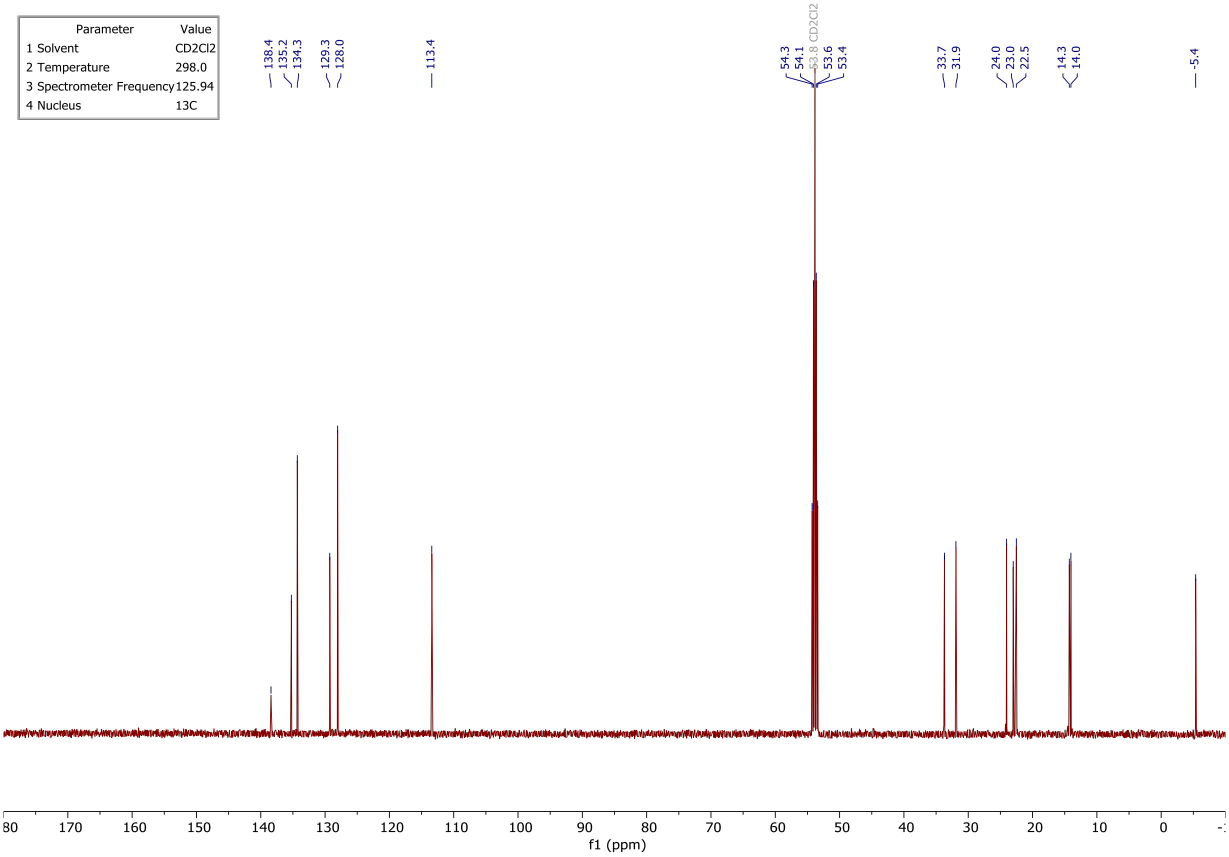

methyl(2-methylallyl)((*S*)-2-methylbutyl)(phenyl)silane 1v

Parameter	Value
1 Solvent	CD2Cl2
2 Temperature	298.0
3 Spectrometer Frequency	500.81
4 Nucleus	1H

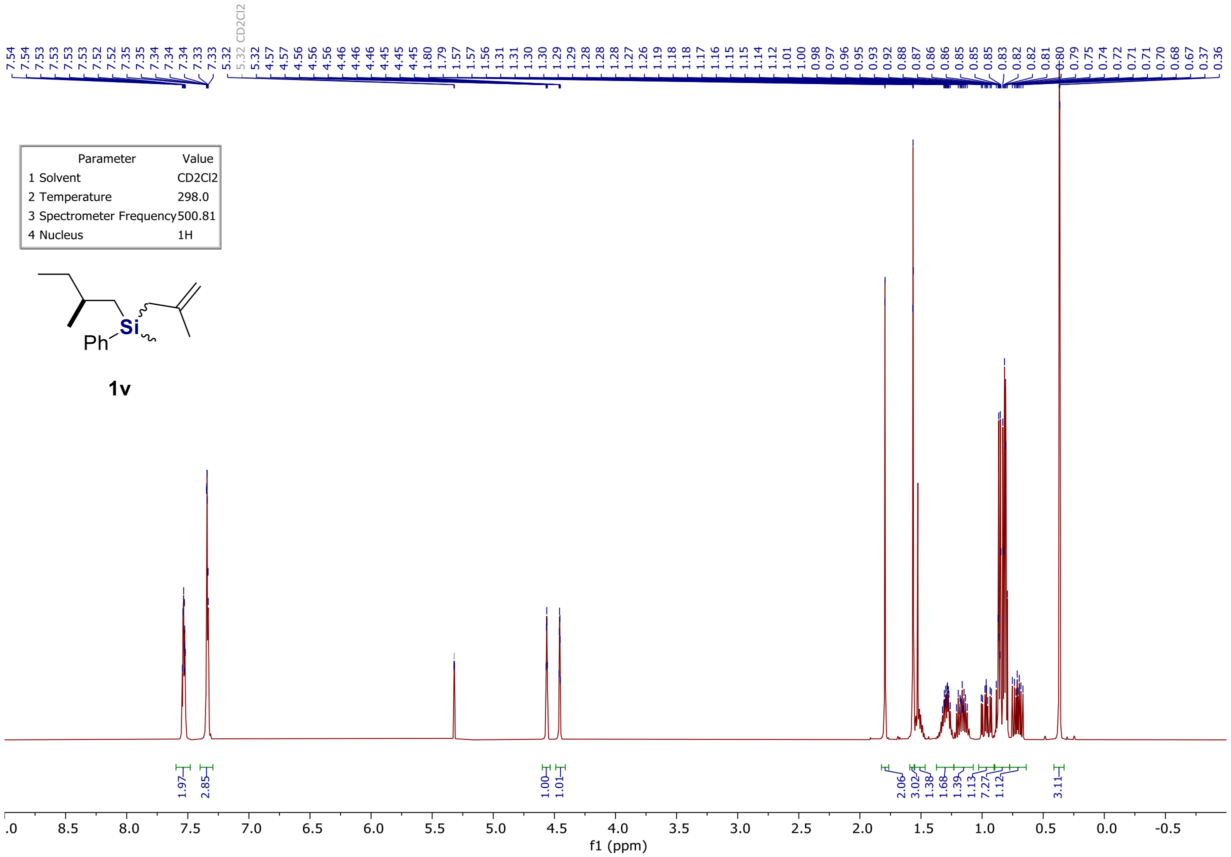

Parameter	Value
1 Solvent	CD2Cl2
2 Temperature	298.0
3 Spectrometer Frequency	125.94
4 Nucleus	13C

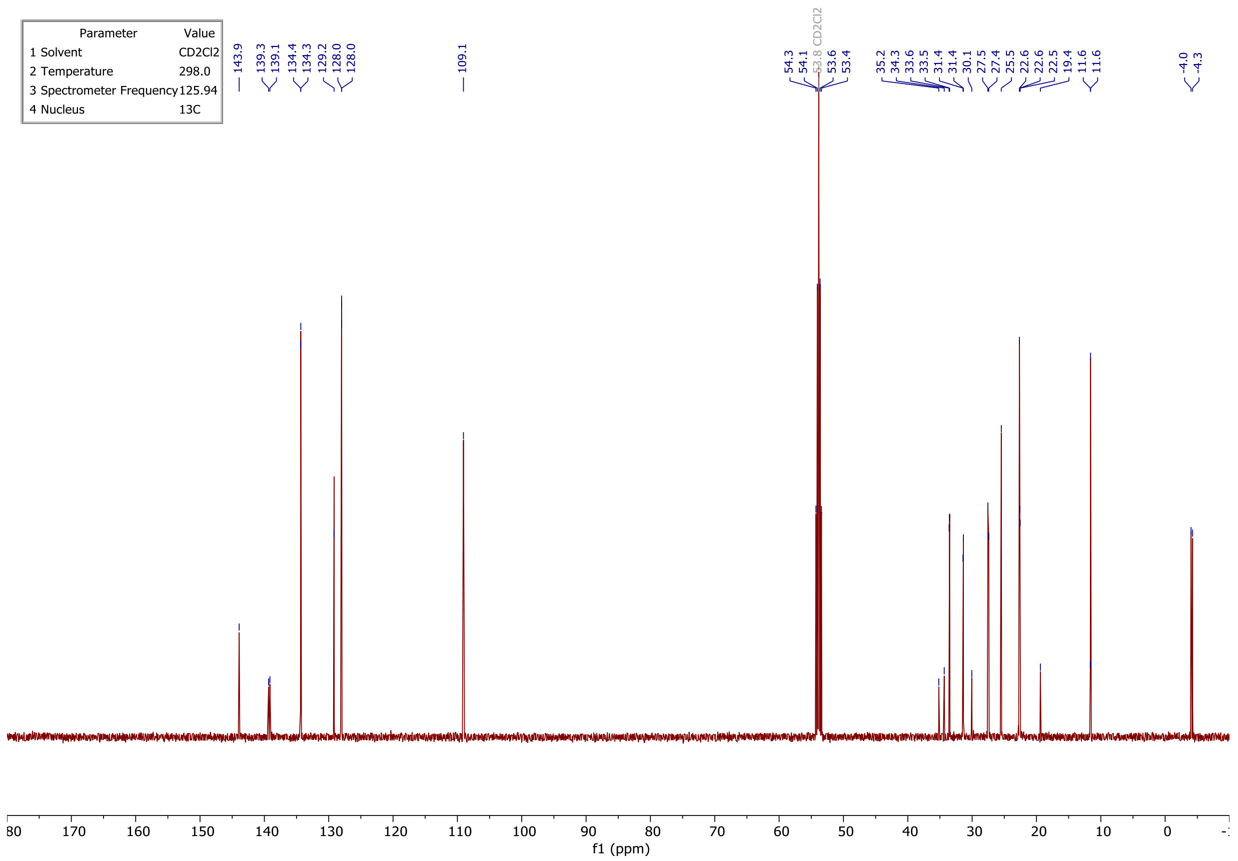

(*S*)-(2,6-diisopropylphenoxy)(hexyl)(methyl)(phenyl)silane 4a

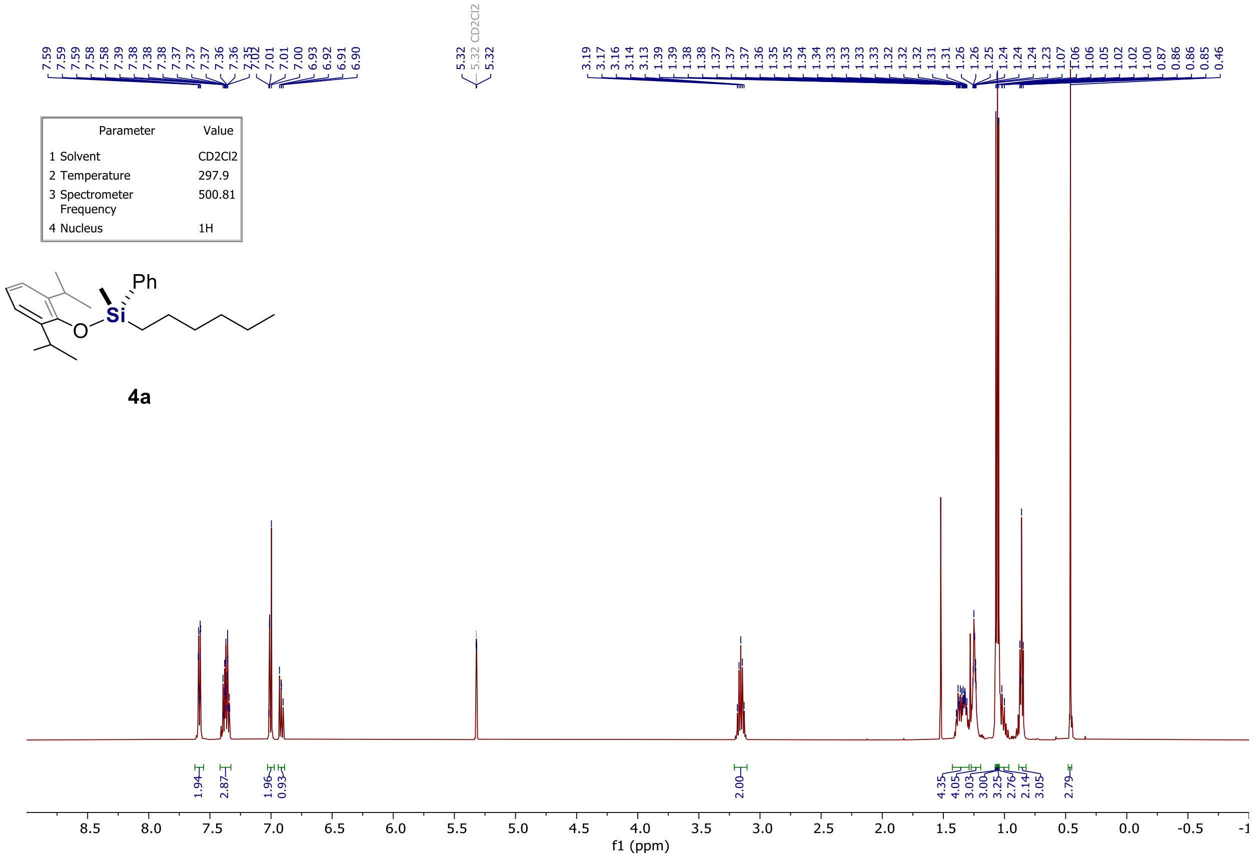

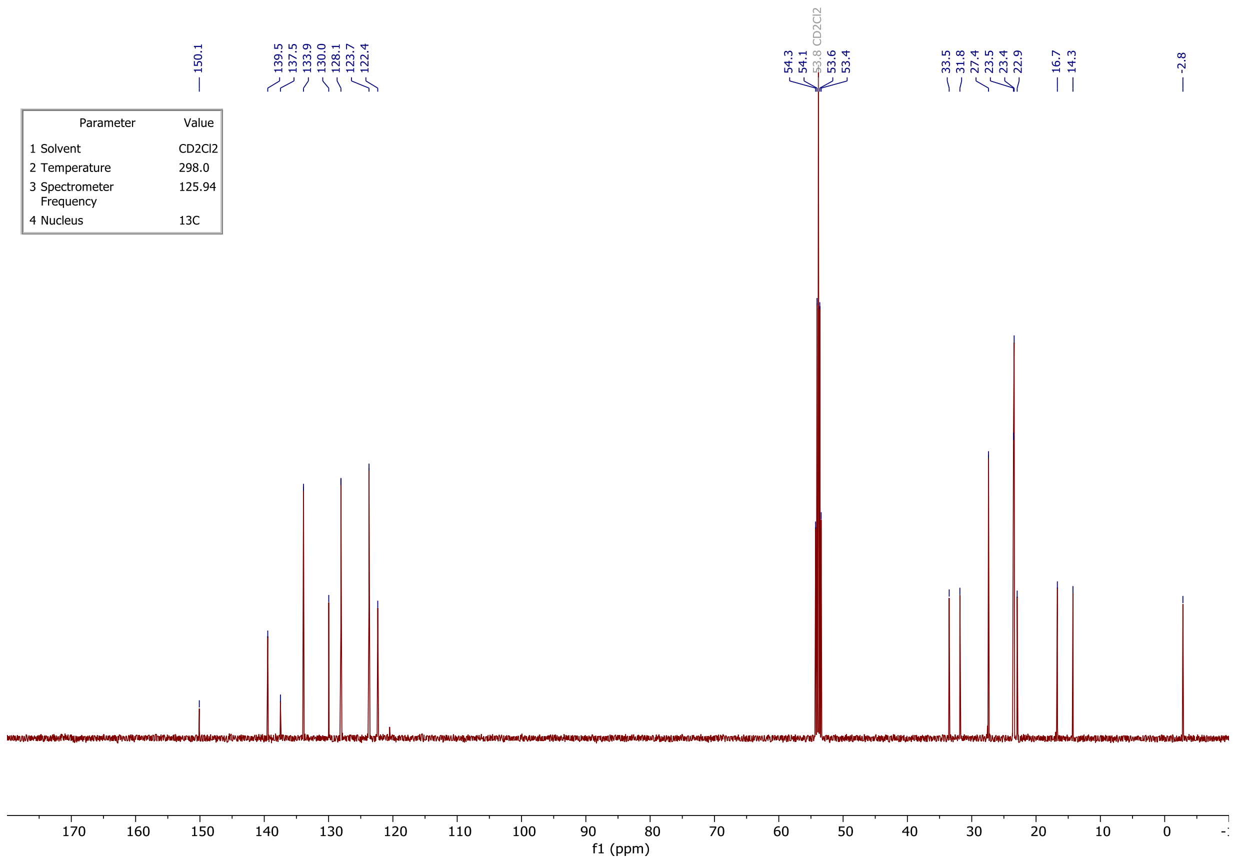

(2,6-diisopropylphenoxy)(ethyl)(methyl)(phenyl)silane 4b

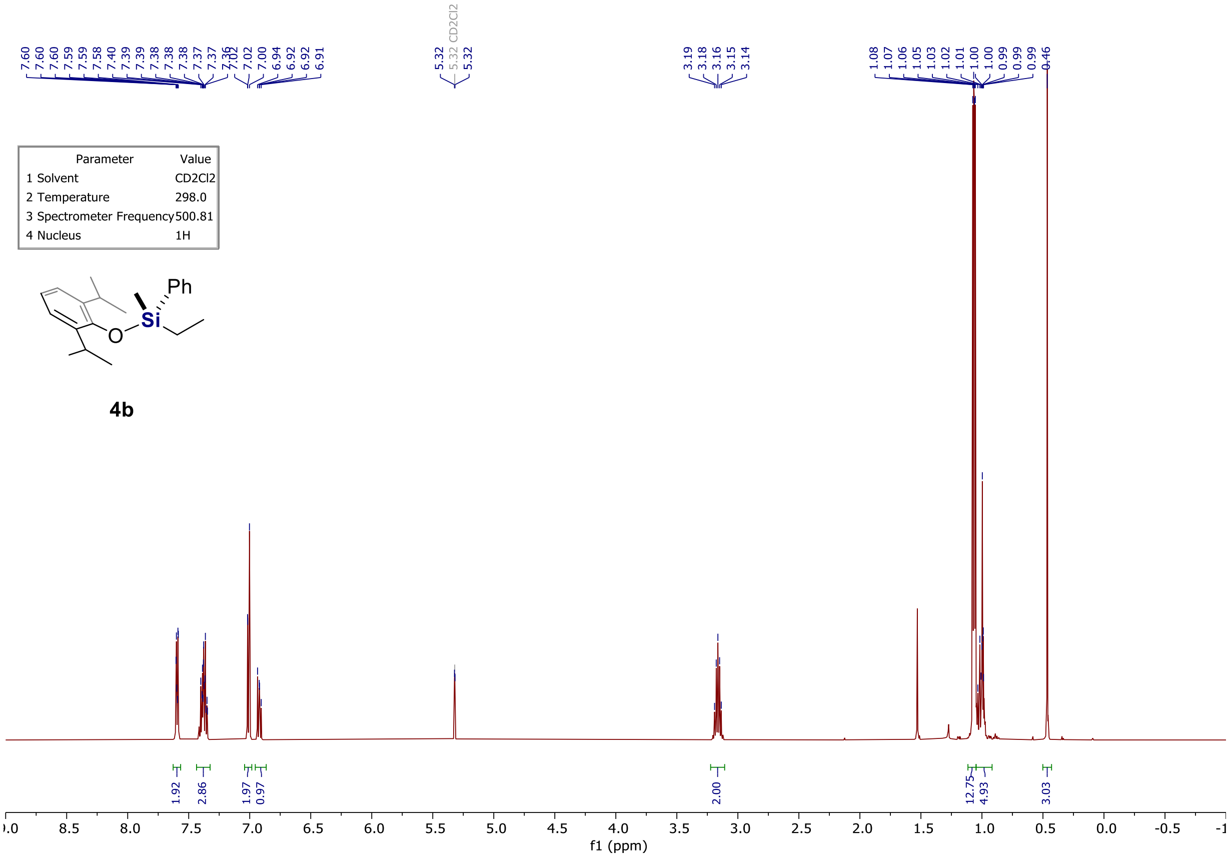

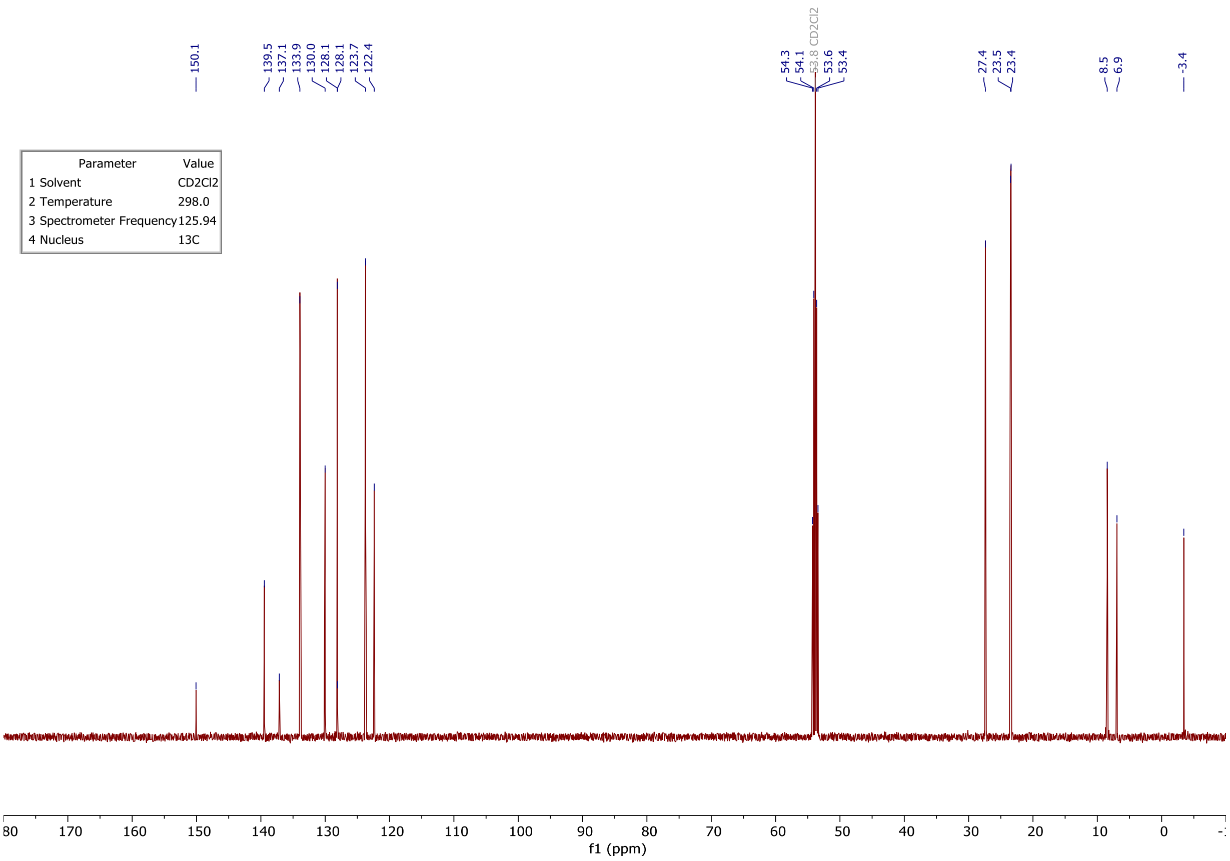

(2,6-diisopropylphenoxy)(methyl)(phenyl)(propyl)silane 4c

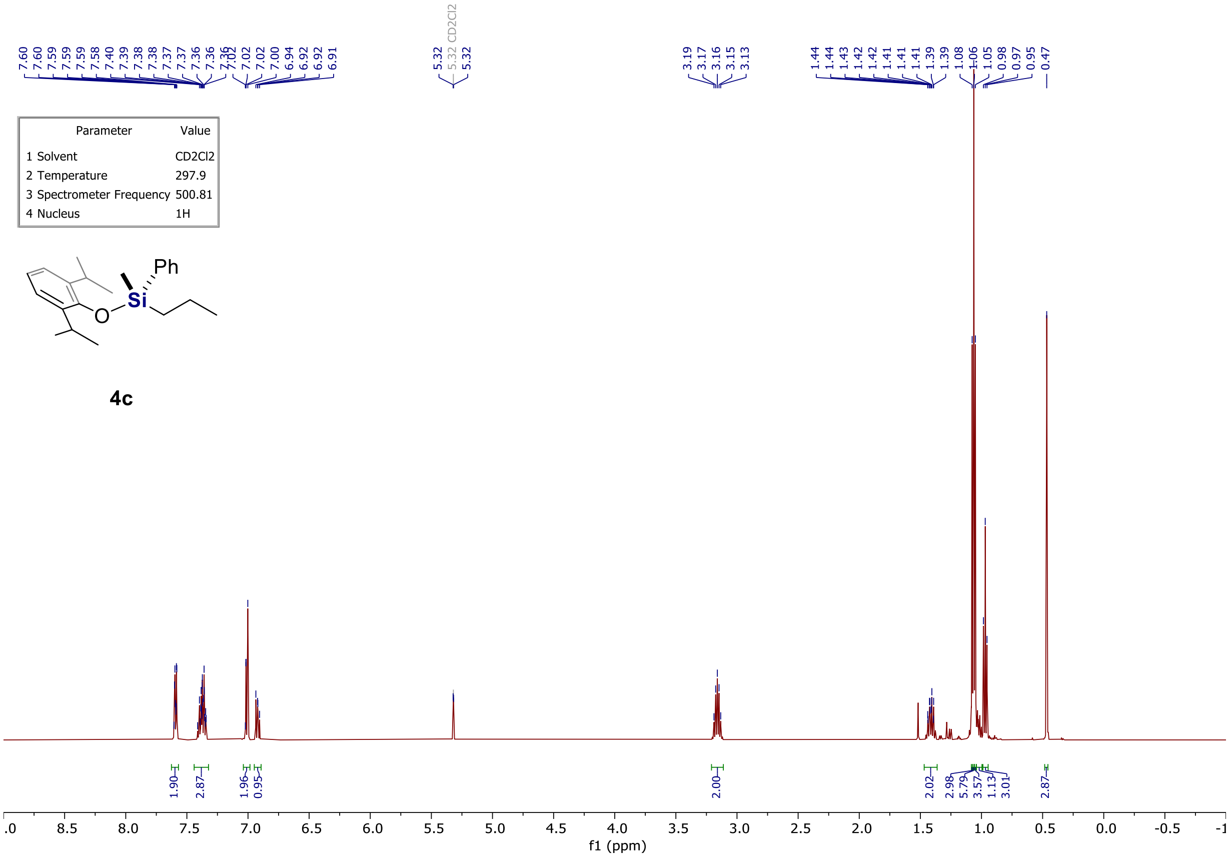

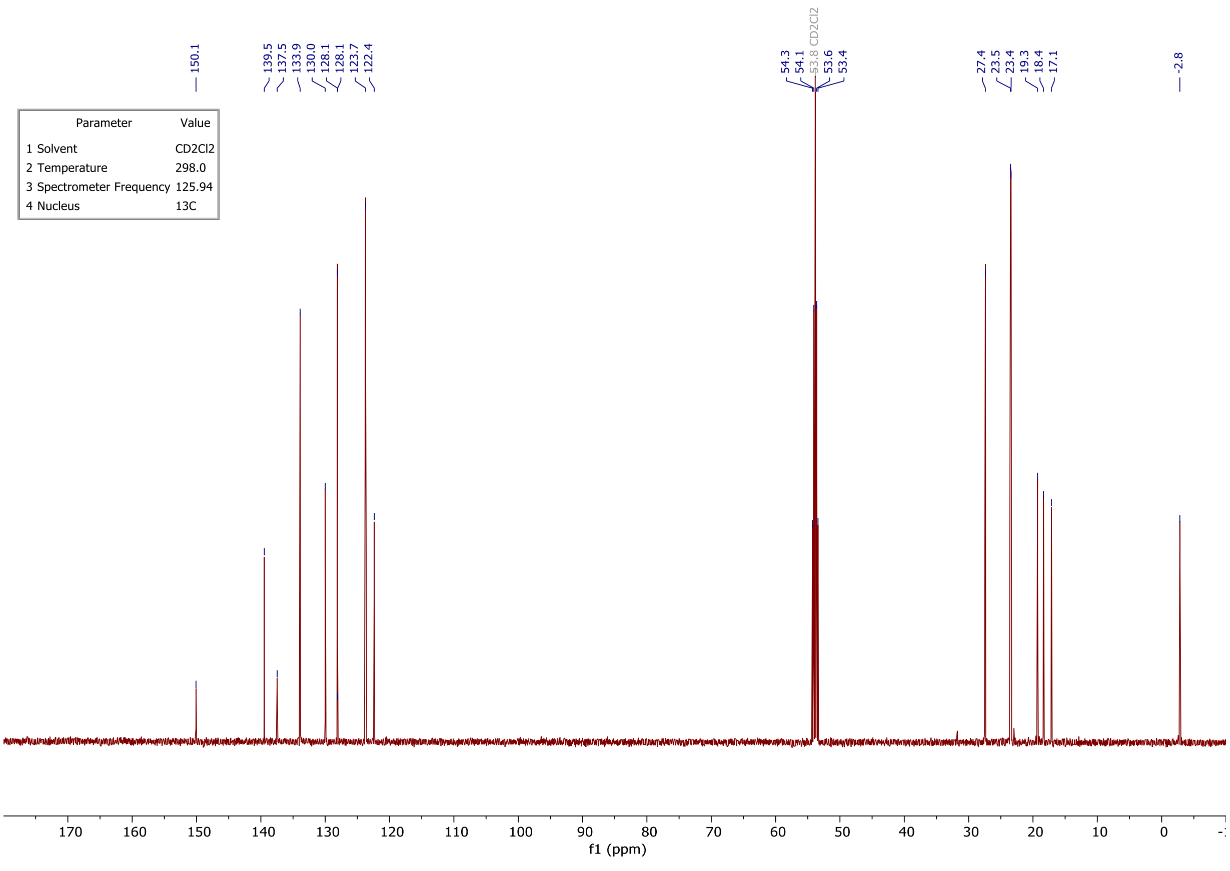

butyl(2,6-diisopropylphenoxy)(methyl)(phenyl)silane 4d

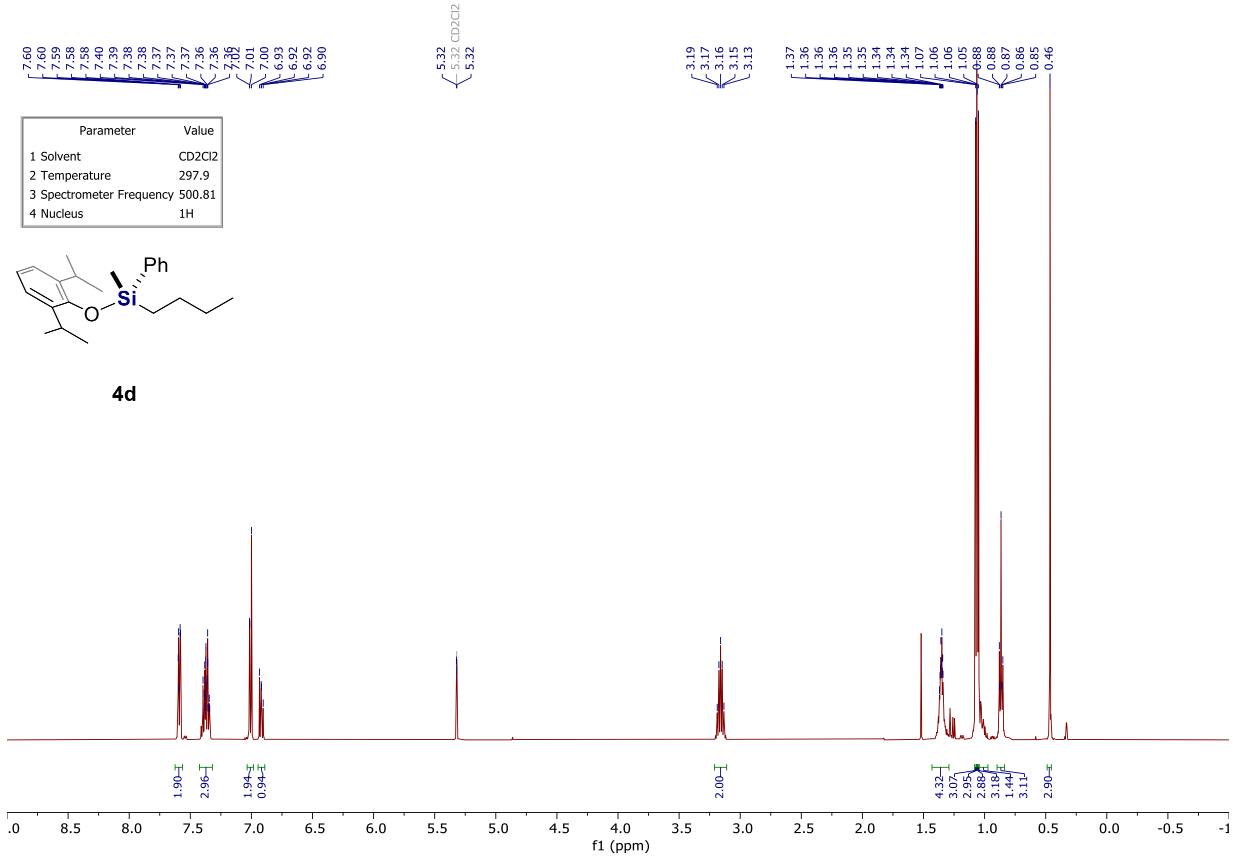

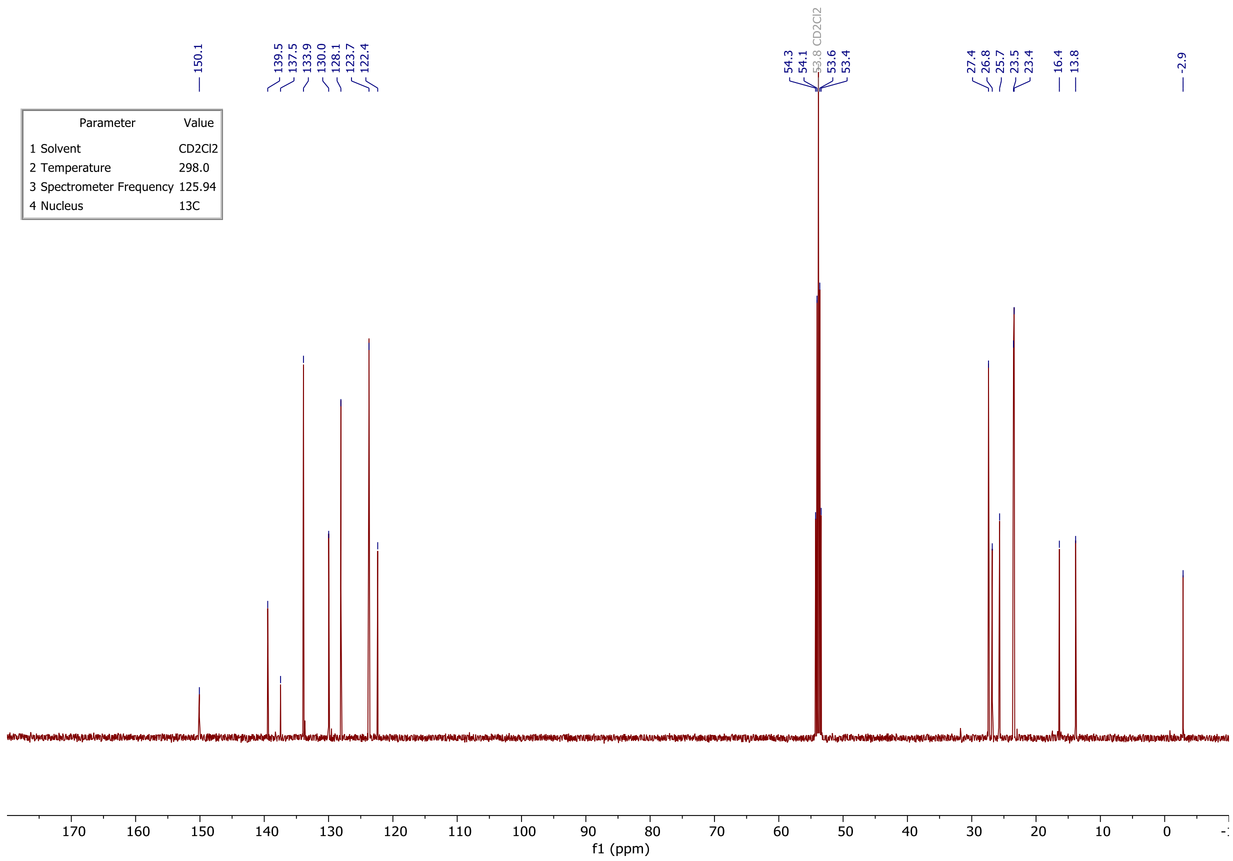

(2,6-diisopropylphenoxy)(methyl)(pentyl)(phenyl)silane 4e

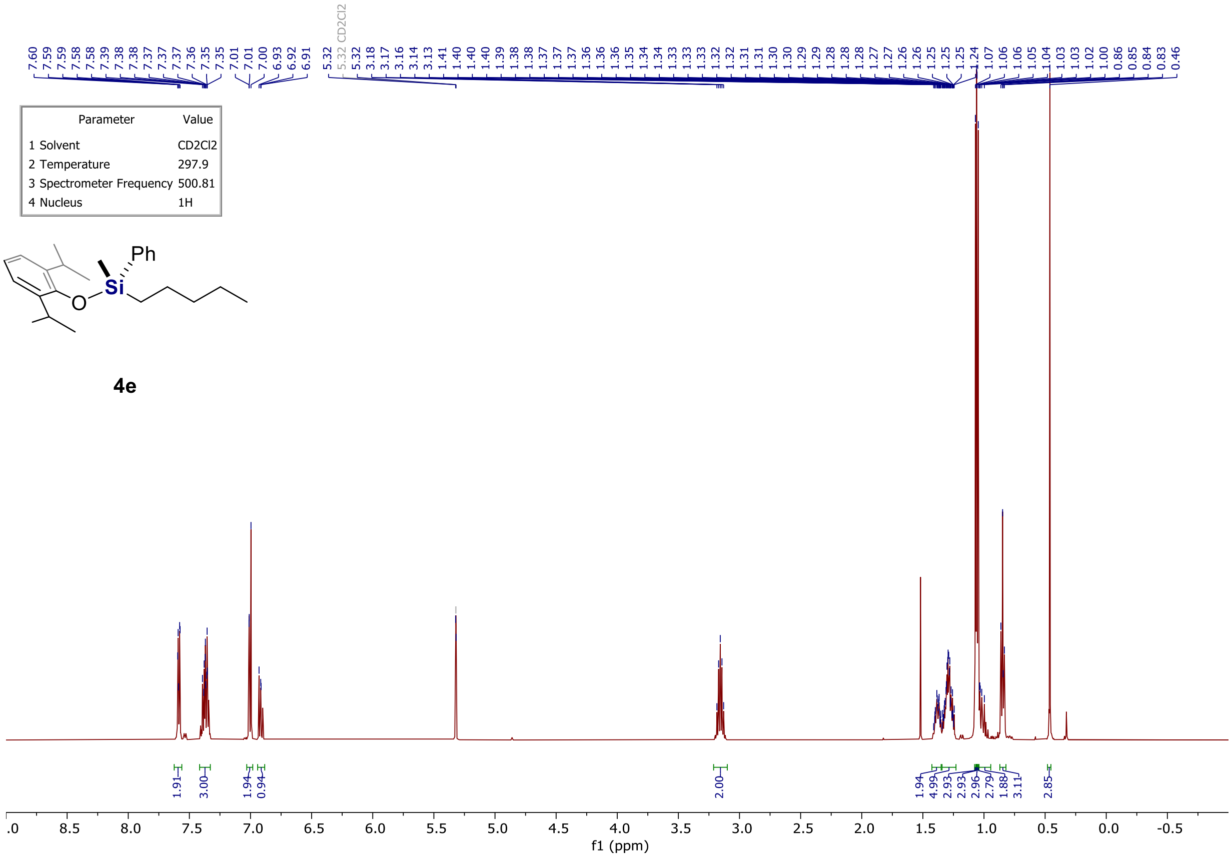

Parameter	Value
1 Solvent	CD2Cl2
2 Temperature	297.9
3 Spectrometer Frequency	500.81
4 Nucleus	1H

4e

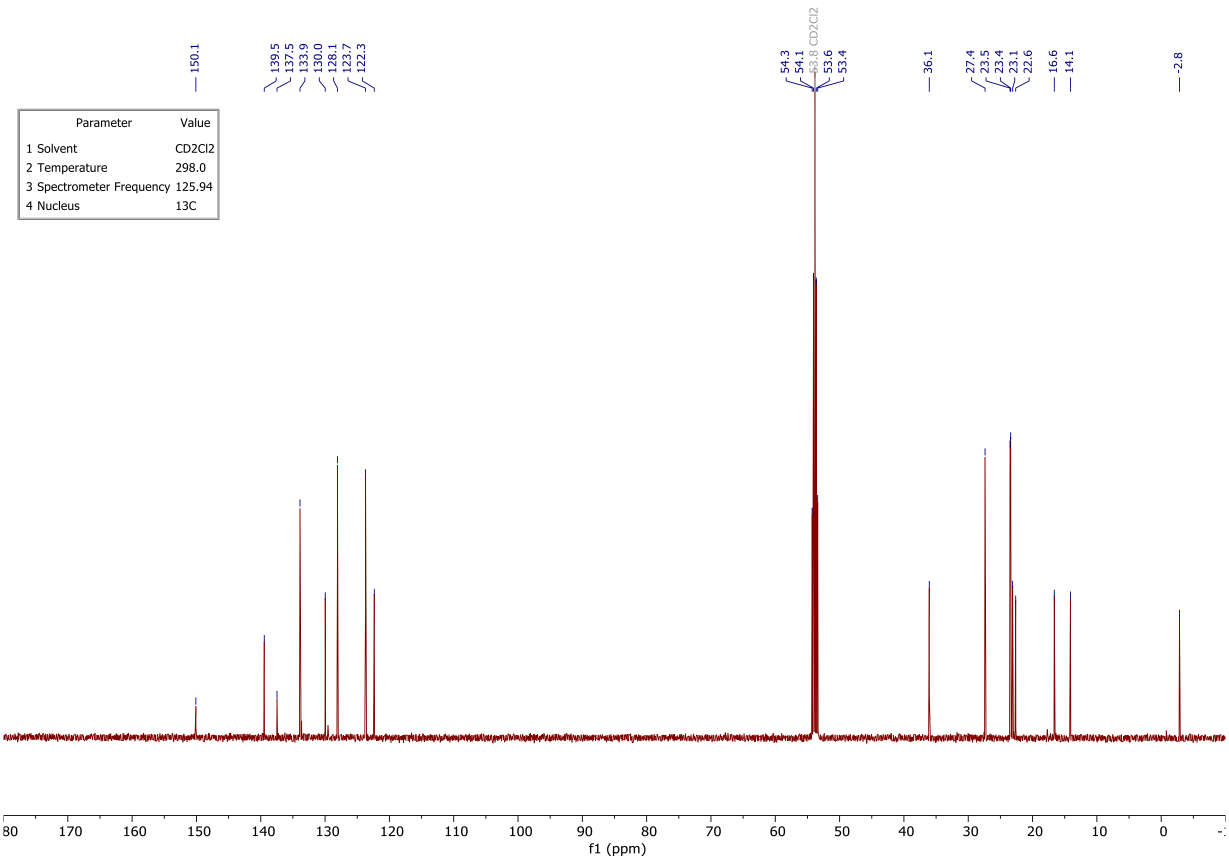

Parameter	Value
1 Solvent	CD2Cl2
2 Temperature	298.0
3 Spectrometer Frequency	125.94
4 Nucleus	13C

(2,6-diisopropylphenoxy)(heptyl)(methyl)(phenyl)silane 4f

Parameter	Value
1 Solvent	CD2Cl2
2 Temperature	297.9
3 Spectrometer Frequency	500.81
4 Nucleus	1H

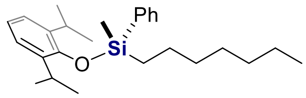

4f

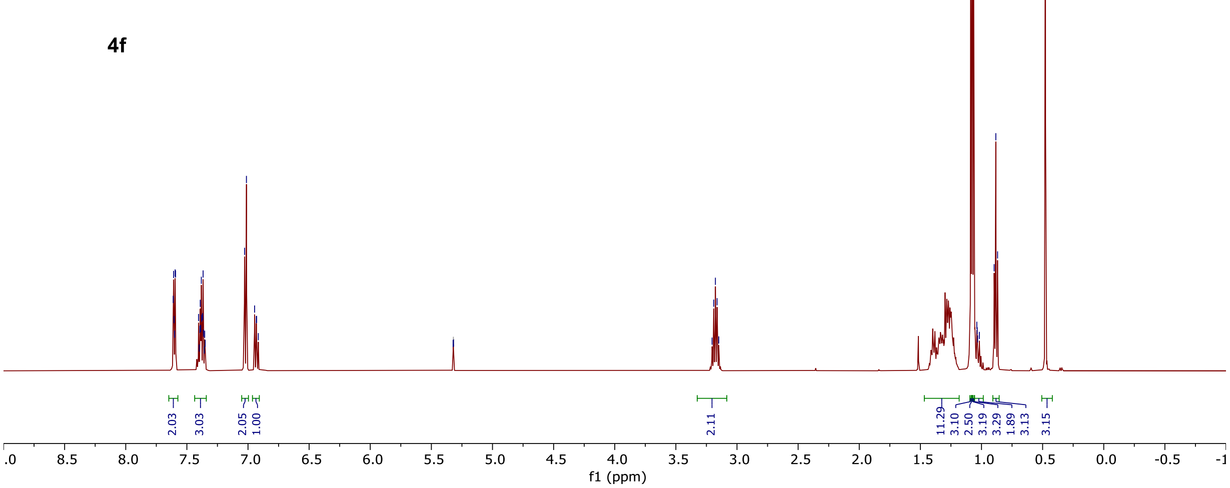

Parameter	Value
1 Solvent	CD2Cl2
2 Temperature	298.0
3 Spectrometer Frequency	125.94
4 Nucleus	13C

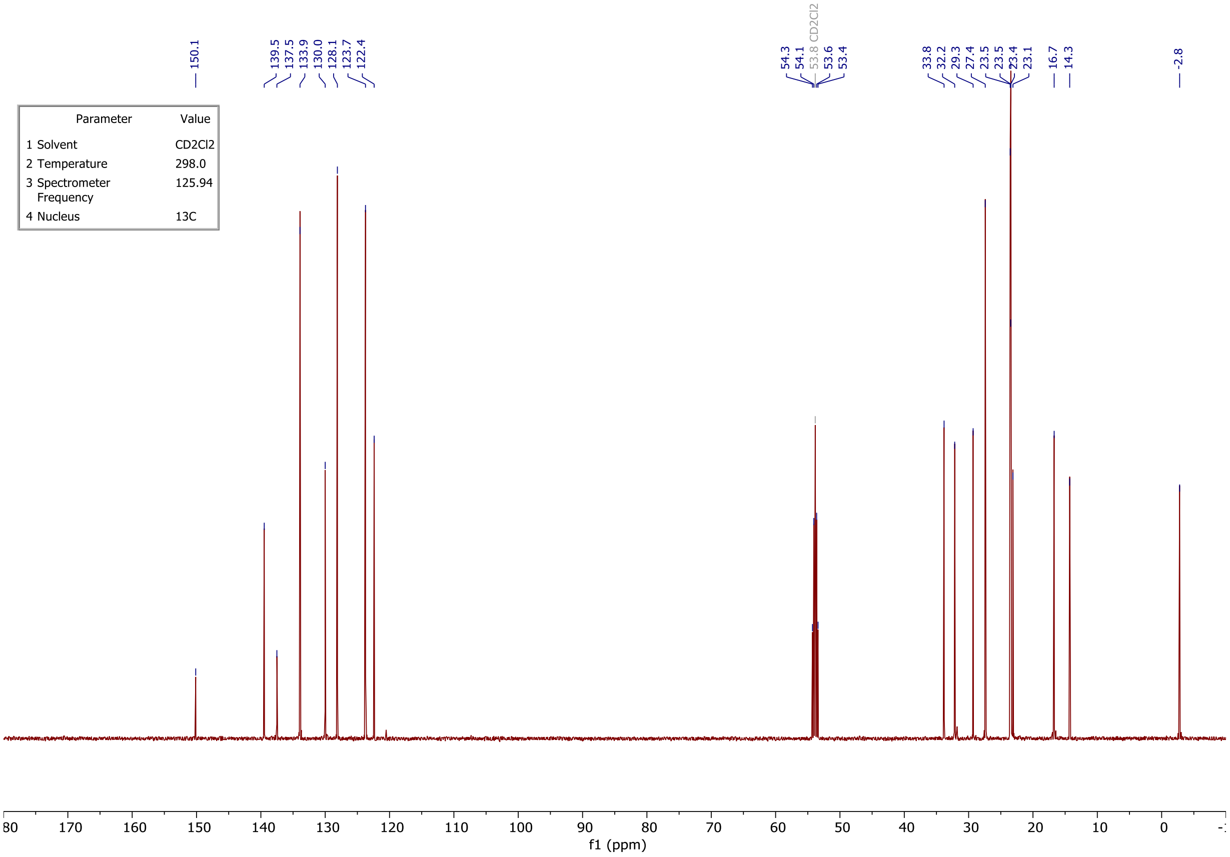

(2,6-dimethylphenoxy)(3-fluorobenzyl)(methyl)(2,2,4-trimethylpent-4-en-1-yl)silane 4g

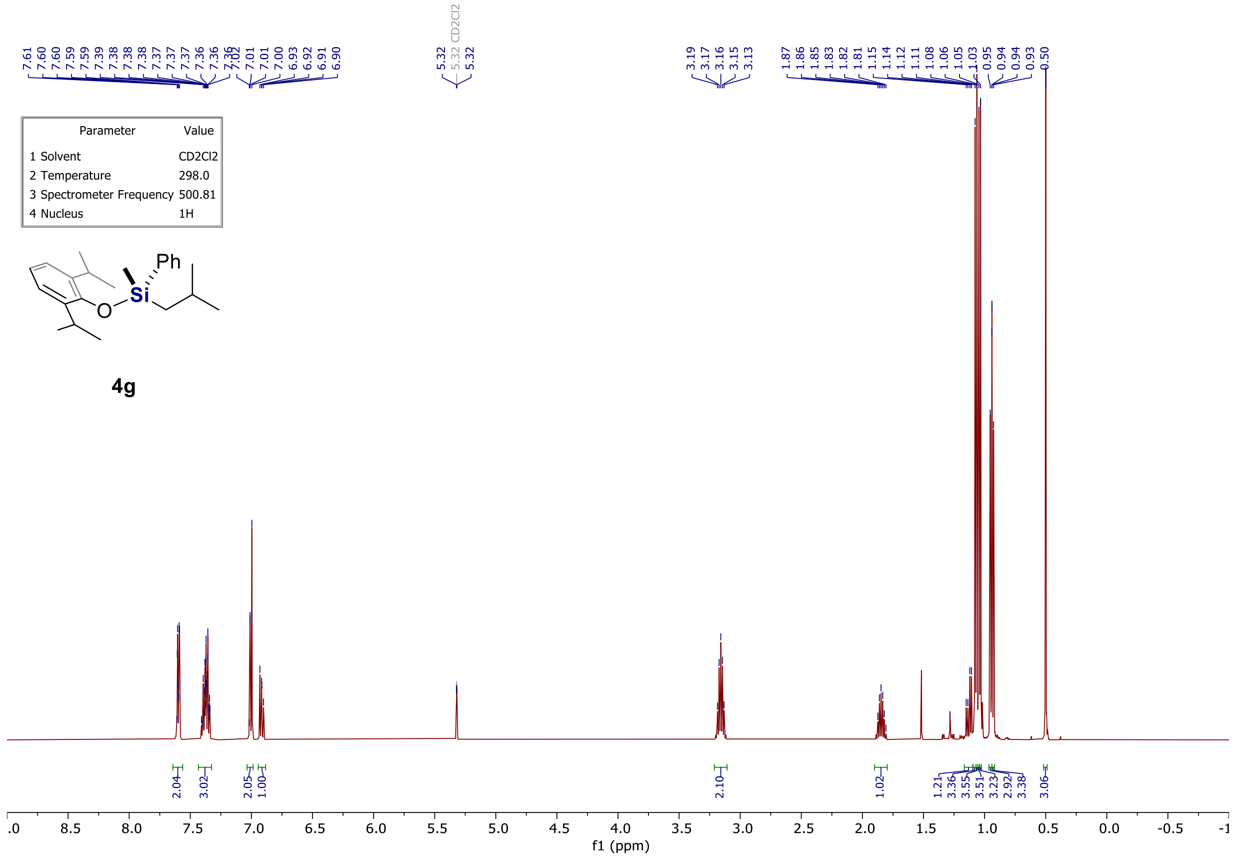

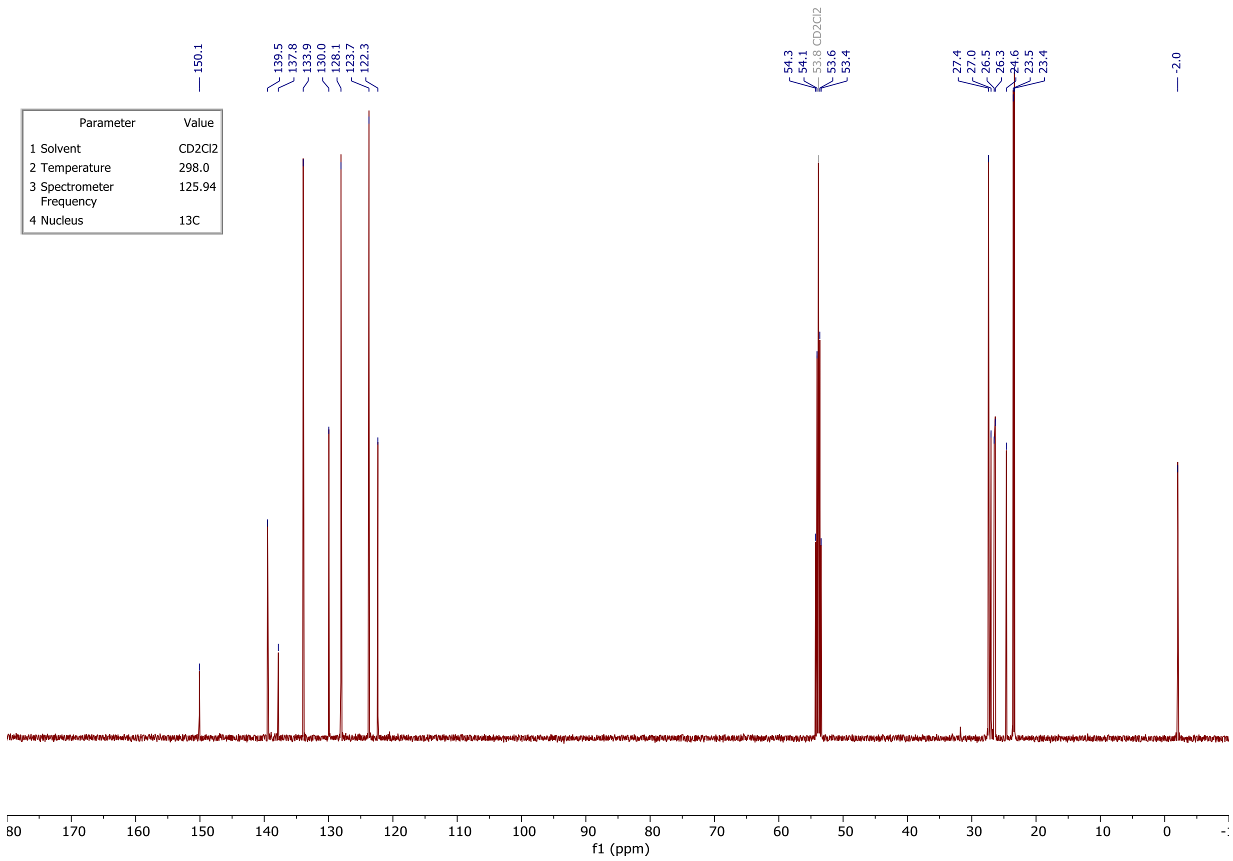

(2,6-dimethylphenoxy)(methyl)(2-methylbenzyl)(2,2,4-trimethylpent-4-en-1-yl)silane 4h

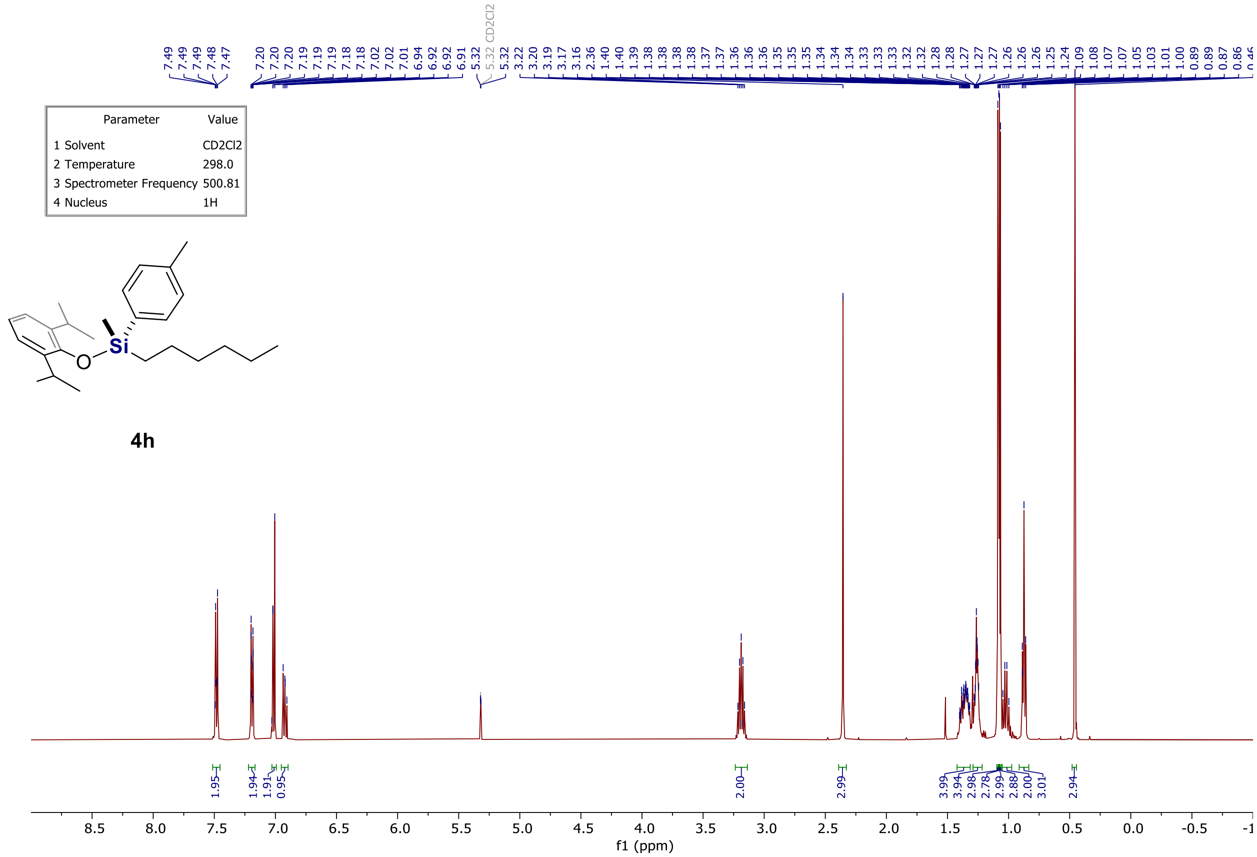

Parameter	Value
1 Solvent	CD2Cl2
2 Temperature	298.0
3 Spectrometer Frequency	500.81
4 Nucleus	1H

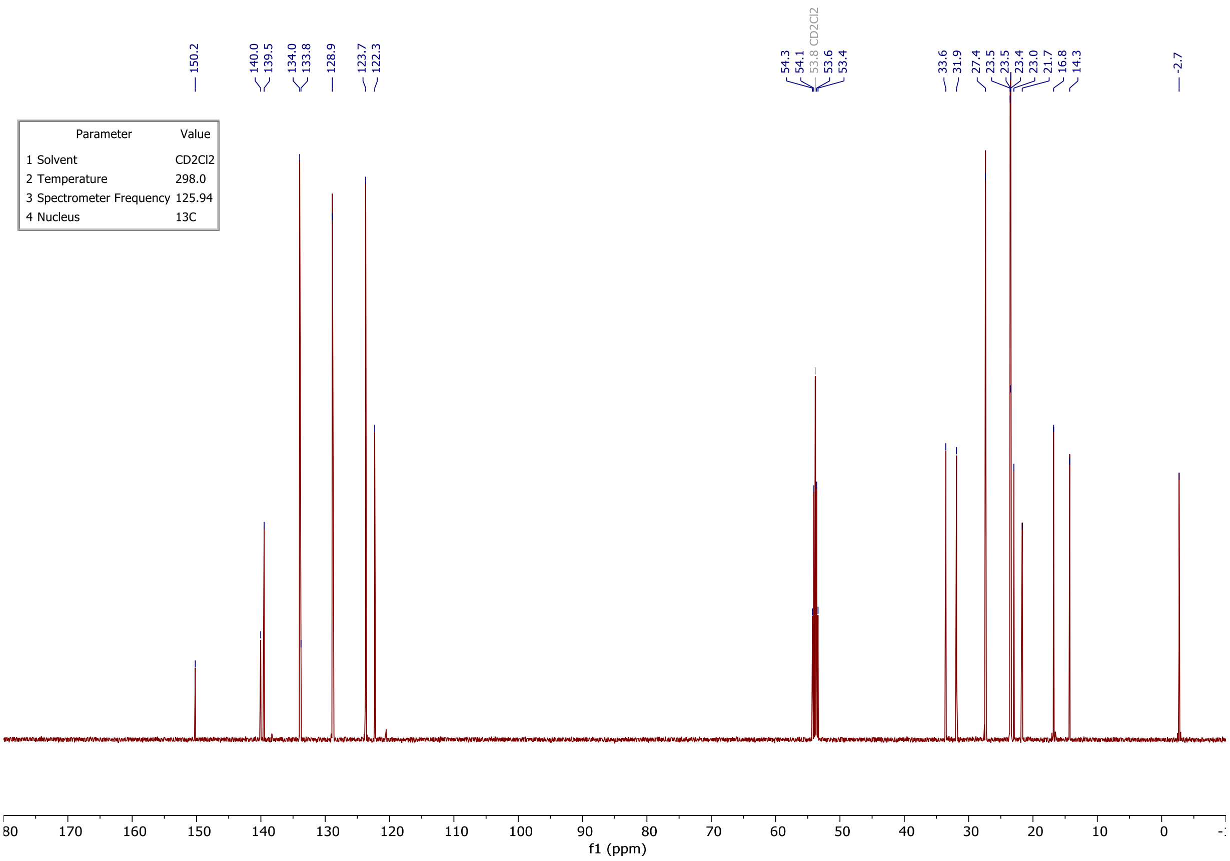

Parameter	Value
1 Solvent	CD2Cl2
2 Temperature	298.0
3 Spectrometer Frequency	125.94
4 Nucleus	13C

(2,6-dimethylphenoxy)(2-fluorobenzyl)(methyl)(2,2,4-trimethylpent-4-en-1-yl)silane 4i

Parameter	Value
1 Solvent	CD2Cl2
2 Temperature	297.9
3 Spectrometer Frequency	500.81
4 Nucleus	1H

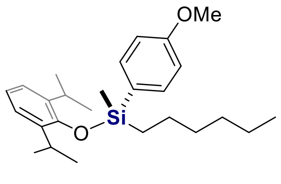

4i

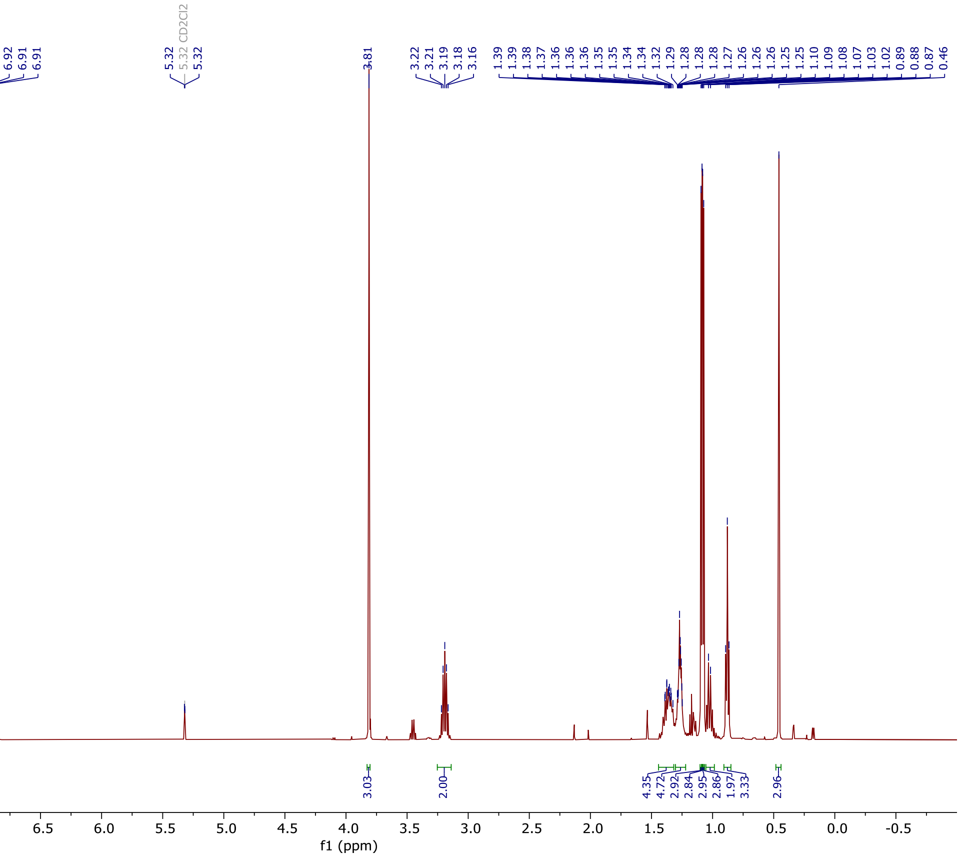

Parameter	Value
1 Solvent	CD2Cl2
2 Temperature	298.0
3 Spectrometer Frequency	125.94
4 Nucleus	13C

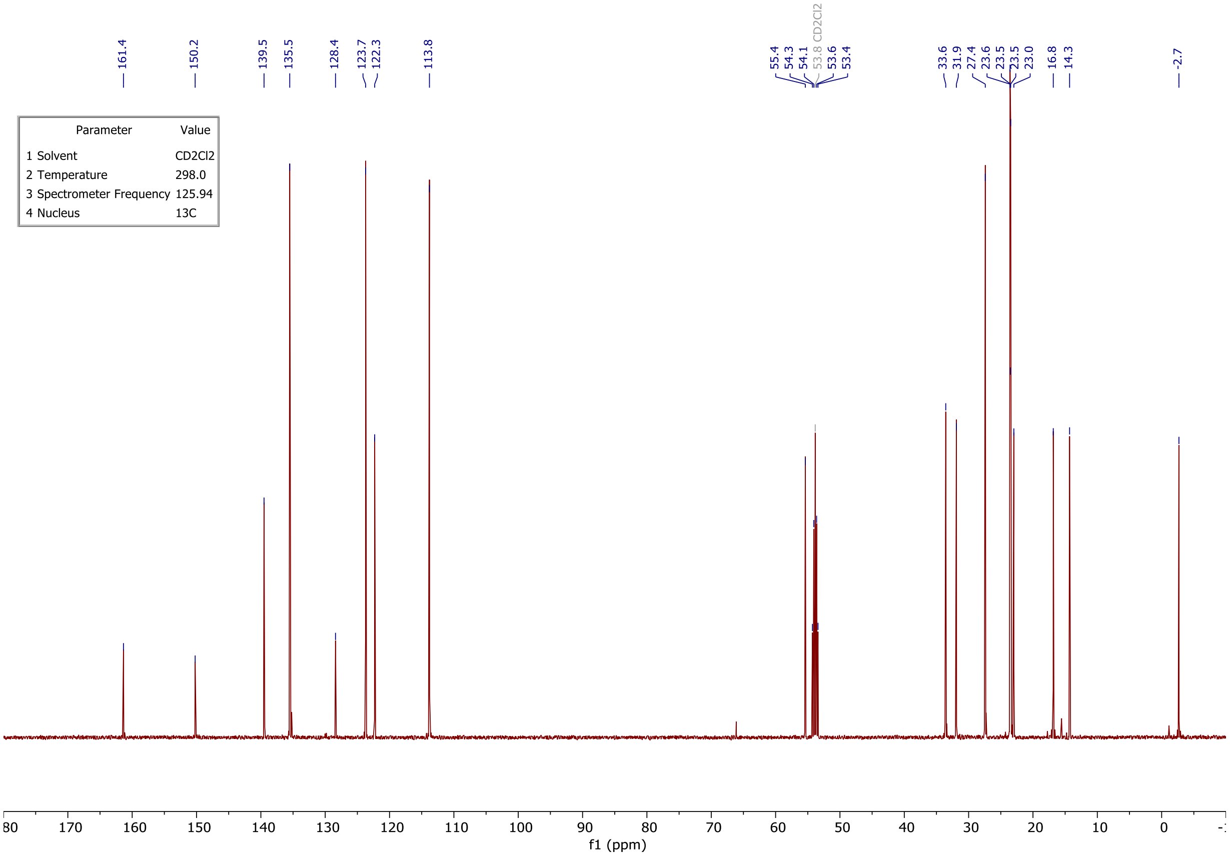

(3,4-dimethylbenzyl)(2,6-dimethylphenoxy)(methyl)(2,2,4-trimethylpent-4-en-1-yl)silane 4j

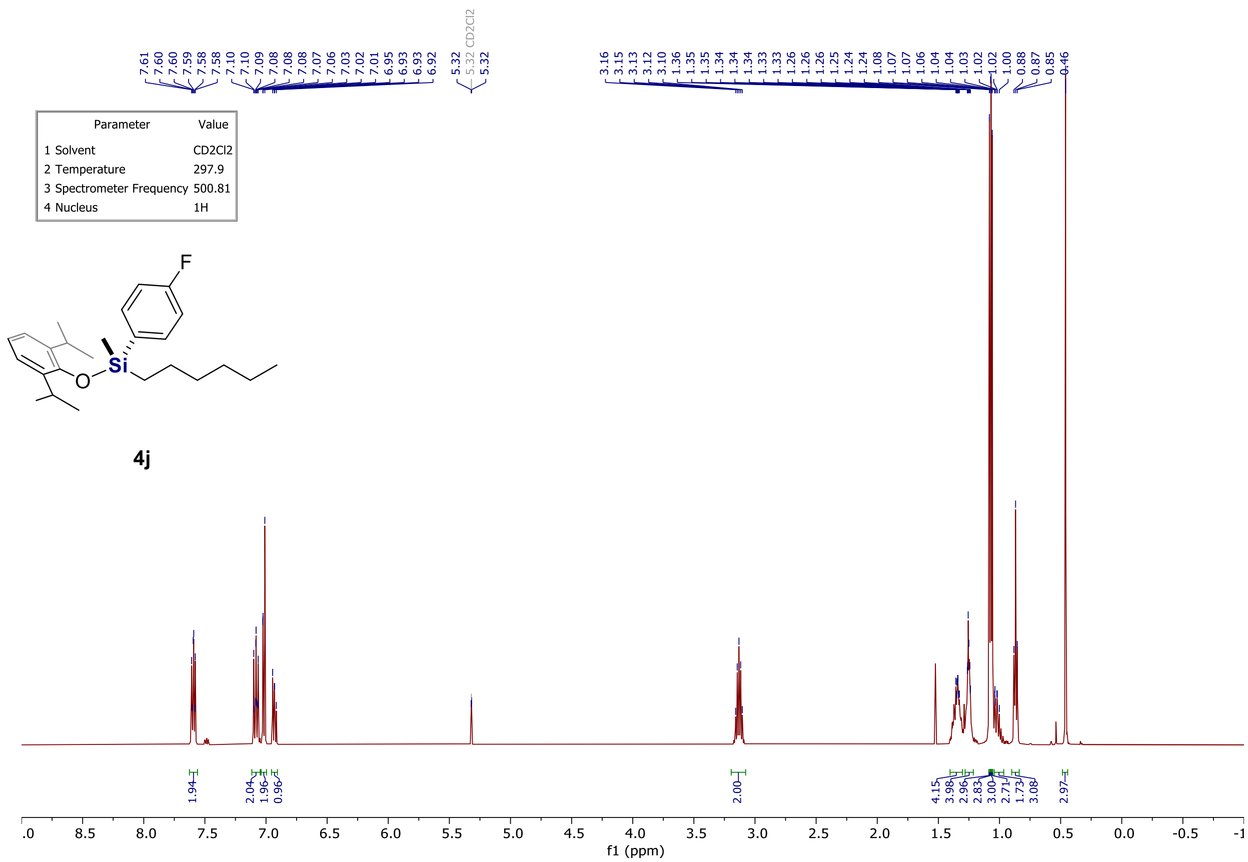

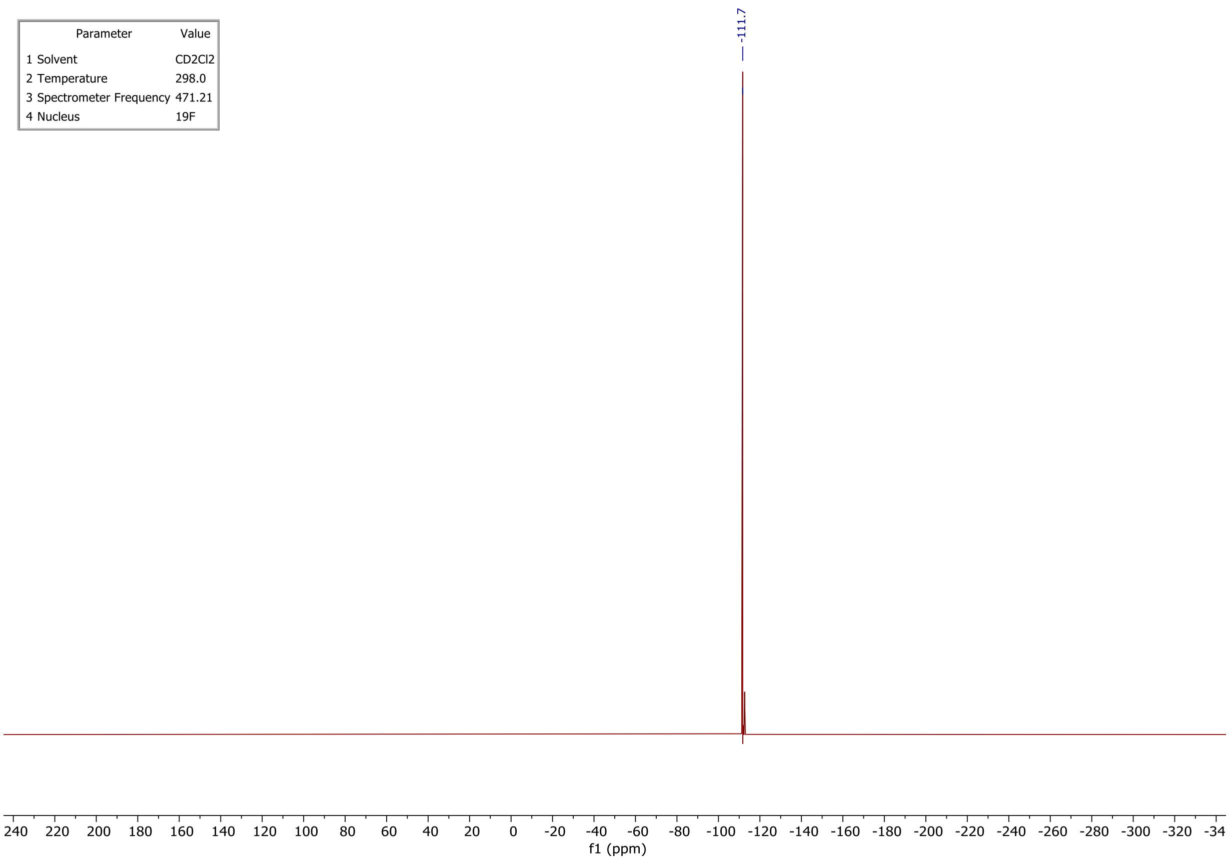

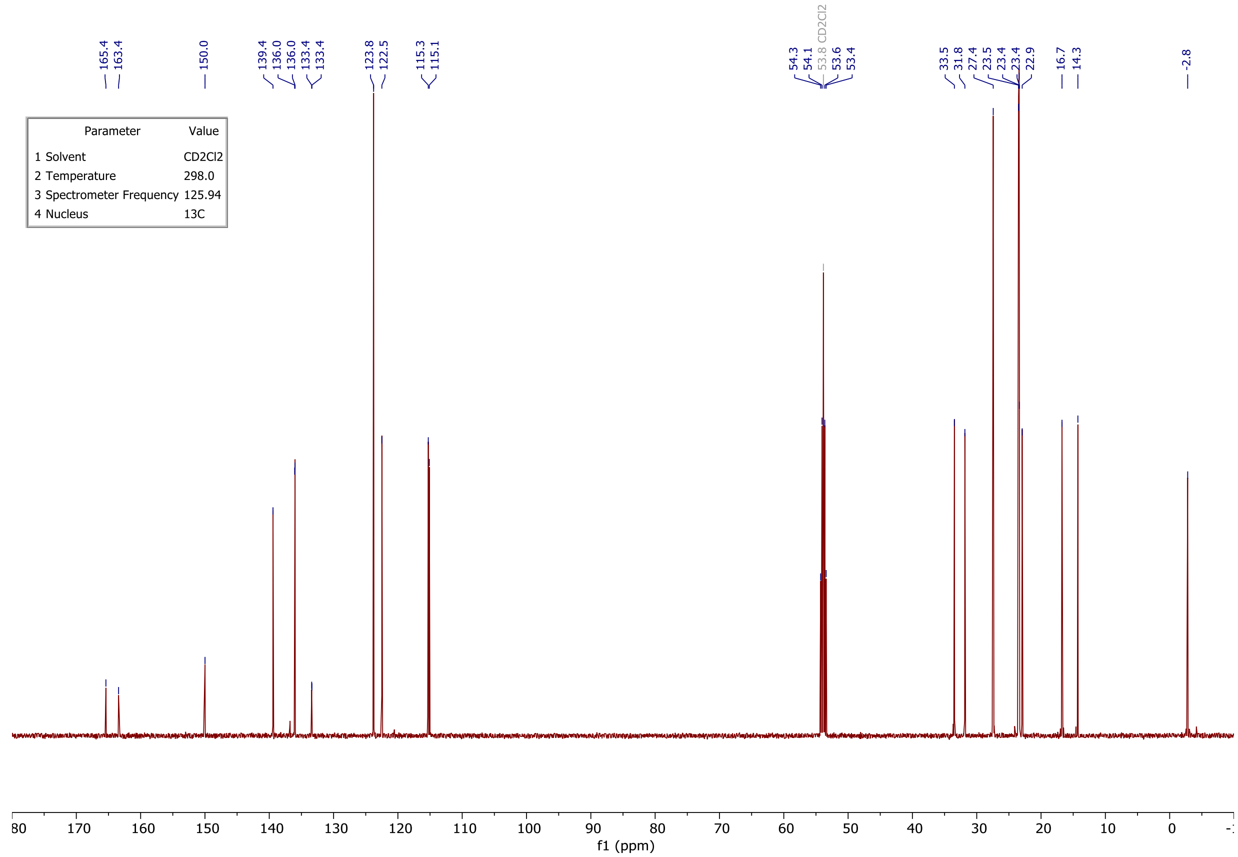

(2,6-dimethylphenoxy)(methyl)(naphthalen-2-ylmethyl)(2,2,4-trimethylpent-4-en-1-yl)silane 4k

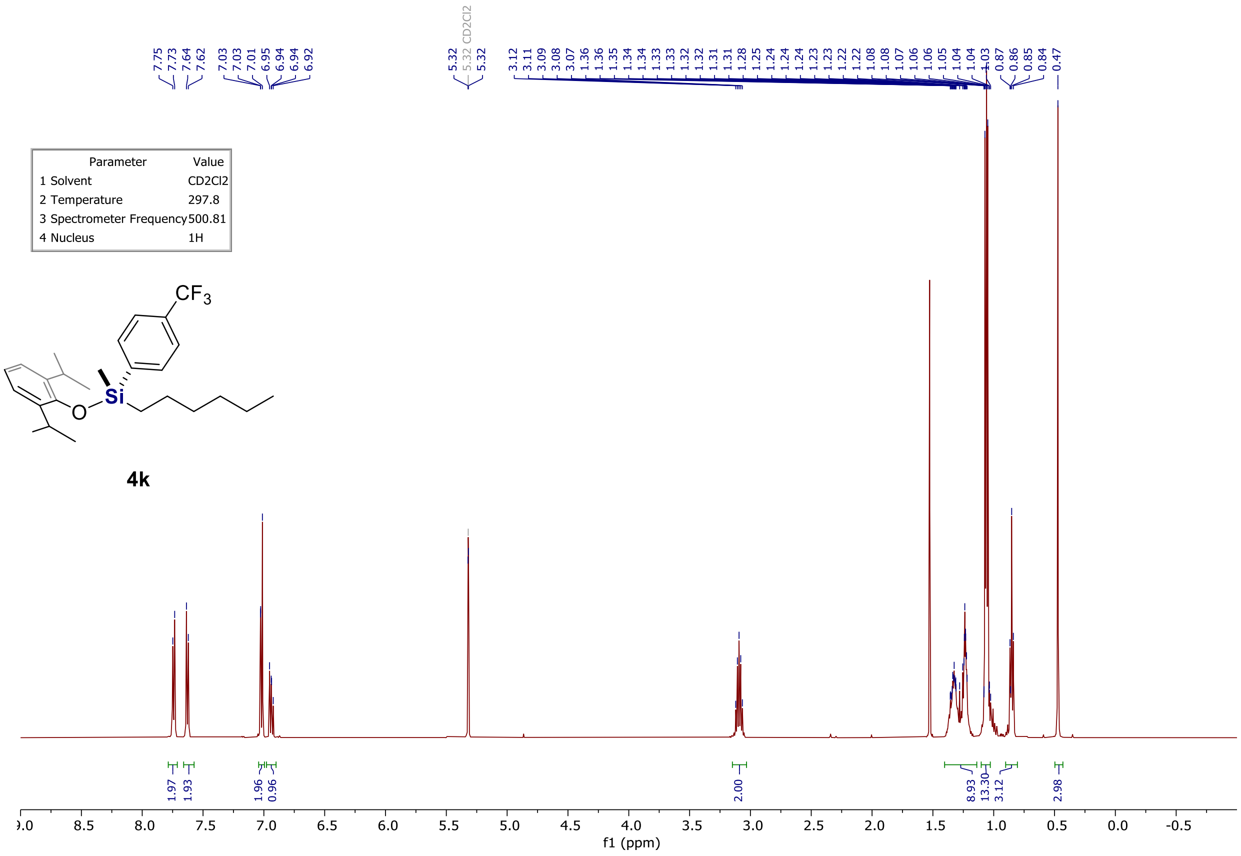

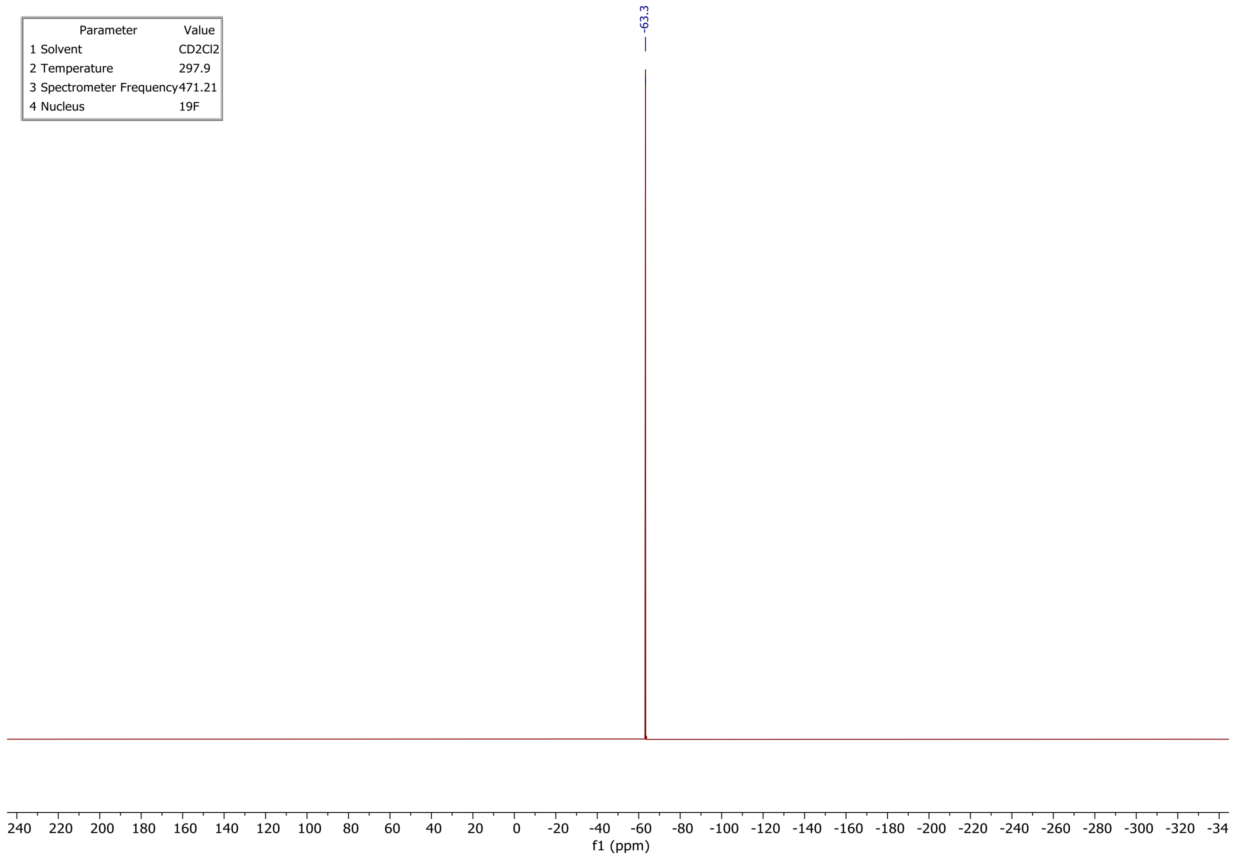
-63.3
Parameter Value
1 Solvent CD2Cl2
2 Temperature 297.9
3 Spectrometer Frequency 471.21
4 Nucleus 19F
f1 (ppm)

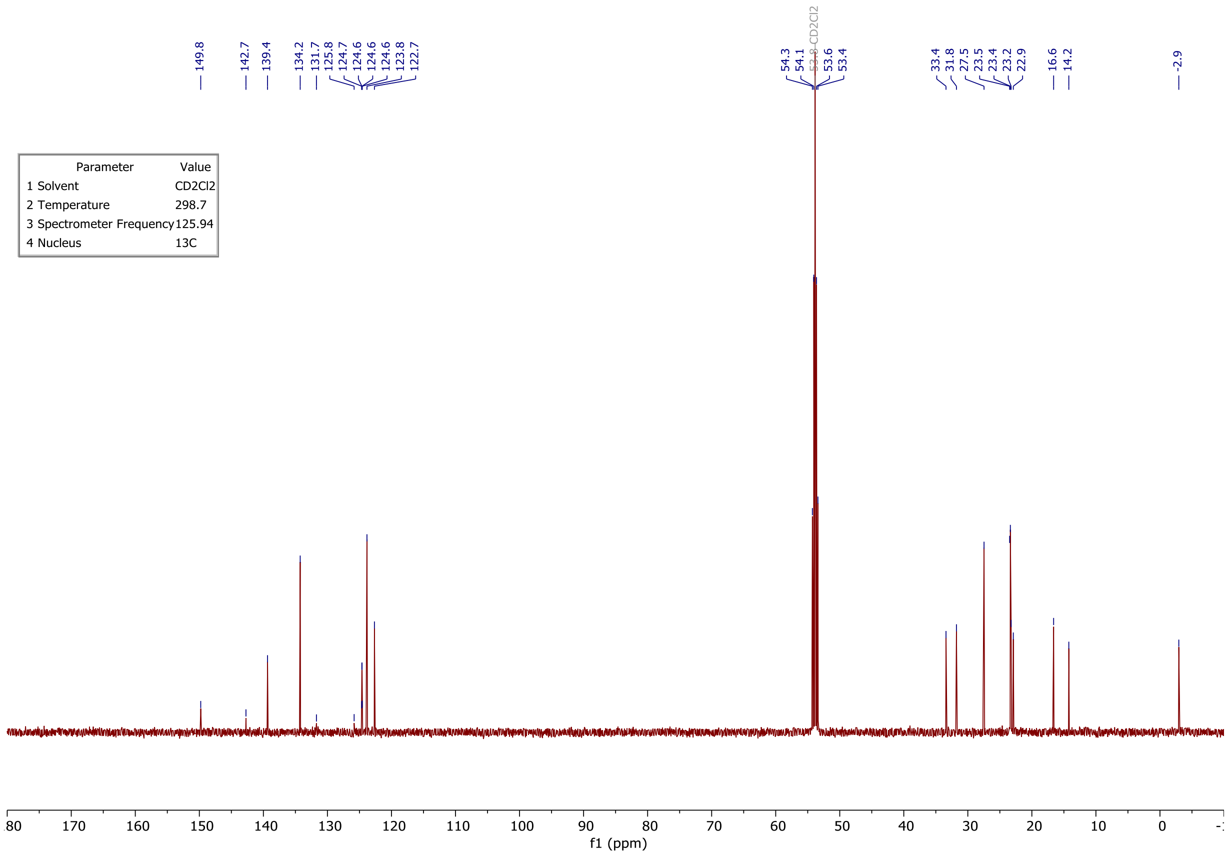
149.8
142.7
139.4
134.2
131.7
125.8
124.7
124.6
124.6
124.6
123.8
122.7
54.3
54.1
53.8 CD2Cl2
53.6
53.4
33.4
31.8
27.5
23.5
23.4
23.2
22.9
16.6
14.2
-2.9
Parameter Value
1 Solvent CD2Cl2
2 Temperature 298.7
3 Spectrometer Frequency 125.94
4 Nucleus 13C
f1 (ppm)

(S)-(2,6-dimethylphenoxy)(methyl)(phenyl)(2,2,4-trimethylpent-4-en-1-yl)silane 4l

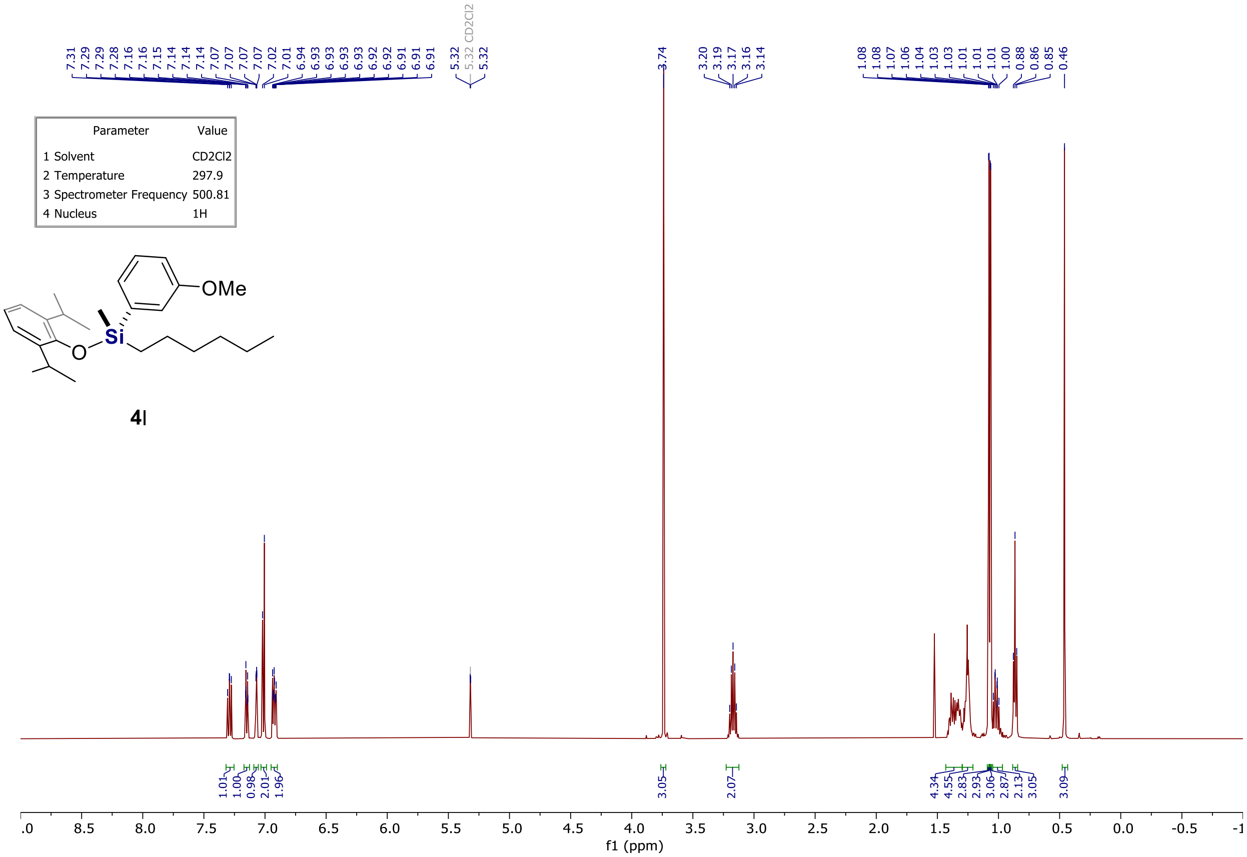

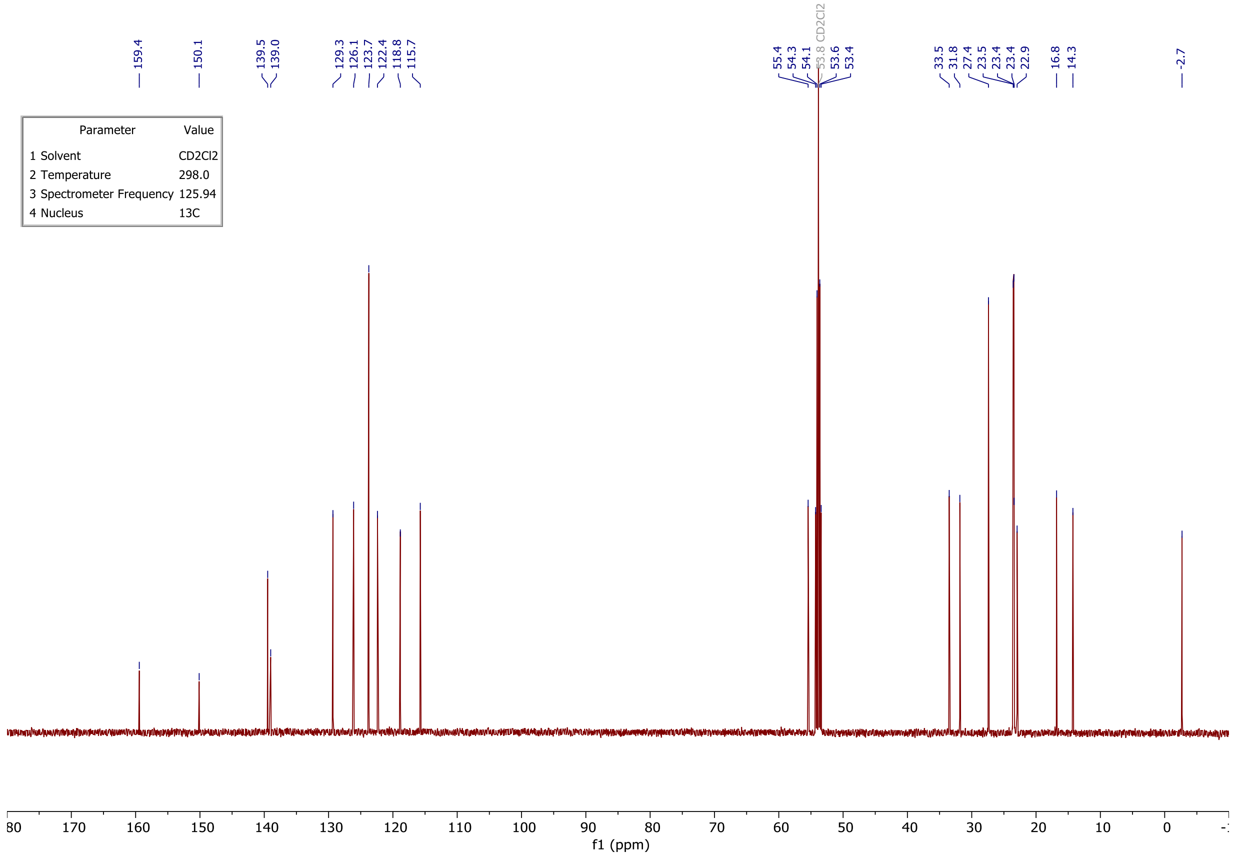

(S)-(2,6-dimethylphenoxy)(methyl)(p-tolyl)(2,2,4-trimethylpent-4-en-1-yl)silane 4m

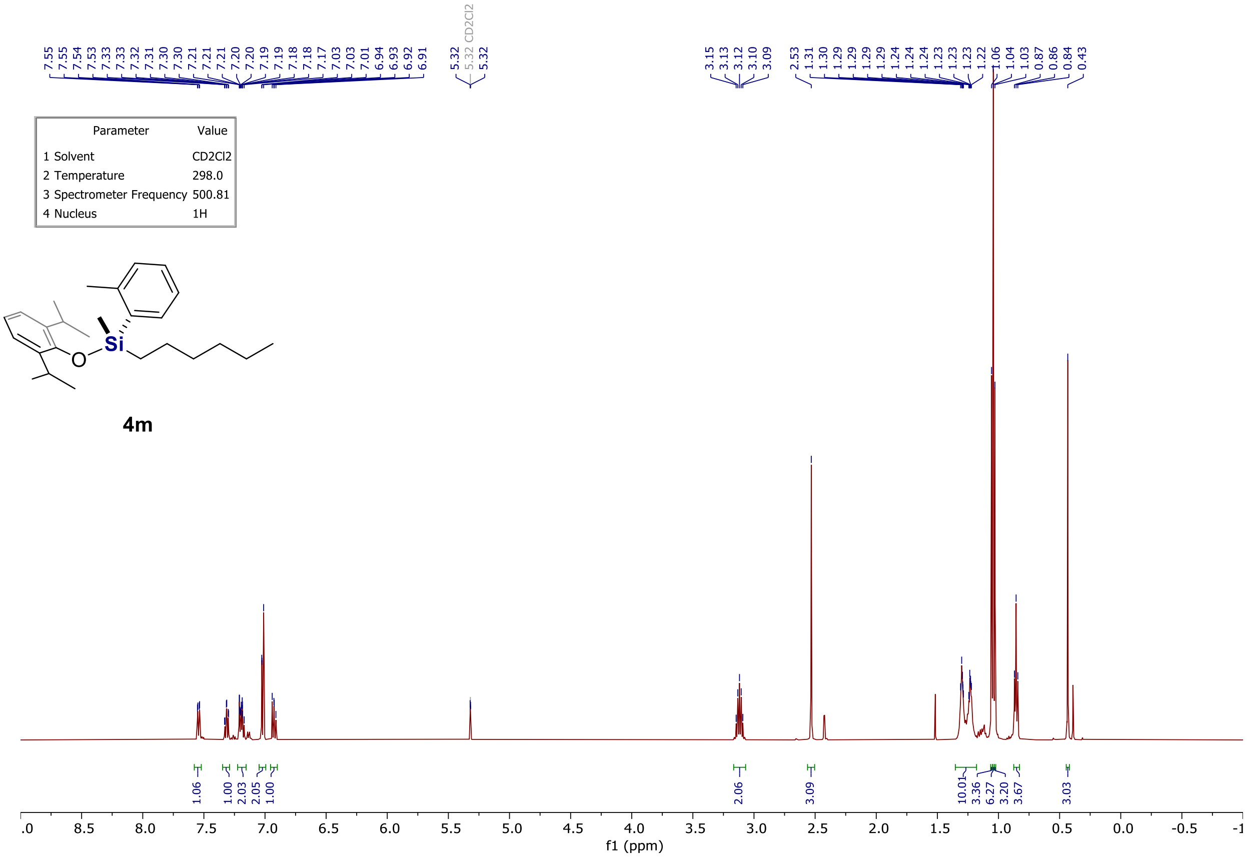

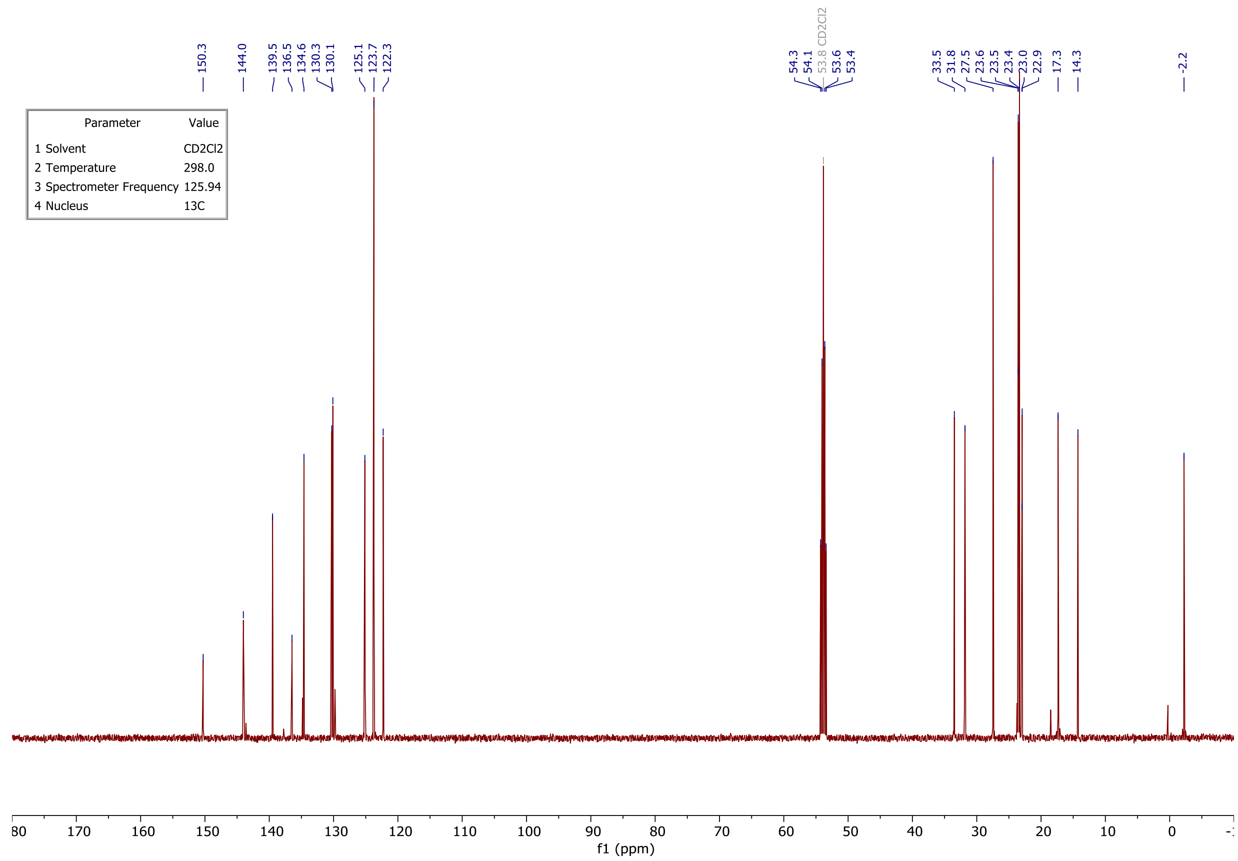

(2,6-dimethylphenoxy)(4-fluorophenyl)(methyl)(2,2,4-trimethylpent-4-en-1-yl)silane 4n

Parameter	Value
1 Solvent	CD2Cl2
2 Temperature	297.7
3 Spectrometer Frequency	500.81
4 Nucleus	1H

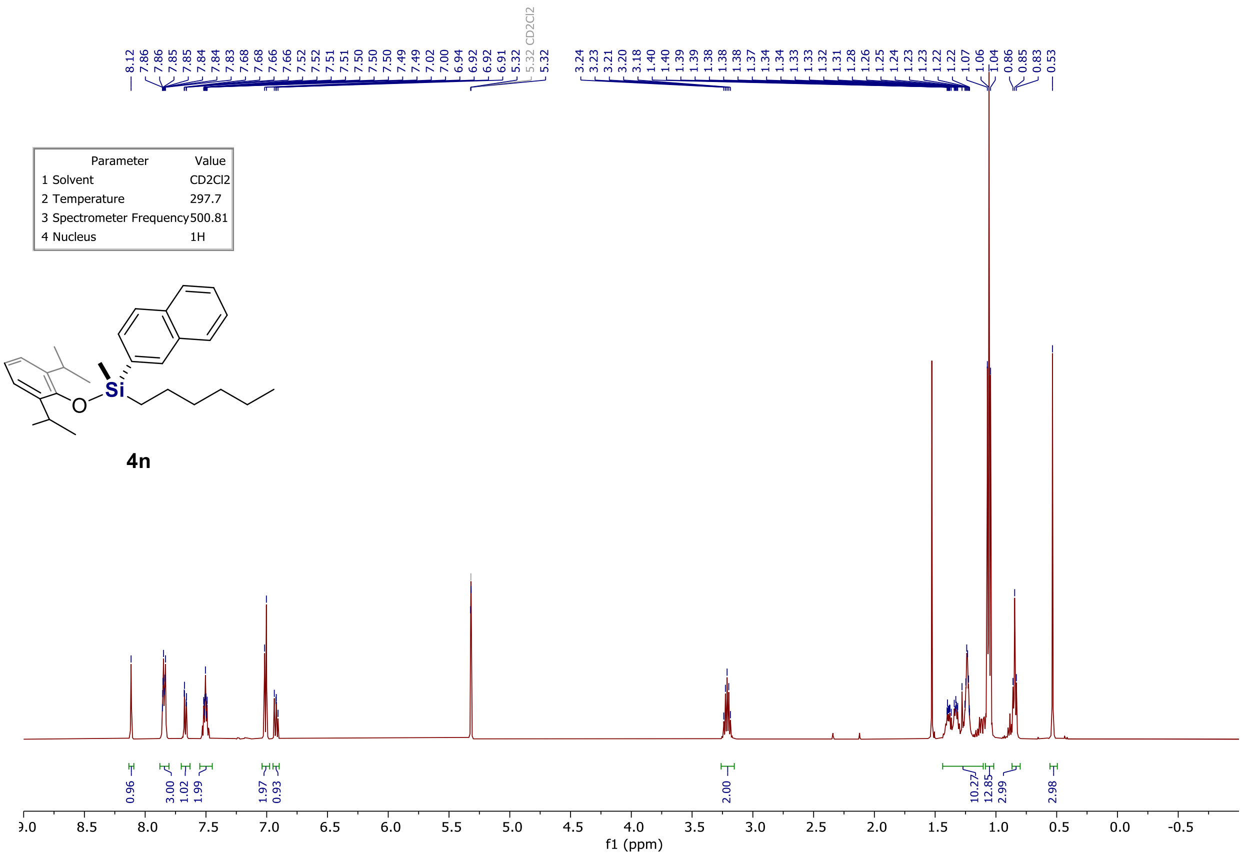

4n

Parameter	Value
1 Solvent	CD2Cl2
2 Temperature	298.6
3 Spectrometer Frequency	125.94
4 Nucleus	13C

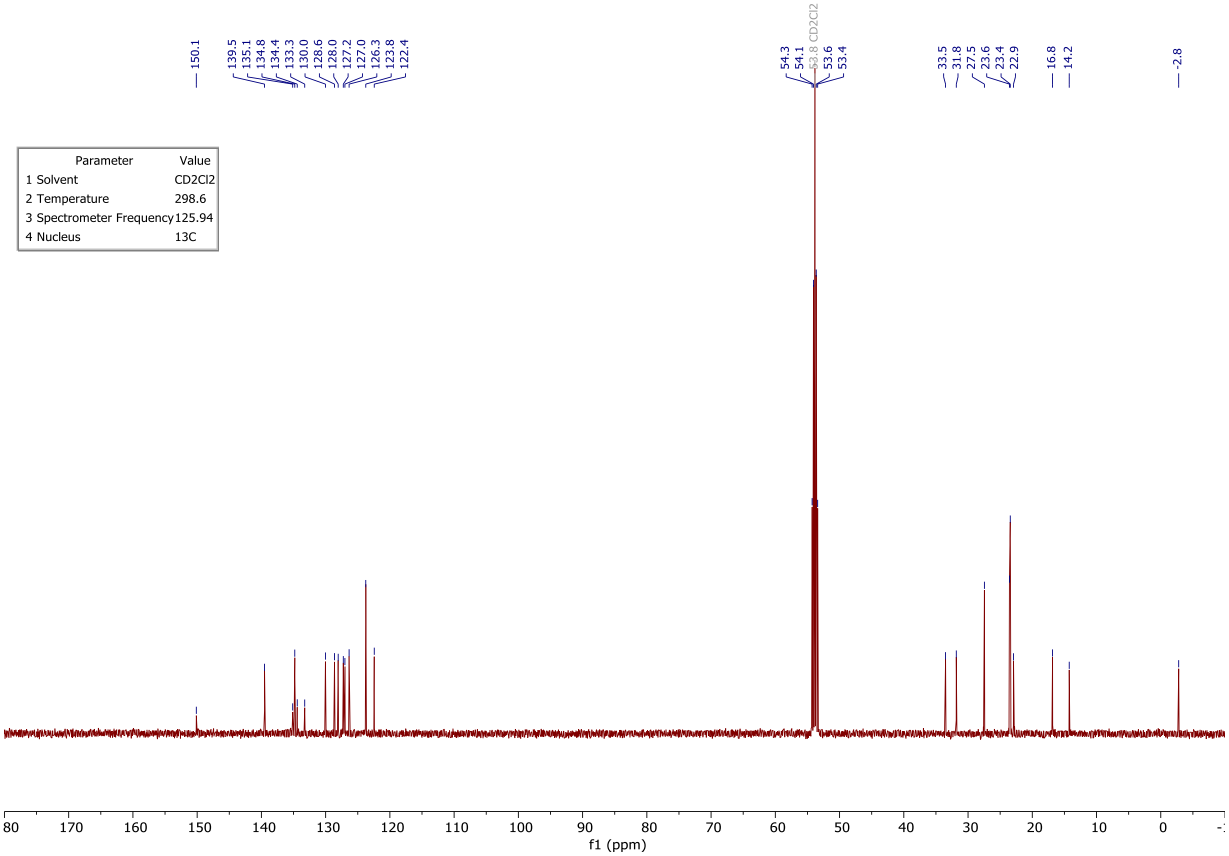

(2,6-dimethylphenoxy)(methyl)(thiophen-2-yl)(2,2,4-trimethylpent-4-en-1-yl)silane 4o

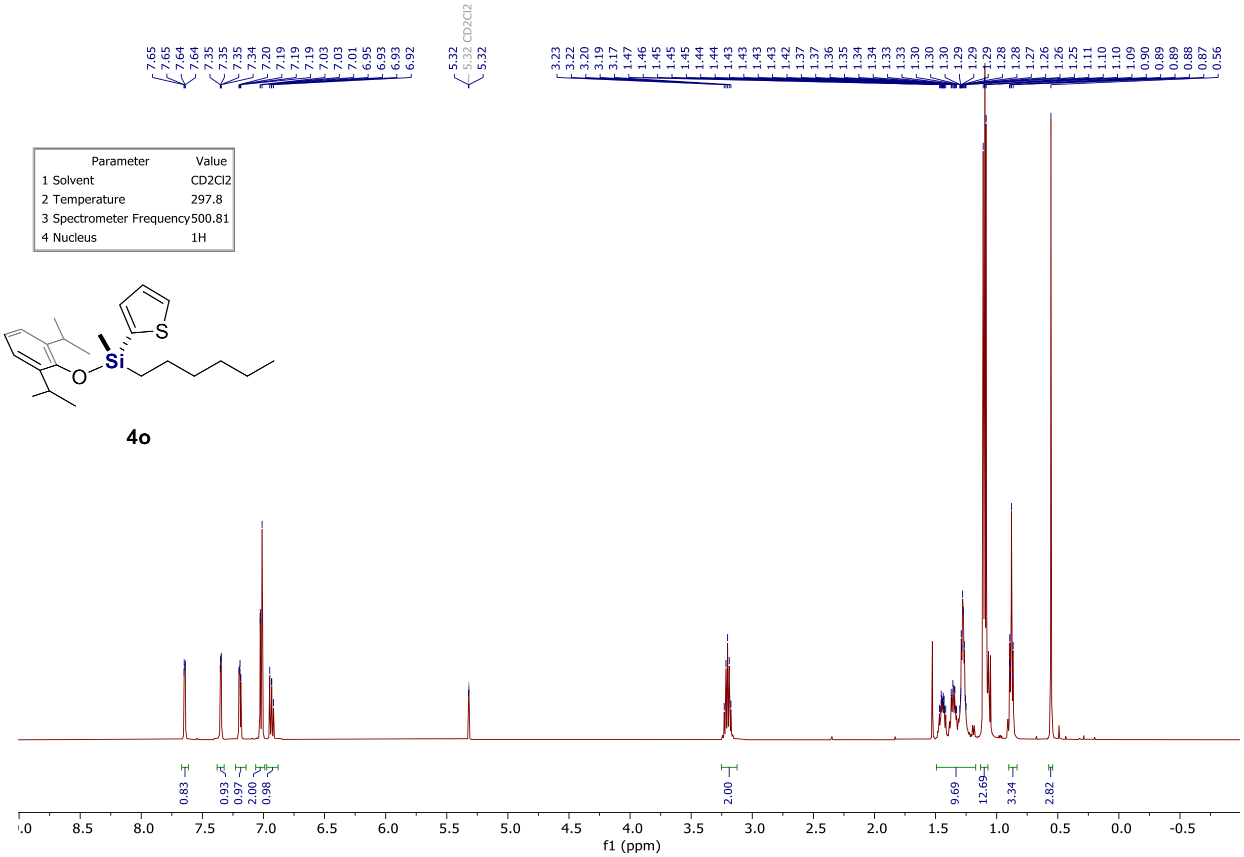

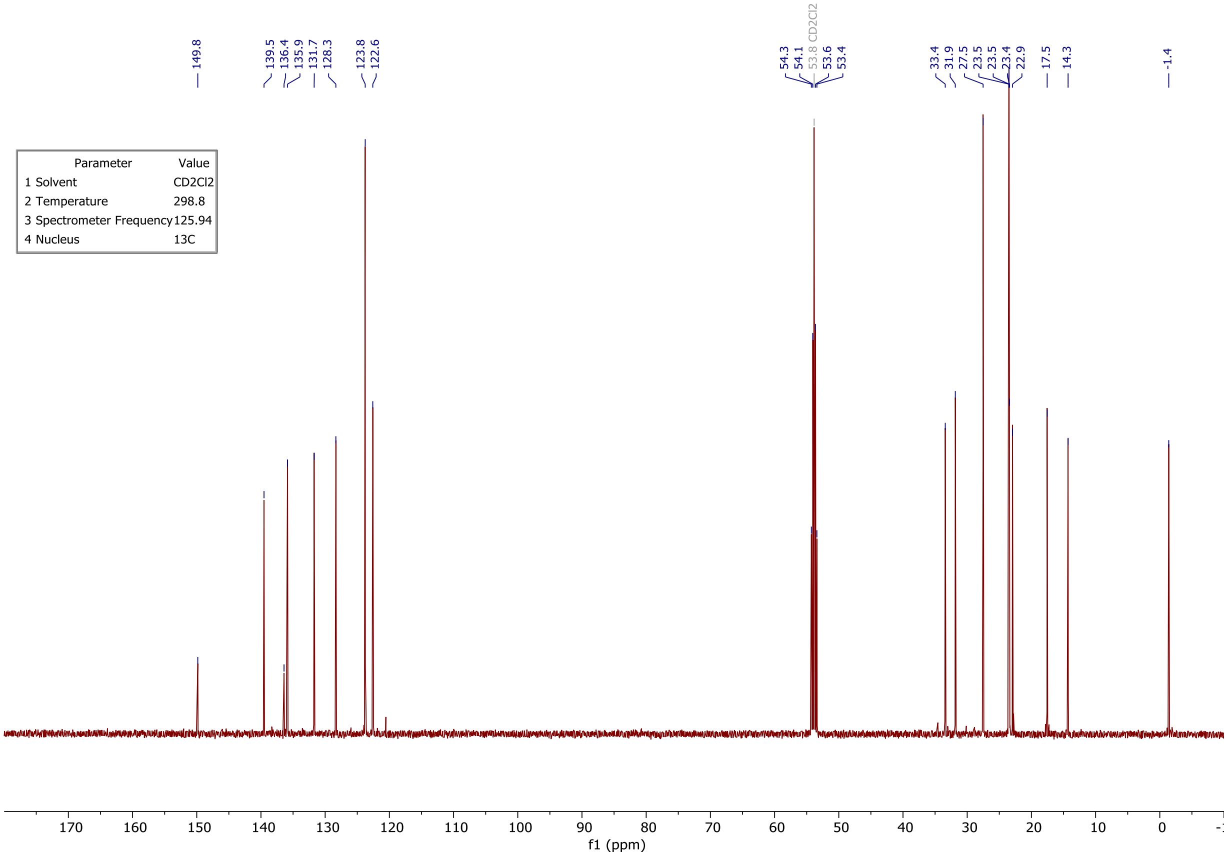

(2,6-dimethylphenoxy)(methyl)(propyl)(2,2,4-trimethylpent-4-en-1-yl)silane 4p

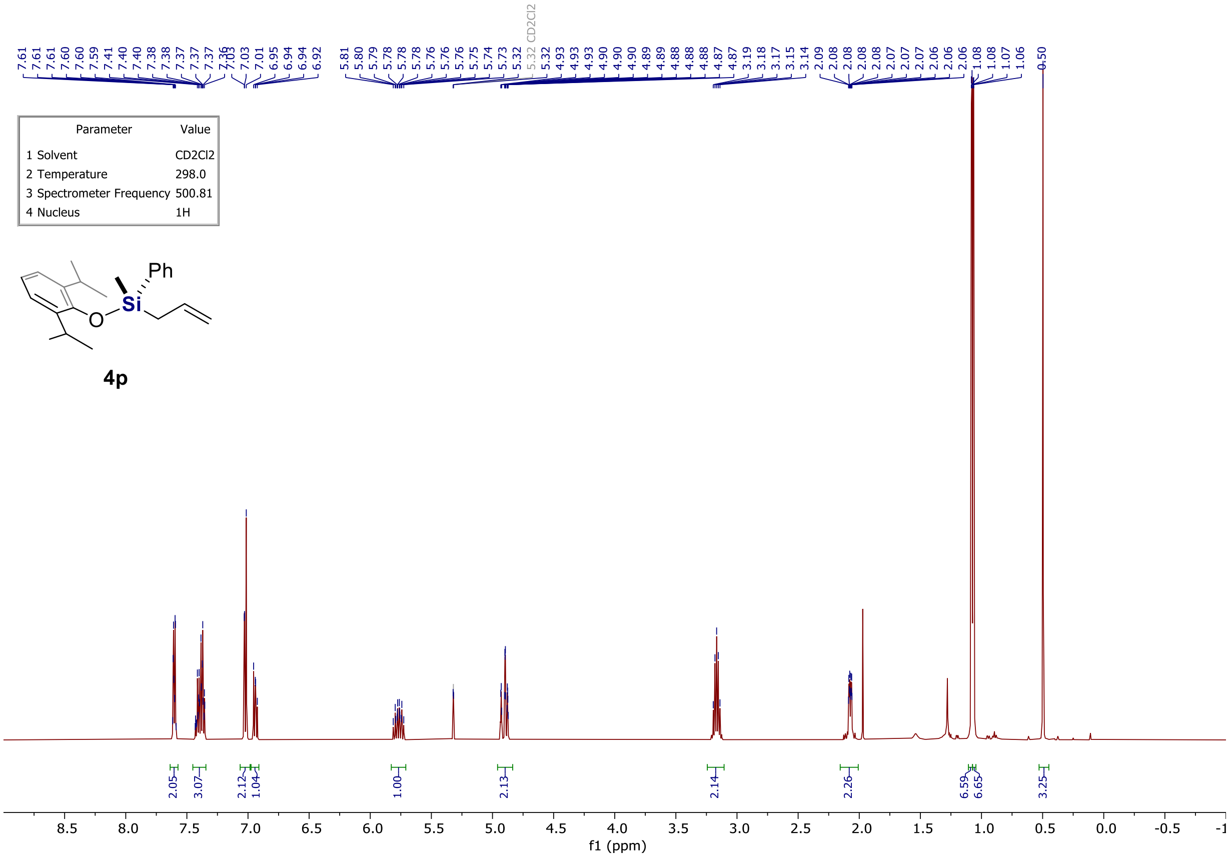

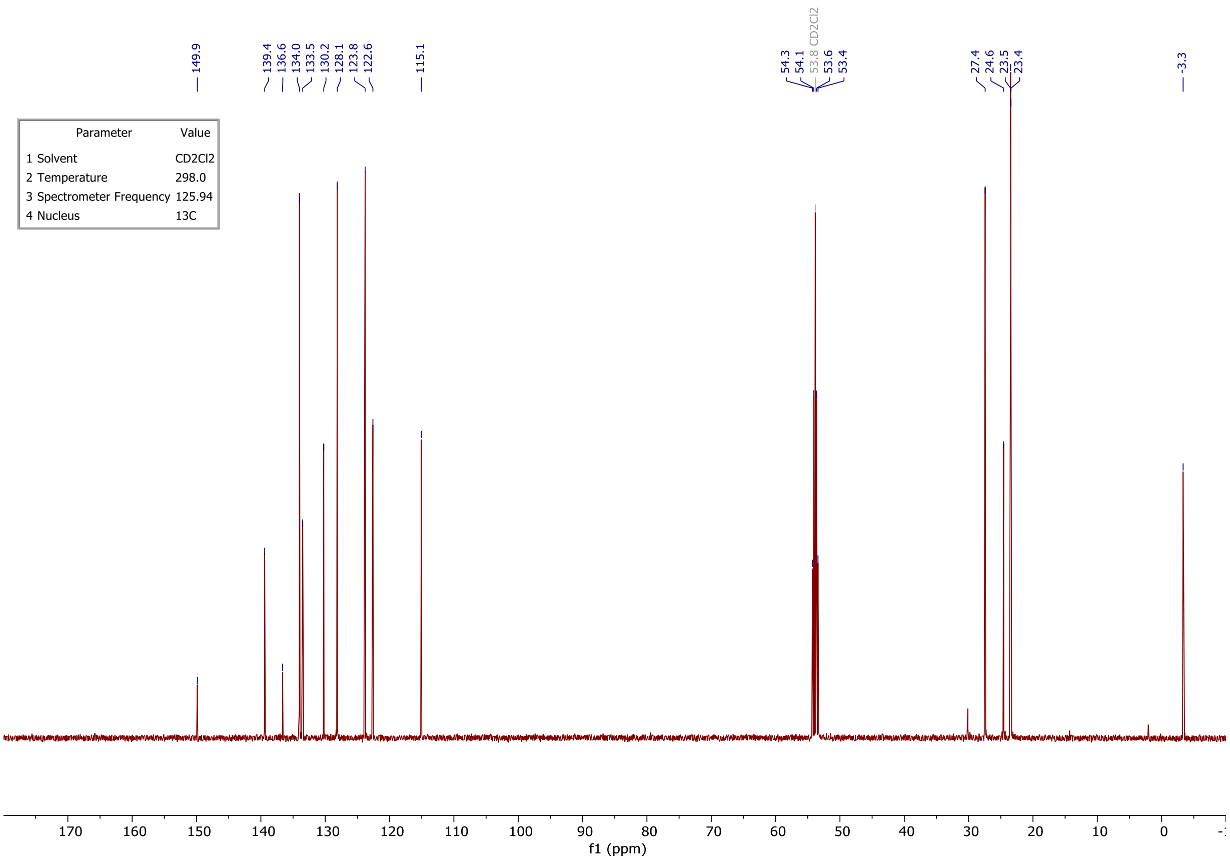

(2,6-dimethoxyphenoxy)(ethyl)(methyl)(2,2,4-trimethylpent-4-en-1-yl)silane 4q

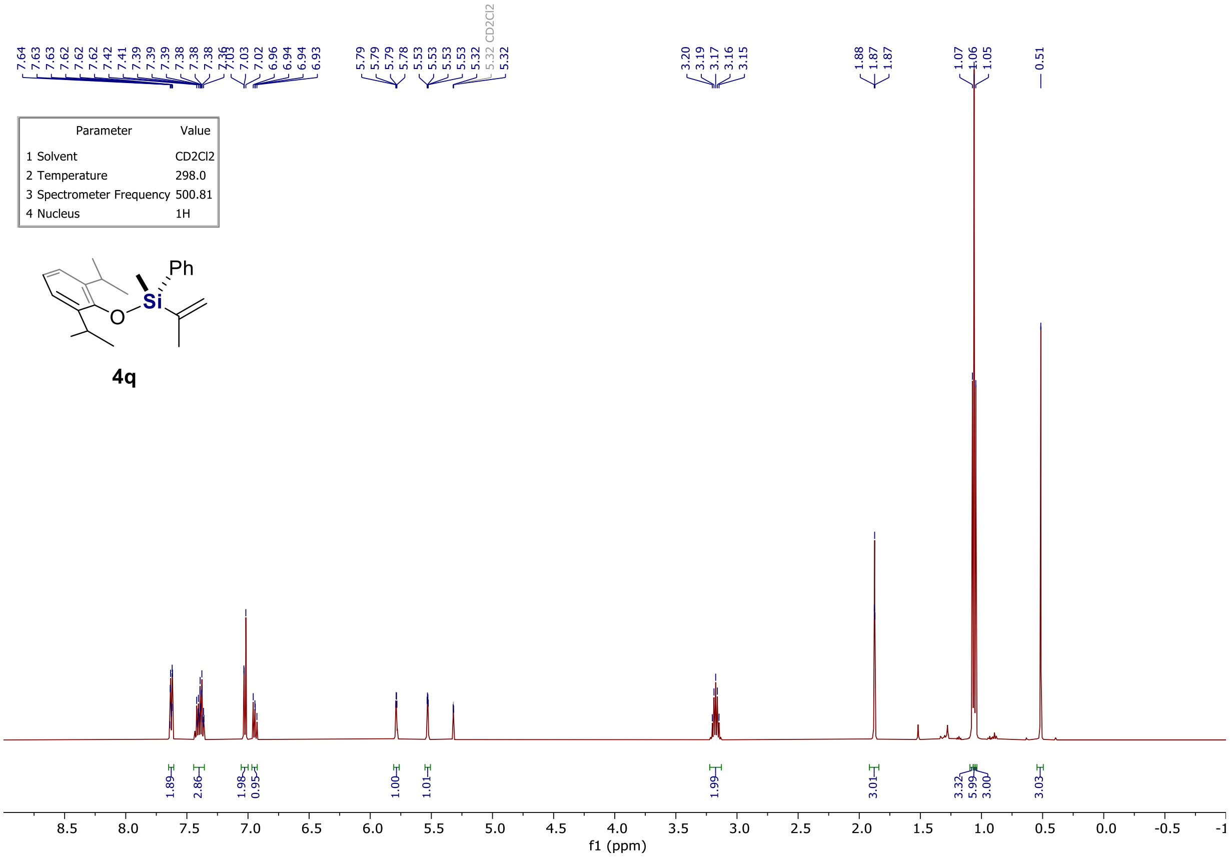

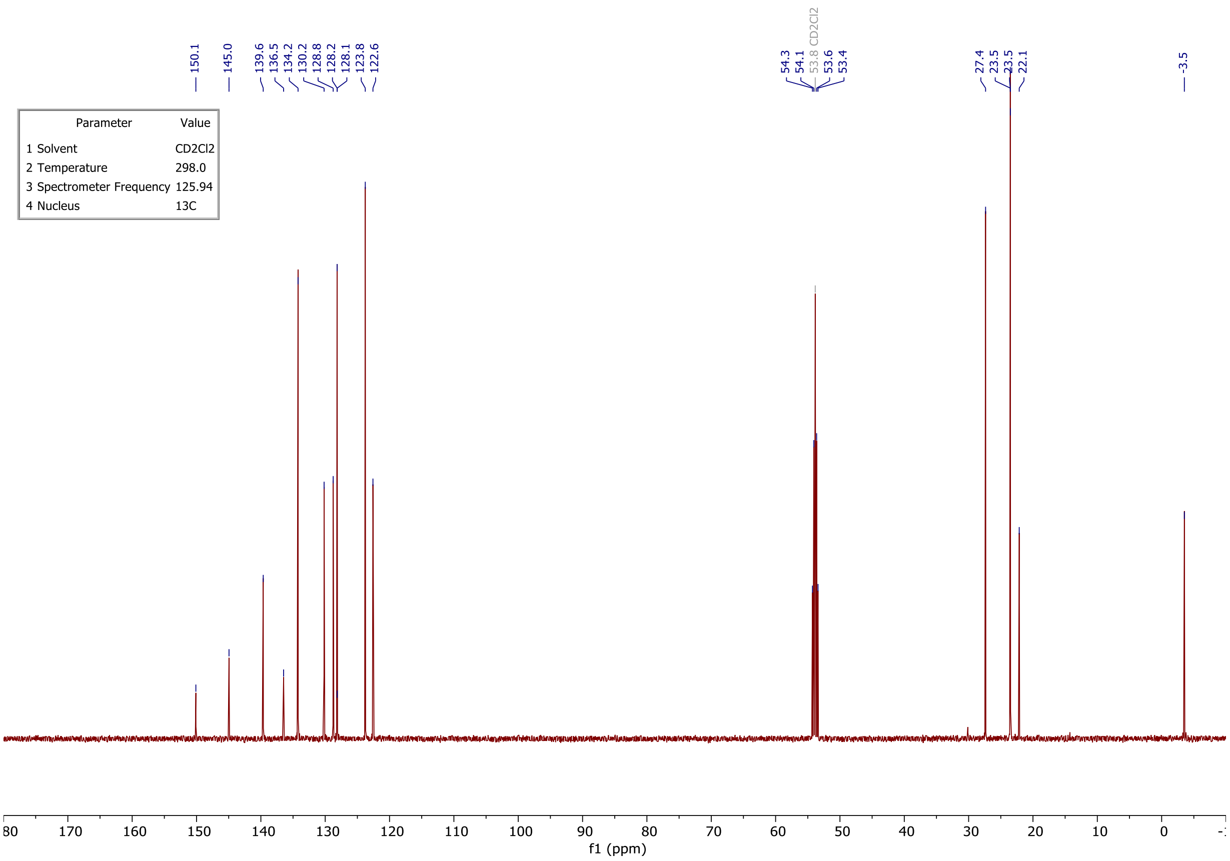

butyl(2,6-dimethoxyphenoxy)(methyl)(2,2,4-trimethylpent-4-en-1-yl)silane 4r

Parameter	Value
1 Solvent	CD2Cl2
2 Temperature	297.9
3 Spectrometer Frequency	500.81
4 Nucleus	1H

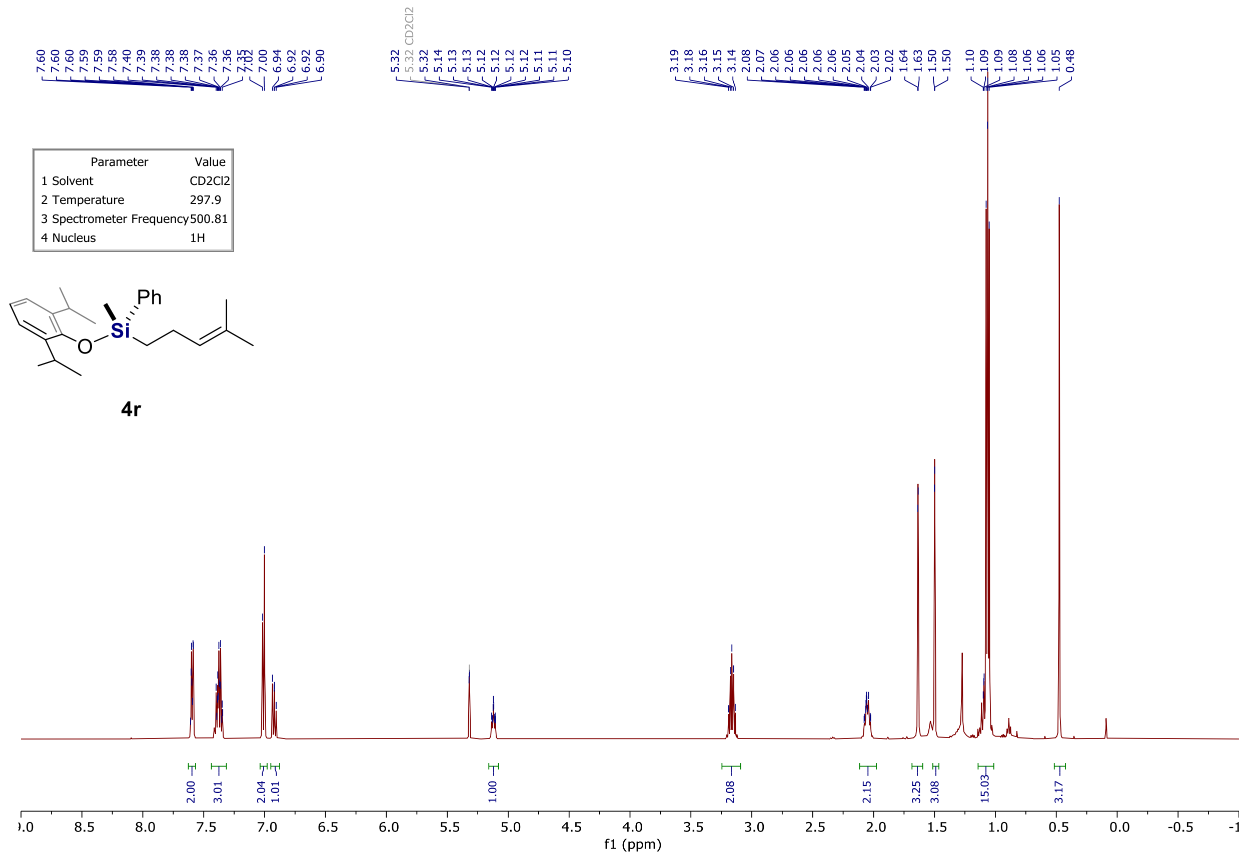

Parameter	Value
1 Solvent	CD2Cl2
2 Temperature	298.9
3 Spectrometer Frequency	125.94
4 Nucleus	13C

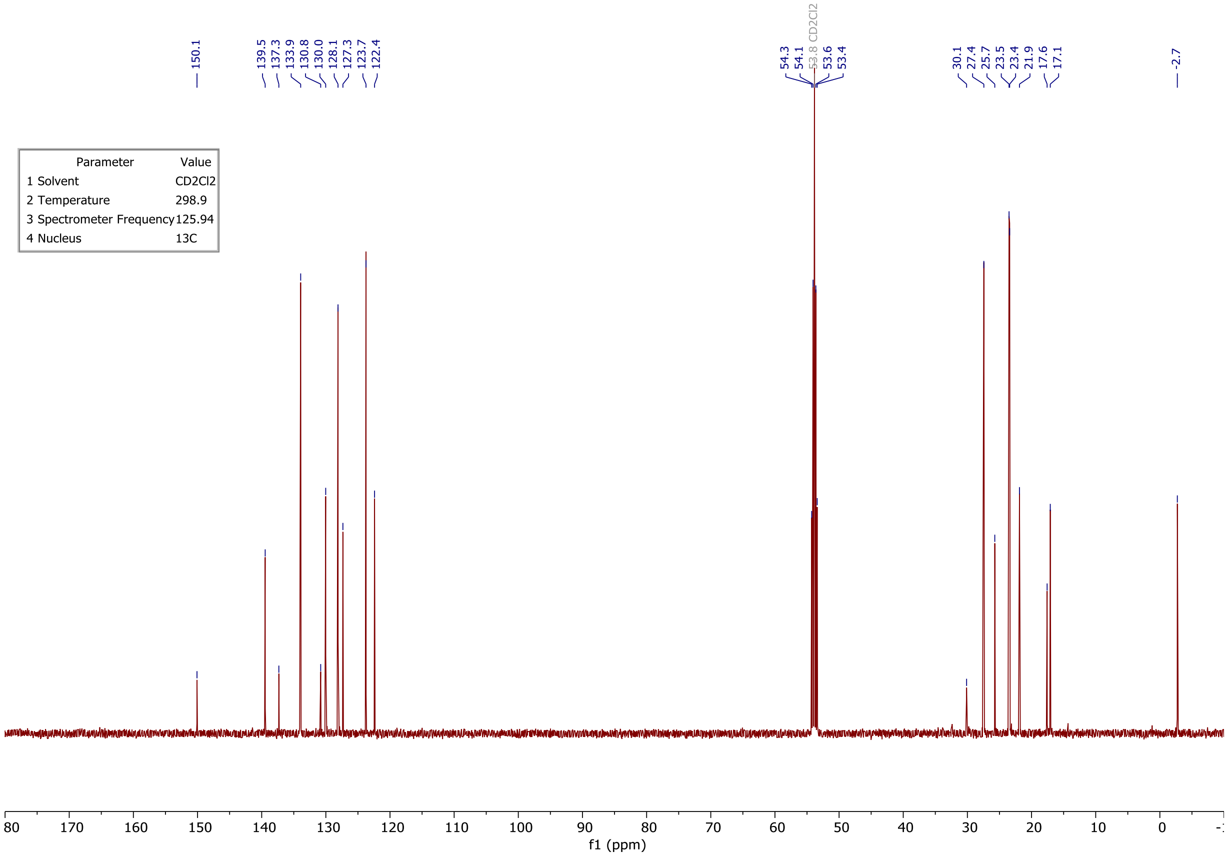

(2,6-dimethoxyphenoxy)(methyl)(pentyl)(2,2,4-trimethylpent-4-en-1-yl)silane 4s

Parameter	Value
1 Solvent	CD2Cl2
2 Temperature	297.9
3 Spectrometer Frequency	500.81
4 Nucleus	1H

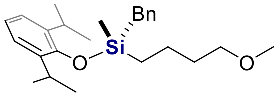

4s

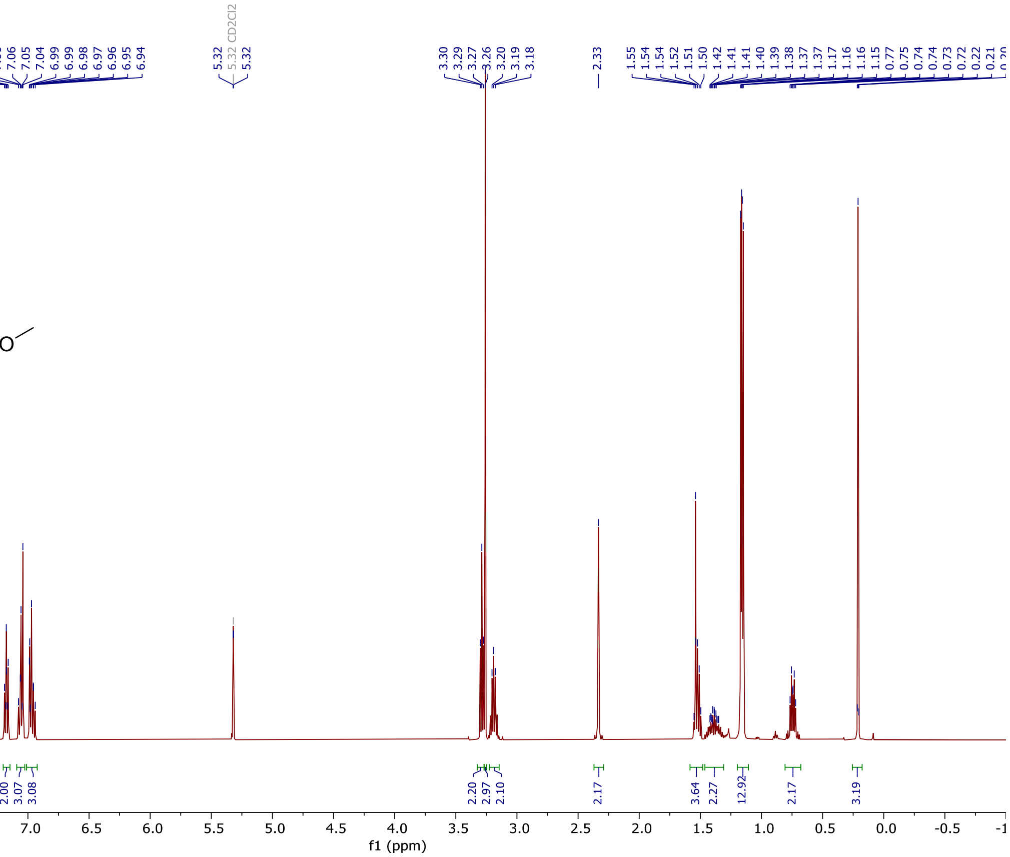

Parameter	Value
1 Solvent	CD2Cl2
2 Temperature	298.0
3 Spectrometer Frequency	125.94
4 Nucleus	13C

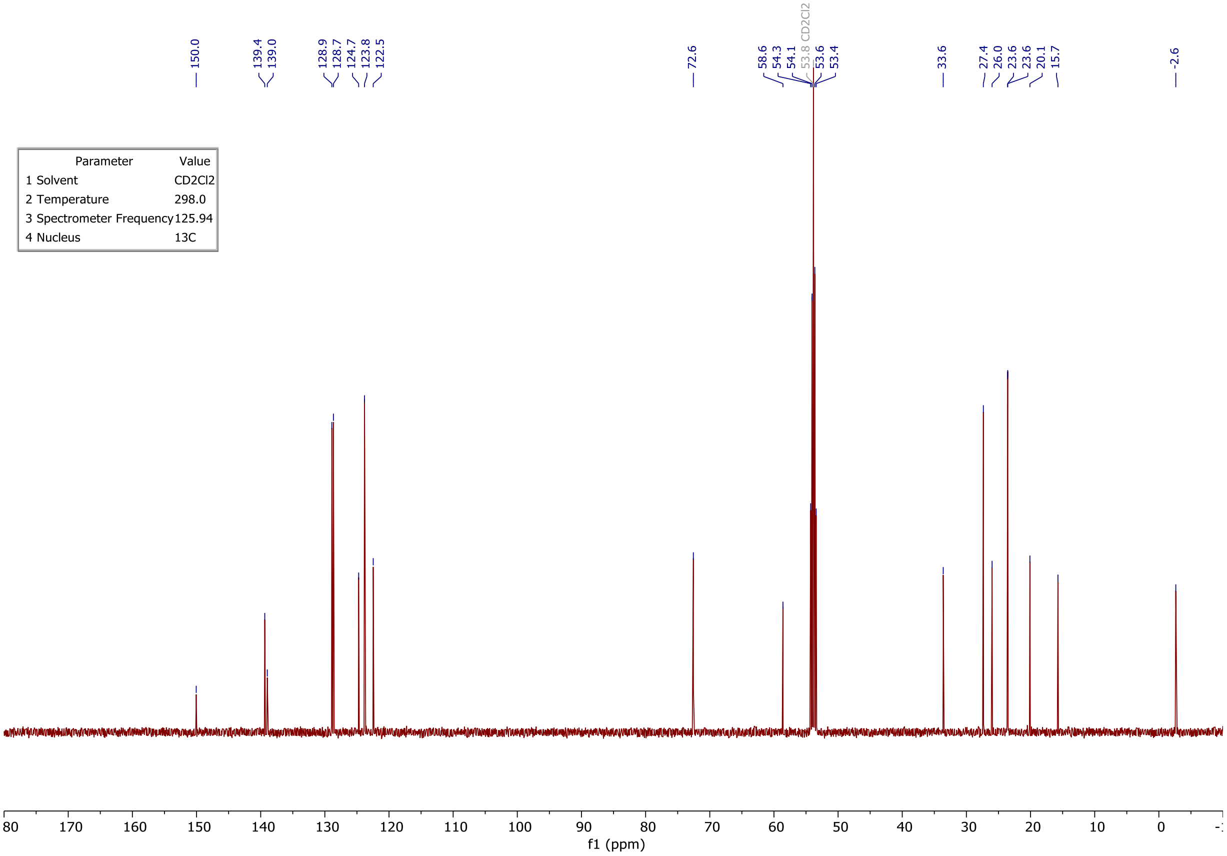

(*R*)-(2,6-diisopropylphenoxy)(methyl)((*S*)-2-methylbutyl)(phenyl)silane 4t

NMR data supports the formation of the following compound:

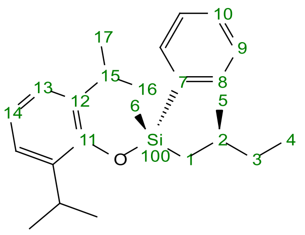

Remarks:
The connectivity of the molecule can be confirmed from the 2D NMR data.
The sample contains one major diastereomer (d:r ~ 18:1, based on ^{29}Si NMR data and comparison to ZHH-ZC-280-02). The relative stereochemistry shown was used as submitted. A confirmation based on NMR is difficult due to the flexibility of the molecule.

User Report
ZHH-ZC-280-03

Atom	J	δ (ppm)	HSQC	COSY	HMBC	NOESY
1 C		24.509	1', 1"		3', 3", 6	
H'	8.60(2), 15.00(1")	0.918	1	1", 2	2, 3, 5, 6, 100	2, 8, 15
H"	15.00(1'), 5.10(2)	1.237	1	1', 2	2, 3, 5, 6, 100	2, 8, 15
2 C		30.727	2		1', 1", 3', 3", 4	
H	8.60(1'), 6.60(5), 5.10(1"), 5.50(3")	1.567	2	1', 1", 3', 3", 5	3, 4, 5, 100	1', 1", 3', 5
3 C		33.486	3', 3"		1', 1", 2, 4	
H'	7.40(4), 12.90(3")	1.206	3	2, 3", 4	1, 2, 4, 5	2
H"	5.50(2), 12.90(3'), 7.40(4)	1.346	3	2, 3', 4	1, 2, 4, 5	
4 C		11.646	4		2, 3', 3"	
H3	7.40(3'), 7.40(3")	0.826	4	3', 3"	2, 3	
5 C		22.466	5		1', 1", 2, 3', 3"	
H3	6.60(2)	0.881	5	2		2, 6
6 C		-1.879	6		1', 1"	
H3		0.491	6		1, 12, 100	5, 8, 15, 16, 17
7 C		137.754			8, 9	
8 C		133.923	8		8, 10	
H		7.588	8	9	7, 8, 10, 100	1', 1", 6, 15, 16, 17
9 C		128.042	9		9	
H		7.351	9	8, 10	7, 9	
10 C		129.929	10		8	
H		7.388	10	9	8	
11 C		150.065			13, 15	
12 C		139.467			6, 14, 15, 16, 17	
13 C		123.697	13		13, 15	
H	7.60(14)	6.995	13	14	11, 13, 15	16, 17
14 C		122.305	14			
H	7.60(13)	6.908	14	13	12	
15 C		27.372	15		13, 16, 17	
H	6.90(16), 6.90(17)	3.147	15	16, 17	11, 12, 13, 16, 17	1', 1", 6, 8, 16, 17
16 C		23.502	16		15, 17	
H3	6.90(15)	1.061	16	15	12, 15, 17	6, 8, 13, 15
17 C		23.353	17		15, 16	
H3	6.90(15)	1.029	17	15	12, 15, 16	6, 8, 13, 15
100 Si		5.914			1', 1", 2, 6, 8	

P-ID: ML00xxx
Measured on: 07/09/2022
CHIFFRE: ZHH-ZC-280-03
ELNA#: 9004
Client: Hui Zhou
Group: List
Spectroscopist: Leutzsch
Analysed on: 11/09/2022
Analysed by: Leutzsch
Amount: 10.0 mg
Solvent: CD2Cl2
Reference: 1H+13C on solvent,other nuclei w/ xiref
Temperature: 298 K
Spectrometer: av600neo
Probe: cryoBBO
Experiments: 1H-zg30, 13C-zgpg30, [13C, 1H]-hsqcedetgpsisp2.3, [13C, 1H]-hmbcetgpl3nd, [1H, 1H]-cosygpppqf, [1H, 1H]-noesygpphpp, [29Si, 1H]-hmbcgpndqf, 29Si-ineptrd

1H NMR (600 MHz, CD_2Cl_2) δ 7.60 – 7.58 (m, 2H), 7.40 – 7.37 (m, 1H), 7.37 – 7.33 (m, 2H), 7.00 (d, J = 7.6 Hz, 2H), 6.93 – 6.89 (m, 1H), 3.15 (hept, J = 6.9 Hz, 2H), 1.61 – 1.53 (m, 1H), 1.35 (ddd, J = 12.9, 7.5, 5.5 Hz, 1H), 1.24 (dd, J = 15.0, 5.1 Hz, 1H), 1.25 – 1.16 (m, 1H), 1.06 (d, J = 6.9 Hz, 6H), 1.03 (d, J = 6.9 Hz, 6H), 0.92 (dd, J = 15.0, 8.6 Hz, 1H), 0.88 (d, J = 6.6 Hz, 3H), 0.83 (t, J = 7.4 Hz, 3H), 0.49 (s, 3H).

1H{off},1D, 600.20 MHz,CD2Cl2,298.0K, pulse sequence: zg30

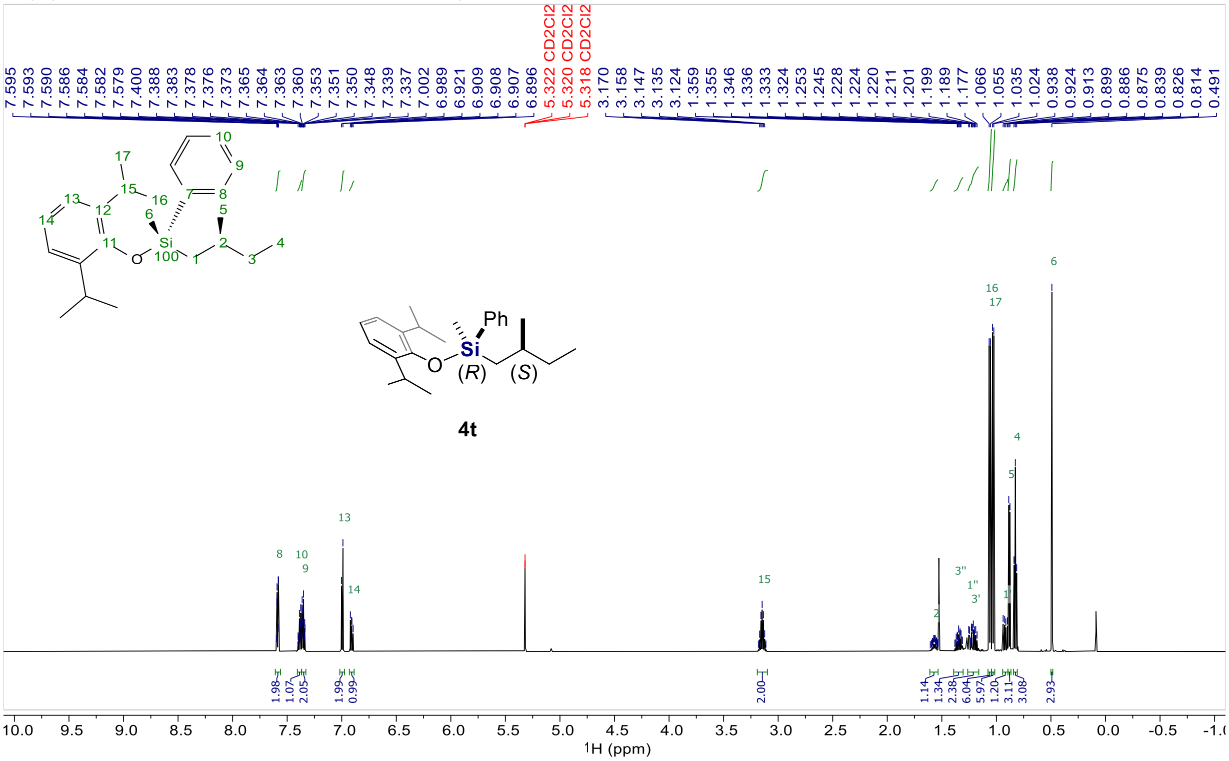

13C NMR (151 MHz, CD_2Cl_2) δ 150.06, 139.47, 137.75, 133.92, 129.93, 128.04, 123.70, 122.31, 33.49, 30.73, 27.37, 24.51, 23.50, 23.35, 22.47, 11.65, -1.88.

13C{1H},1D, 150.94 MHz,CD2Cl2,298.0K, pulse sequence: zgpg30

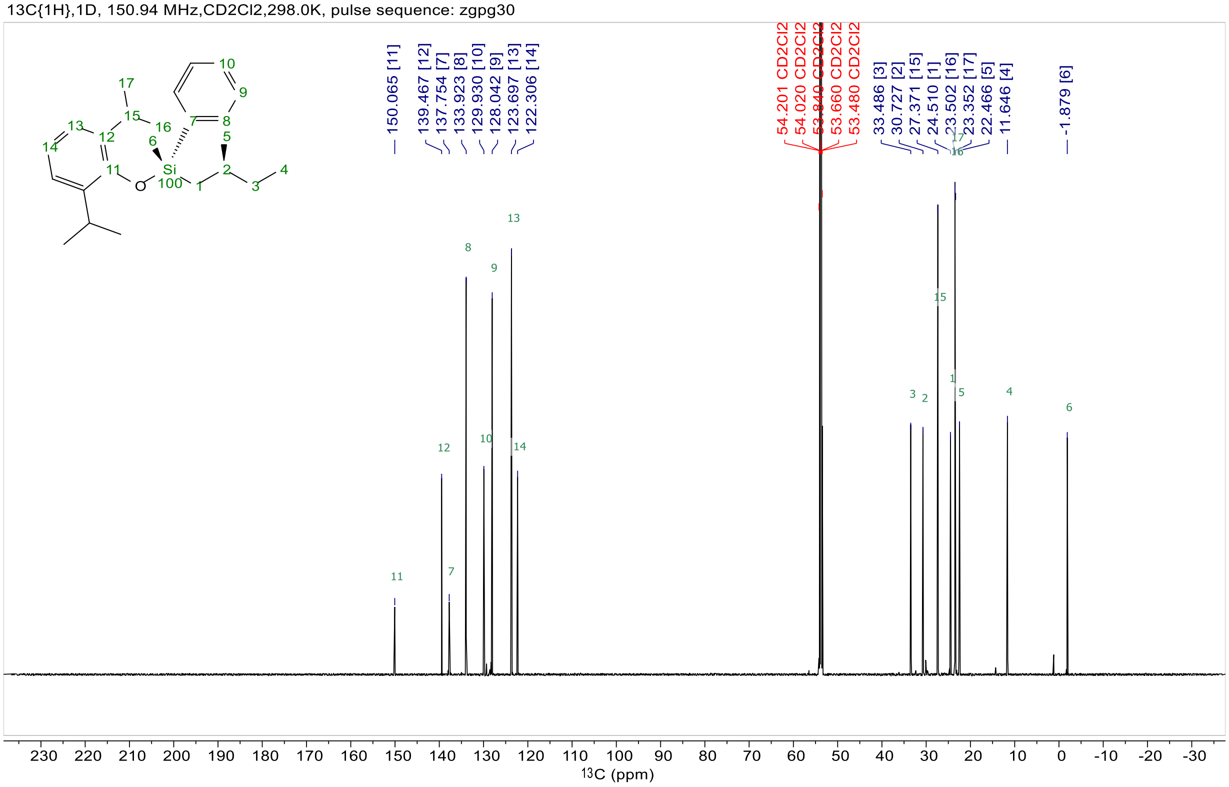

1H{13C},HSQC-EDITED, 600.20 MHz,CD2Cl2,298.0K, pulse sequence: hsqcedetgpsisp2.3

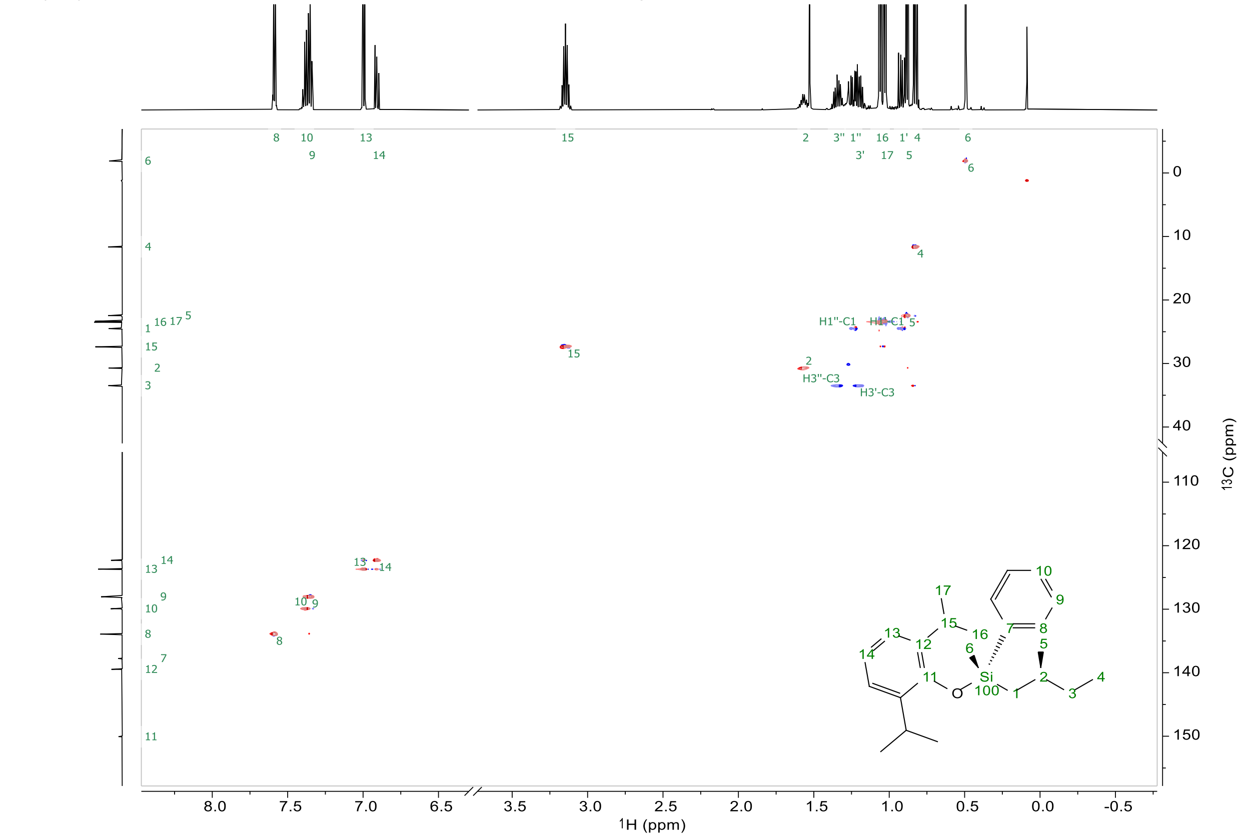

1H{13C},HMBC, 600.20 MHz,CD2Cl2,298.0K, pulse sequence: hmbcetgpl3nd

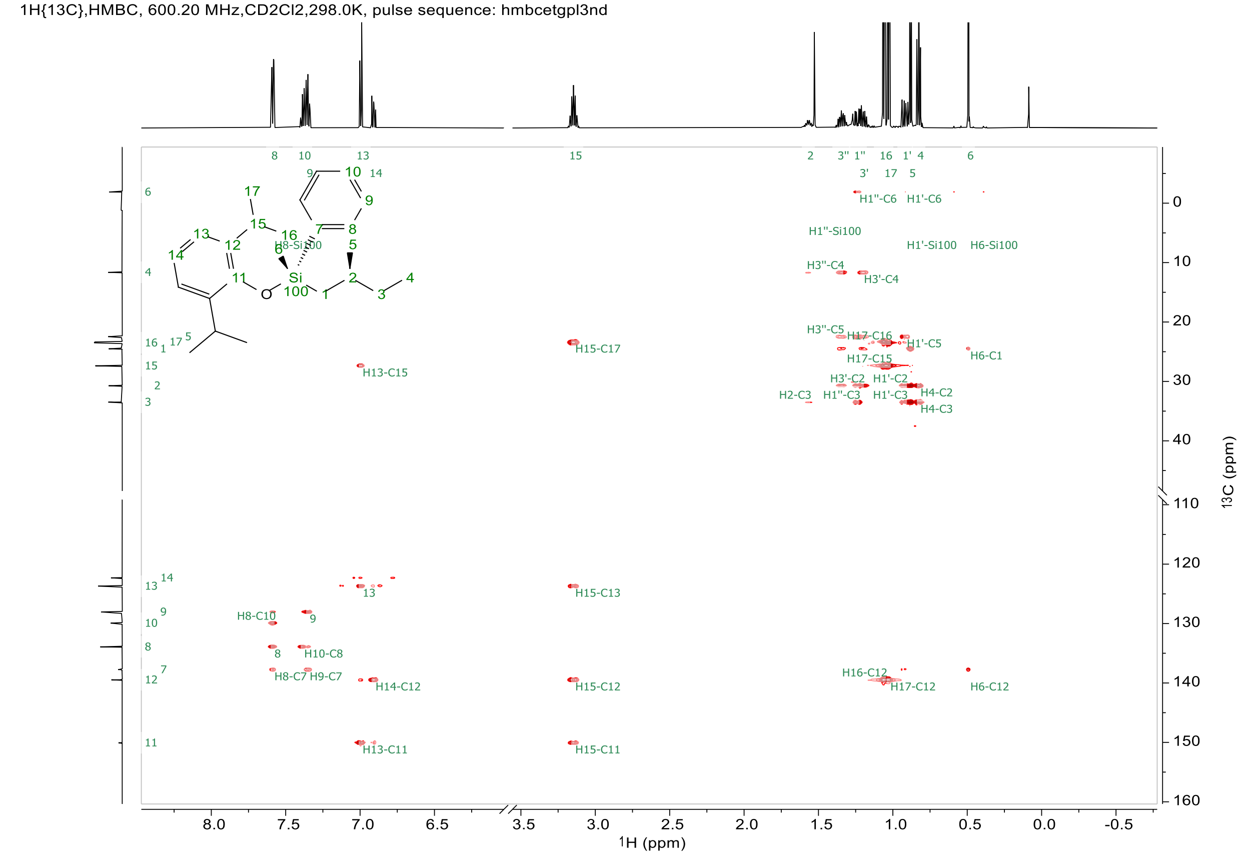

1H{off},COSY, 600.20 MHz,CD2Cl2,298.0K, pulse sequence: cosygpppqf

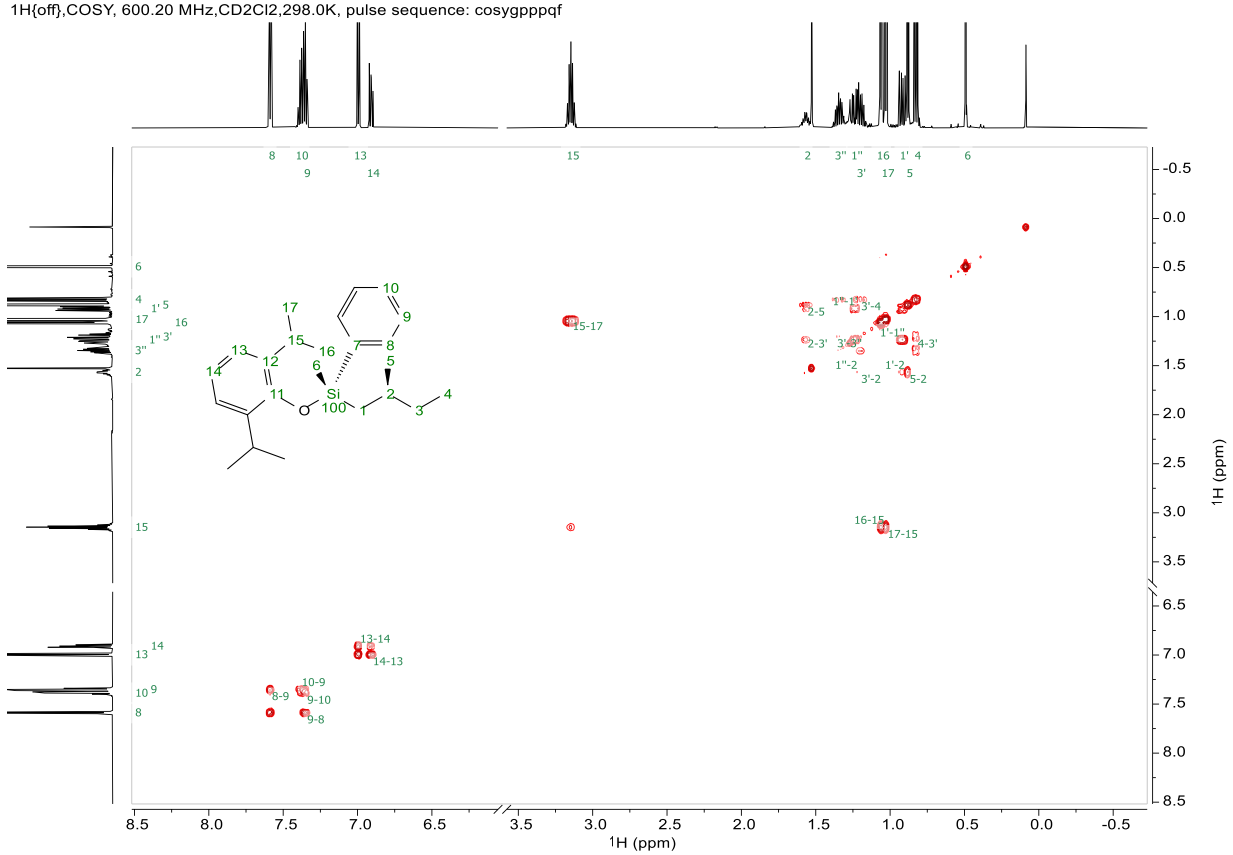

1H{off},NOESY, 600.20 MHz,CD2Cl2,298.0K, pulse sequence: noesygpphpp

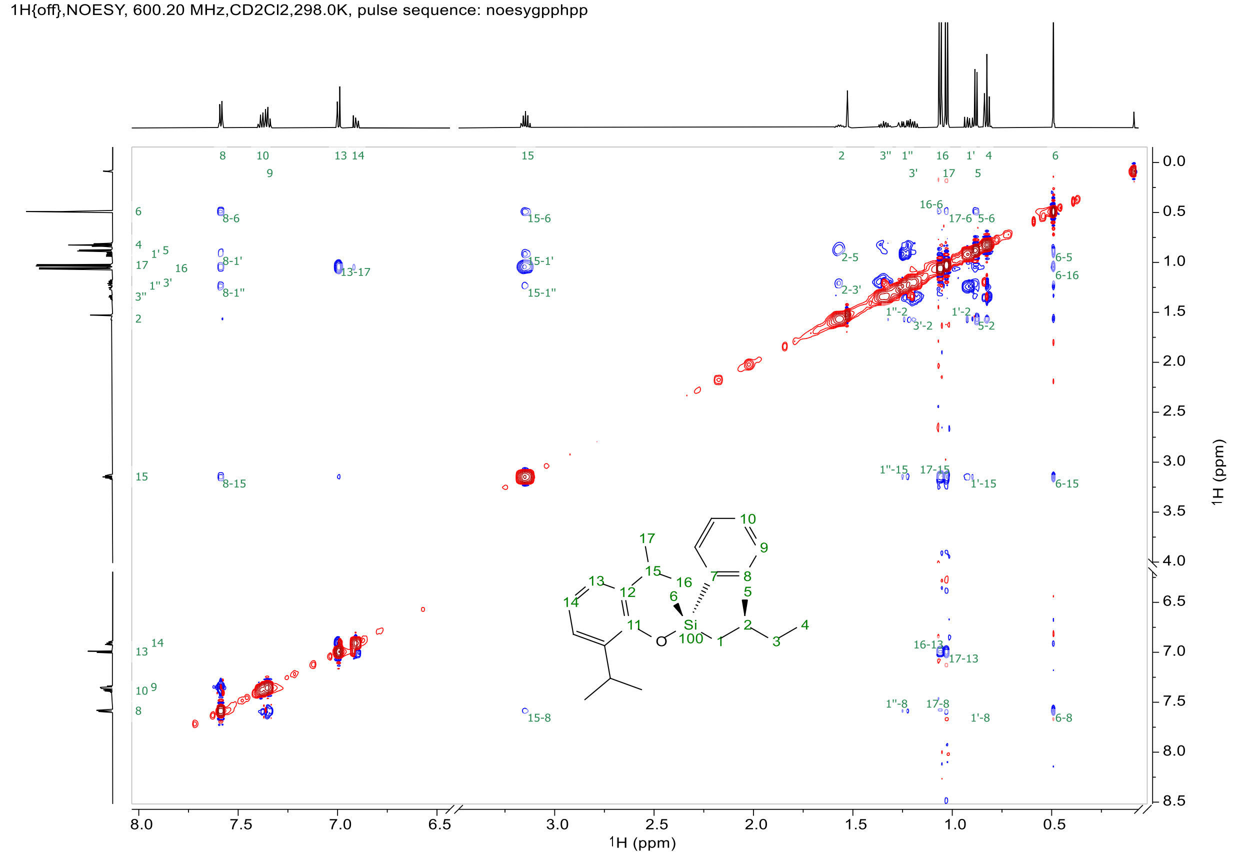

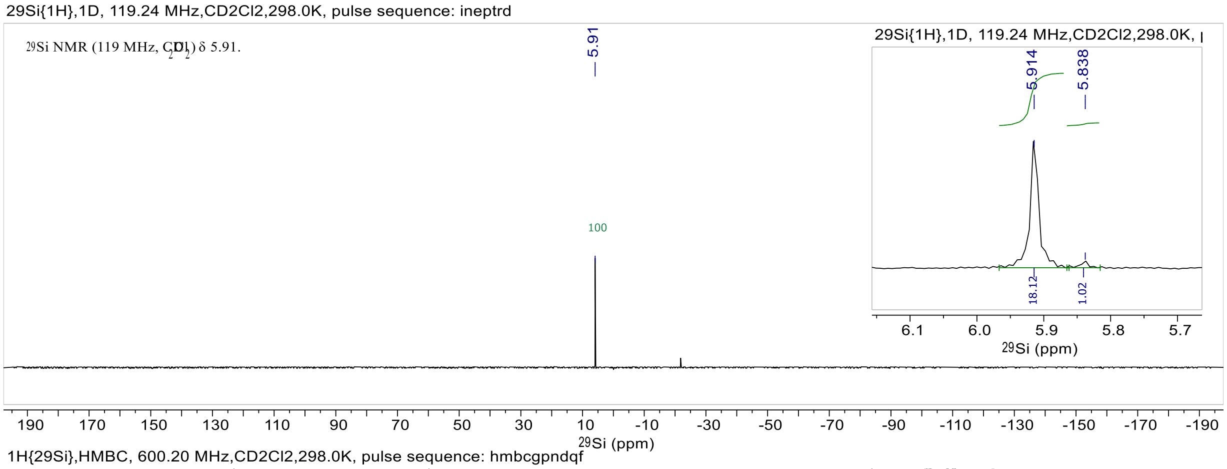
29Si{1H},1D, 119.24 MHz,CD2Cl2,298.0K, pulse sequence: ineptrd
29Si NMR (119 MHz, CD2Cl2) δ 5.91.
5.91
100
29Si{1H},1D, 119.24 MHz,CD2Cl2,298.0K,
5.914
5.838
18.12
1.02
29Si (ppm)

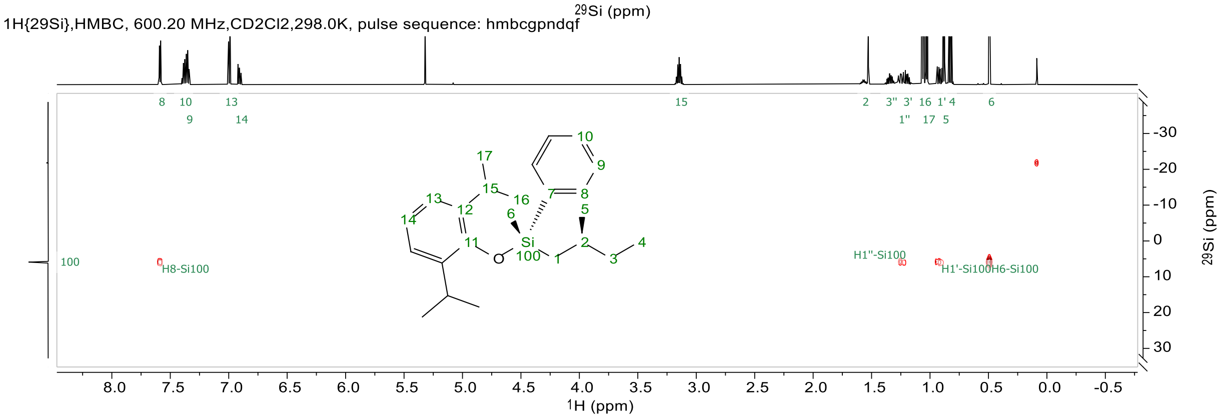
1H{29Si},HMBC, 600.20 MHz,CD2Cl2,298.0K, pulse sequence: hmbcgpndqf
H8-Si100
H1''-Si100
H1'-Si100
H6-Si100
1H (ppm)
29Si (ppm)

(*S*)-(2,6-diisopropylphenoxy)(methyl)((*S*)-2-methylbutyl)(phenyl)silane 4u

NMR data supports the formation of the following compound:

User Report
ZHH-ZC-280-02

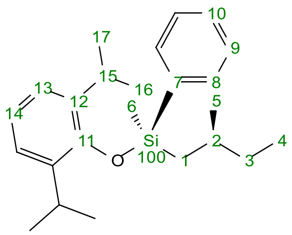

Remarks:
The connectivity of the molecule can be confirmed from the 2D NMR data.
The sample contains one major diastereomer (d:r > 20:1, based on ^{29}Si NMR data and comparison to ZHH-ZC-280-03). The relative stereochemistry shown was used as submitted. A confirmation based on NMR is difficult due to the flexibility of the molecule.

Atom	J	δ (ppm)	HSQC	COSY	HMBC	NOESY
1 C		24.390	1', 1''		3', 3'', 6	
H'	8.60(2), 15.00(1'')	0.974	1	1'', 2	2, 3, 5, 6, 100	2, 8
H''	15.00(1'), 5.10(2)	1.152	1	1', 2	2, 3, 5, 6, 100	2
2 C		30.776	2		1', 1'', 3', 3'', 4	
H	8.60(1'), 6.60(5), 5.10(1''), 5.50(3'')	1.599	2	1', 1'', 3', 3'', 5	3, 4, 5, 100	1', 1'', 3', 3'', 5
3 C		33.374	3', 3''		1', 1'', 2, 4	
H'	7.40(4), 12.90(3'')	1.202	3	2, 3'', 4	1, 2, 4, 5	2
H''	5.50(2), 12.90(3'), 7.40(4)	1.321	3	2, 3', 4	1, 2, 4, 5	2
4 C		11.555	4		2, 3', 3''	
H3	7.40(3'), 7.40(3'')	0.815	4	3', 3''	2, 3	
5 C		22.624	5		1', 1'', 2, 3', 3''	
H3	6.60(2)	0.890	5	2		2
6 C		-2.096	6		1', 1''	
H3		0.494	6		1, 12, 100	6, 8, 15
7 C		137.975			8, 9	
8 C		133.897	8		8, 10	
H		7.590	8	9	7, 8, 10, 100	1', 6, 15, 16, 17

Atom	J	δ (ppm)	HSQC	COSY	HMBC	NOESY
9 C		128.024	9		9	
H		7.354	9	8, 10	7, 9	
10 C		129.911	10		8	
H		7.381	10	9	8	
11 C		150.092			13, 15	
12 C		139.461			6, 14, 15, 16, 17	
13 C		123.692	13		13, 15	
H		6.994	13	14	11, 13, 15	16, 17
14 C		122.305	14			
H		6.906	14	13	12	
15 C		27.374	15		13, 16, 17	
H	6.90(16), 6.90(17)	3.153	15	16, 17	11, 12, 13, 16, 17	6, 8, 16, 17
16 C		23.518	16		15, 17	
H3	6.90(15)	1.061	16	15	12, 15, 17	8, 13, 15
17 C		23.332	17		15, 16	
H3	6.90(15)	1.029	17	15	12, 15, 16	8, 13, 15
100 Si		5.880			1', 1'', 2, 6, 8	

P-ID: ML00xxx
Measured on: 07/09/2022
CHIFFRE: ZHH-ZC-280-02
ELNA#: 9003
Client: Hui Zhou
Group: List
Spectroscopist: Leutzsch
Analysed on: 11/09/2022
Analysed by: Leutzsch
Amount: 10.0 mg
Solvent: CD2Cl2
Reference: 1H+13C on solvent,other nuclei w/ xiref
Temperature: 298 K
Spectrometer: av600neo
Probe: cryoBBO
Experiments: 1H-zg30, 13C-zgpg30, [13C, 1H]-hsqcedetgpsisp2.3, [13C, 1H]-hmbcetgpl3nd, [1H, 1H]-cosygpppqf, [1H, 1H]-noesygpphpp, 29Si-ineptrd, [29Si, 1H]-hmbcgpndqf

1H NMR (600 MHz, CD_2Cl_2) δ 7.61 – 7.57 (m, 2H), 7.41 – 7.36 (m, 1H), 7.36 – 7.32 (m, 2H), 6.99 (d, J = 7.6 Hz, 2H), 6.94 – 6.88 (m, 1H), 3.15 (hept, J = 6.9 Hz, 2H), 1.64 – 1.55 (m, 1H), 1.32 (dqd, J = 12.9, 7.4, 5.5 Hz, 1H), 1.27 – 1.16 (m, 1H), 1.13 (dd, J = 15.0, 5.1 Hz, 1H), 1.06 (d, J = 6.9 Hz, 6H), 1.03 (d, J = 6.9 Hz, 6H), 0.97 (dd, J = 15.0, 8.6 Hz, 1H), 0.89 (d, J = 6.6 Hz, 3H), 0.82 (t, J = 7.4 Hz, 3H), 0.49 (s, 3H).

1H{off},1D, 600.20 MHz,CD2Cl2,298.0K, pulse sequence: zg30

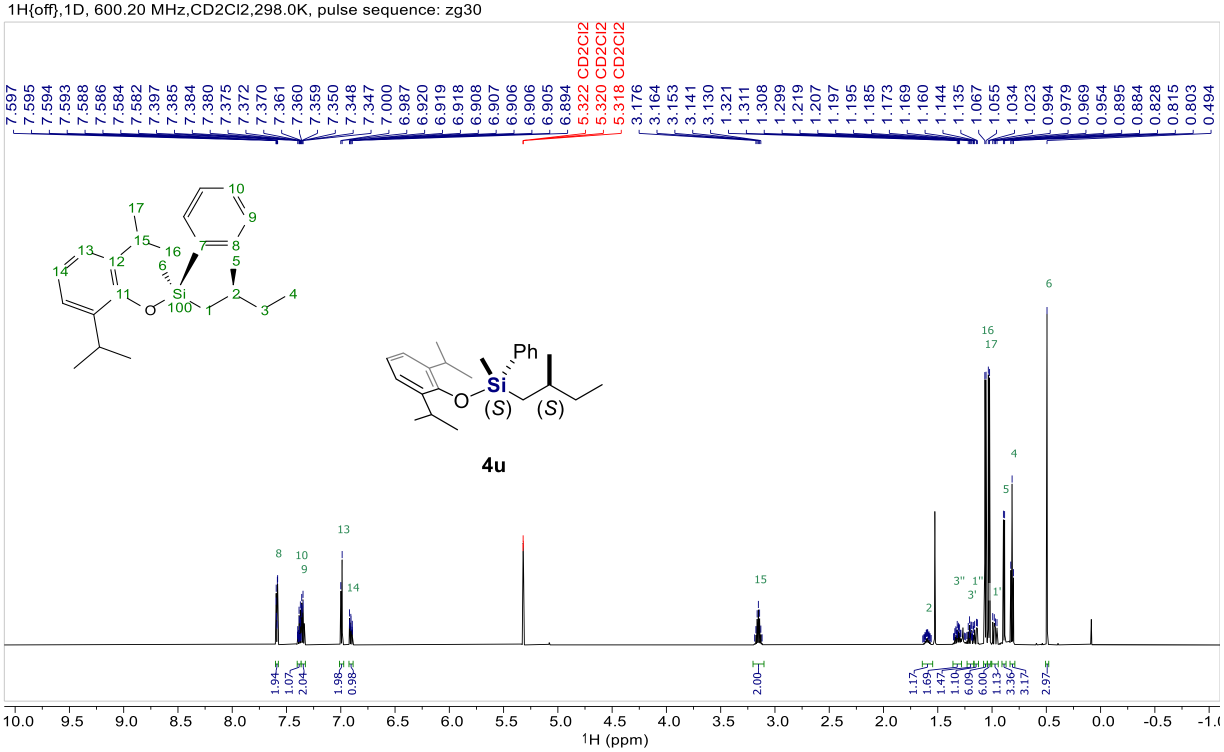

13C NMR (151 MHz, CD_2Cl_2) δ 150.09 (11), 139.46 (12), 137.98 (7), 133.90 (8), 129.91 (10), 128.02 (9), 123.69 (13), 122.30 (14), 33.37 (3), 30.78 (2), 27.37 (15), 24.39 (1), 23.52 (16), 23.33 (17), 11.56 (4), -2.10 (6).

13C{1H},1D, 150.94 MHz,CD2Cl2,298.0K, pulse sequence: zgpg30

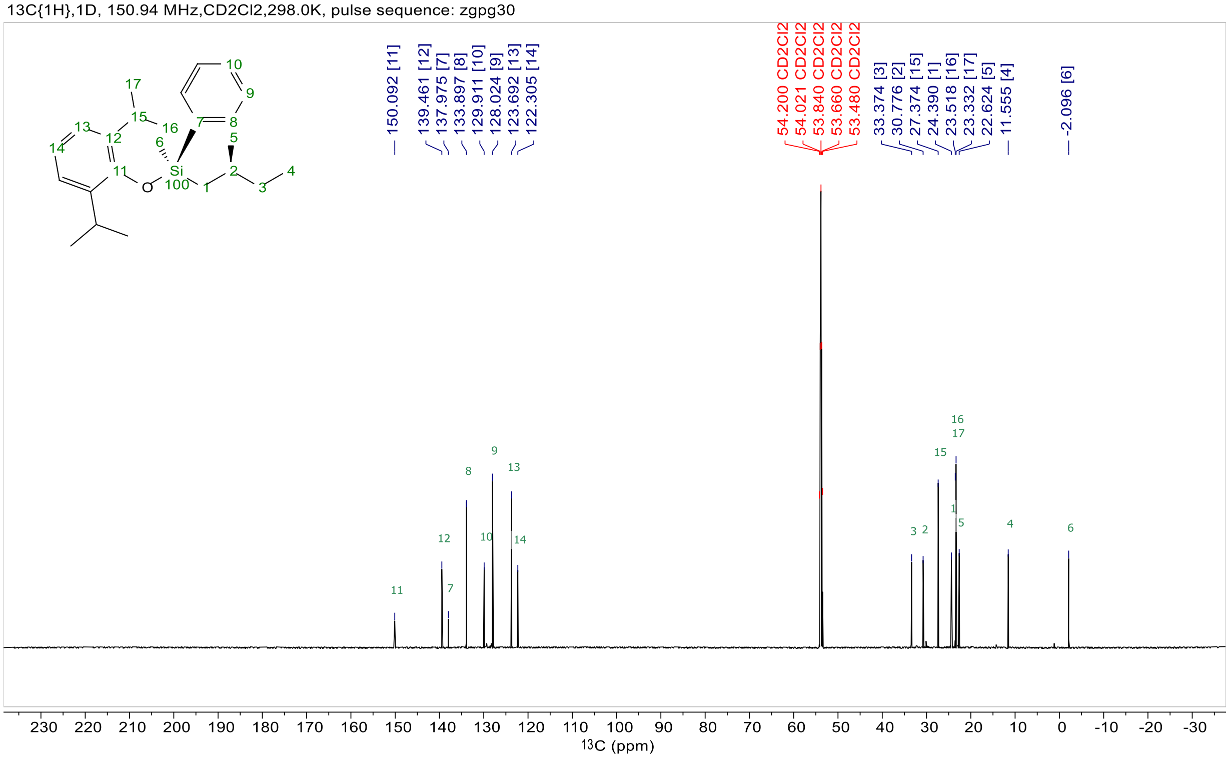

1H{13C},HSQC-EDITED, 600.20 MHz,CD2Cl2,298.0K, pulse sequence: hsqcedetgpsisp2.3

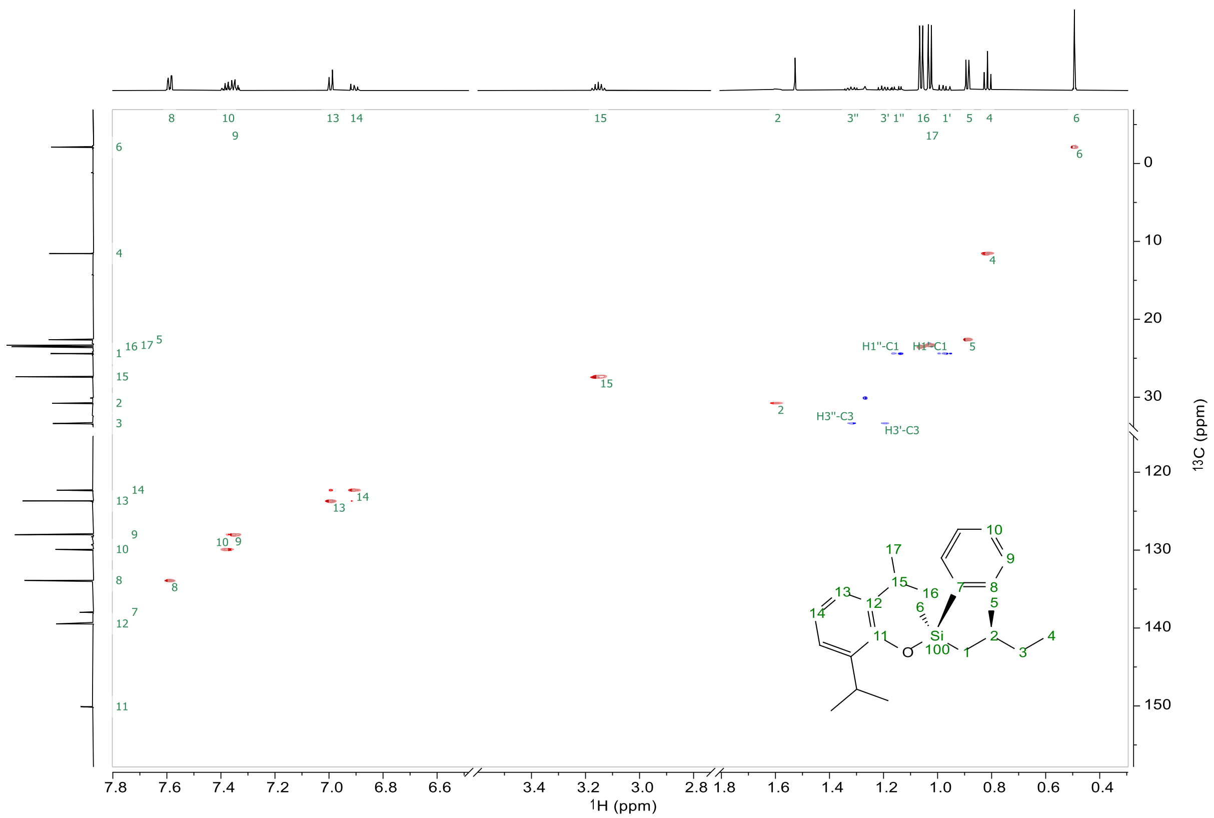

1H{13C},HMBC, 600.20 MHz,CD2Cl2,298.0K, pulse sequence: hmbcetgpl3nd

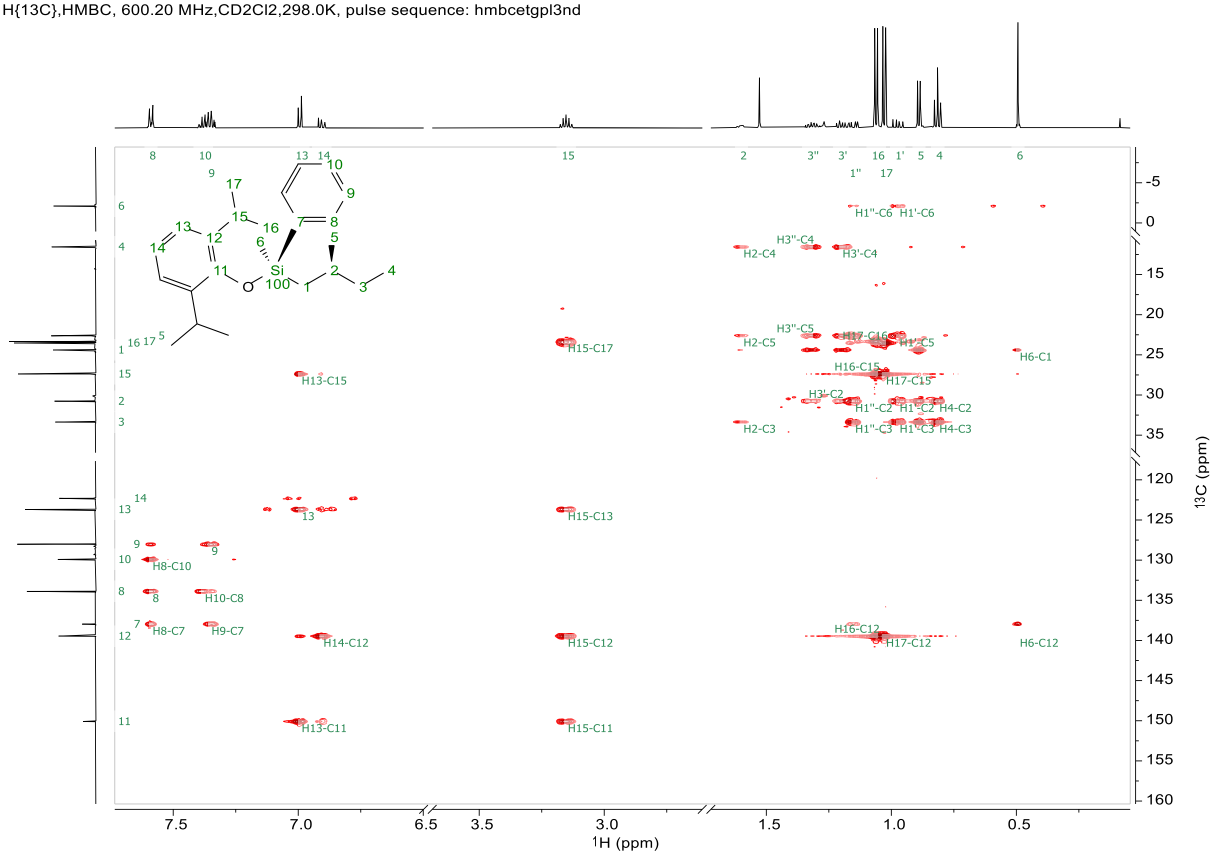

1H{off},COSY, 600.20 MHz,CD2Cl2,298.0K, pulse sequence: cosygpppqf

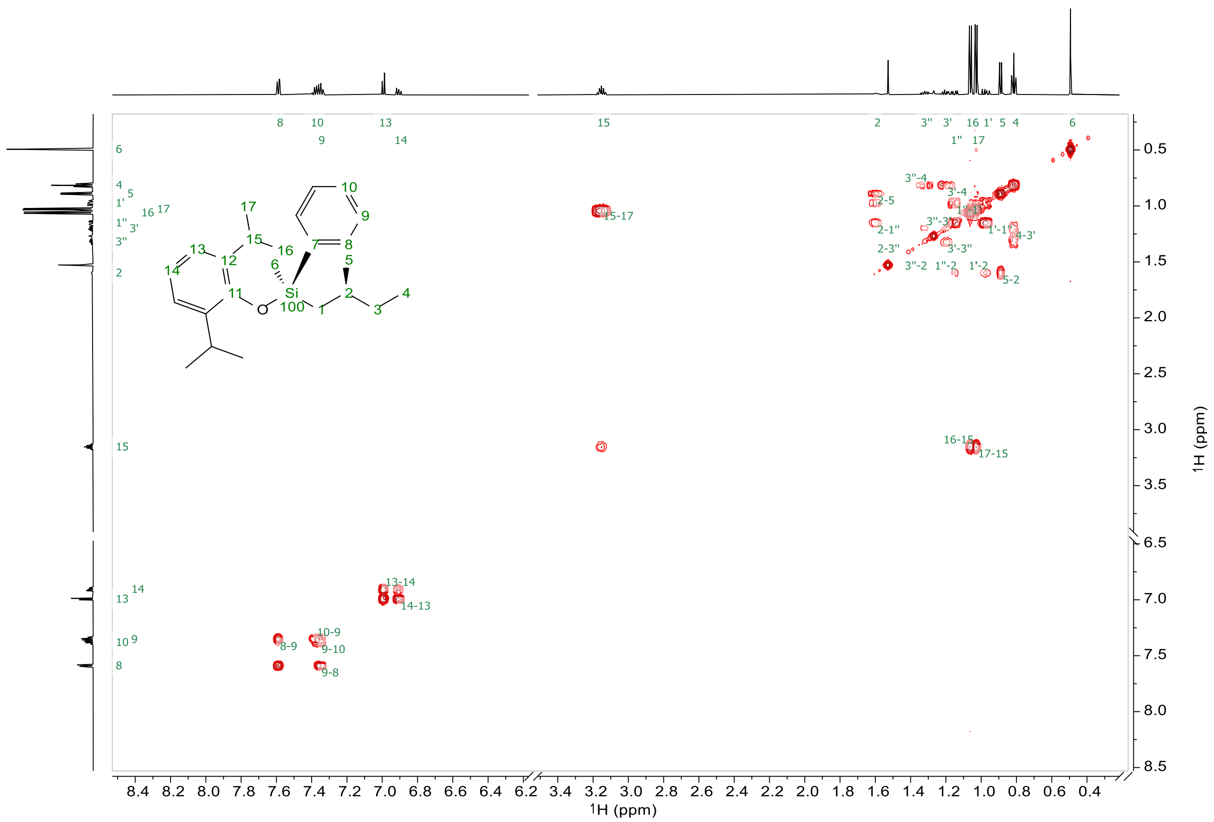

1H{off},NOESY, 600.20 MHz,CD2Cl2,298.0K, pulse sequence: noesygpphpp

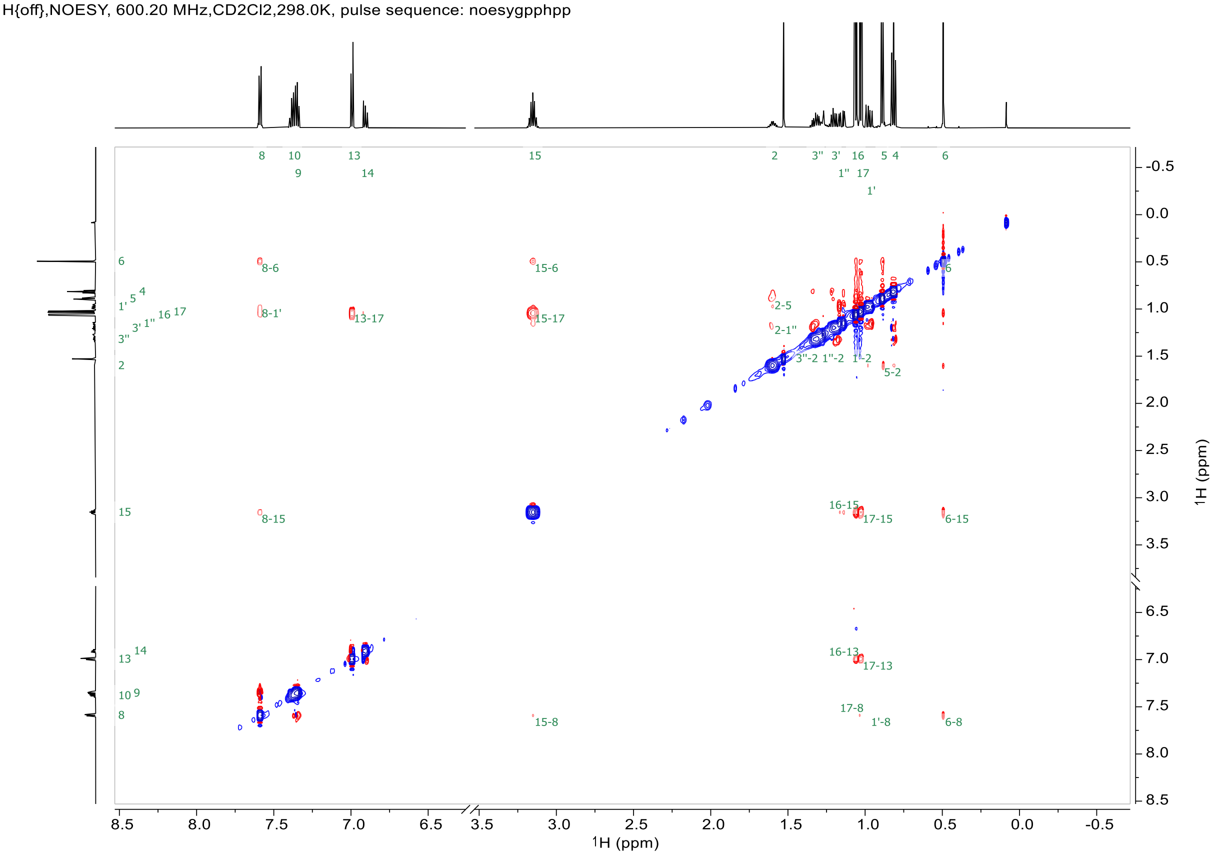

29Si{1H},1D, 119.24 MHz,CD2Cl2,298.0K, pulse sequence: ineptrd

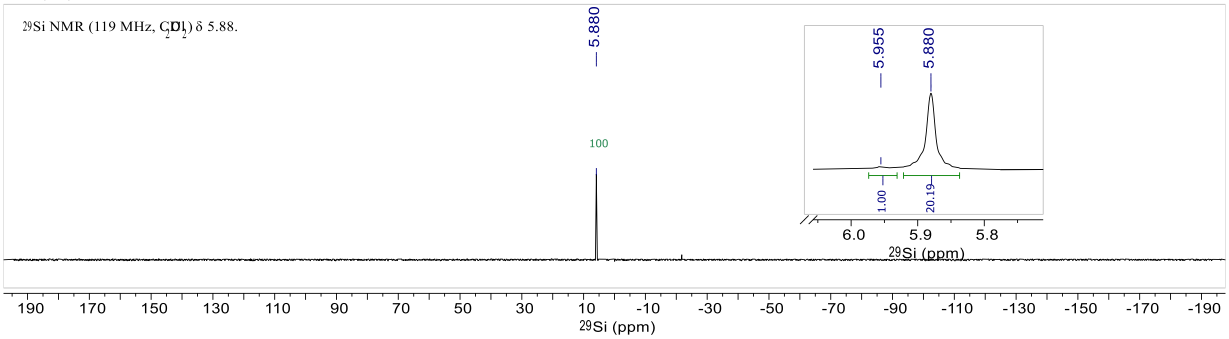

1H{29Si},HMBC, 600.20 MHz,CD2Cl2,298.0K, pulse sequence: hmbcgpndqf

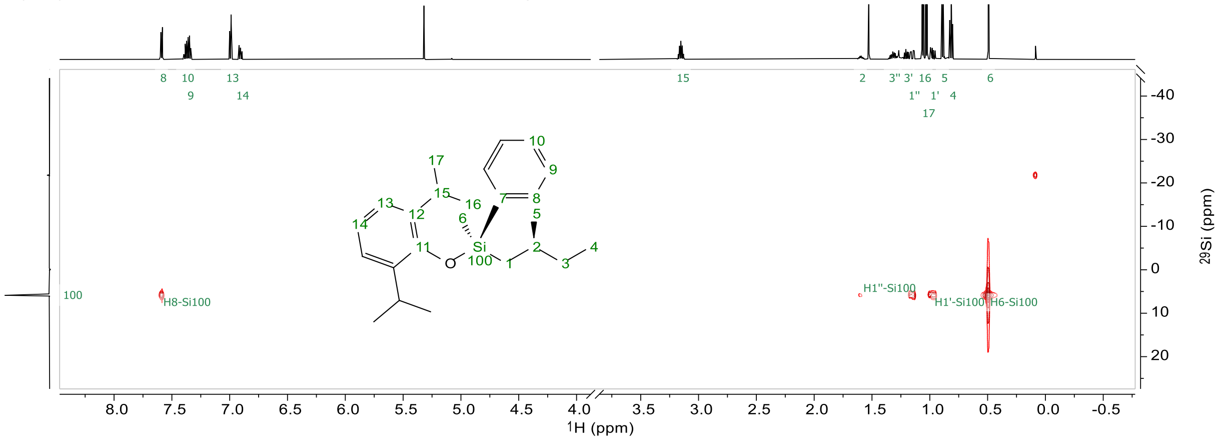

hexyl(methyl)(phenyl)silane 5

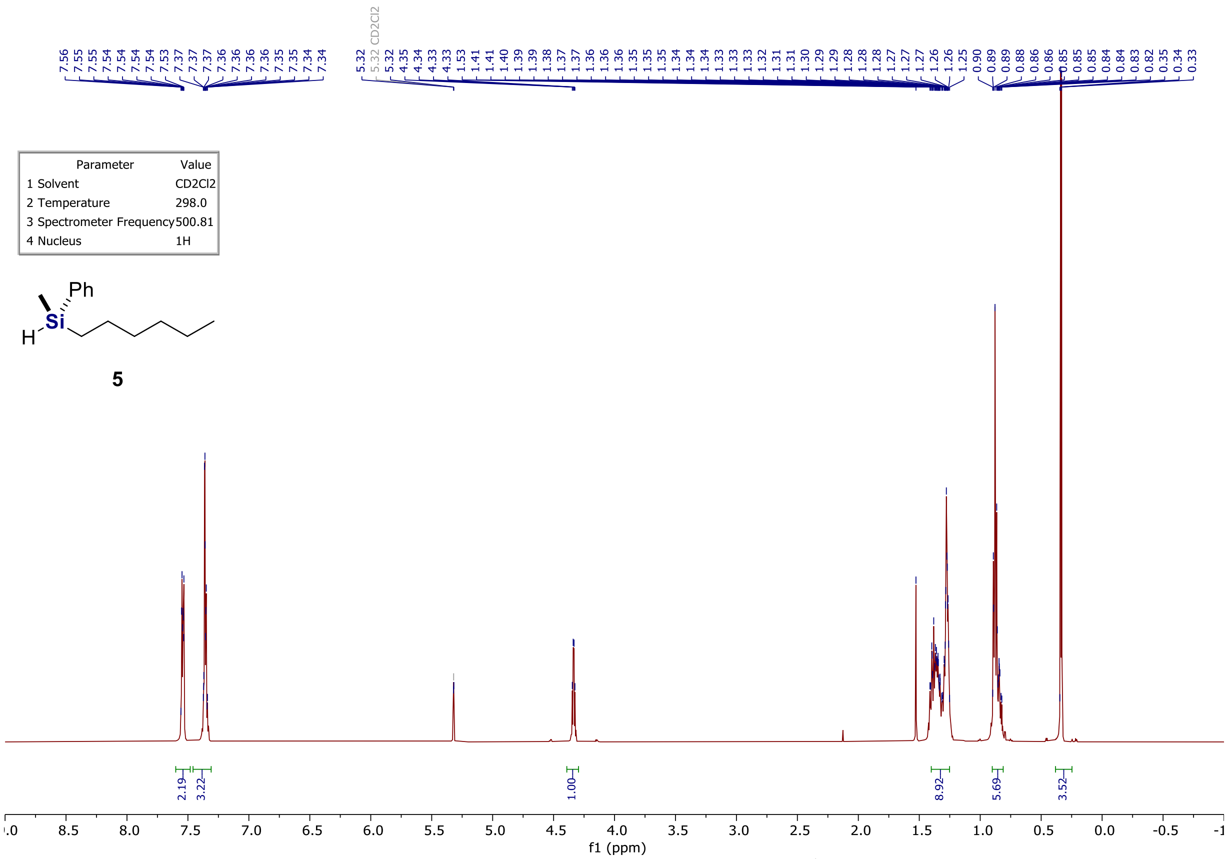

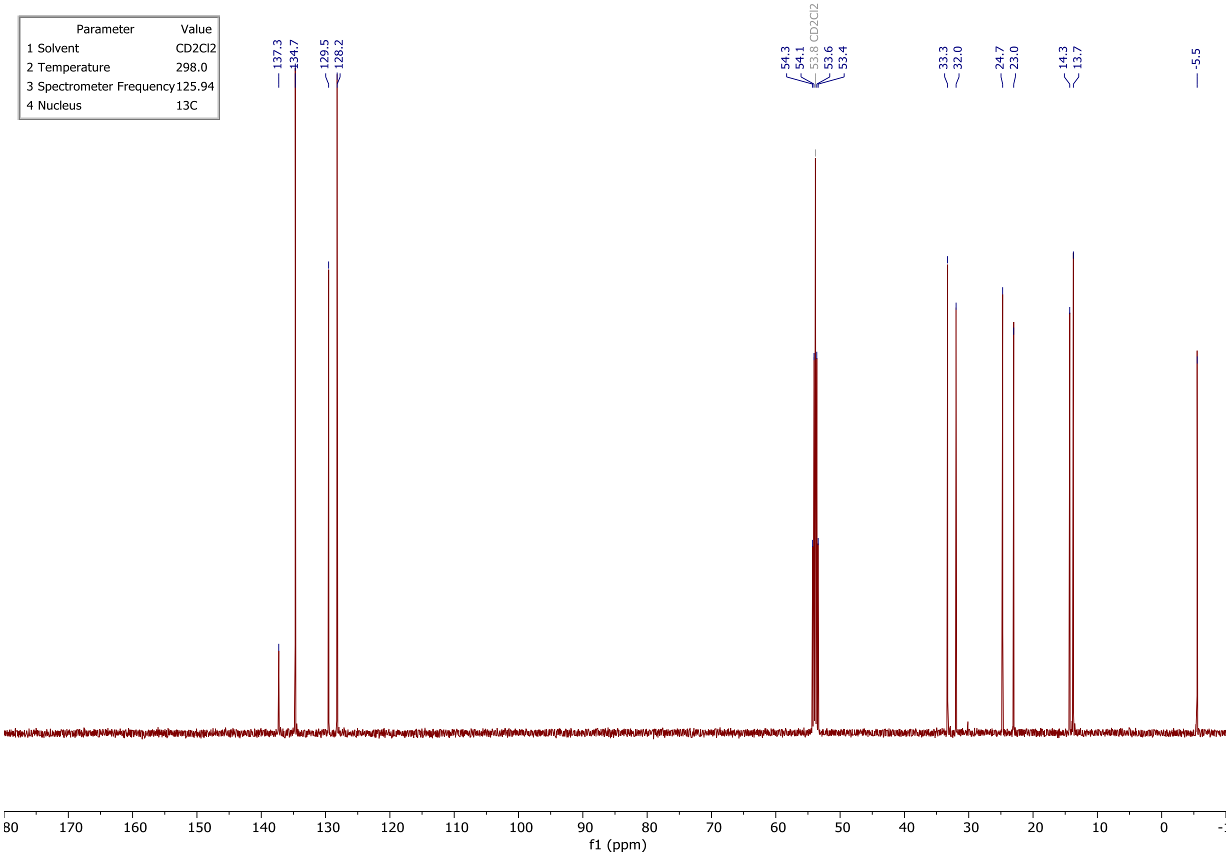

1,3-dihexyl-1,3-dimethyl-1,3-diphenyldisiloxane 6

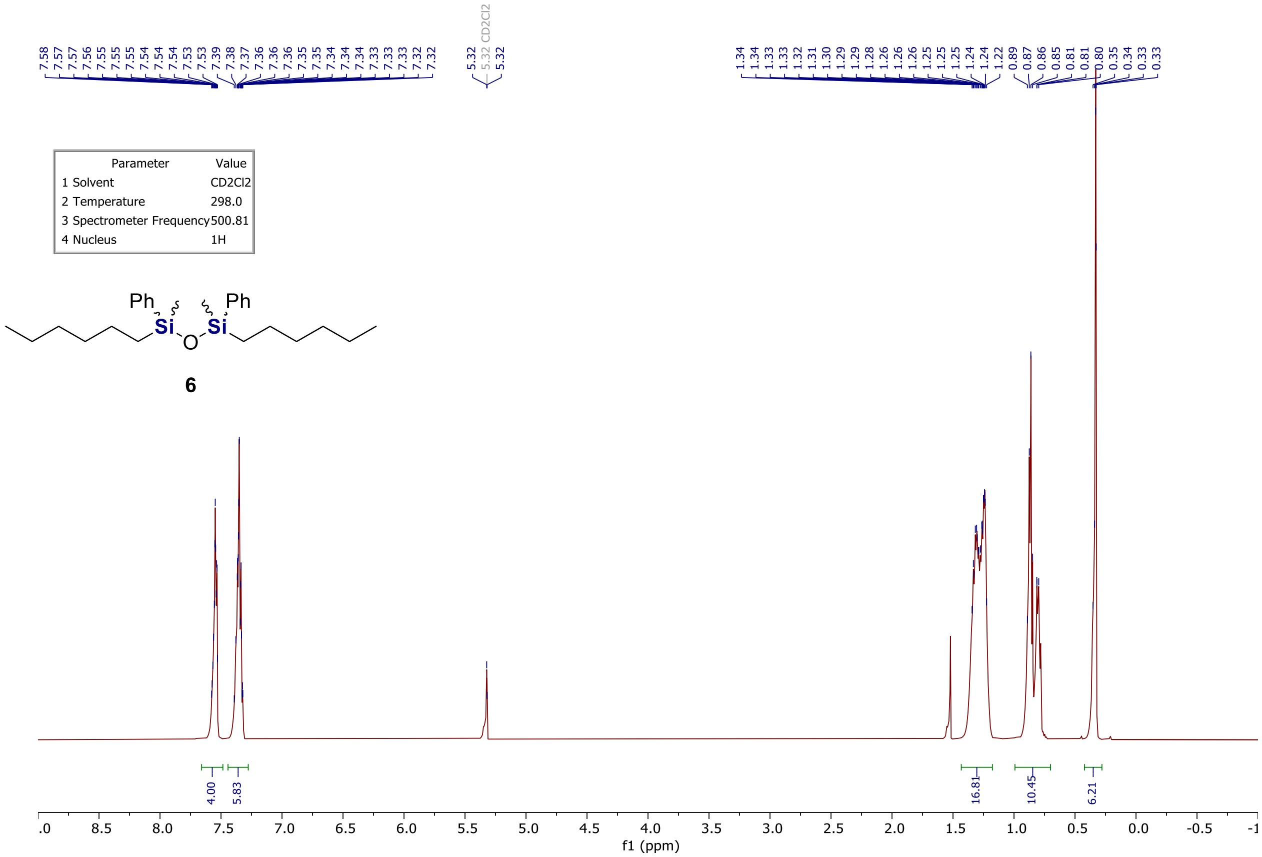

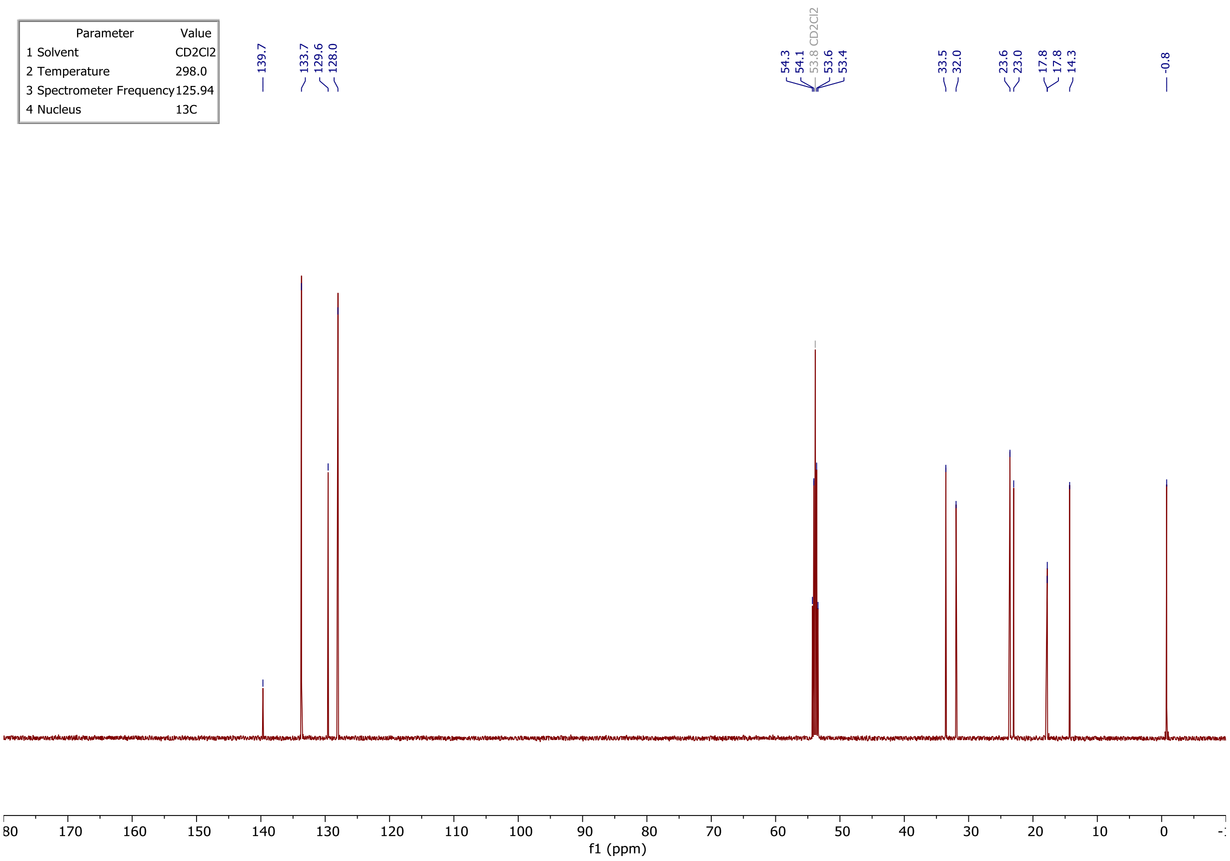

hexyl(methyl)(phenyl)silanol

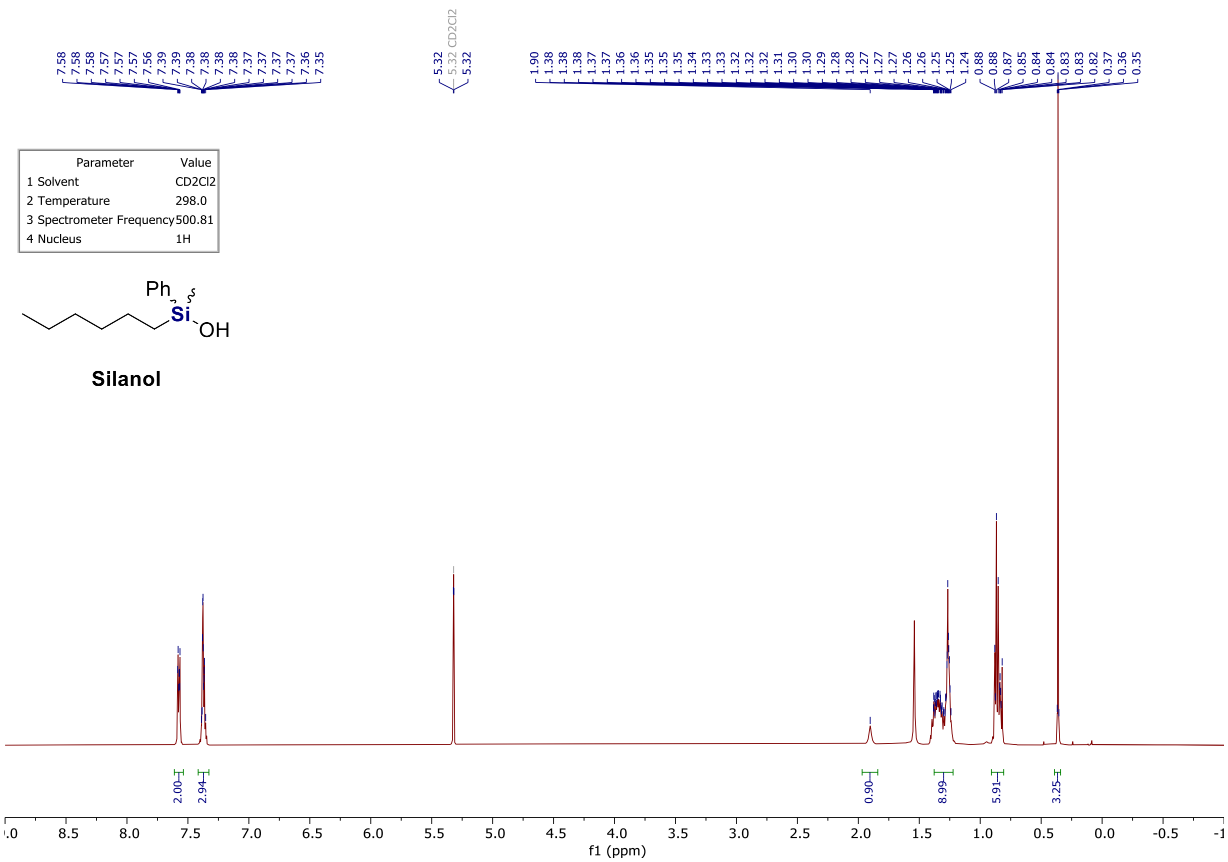

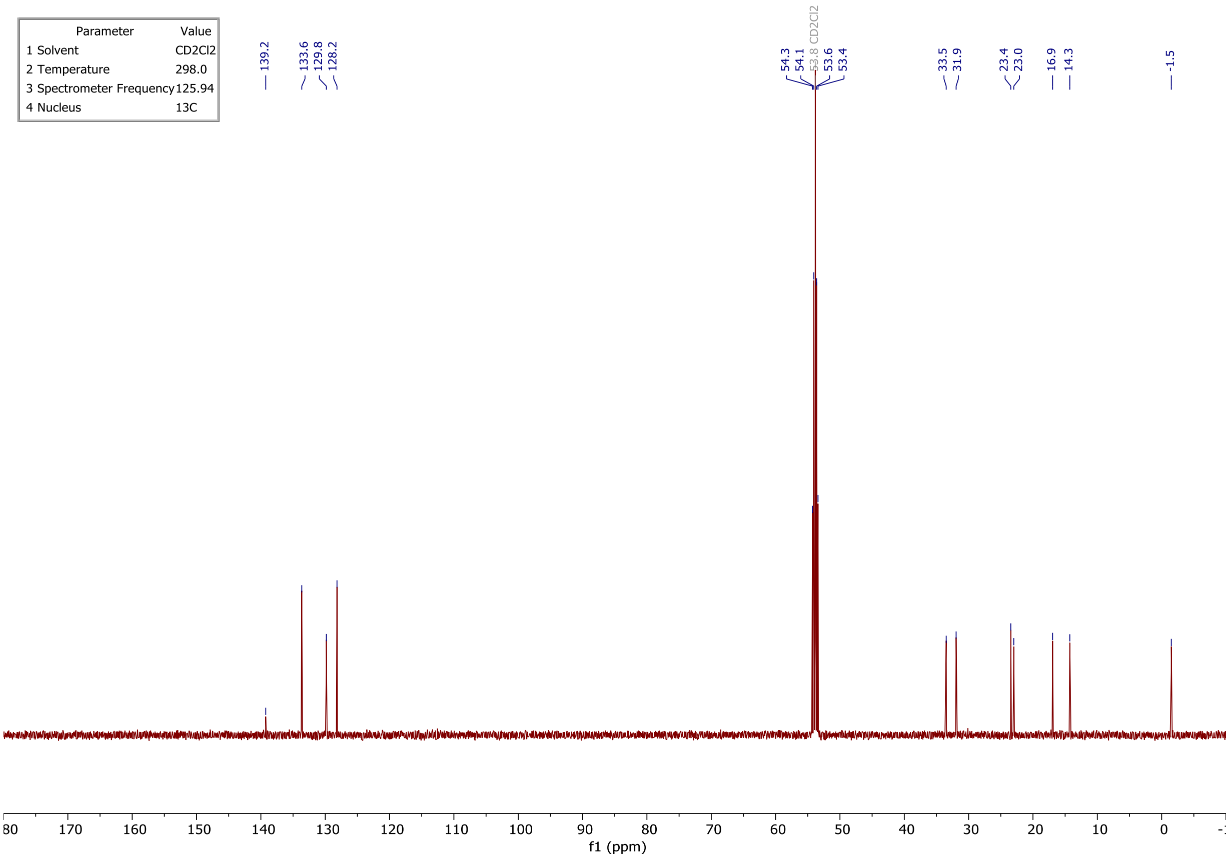

[1,1'-biphenyl]-4-yl(cyclohexyl)(methyl)(2-methylallyl)silane

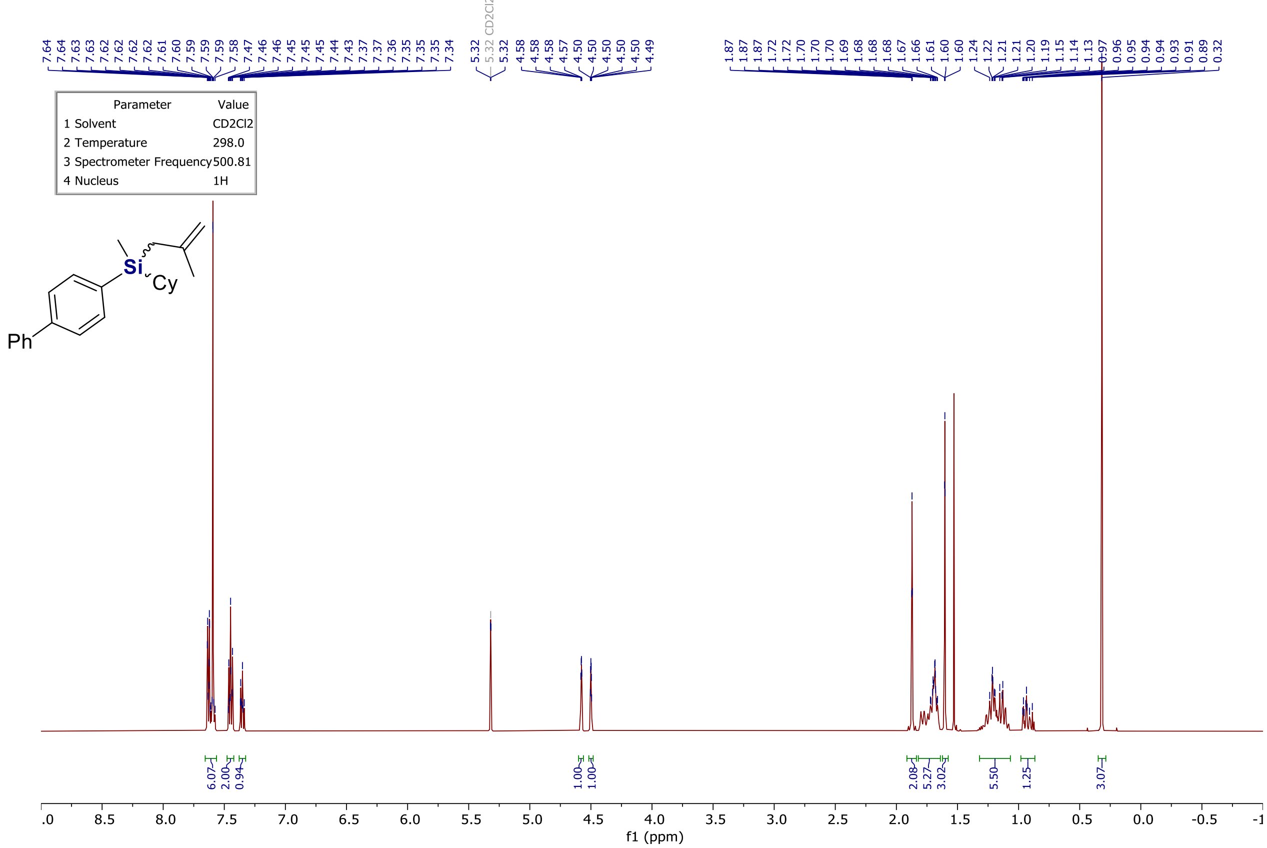

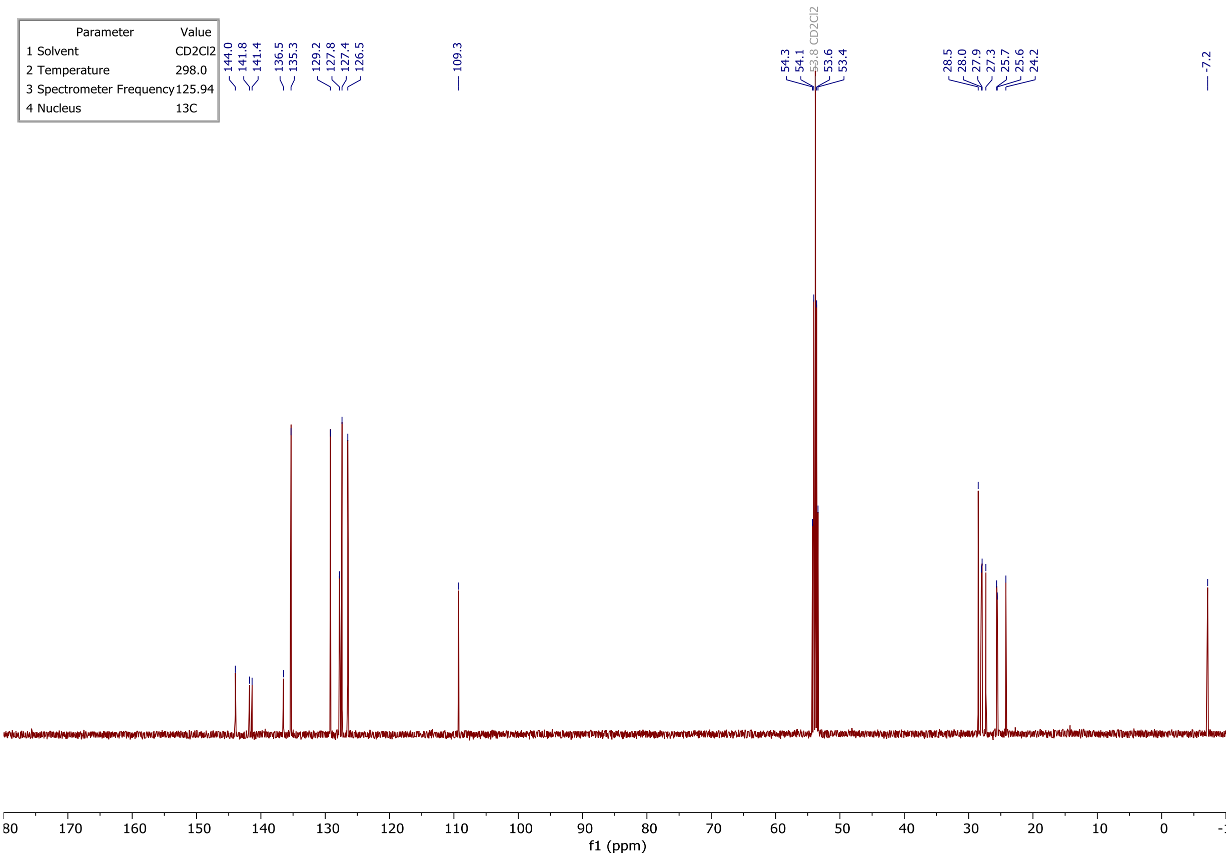

(*S*)-[1,1'-biphenyl]-4-yl(cyclohexyl)(2,6-diisopropylphenoxy)(methyl)silane

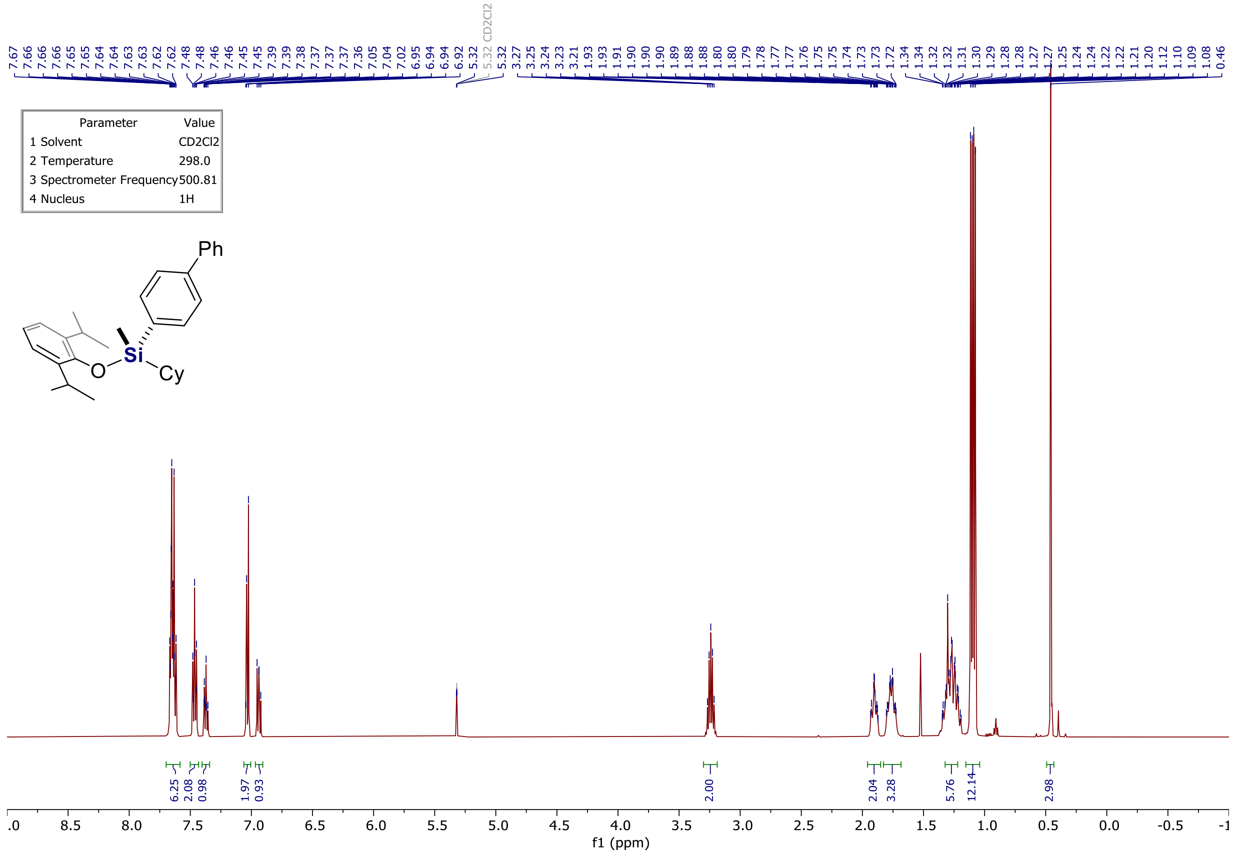

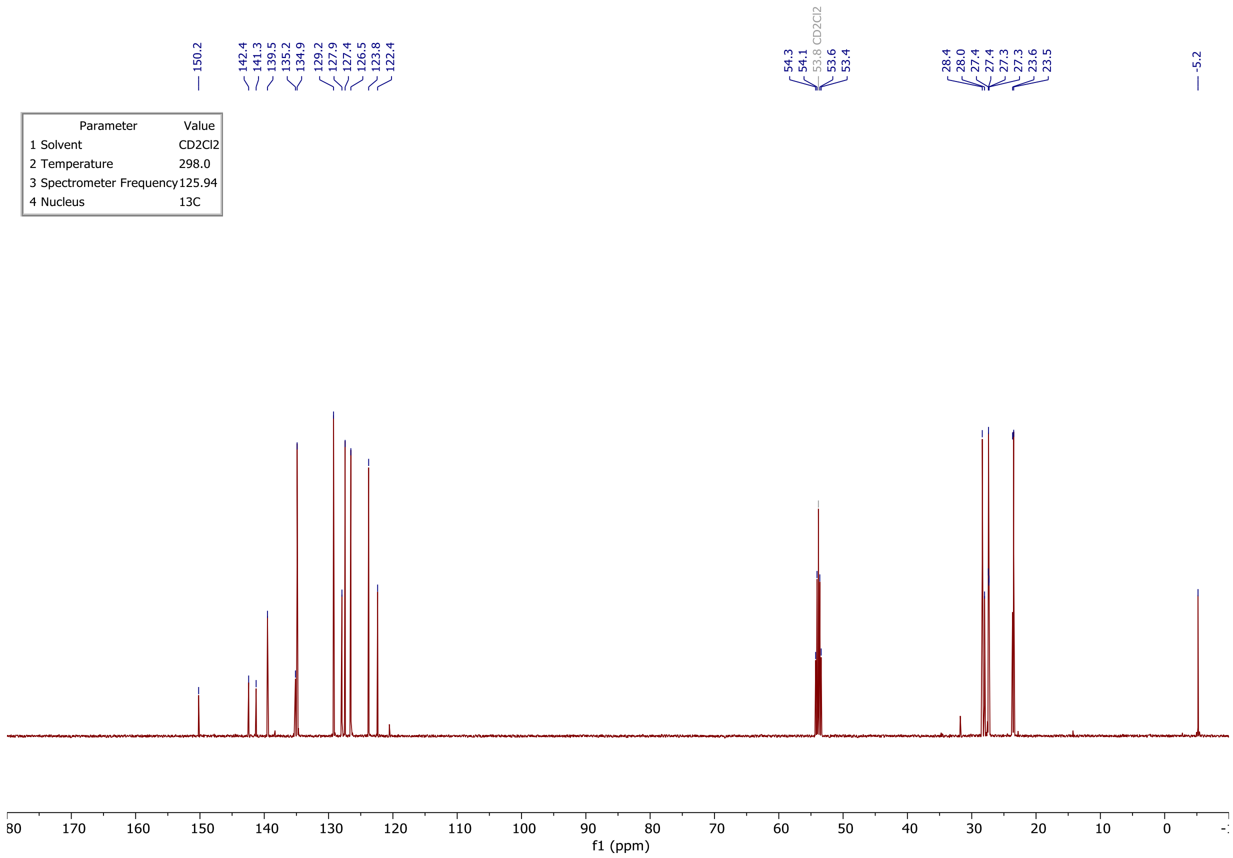

(*R*)-[1,1'-biphenyl]-4-yl(cyclohexyl)(methyl)silane

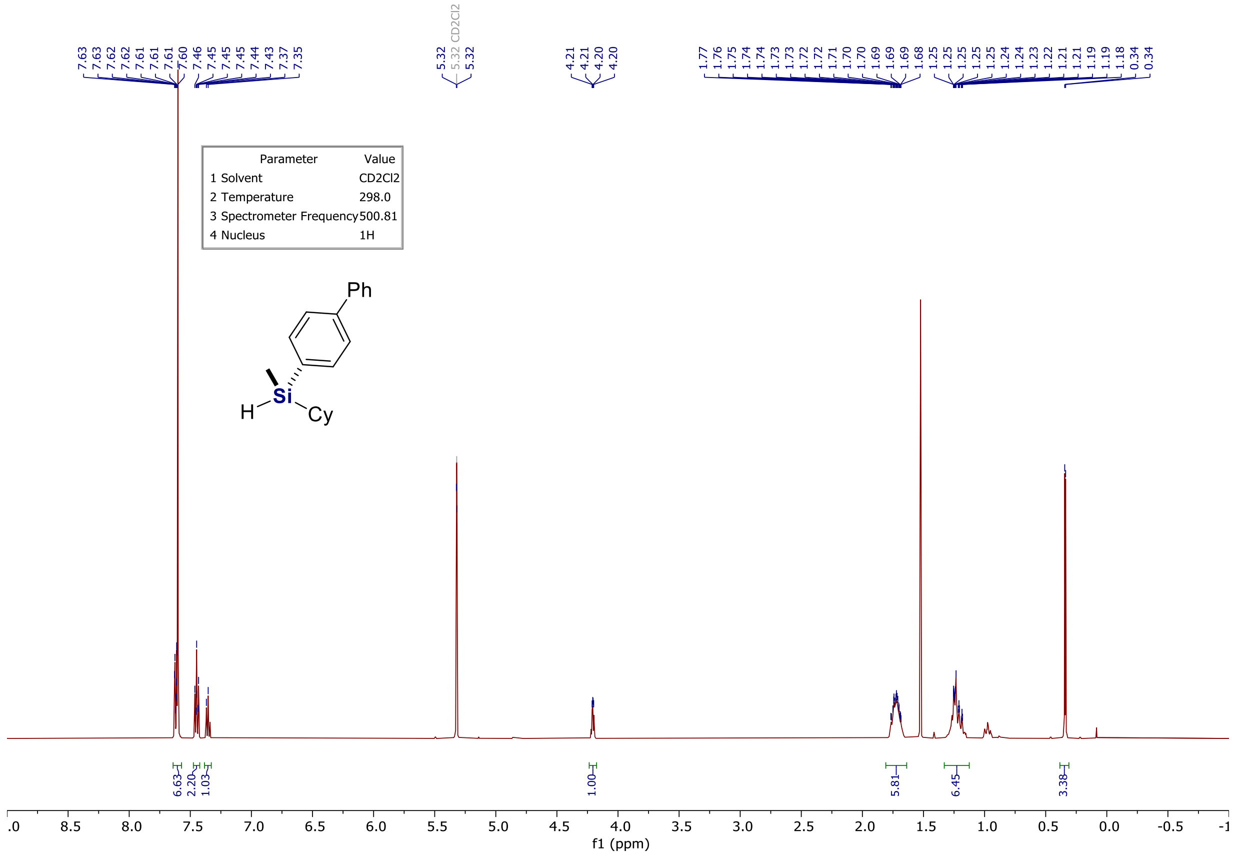

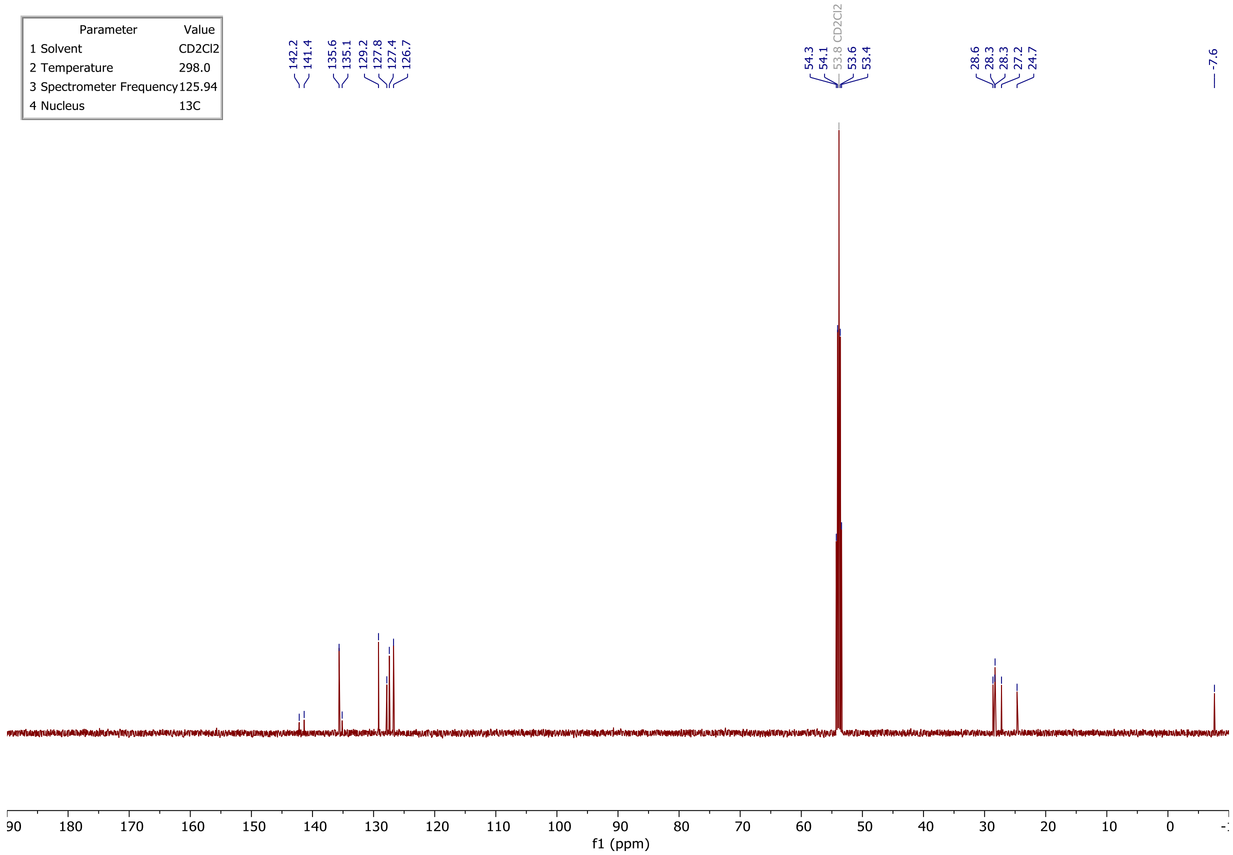

(*S*, *S*)-IDPi-3d

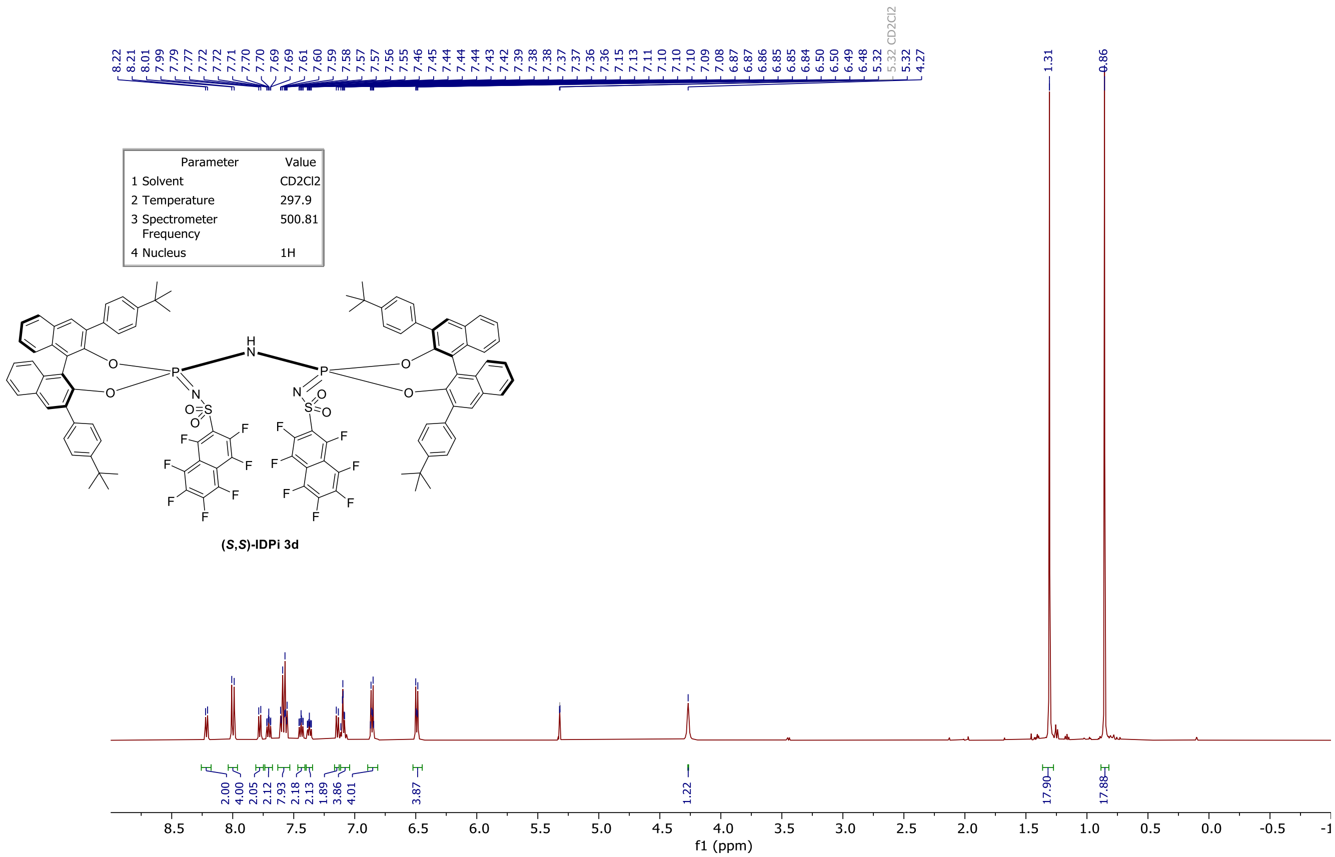
8.22 8.21 8.01 7.99 7.79 7.77 7.72 7.72 7.71 7.70 7.70 7.69 7.69 7.61 7.60 7.59 7.58 7.57 7.57 7.56 7.55 7.46 7.45 7.44 7.44 7.44 7.43 7.42 7.39 7.38 7.38 7.37 7.37 7.36 7.15 7.13 7.11 7.10 7.10 7.10 7.09 7.08 6.87 6.87 6.86 6.85 6.85 6.84 6.50 6.50 6.49 6.48 5.32 5.32 CD2Cl2 5.32 4.27
1.31
0.86
Parameter Value
1 Solvent CD2Cl2
2 Temperature 297.9
3 Spectrometer Frequency 500.81
4 Nucleus 1H
(S,S)-IDPi 3d
2.00 4.00 2.05 2.12 7.93 2.18 2.13 1.89 3.86 4.01 3.87 1.22 17.90 17.88
f1 (ppm)

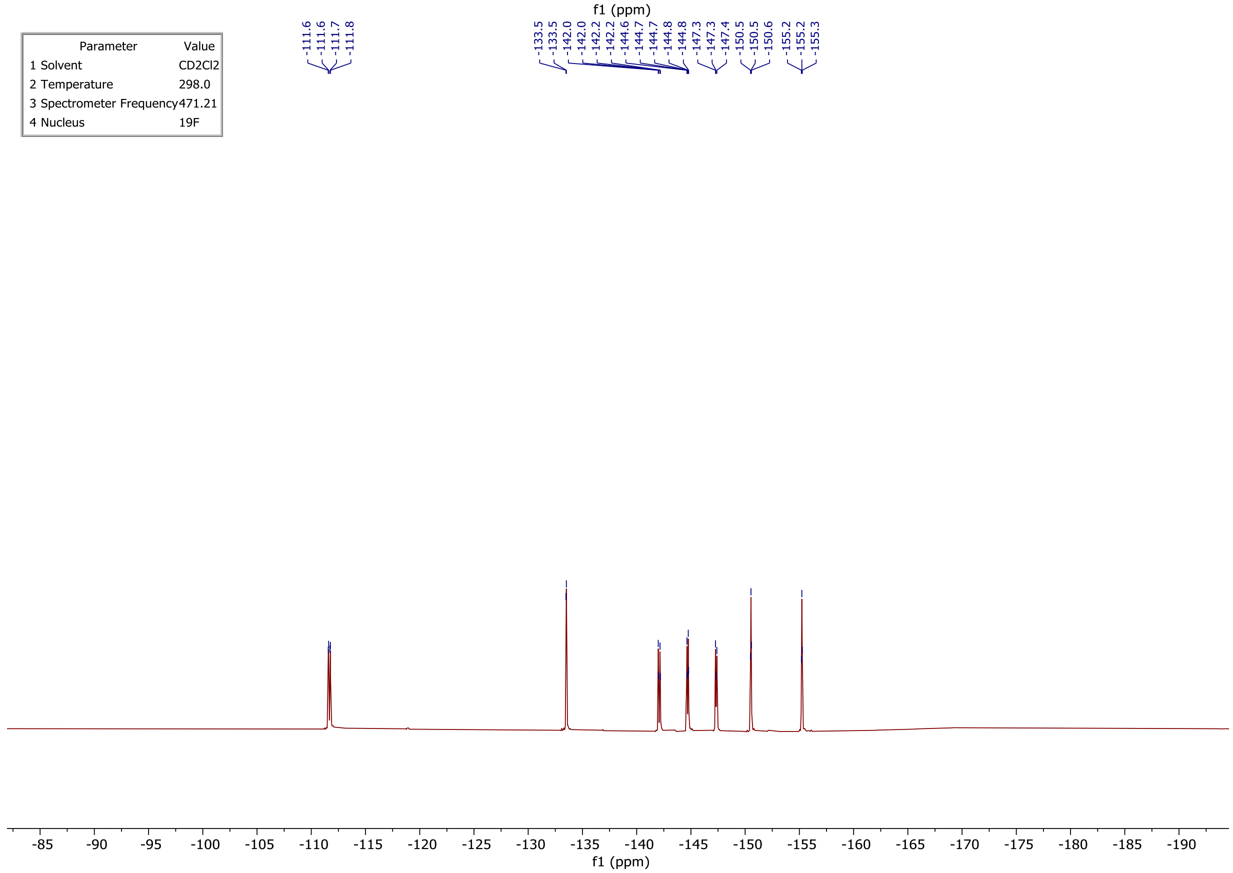
-111.6 -111.6 -111.7 -111.8
-133.5 -133.5 -142.0 -142.0 -142.2 -142.2 -142.2 -144.6 -144.7 -144.7 -144.8 -144.8 -147.3 -147.3 -147.4 -150.5 -150.5 -150.6 -155.2 -155.2 -155.3
Parameter Value
1 Solvent CD2Cl2
2 Temperature 298.0
3 Spectrometer Frequency 471.21
4 Nucleus 19F
f1 (ppm)

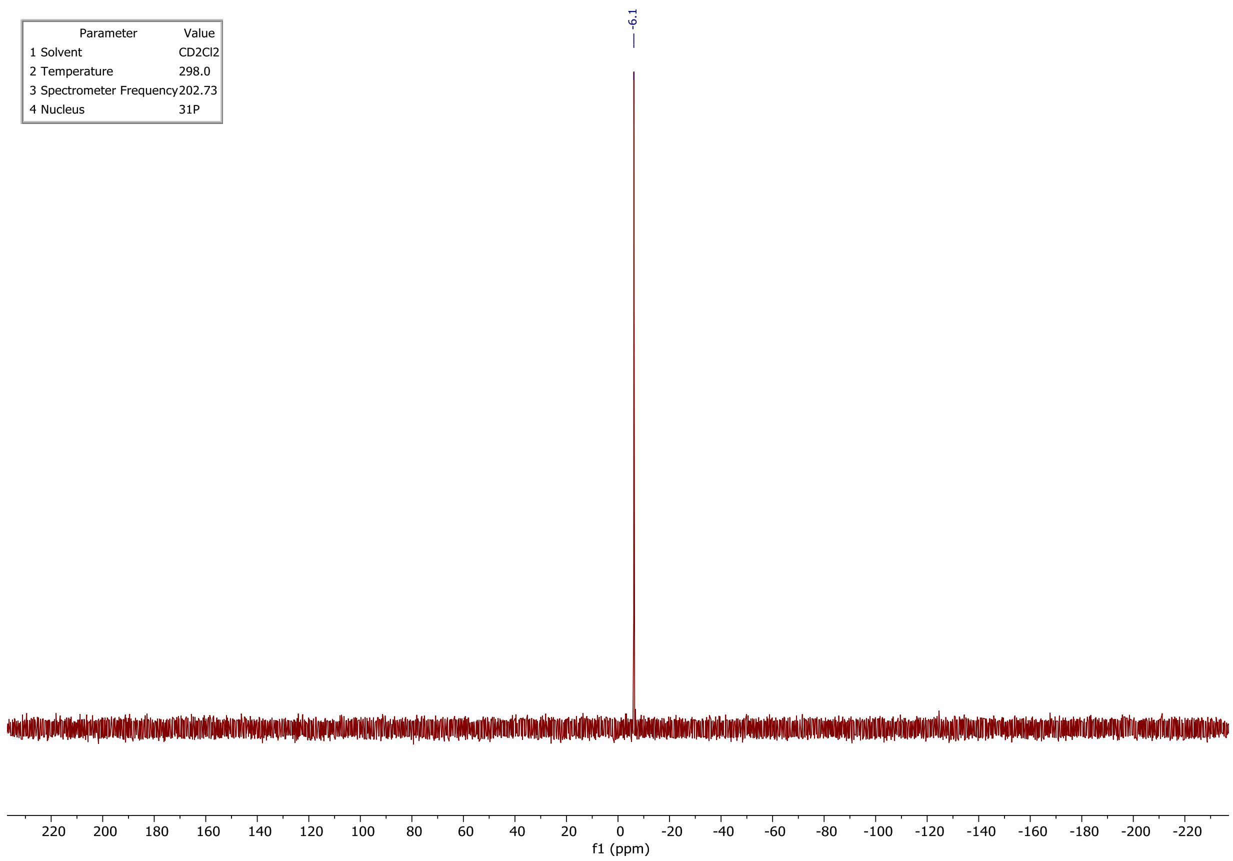

-6.1
Parameter Value
1 Solvent CD2Cl2
2 Temperature 298.0
3 Spectrometer Frequency 202.73
4 Nucleus 31P
f1 (ppm)

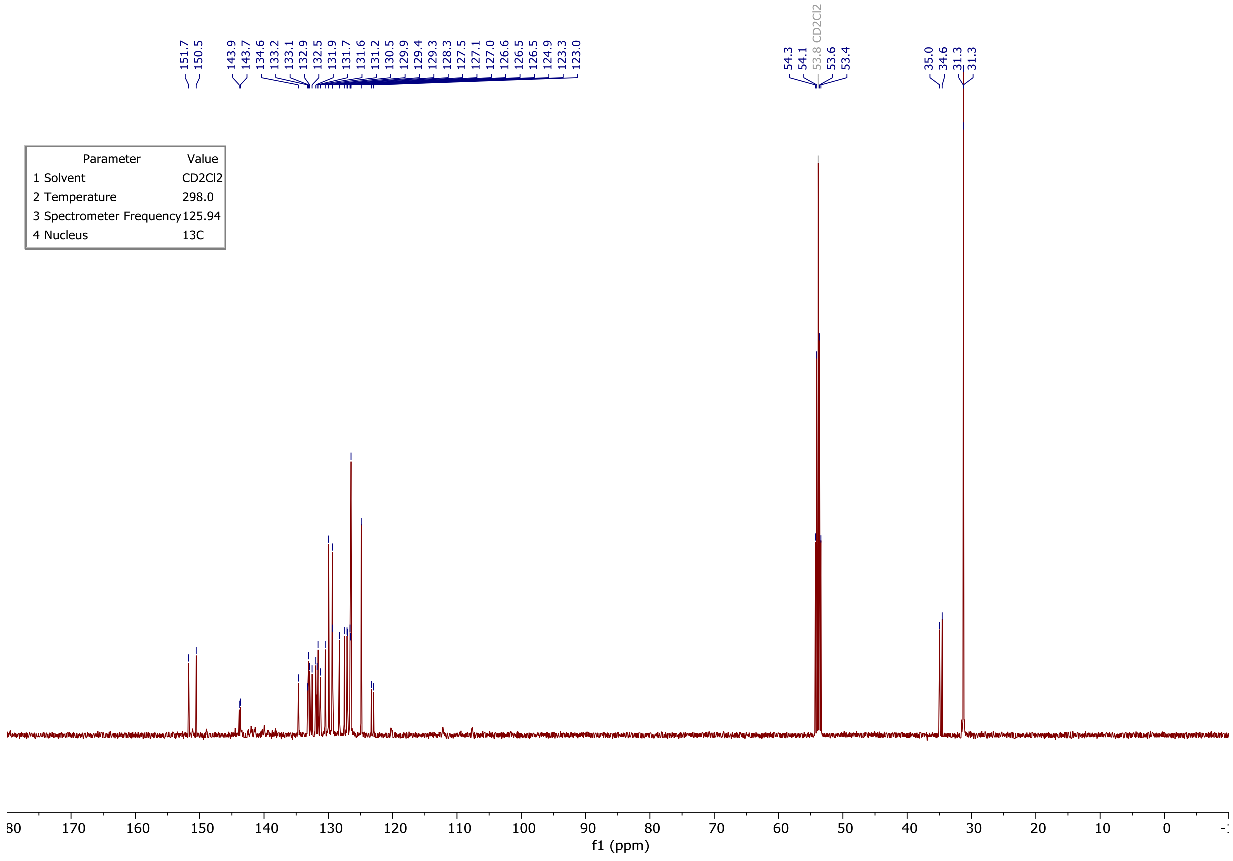

151.7
150.5
143.9
143.7
134.6
133.2
133.1
132.9
132.5
131.9
131.7
131.6
131.2
130.5
129.9
129.4
129.3
128.3
127.5
127.1
127.0
126.6
126.5
126.5
124.9
123.3
123.0
54.3
54.1
53.8 CD2Cl2
53.6
53.4
35.0
34.6
31.3
31.3
Parameter Value
1 Solvent CD2Cl2
2 Temperature 298.0
3 Spectrometer Frequency 125.94
4 Nucleus 13C
f1 (ppm)

11. Copies of HPLC traces

HPLC column: OJ-3R, MeOH : H_2O = 80:20, 1 mL/min, 298 K, 220 nm.

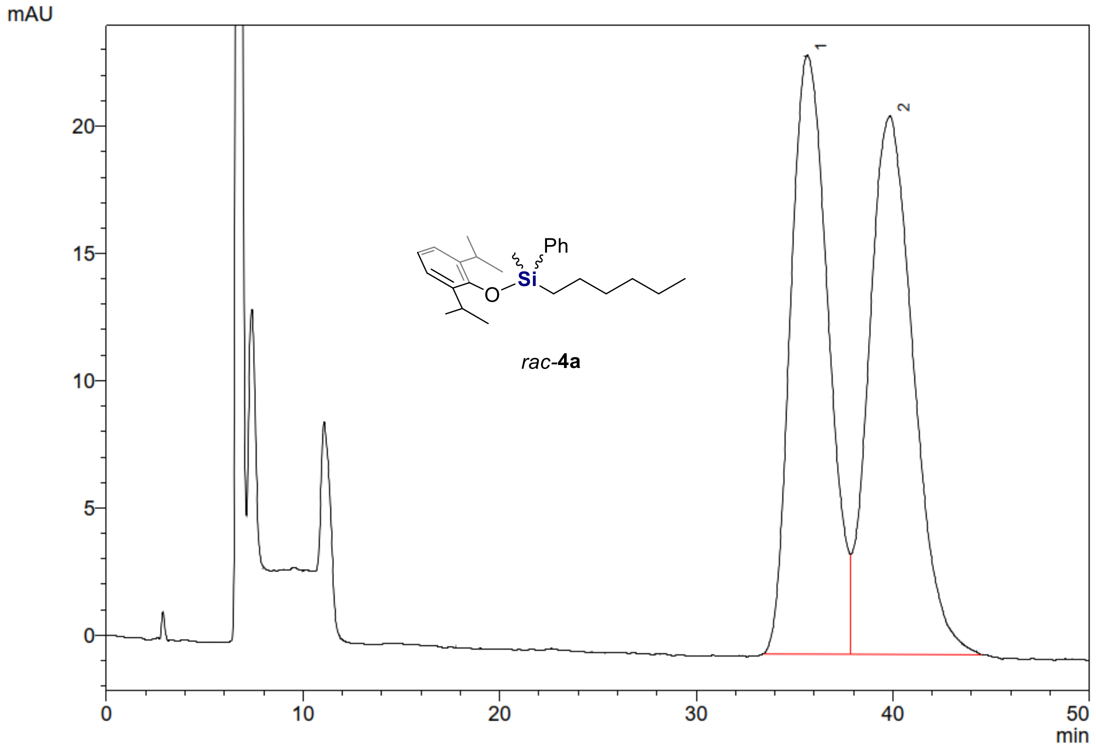

Peak #	t_R/min	% peak area
1	35.6	48.40
2	39.9	51.60
Total		100

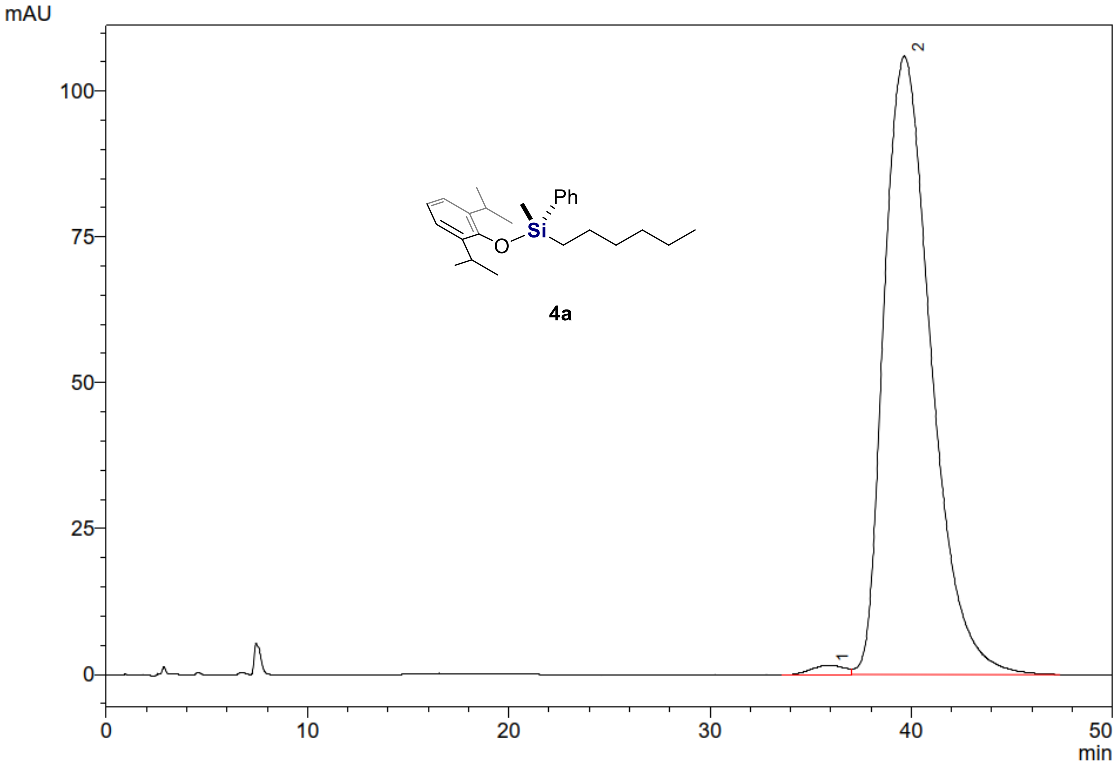

Peak #	t_R/min	% peak area
1	35.9	1.03
2	39.7	98.97
Total		100

For gram-scale reaction, the retention time changed significantly, thus the corresponding racemate was measured again, and the traces were shown below:

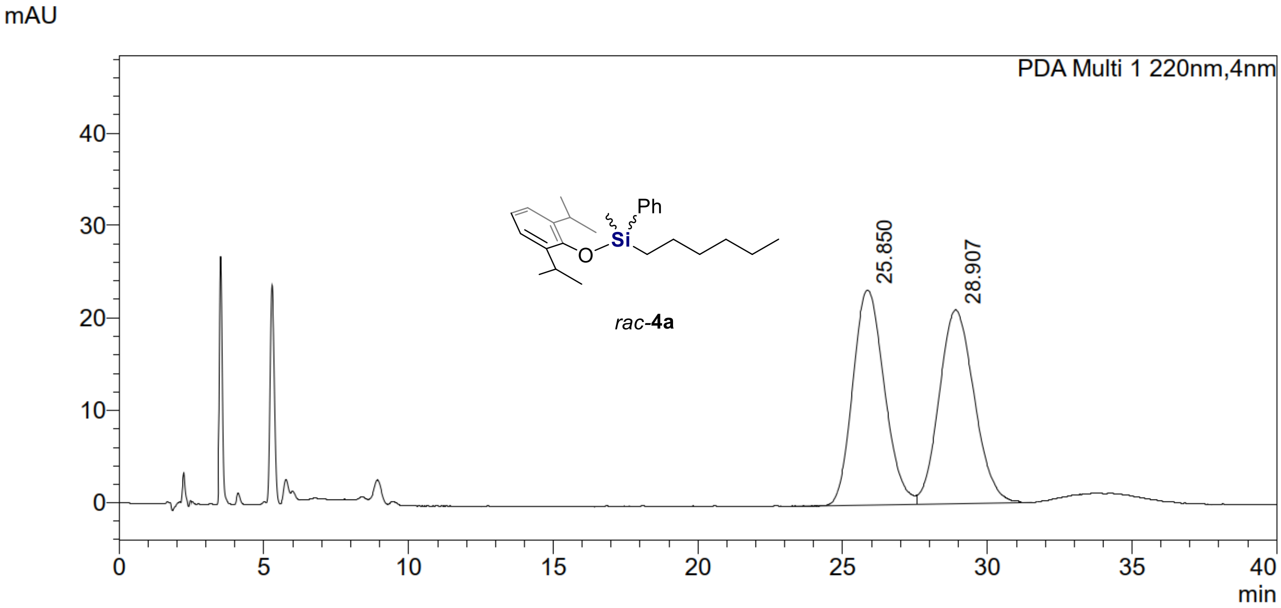

Peak #	t_R/min	% peak area
1	25.8	49.97
2	28.9	50.03
Total		100

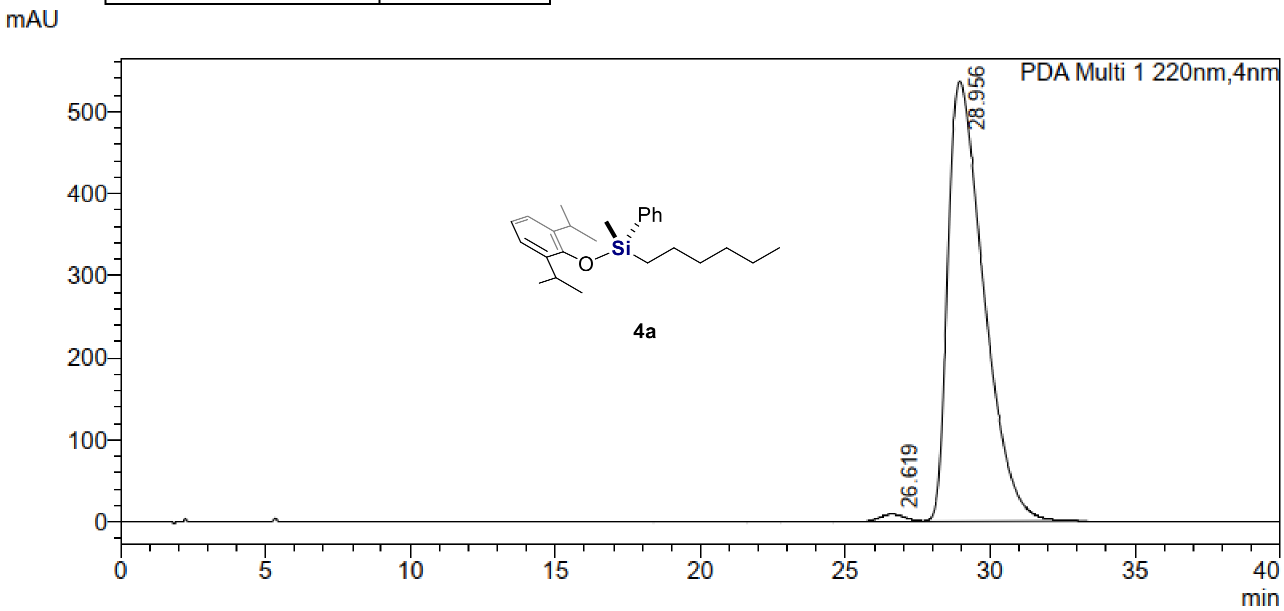

Peak #	t_R/min	% peak area
1	26.6	1.22
2	29.0	98.78
Total		100

HPLC column: OJ-3R, Acetonitrile/Water = 70:30, 1 mL/min, 298 K, 220 nm.

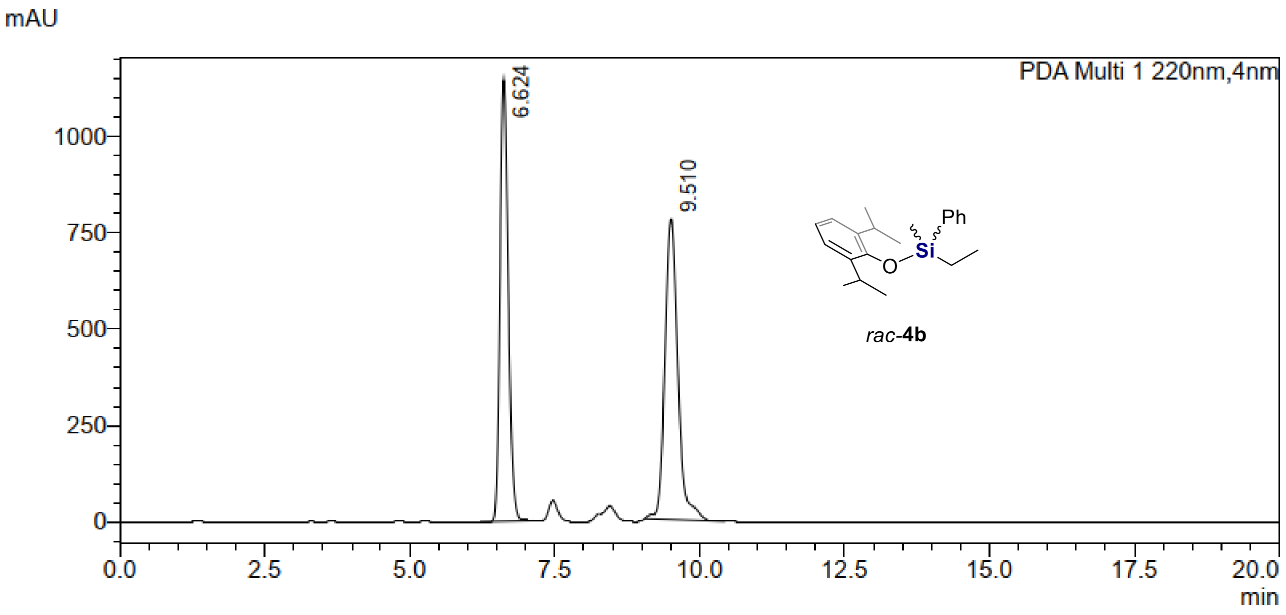

Peak #	t_R/min	% peak area
1	6.6	48.22
2	9.5	51.78
Total		100

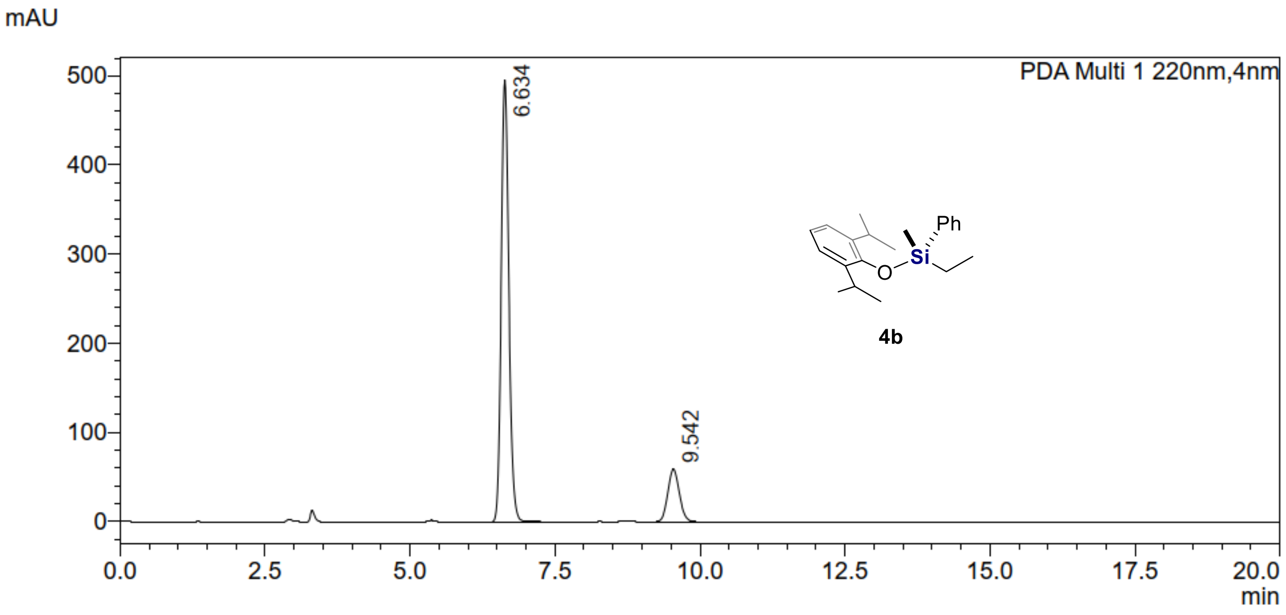

Peak #	t_R/min	% peak area
1	6.6	84.78
2	9.5	15.22
Total		100

HPLC column: OJ-3R, MeOH : H_2O = 80:20, 1 mL/min, 298 K, 220 nm.

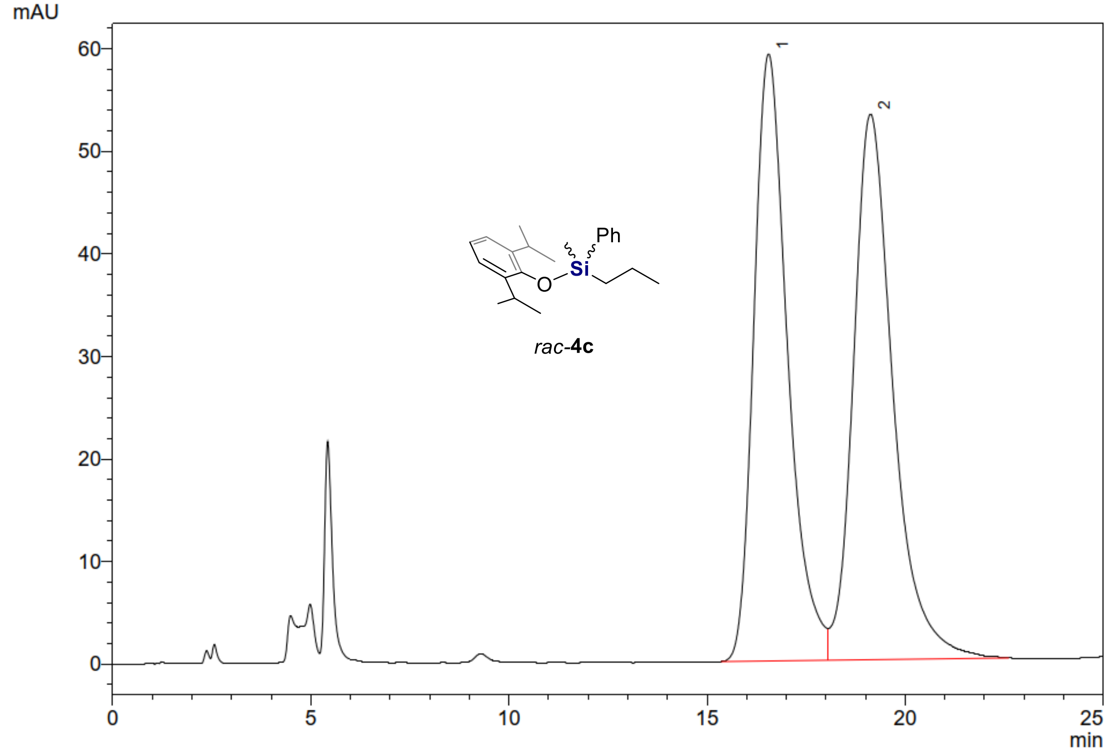

Peak #	t_R/min	% peak area
1	16.6	48.66
2	19.1	51.34
Total		100

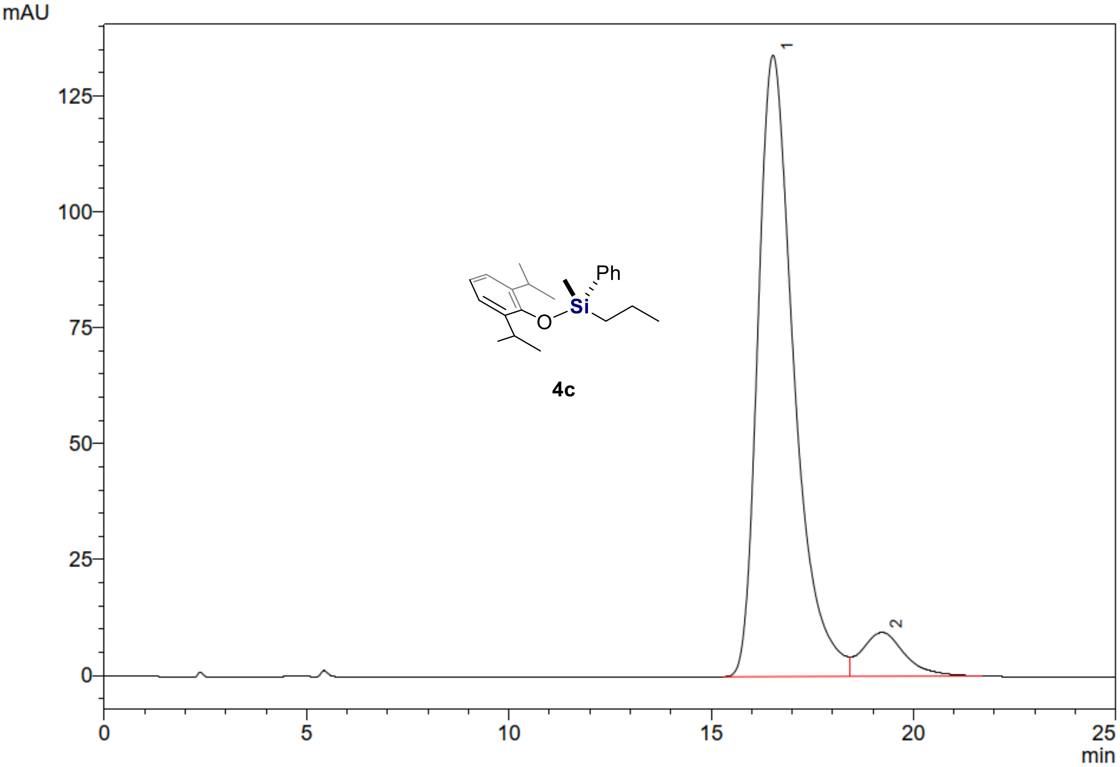

Peak #	t_R/min	% peak area
1	16.5	92.18
2	19.2	7.82
Total		100

HPLC column: OJ-3R, Acetonitrile/Water = 60:40, 1.0 mL/min, 298 K, 220 nm.

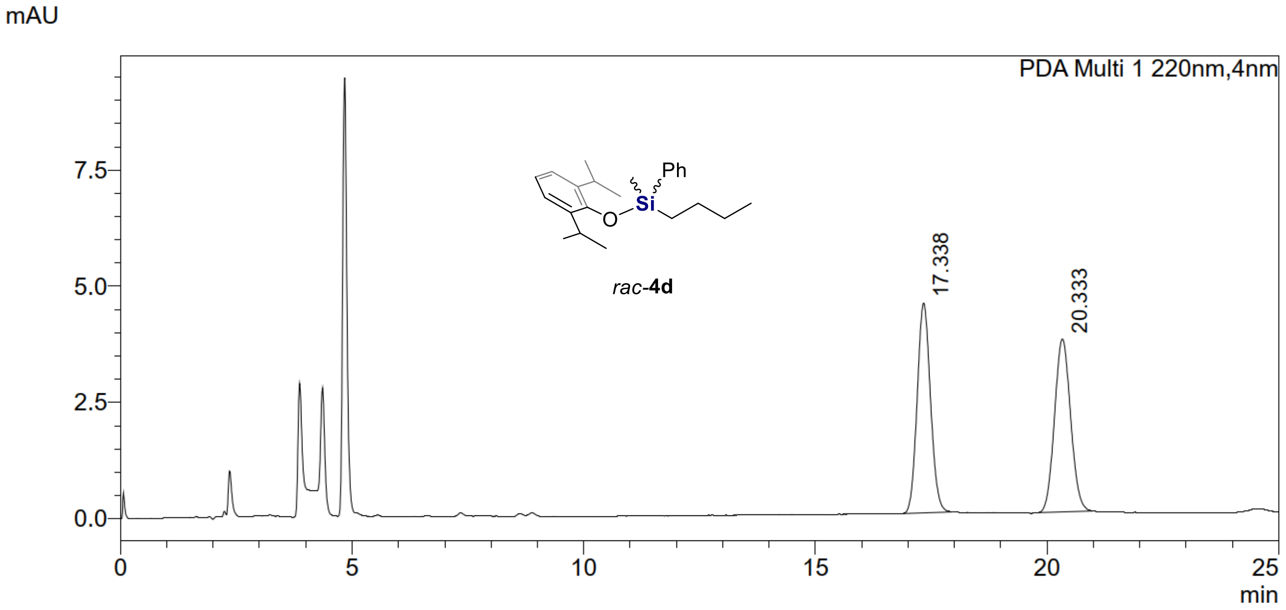

Peak #	t_R/min	% peak area
1	17.3	50.11
2	20.3	49.89
Total		100

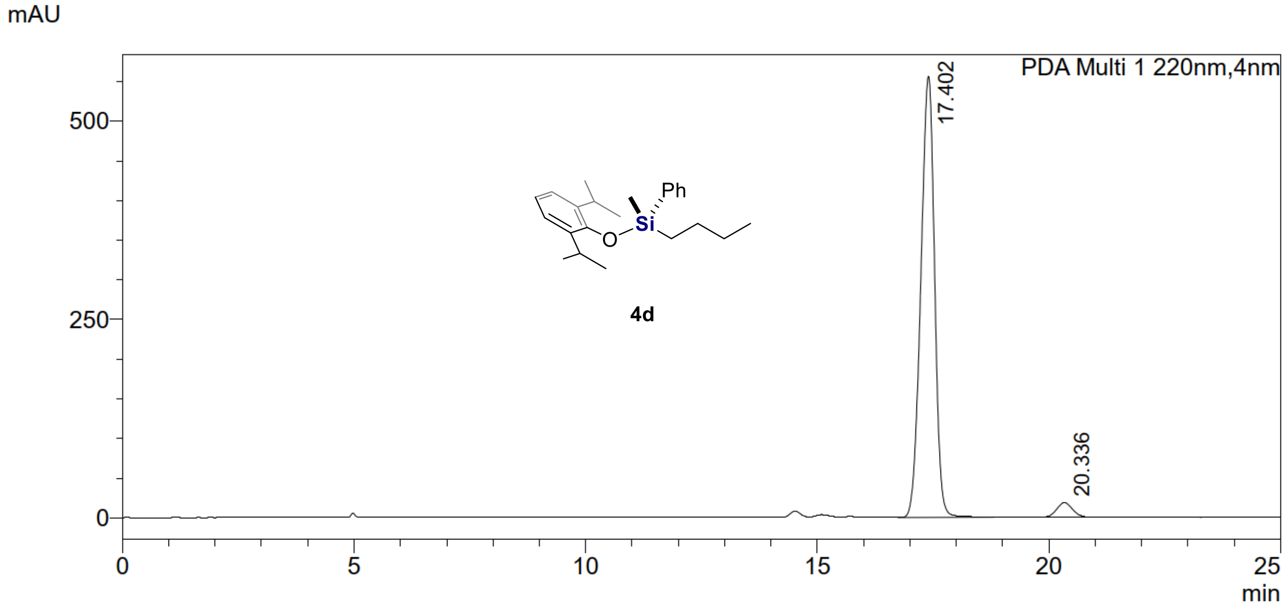

Peak #	t_R/min	% peak area
1	17.4	96.24
2	20.3	3.76
Total		100

HPLC column: OJ-3R, Acetonitrile/Water = 60:40, 1.0 mL/min, 298 K, 220 nm.

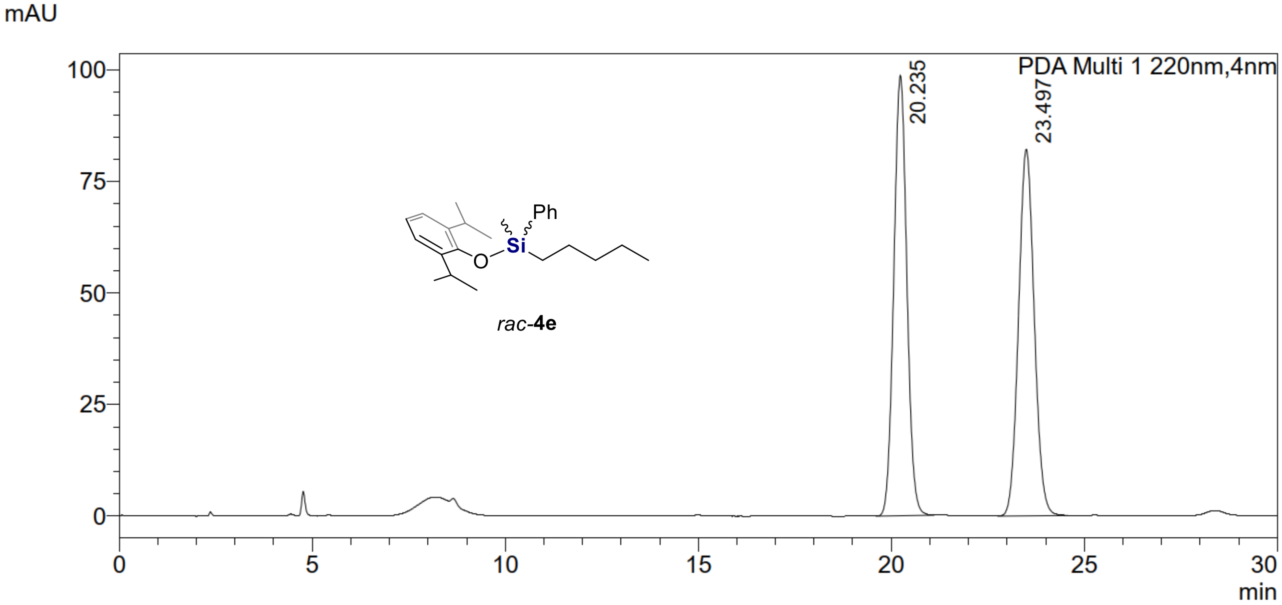

Peak #	t_R/min	% peak area
1	20.2	49.90
2	23.5	50.10
Total		100

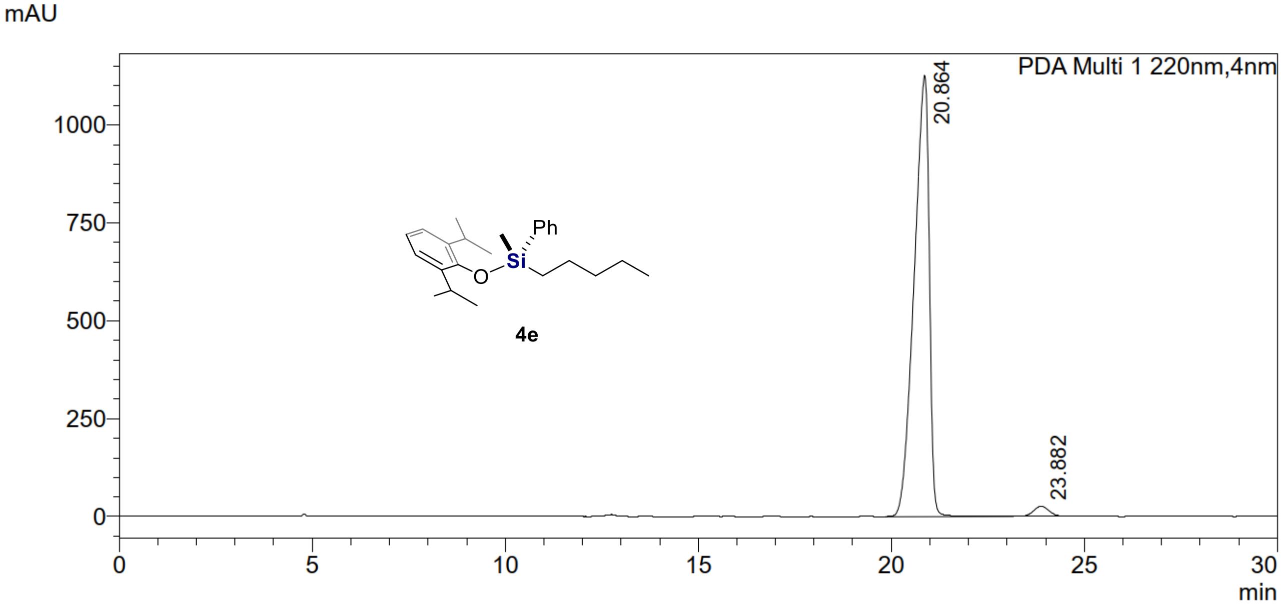

Peak #	t_R/min	% peak area
1	20.9	97.62
2	23.9	2.38
Total		100

HPLC column: OJ-3R, MeOH : H_2O = 80:20, 1 mL/min, 298 K, 220 nm.

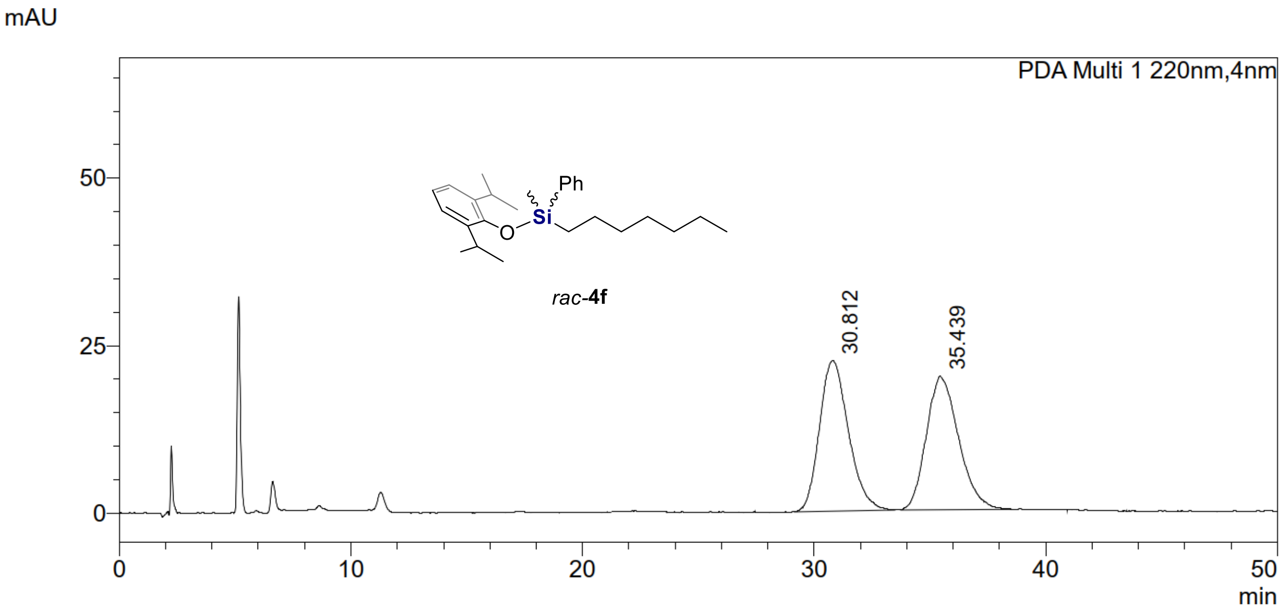

Peak #	t_R/min	% peak area
3	30.8	50.31
4	35.4	49.69
Total		100

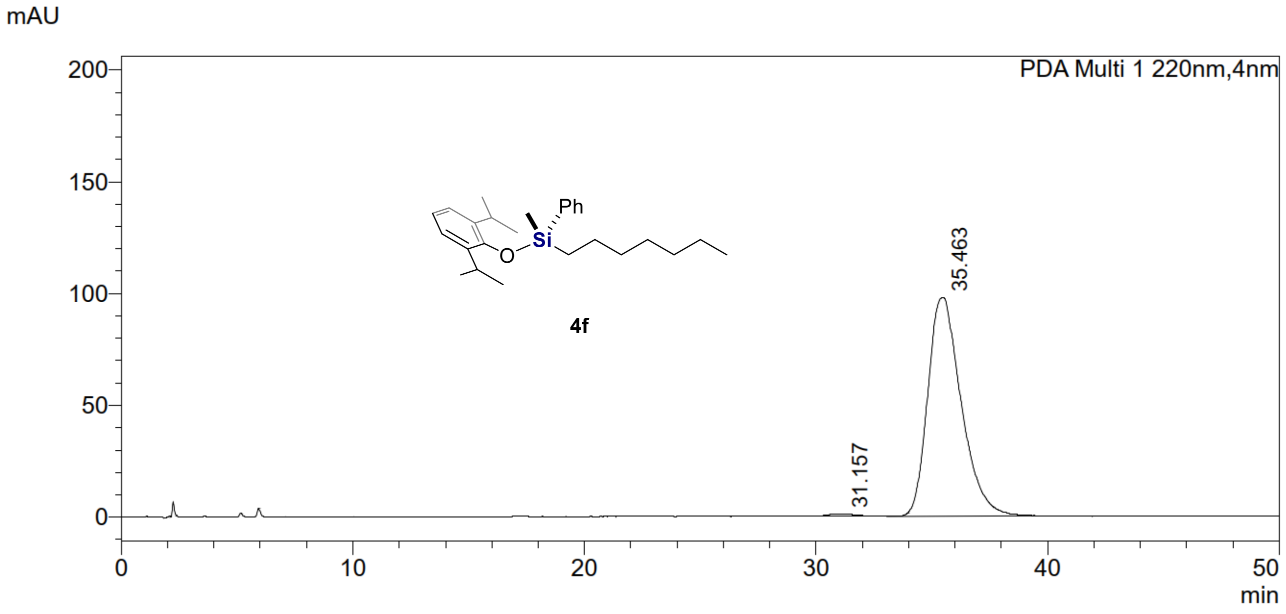

Peak #	t_R/min	% peak area
11	31.2	0.91
12	35.5	99.09
Total		100

HPLC column: OJ-3R, Acetonitrile/Water = 60:40, 1.0 mL/min, 298 K, 220 nm.

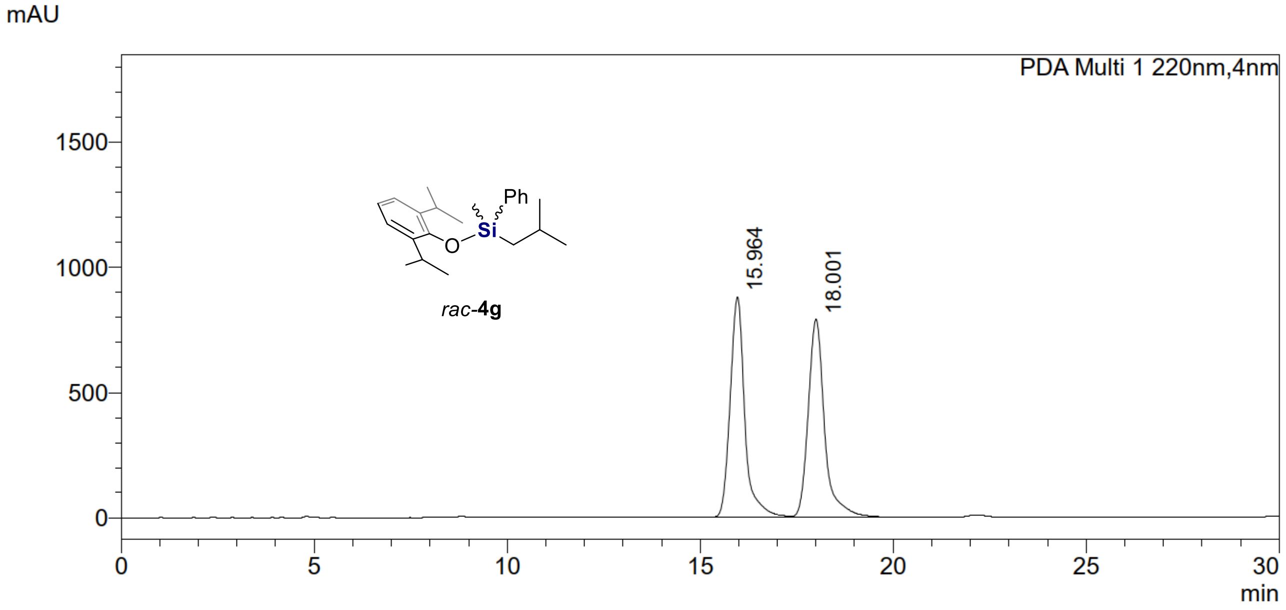

Peak #	t_R/min	% peak area
1	16.0	49.82
2	18.0	50.18
Total		100

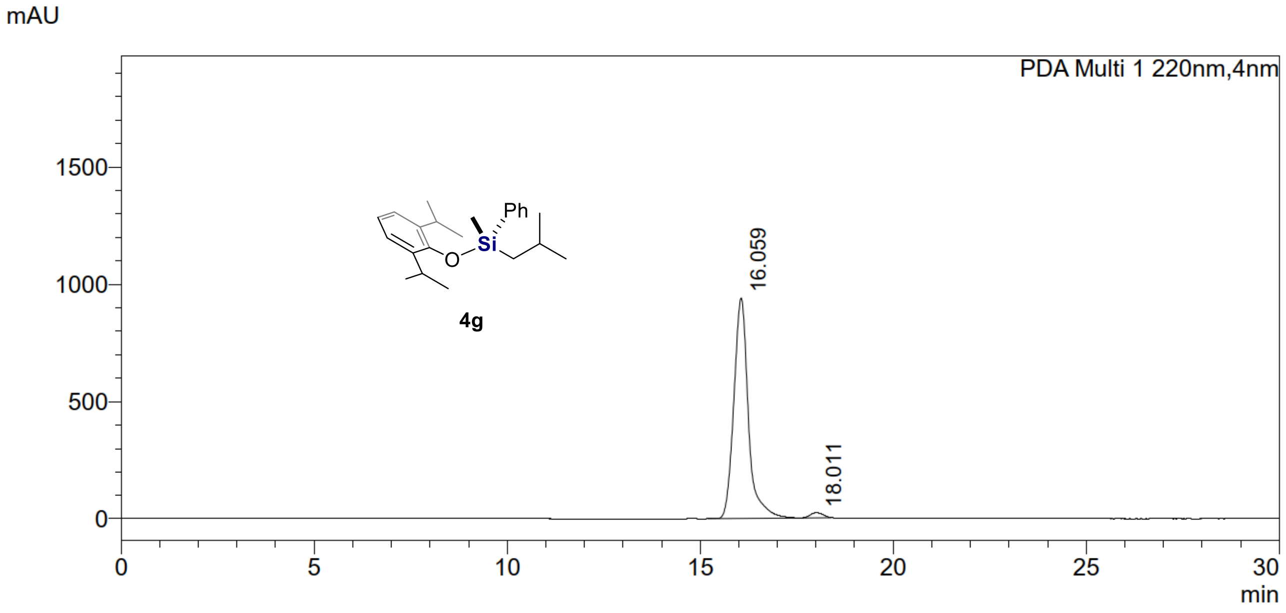

Peak #	t_R/min	% peak area
1	16.1	97.98
2	18.0	2.02
Total		100

HPLC column: OJ-3R, Acetonitrile/Water = 60:40, 1.0 mL/min, 298 K, 220 nm.

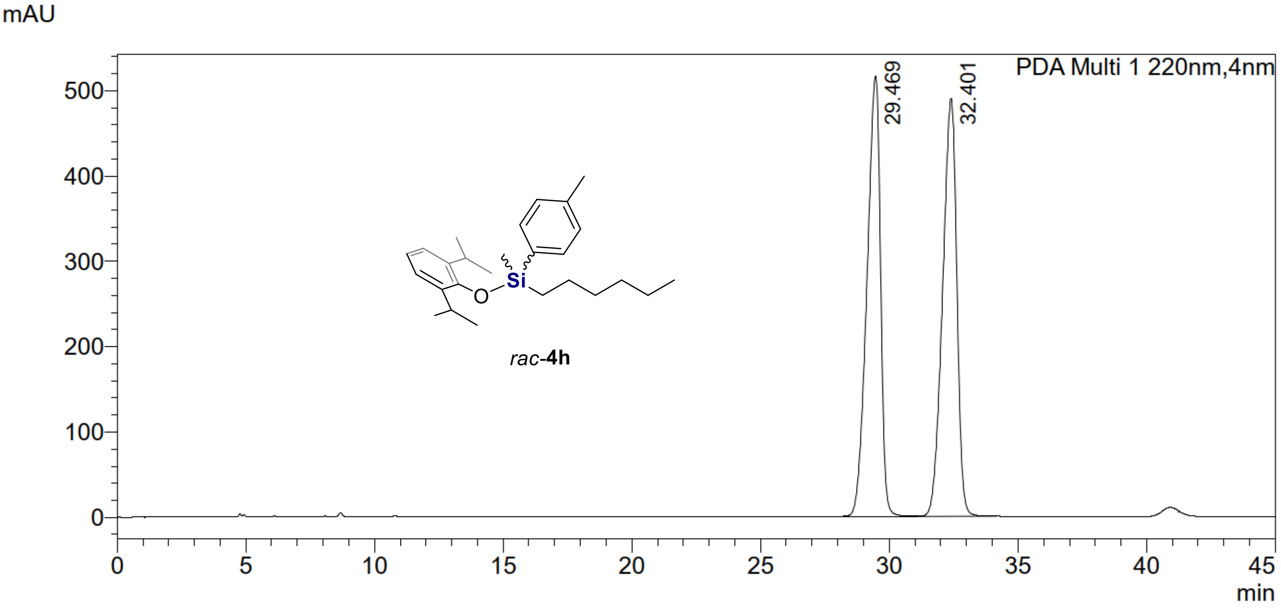

Peak #	t_R/min	% peak area
1	29.5	50.03
2	32.4	49.97
Total		100

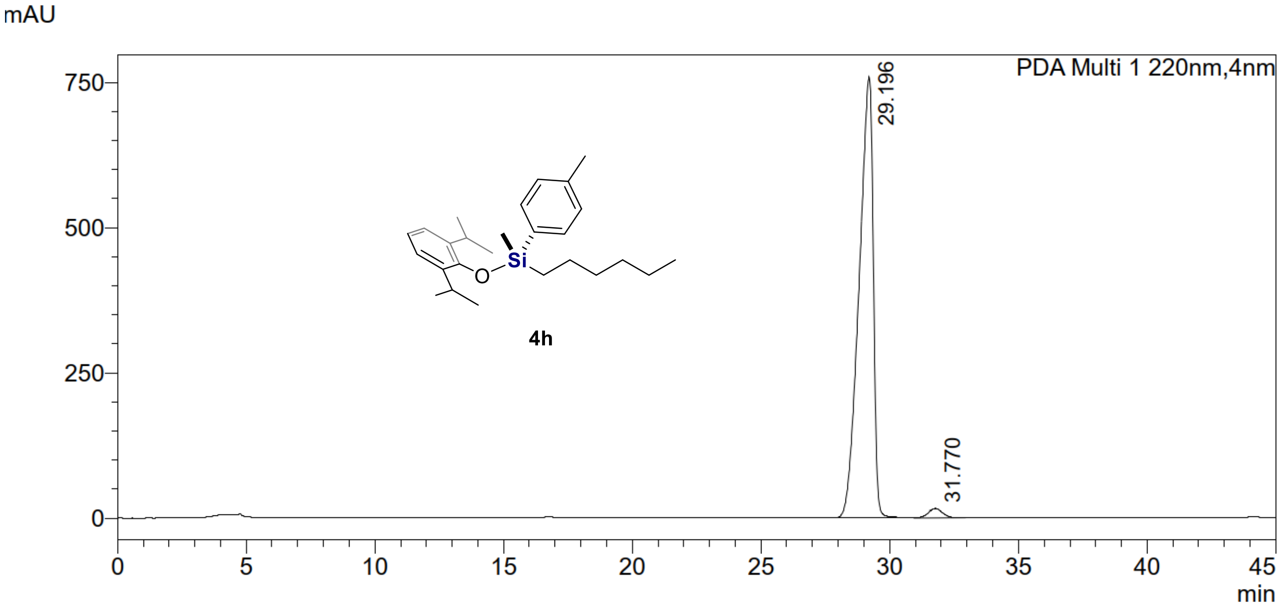

Peak #	t_R/min	% peak area
1	29.2	97.93
2	31.8	2.07
Total		100

HPLC column: OJ-3R, Acetonitrile/Water = 60:40, 1.0 mL/min, 298 K, 220 nm.

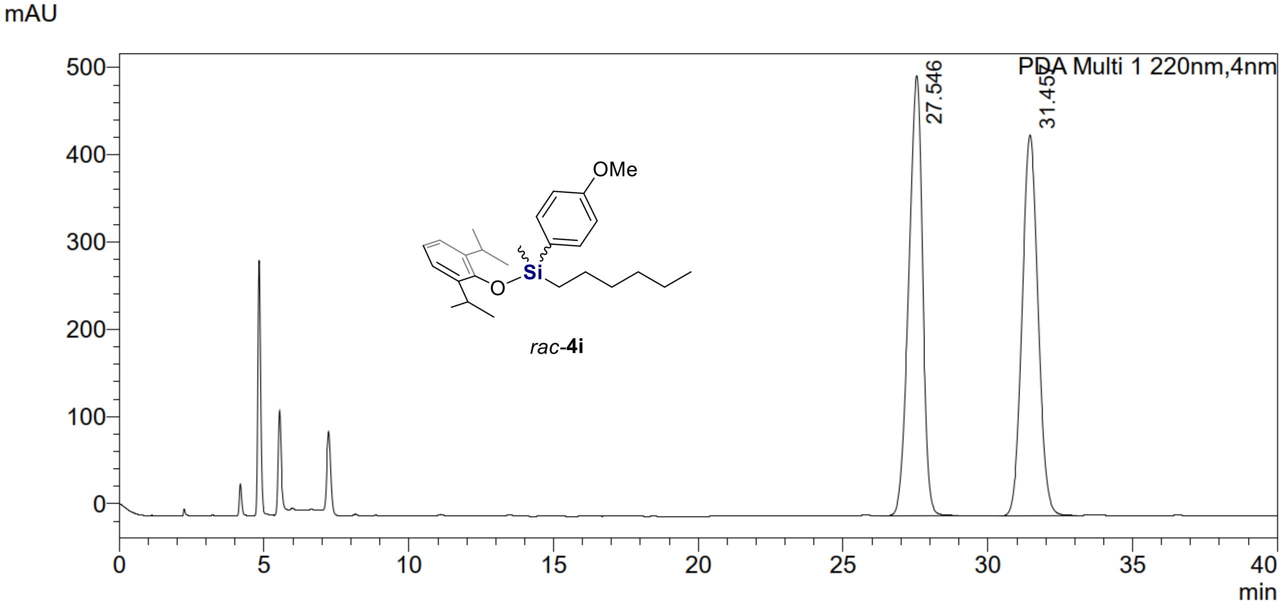

Peak #	t_R/min	% peak area
1	27.5	50.02
2	31.5	49.98
Total		100

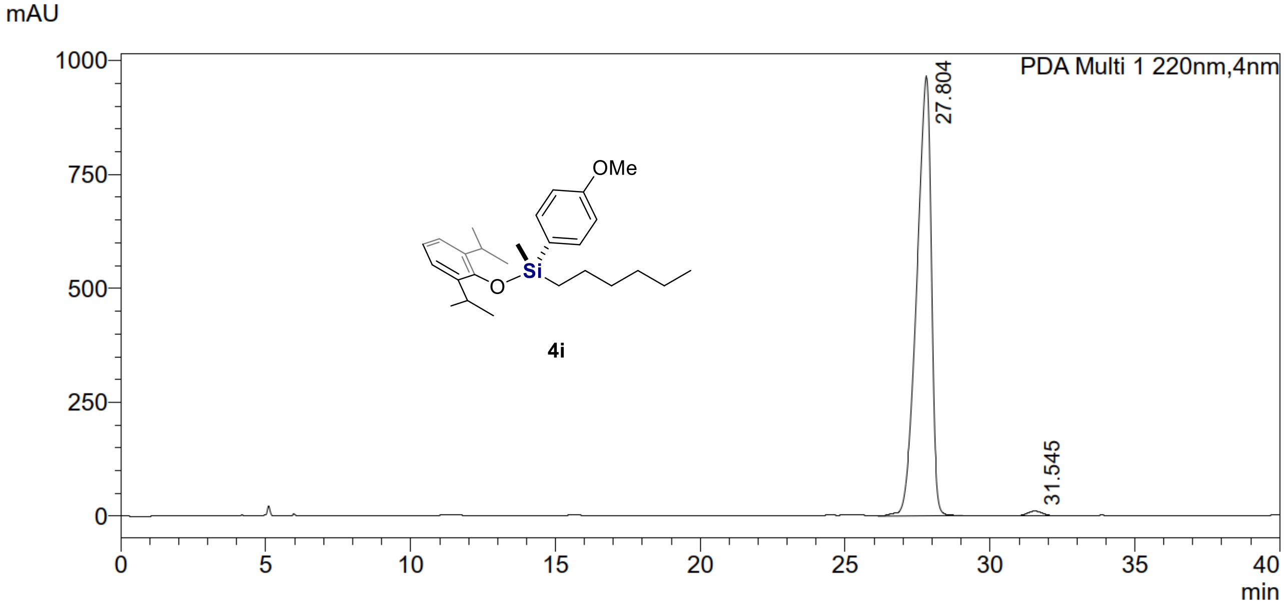

Peak #	t_R/min	% peak area
1	27.8	98.81
2	31.5	1.19
Total		100

HPLC column: OJ-3R, MeOH : H_2O = 80:20, 1 mL/min, 298 K, 220 nm.

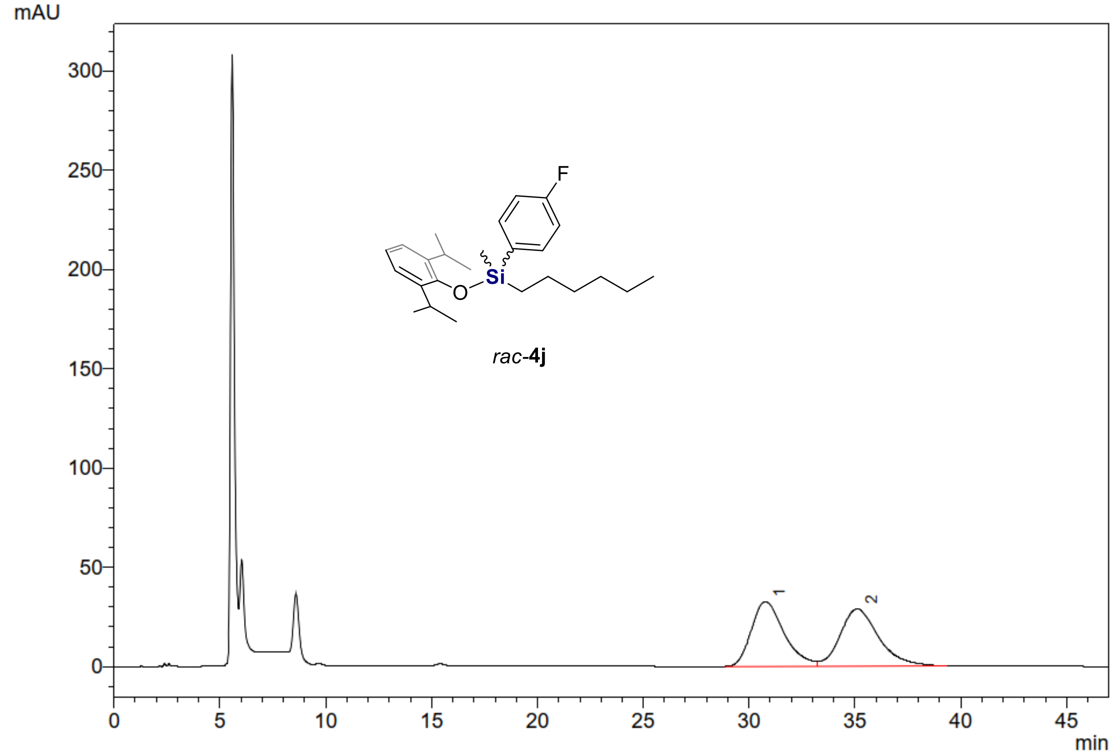

Peak #	t_R/min	% peak area
1	30.8	49.28
2	35.1	50.72
Total		100

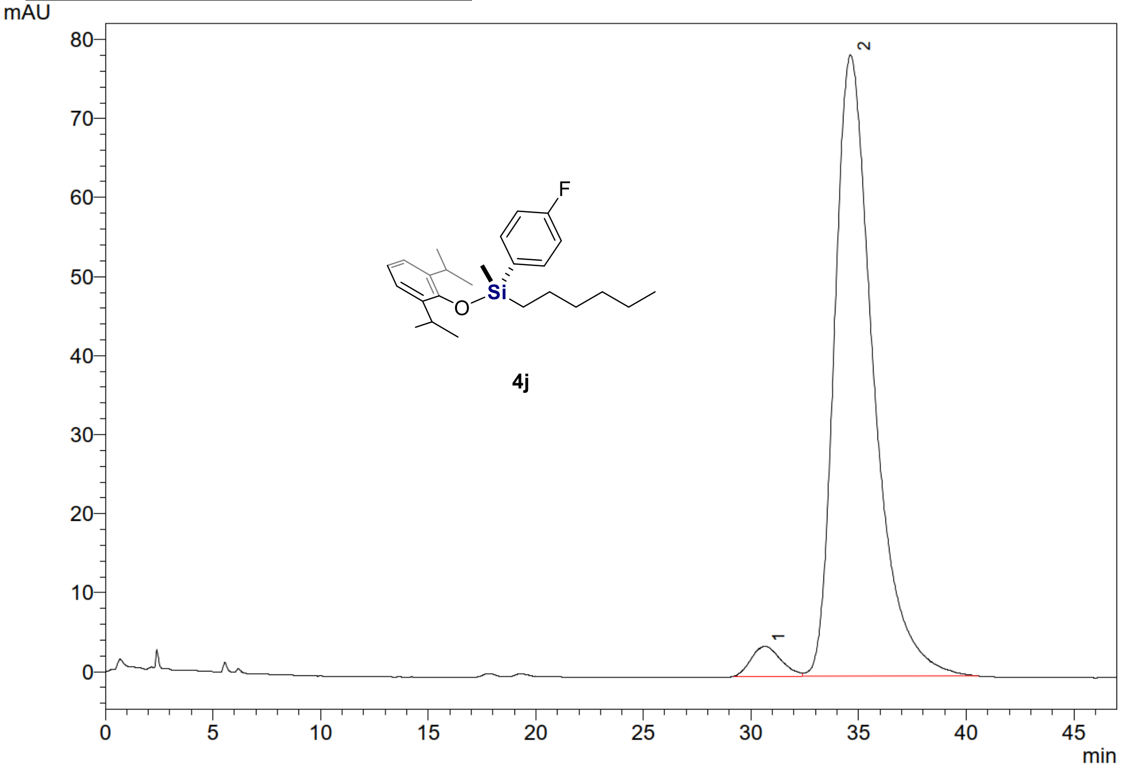

Peak #	t_R/min	% peak area
1	30.6	3.52
2	34.6	96.48
Total		100

HPLC column: OJ-3R, MeOH : H_2O = 80:20, 1 mL/min, 298 K, 220 nm.

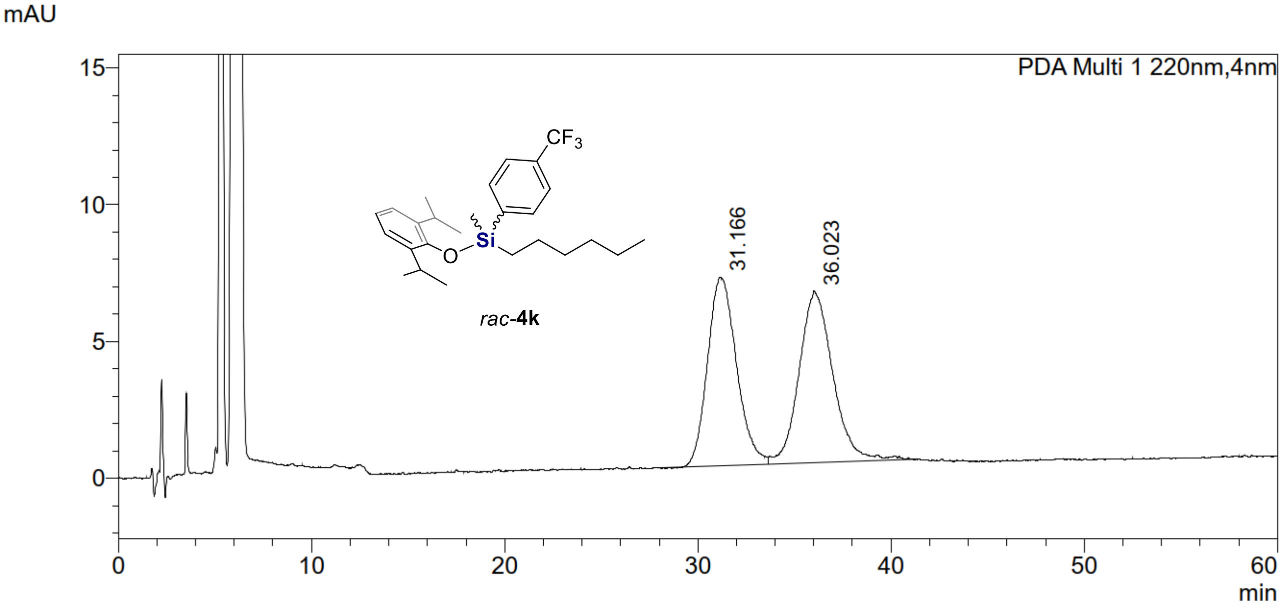

Peak #	t_R/min	% peak area
1	31.2	48.45
2	36.0	51.55
Total		100

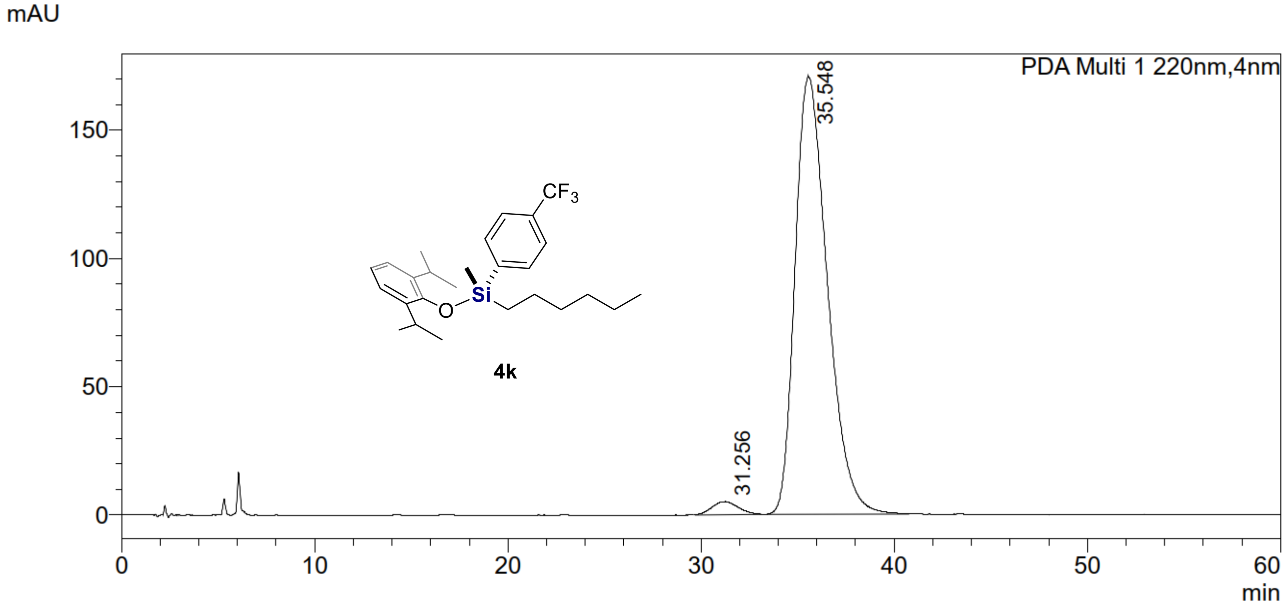

Peak #	t_R/min	% peak area
1	31.3	2.33
2	35.5	97.67
Total		100

HPLC column: IB-N3, CO_2, BP, 150 bar, 50 °C, 2.0 mL / min, 313 K, 19.0 MPa UV, 220 nm 0.5 mL/min 10mM NH_4AC in MeOH.

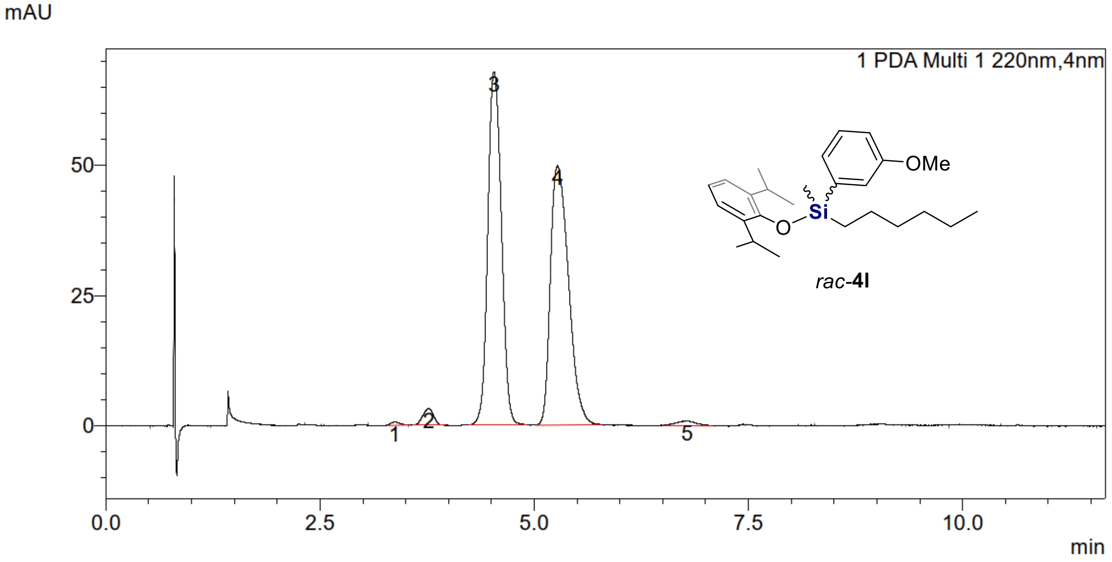

Peak #	t_R/min	% peak area
3	4.5	50.60
4	5.3	49.40
Total		100

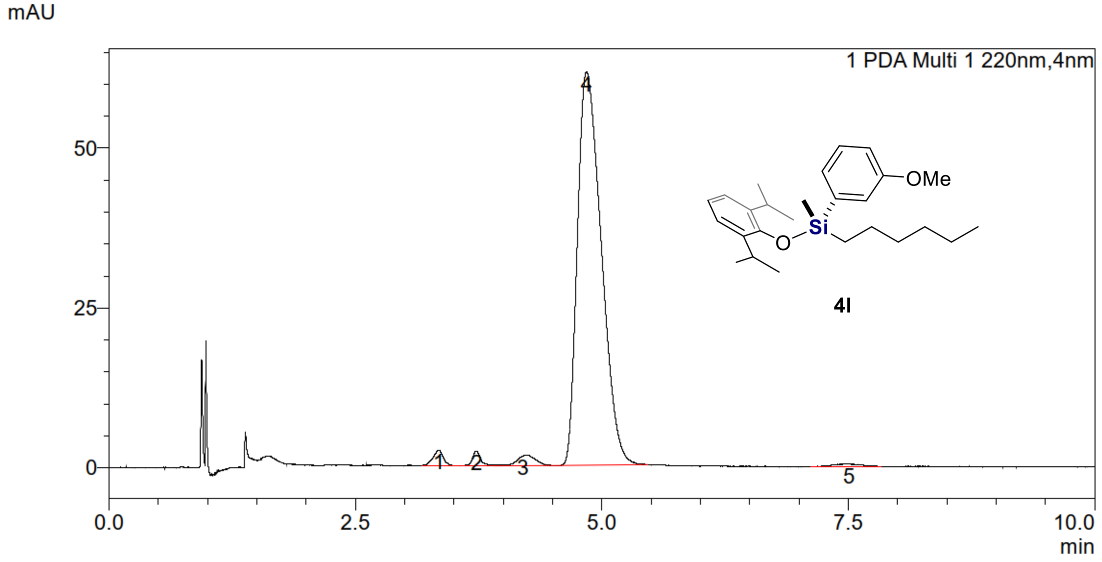

Peak #	t_R/min	% peak area
3	4.2	98.00
4	4.8	2.00
Total		100

HPLC column: OJ-3R, MeOH : H_2O = 80:20, 1 mL/min, 298 K, 220 nm.

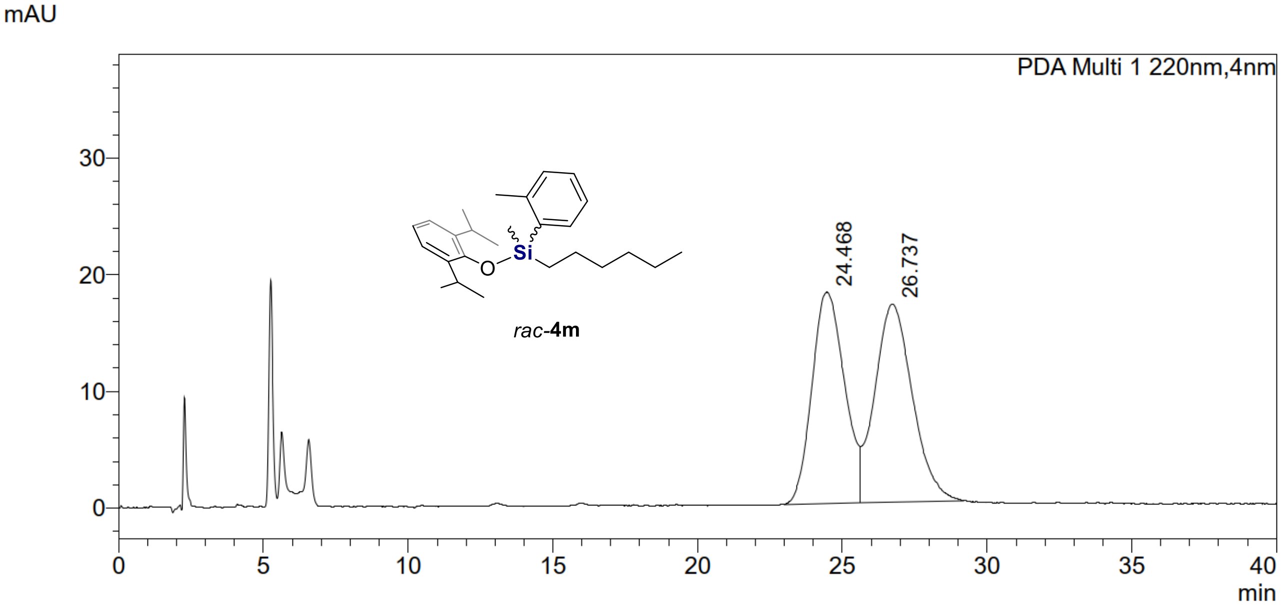

Peak #	t_R/min	% peak area
1	24.5	48.10
2	26.7	51.90
Total		100

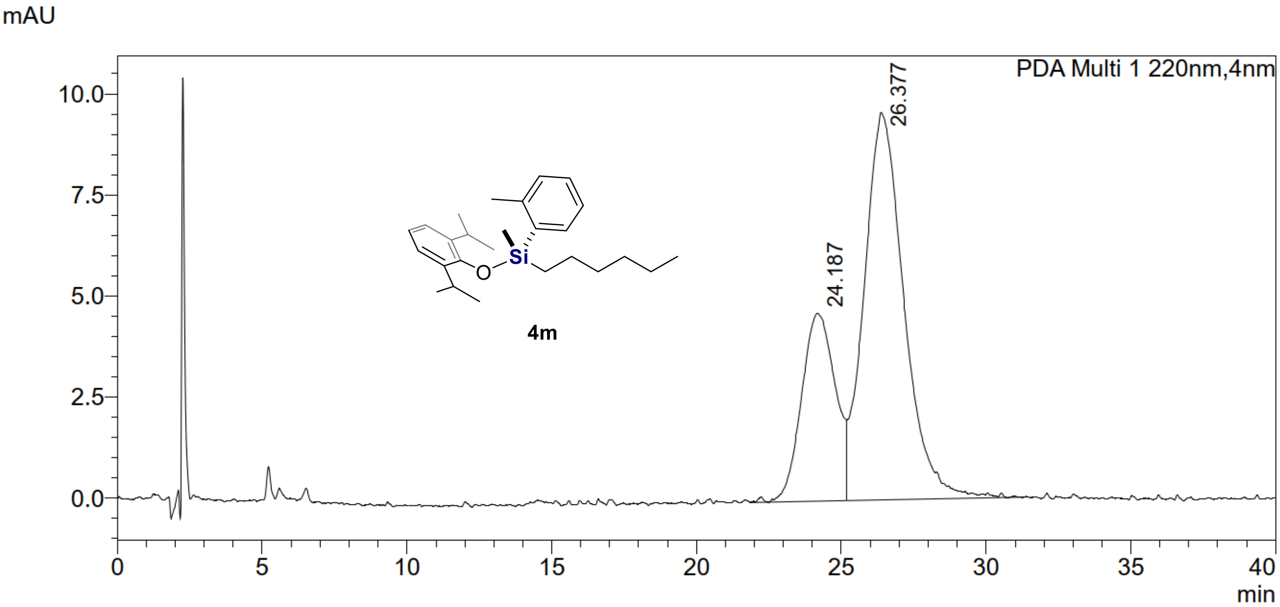

Peak #	t_R/min	% peak area
1	24.2	29.25
2	26.4	70.75
Total		100

HPLC column: OJ-3R, Acetonitrile/Water = 70:30, 1.0 mL/min, 298 K, 220 nm.

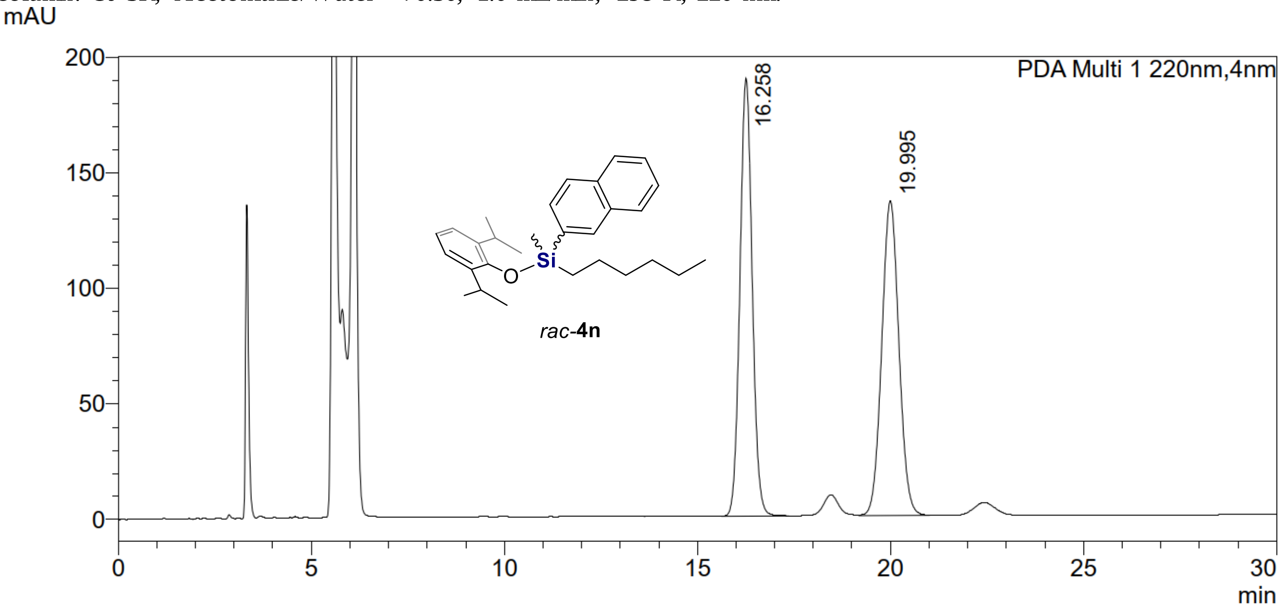

Peak #	t_R/min	% peak area
1	16.3	49.98
2	20.0	50.02
Total		100

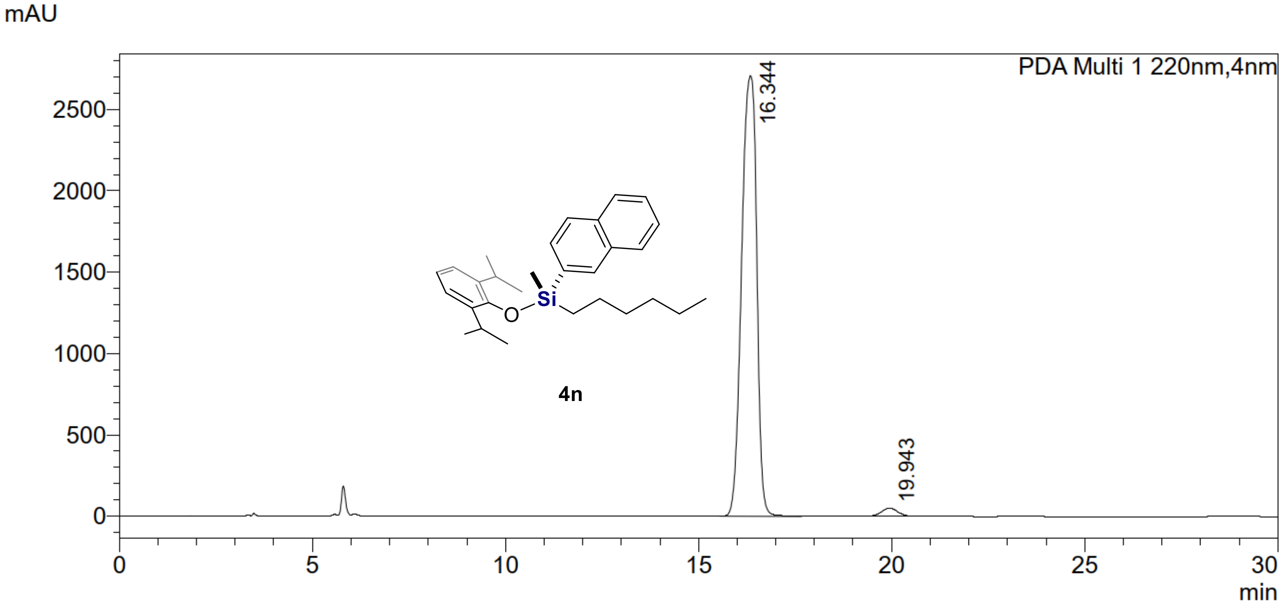

Peak #	t_R/min	% peak area
1	16.3	97.94
2	19.9	2.06
Total		100

HPLC column: OJ-3R, MeOH : H_2O = 80:20, 1 mL/min, 298 K, 220 nm.

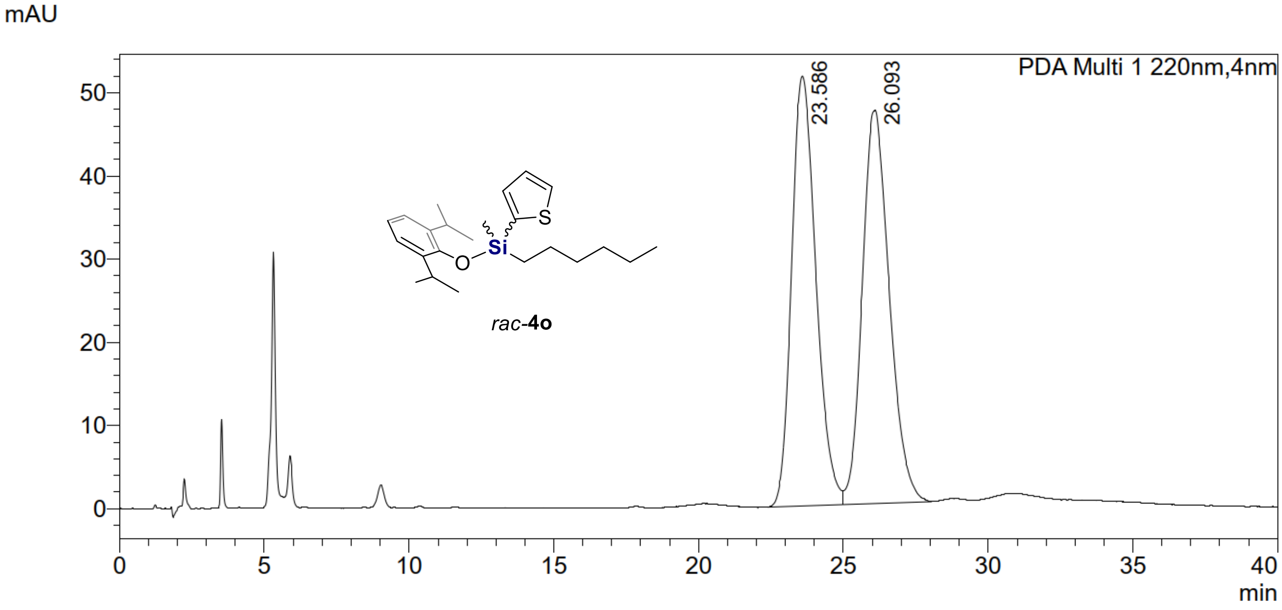

Peak #	t_R/min	% peak area
1	23.6	49.53
2	26.1	50.47
Total		100

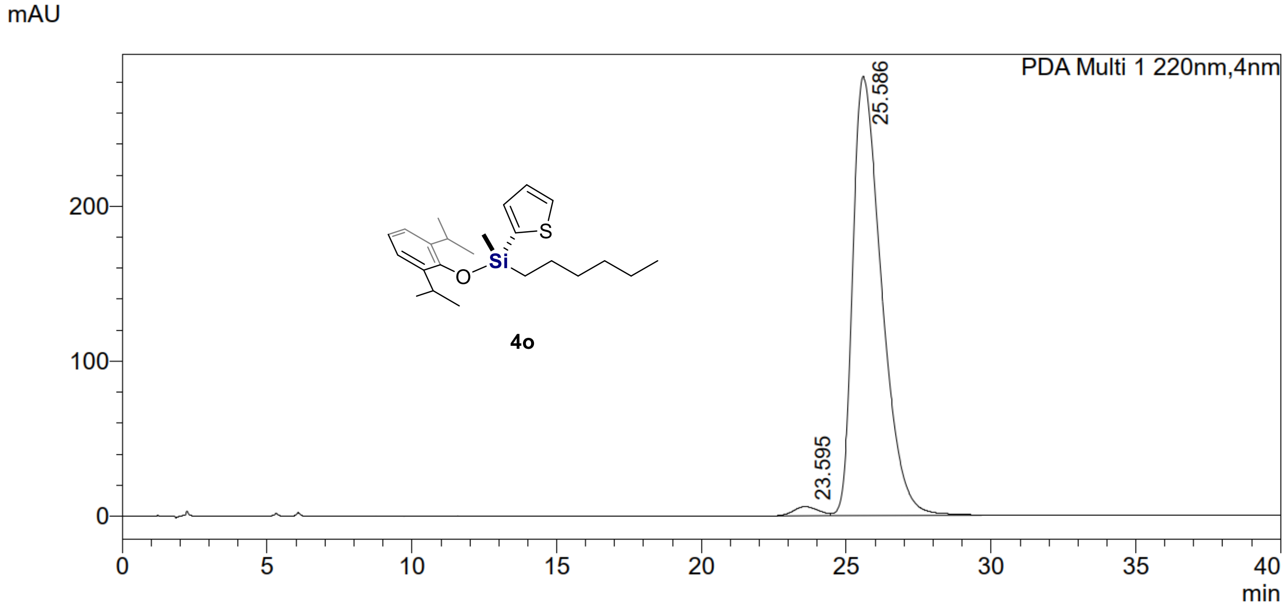

Peak #	t_R/min	% peak area
1	23.6	1.74
2	25.6	98.26
Total		100

HPLC column: OJ-3R, MeOH : H_2O = 80:20, 1 mL/min, 298 K, 220 nm.

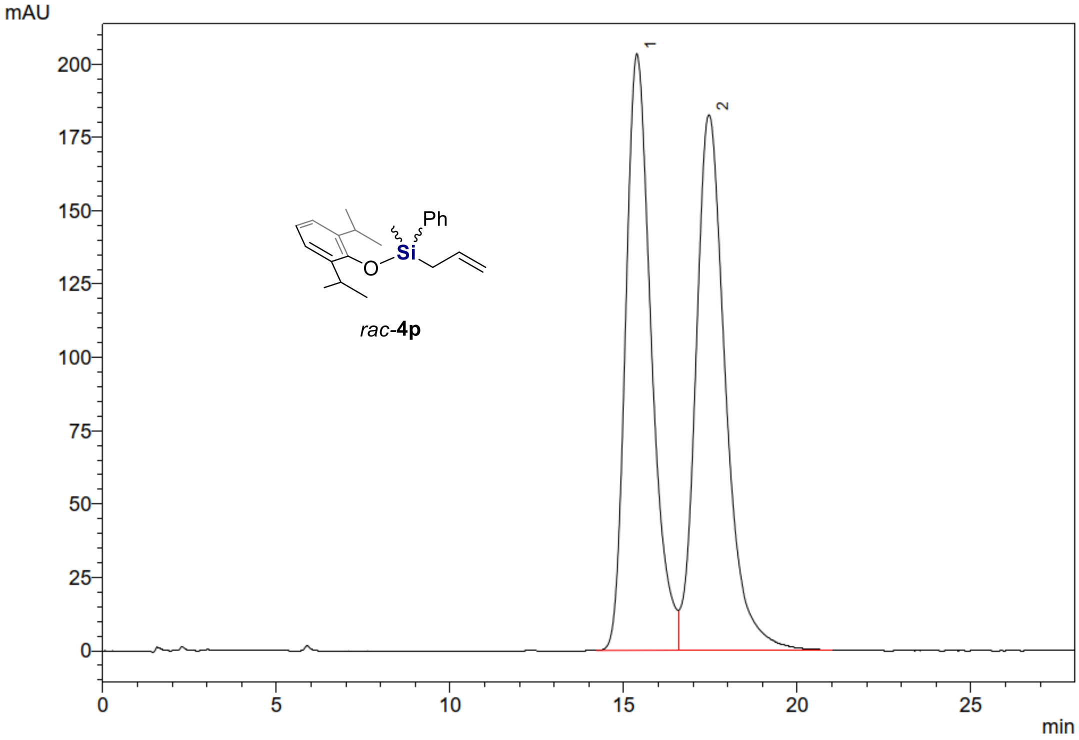

Peak #	t_R/min	% peak area
1	15.4	49.04
2	17.5	50.96
Total		100

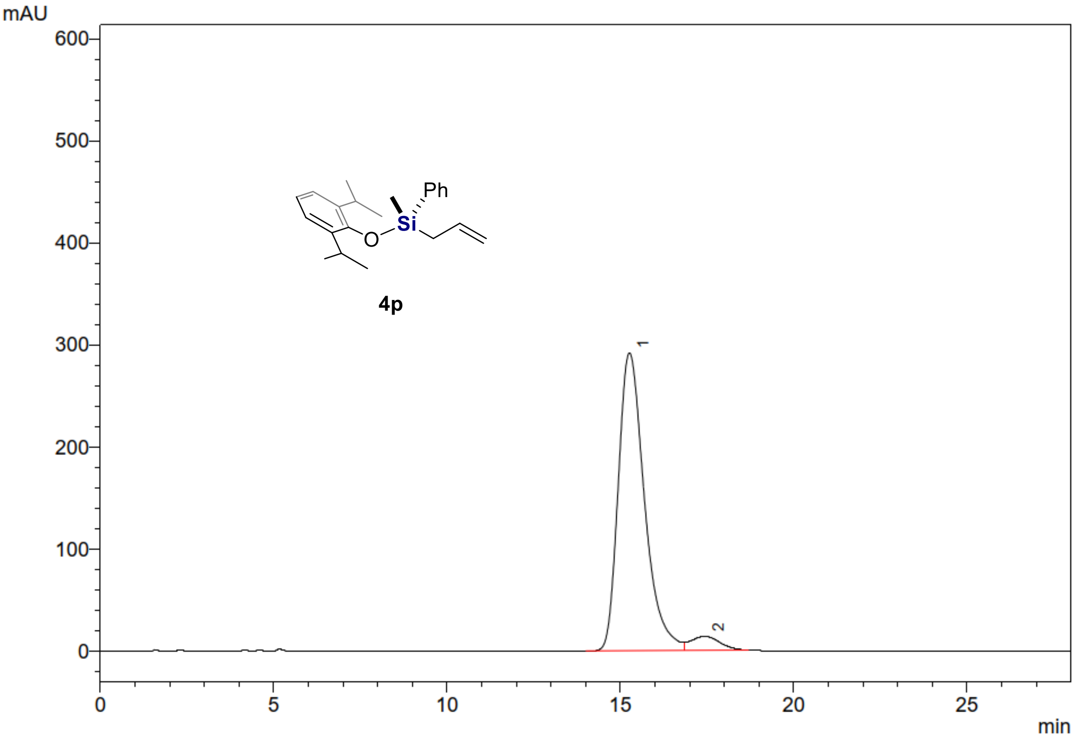

Peak #	t_R/min	% peak area
1	15.3	95.07
2	17.4	4.93
Total		100

HPLC column: OJ-3R, MeOH : H_2O = 80:20, 1 mL/min, 298 K, 220 nm.

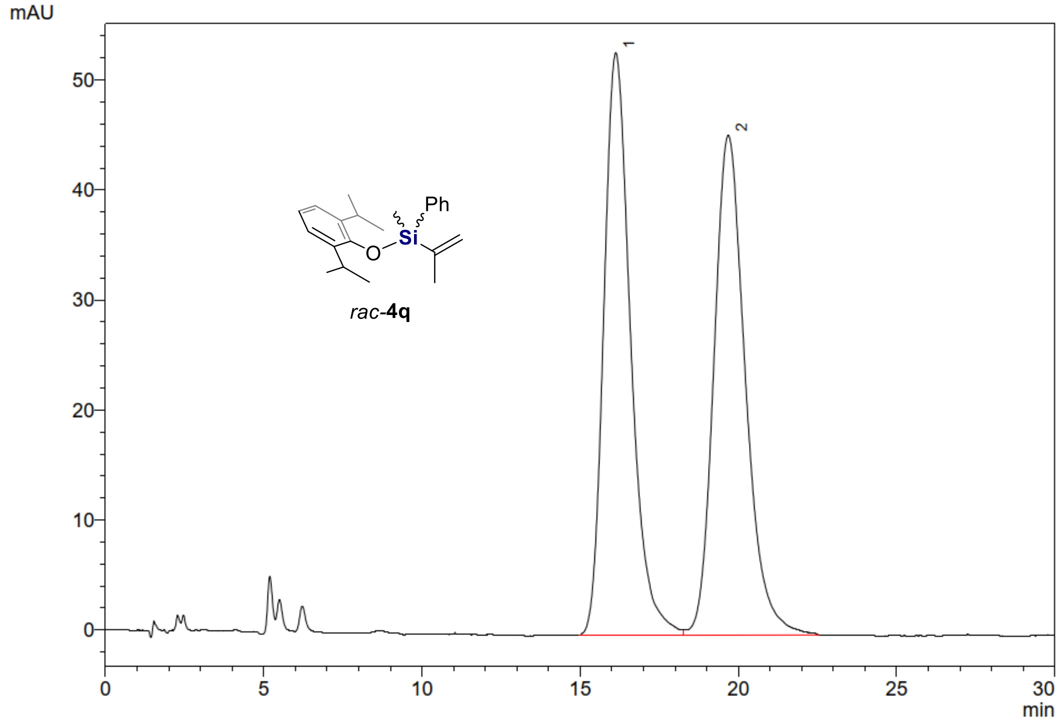

Peak #	t_R/min	% peak area
1	16.1	49.86
2	19.7	50.14
Total		100

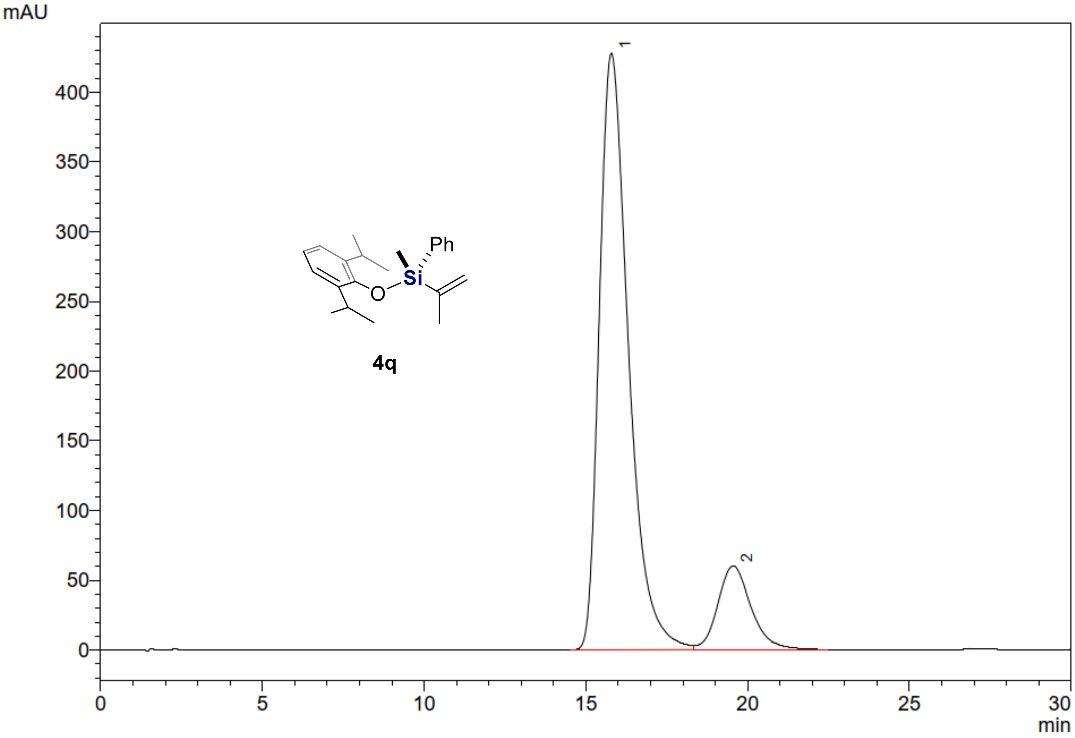

Peak #	t_R/min	% peak area
1	15.8	86.18
2	19.6	13.82
Total		100

HPLC column: OJ-3R, Acetonitrile/Water = 70:30, 1.0 mL/min, 298 K, 220 nm.

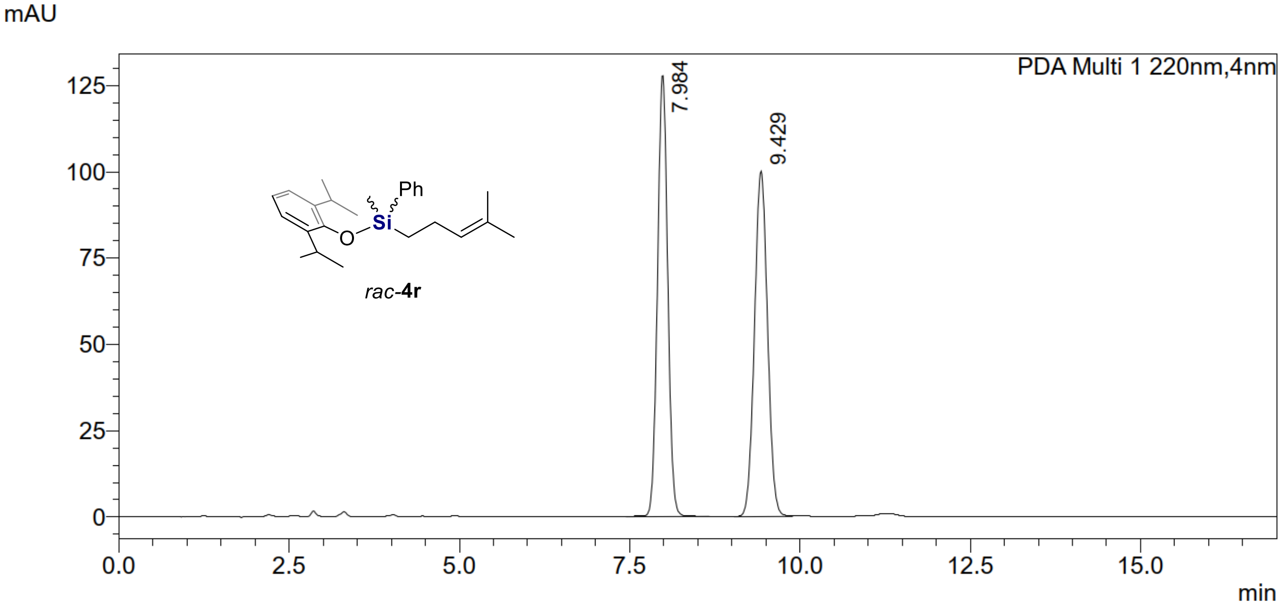

Peak #	t_R/min	% peak area
1	8.0	50.04
2	9.4	49.96
Total		100

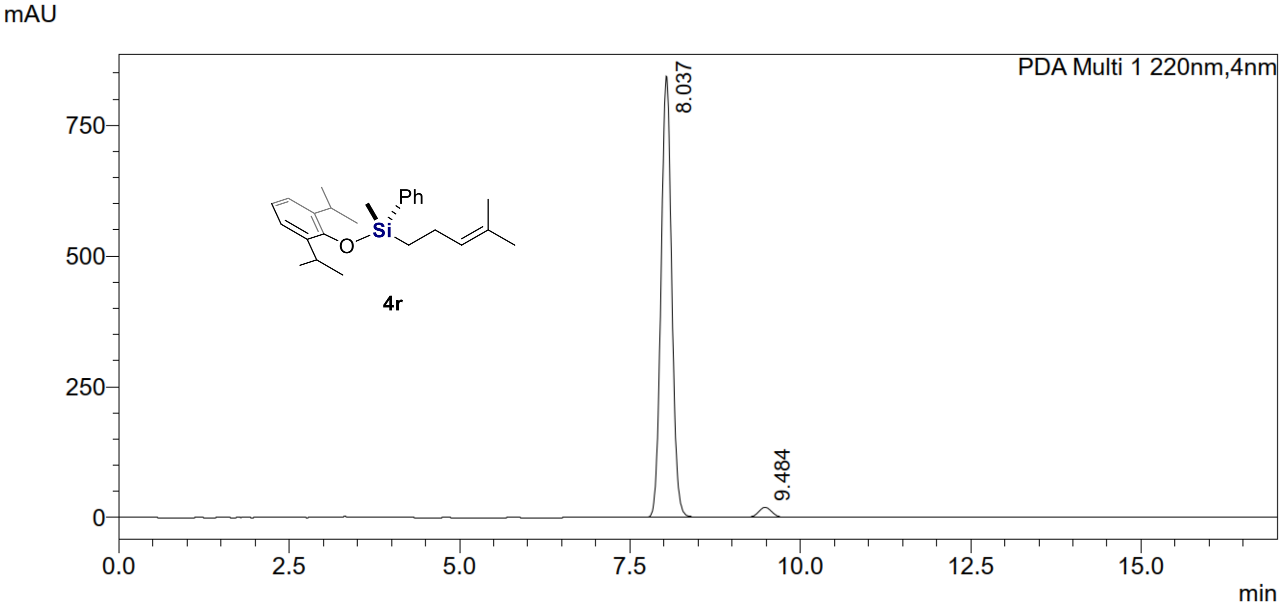

Peak #	t_R/min	% peak area
1	8.0	97.07
2	9.5	2.93
Total		100

HPLC column: OJ-3R, MeOH : H_2O = 80:20, 1 mL/min, 298 K, 220 nm.

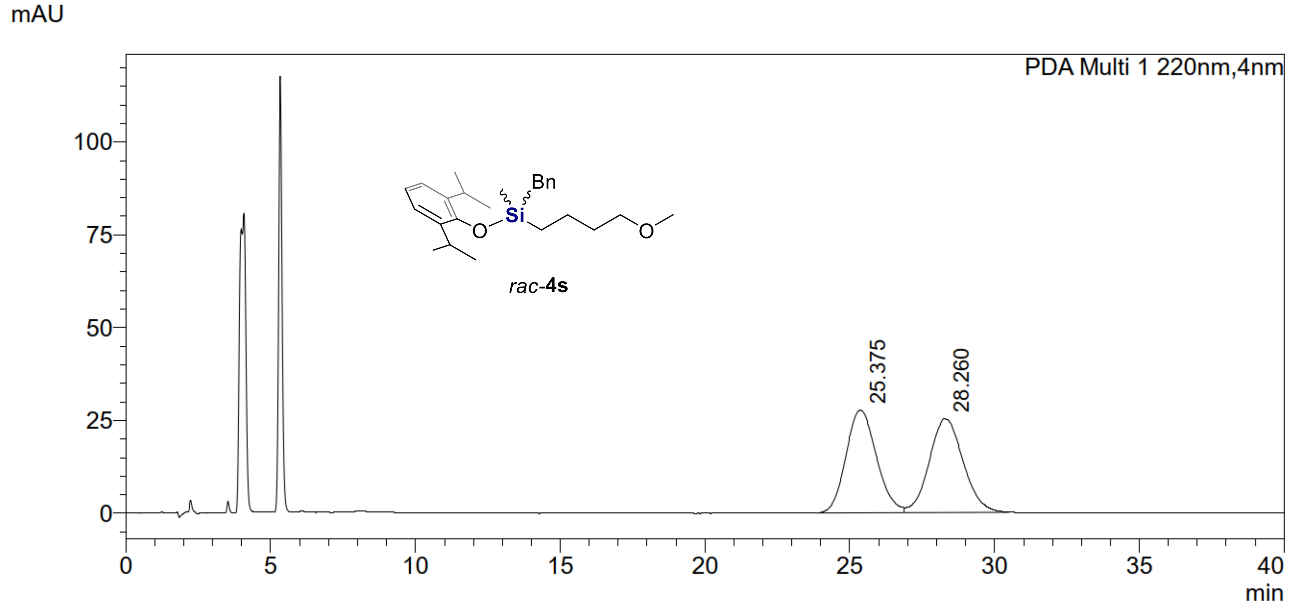

Peak #	t_R/min	% peak area
1	25.4	49.66
2	28.3	50.34
Total		100

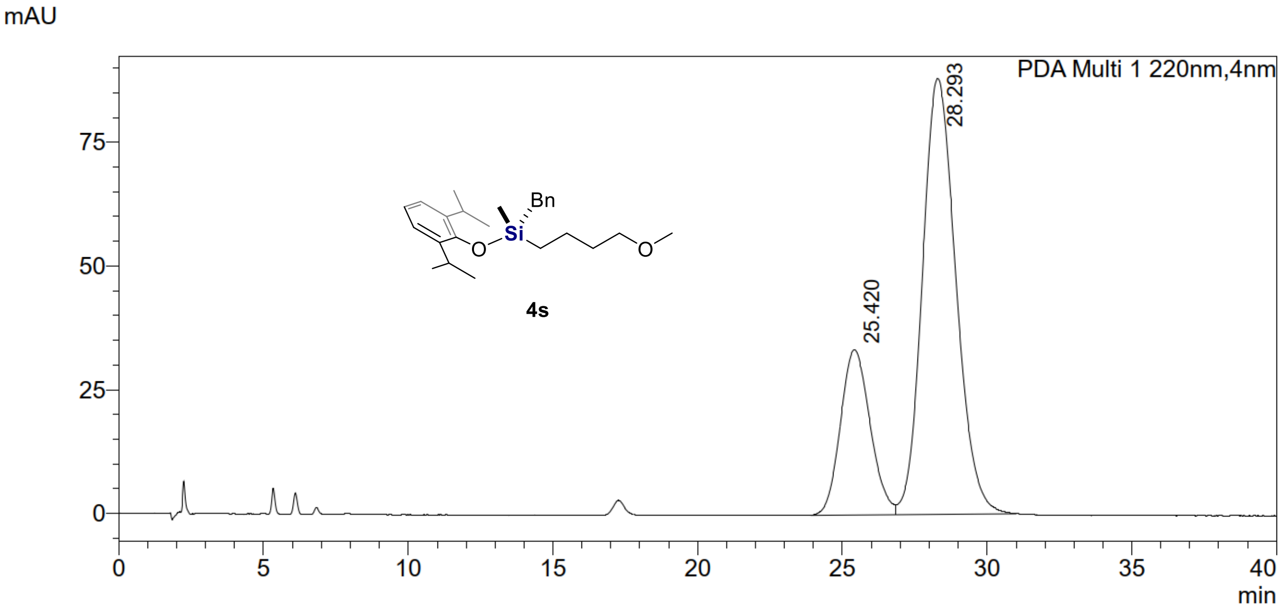

Peak #	t_R/min	% peak area
1	25.4	25.20
2	28.3	74.80
Total		100

Product **4a** from the reaction of **1u** and **2** was obtained with 98:2 e.r., the HPLC traces were shown below:
HPLC column: OJ-3R, MeOH : H_2O = 80:20, 1 mL/min, 298 K, 220 nm.

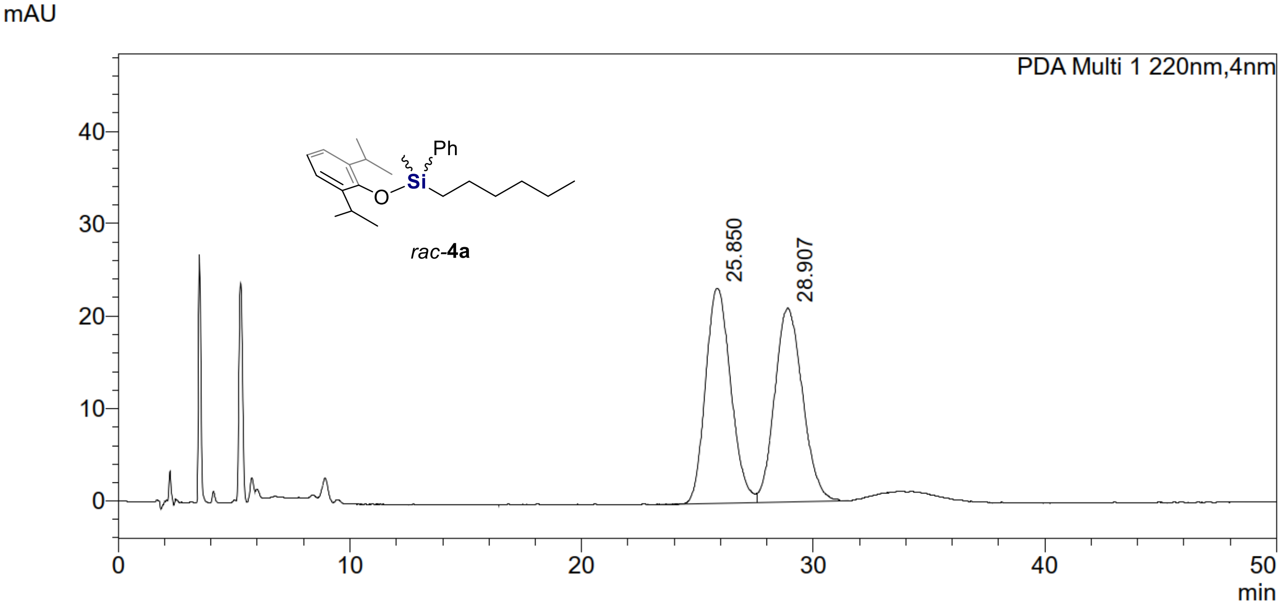

Peak #	t_R/min	% peak area
1	25.8	49.97
2	28.9	50.03
Total		100

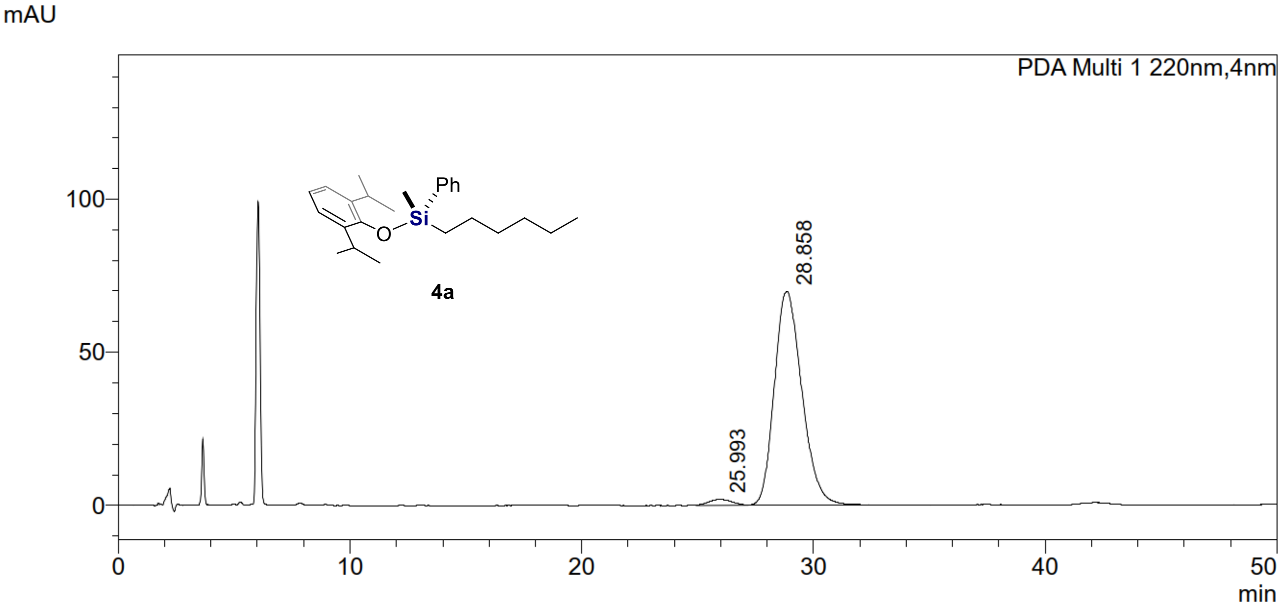

Peak #	t_R/min	% peak area
1	26.0	2.20
2	28.9	97.80
Total		100

HPLC column: IG-3, MeOH/Water = 80:20, 1.0 mL/min, 298 K, 220 nm.

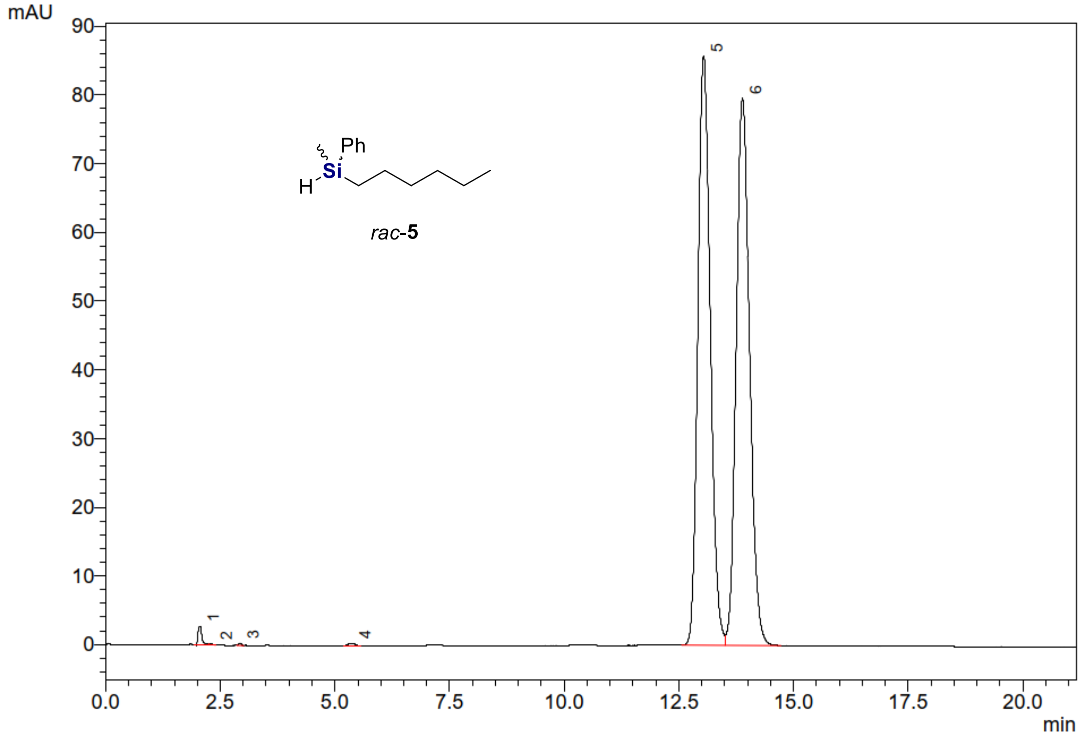

Peak #	t_R/min		% peak area
5	13.0		49.91
6	13.9		50.09
Total			100

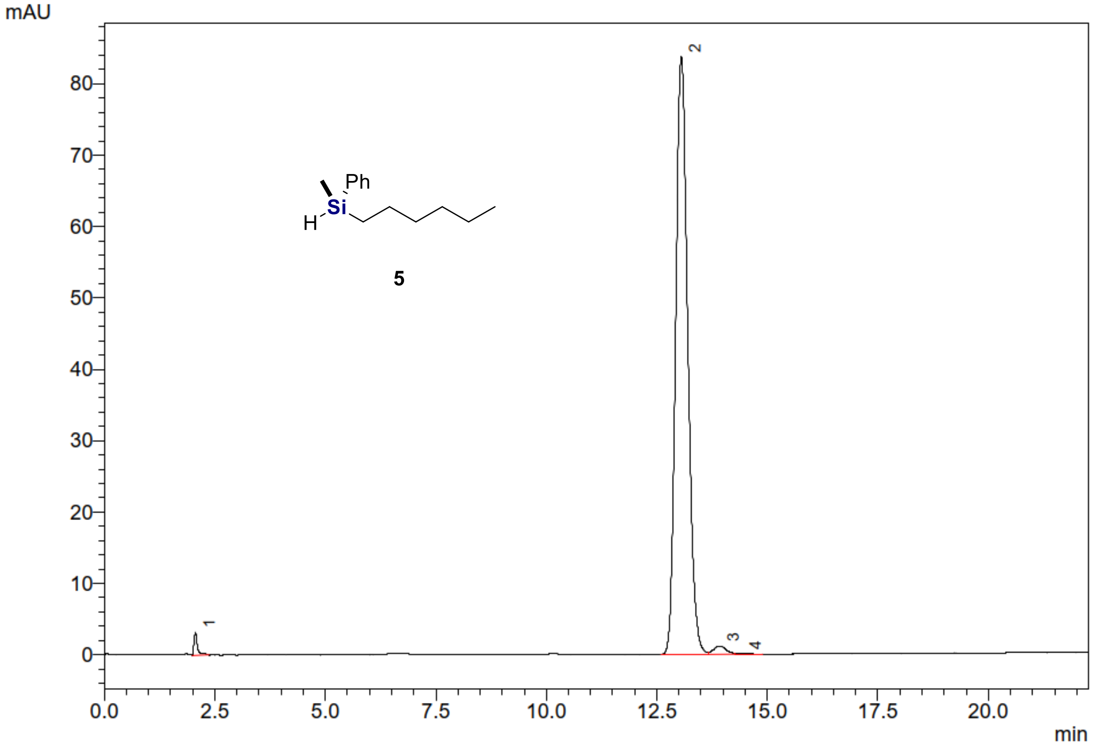

Peak #	t_R/min	% peak area
2	13.0	98.56
3	13.9	1.44
Total		100

LC traces of IDPi catalysts

Purity of the previously prepared IDPi Catalyst **3b** is 94.57%. Method: 50 mm Zorbax SB300-C8 3.5 µm, 4.6 mm i.D. Acetonitril / 1 % TFA = 80:20, 1 mL / min, 5.3 MPa, 308 K, UV, 254 nm.

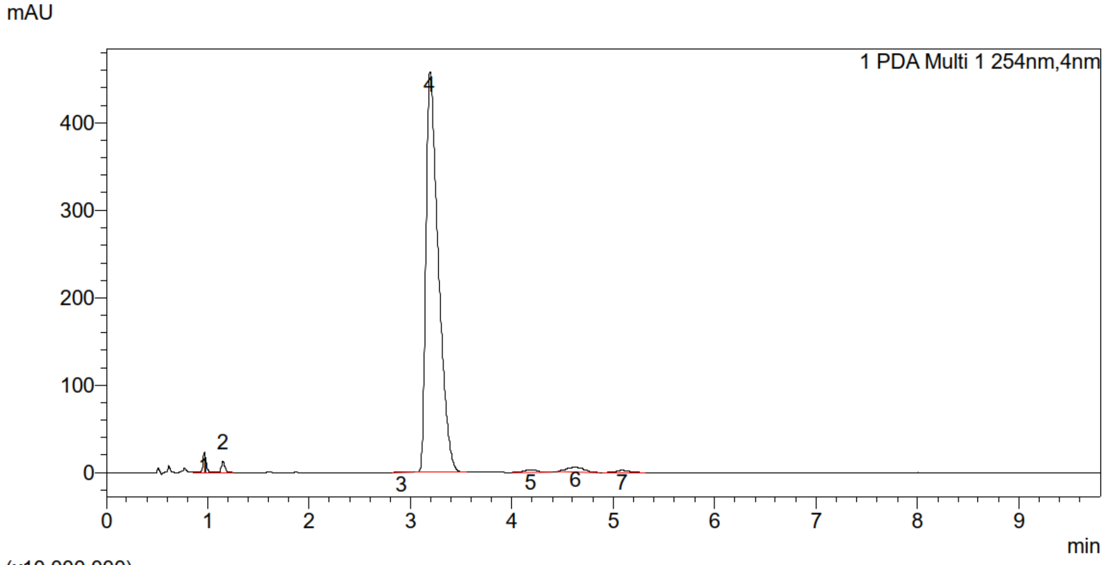

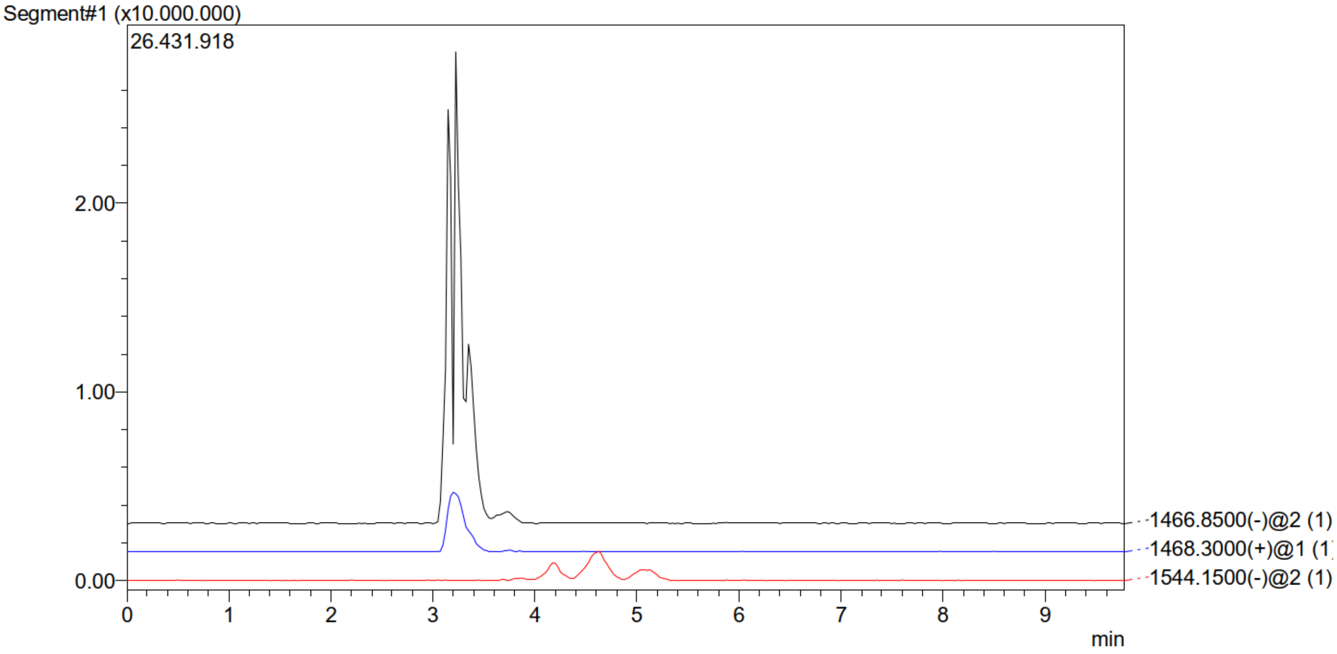

PDA Ch1 254nm

Peak#	Ret. Time	Area%	Name
1	0.97	1.30	
2	1.15	0.80	
3	2.91	0.16	
4	3.19	94.57	Product
5	4.19	0.70	
6	4.63	1.75	
7	5.09	0.72	
Total		100.00	

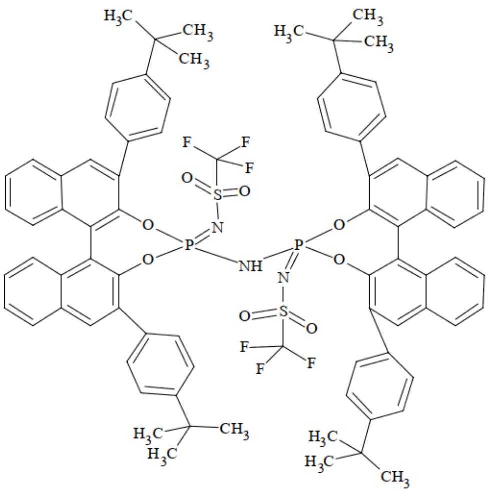

M = 1467

m/z 1466: M - H
m/z 1468: M + H

Retention Time: 3.265 min
Base Mass: 1468
DUIS Positive

Peak 3 positiv

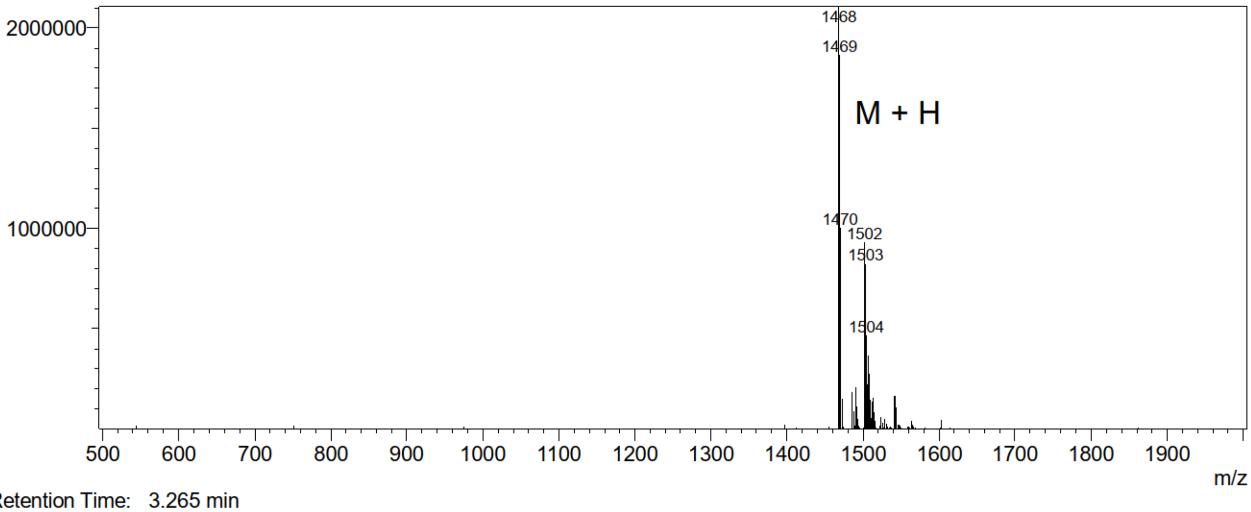

Retention Time: 3.265 min
Base Mass: 1467
DUIS Negative

Peak 3 negativ

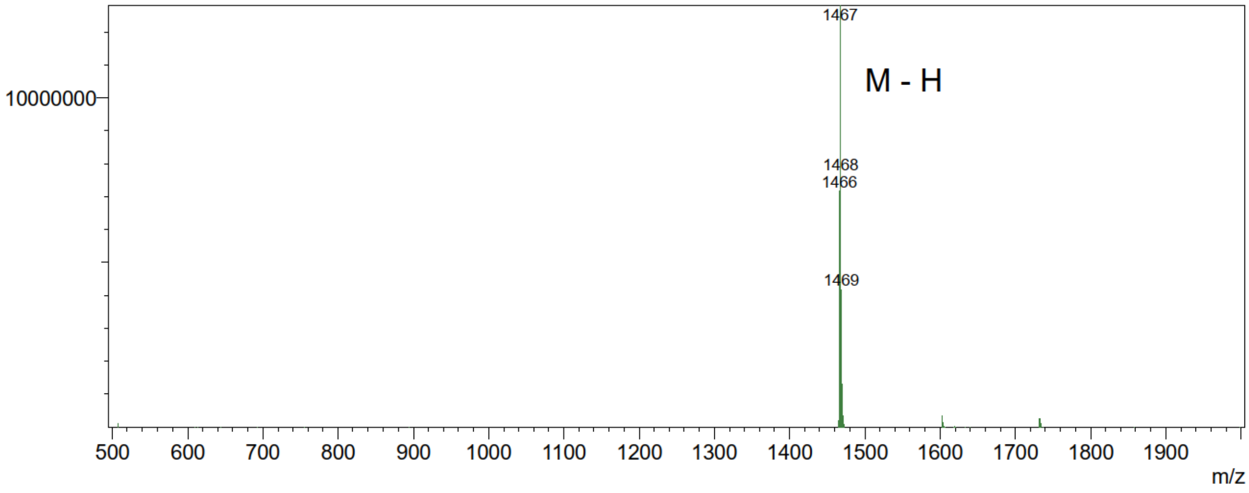

Purity of the freshly prepared IDPi Catalyst **3d** is 98.42%. Method: 50 mm Zorbax SB300-C8 3.5 µm, 4.6 mm i.D. Acetonitril / 1 % TFA = 80:20, 1 mL / min, 5.3 MPa, 308 K, UV, 254 nm.

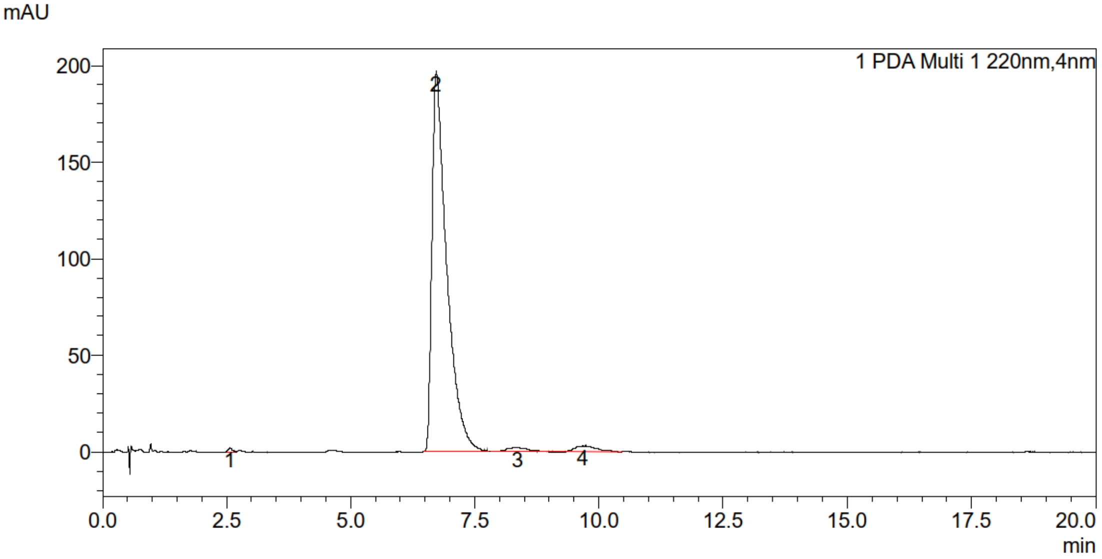

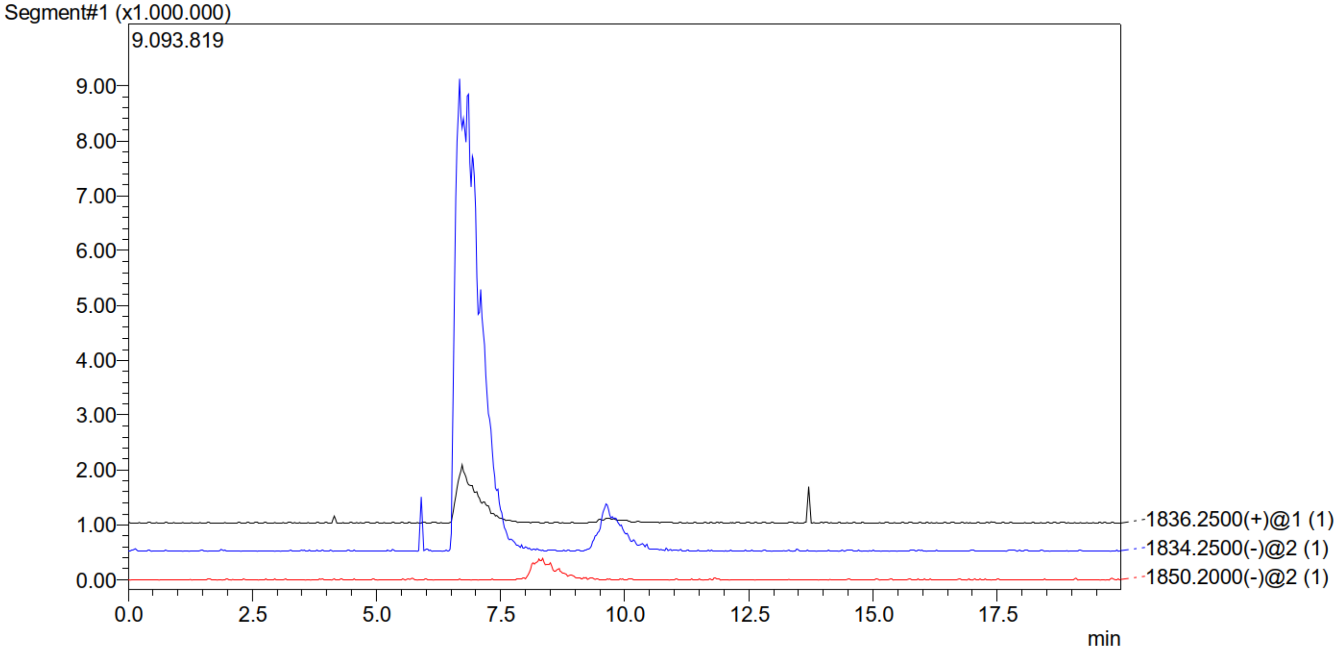

PDA Ch1 220nm

Peak#	Ret. Time	Area%	Name
1	2.57	0.25	
2	6.72	96.39	Product
3	8.36	1.33	
4	9.68	2.03	Isomer
Total		100.00	

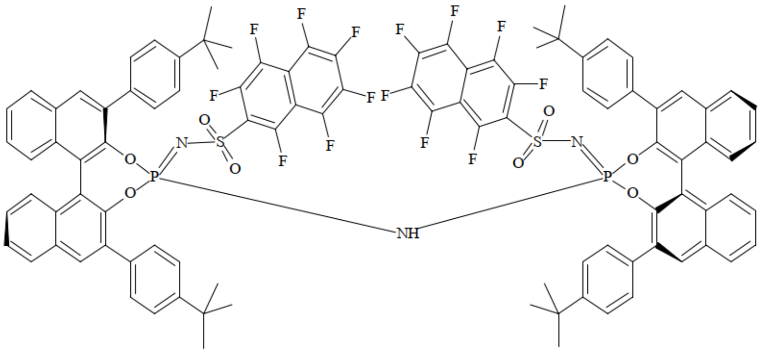

M = 1835

m/z 1836: M + H
m/z 1834: M - H

Retention Time: 6.807 min
Base Mass: 1837
DUIS Positive

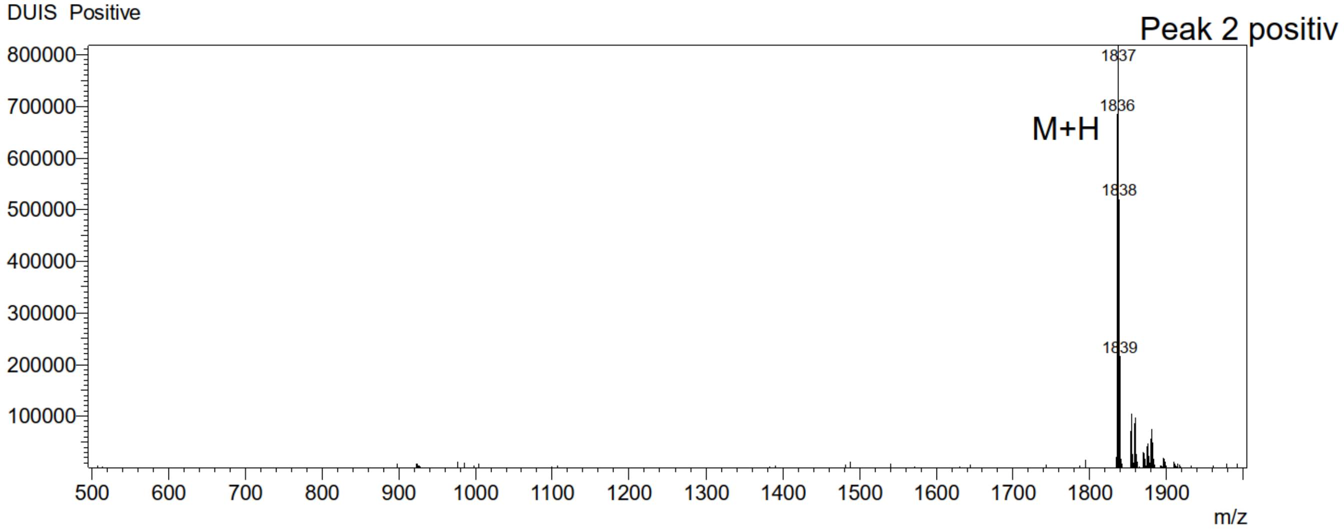

Retention Time: 6.807 min
Base Mass: 1835
DUIS Negative

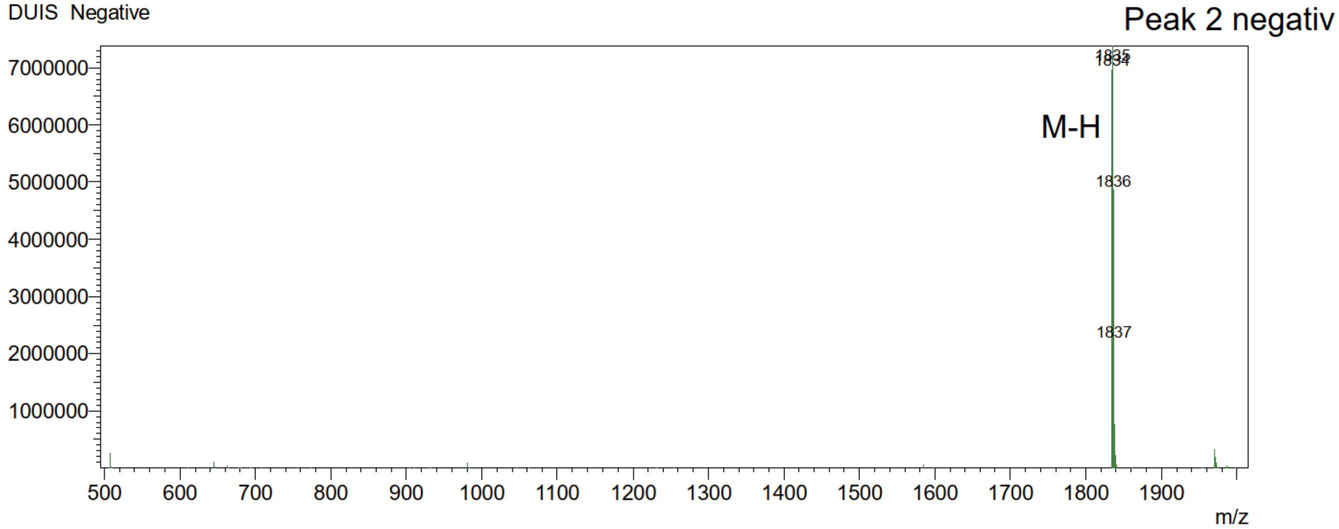

Retention Time: 9.646 min
Base Mass: 1834
DUIS Negative

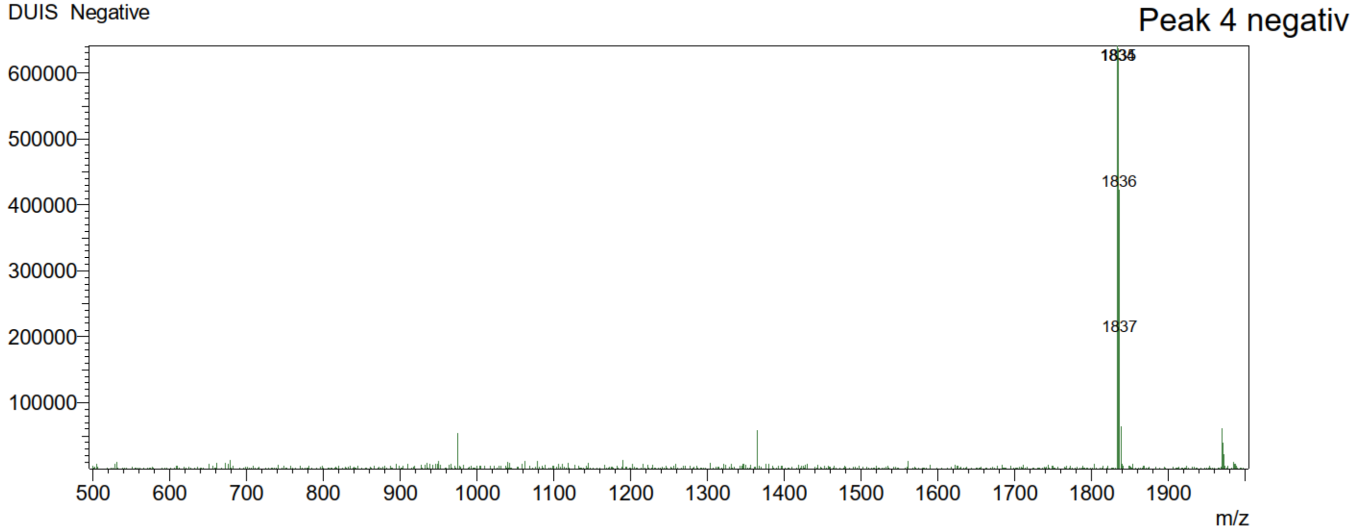

(R)-**1a** and (S)-**1a** were separated by prep LC, after separation, the pure enantiomers were again confirmed by HPLC analysis, the traces are shown below:

Method: HPLC column: AD-3R, MeOH:Water = 90:10, 1.0 mL/min, 298 K, 220 nm.

Racemate traces:

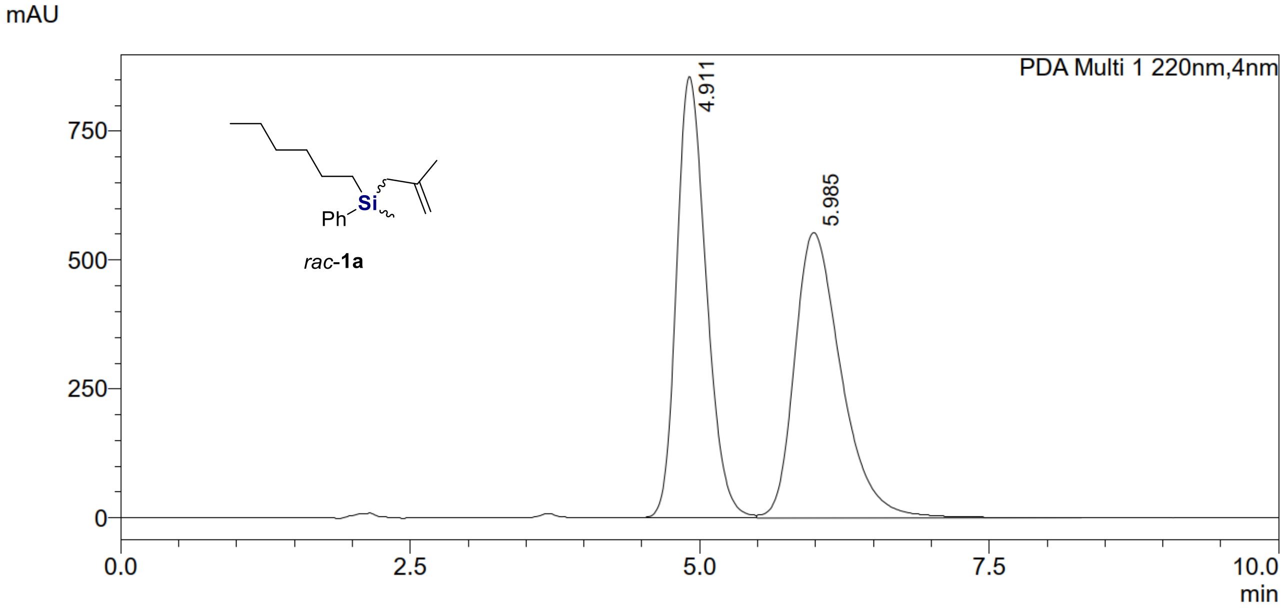

Peak #	t_R/min	% peak area
1	4.9	49.59
2	6.0	50.41
Total		100

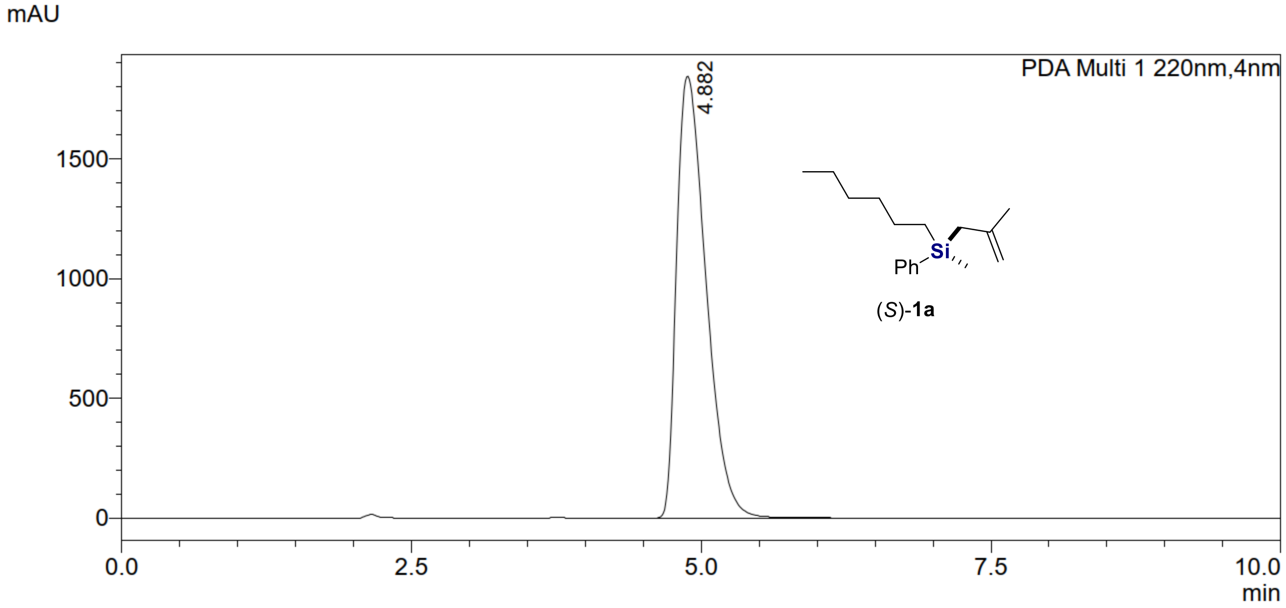

Peak #	t_R/min	% peak area
1	4.9	100
Total		100

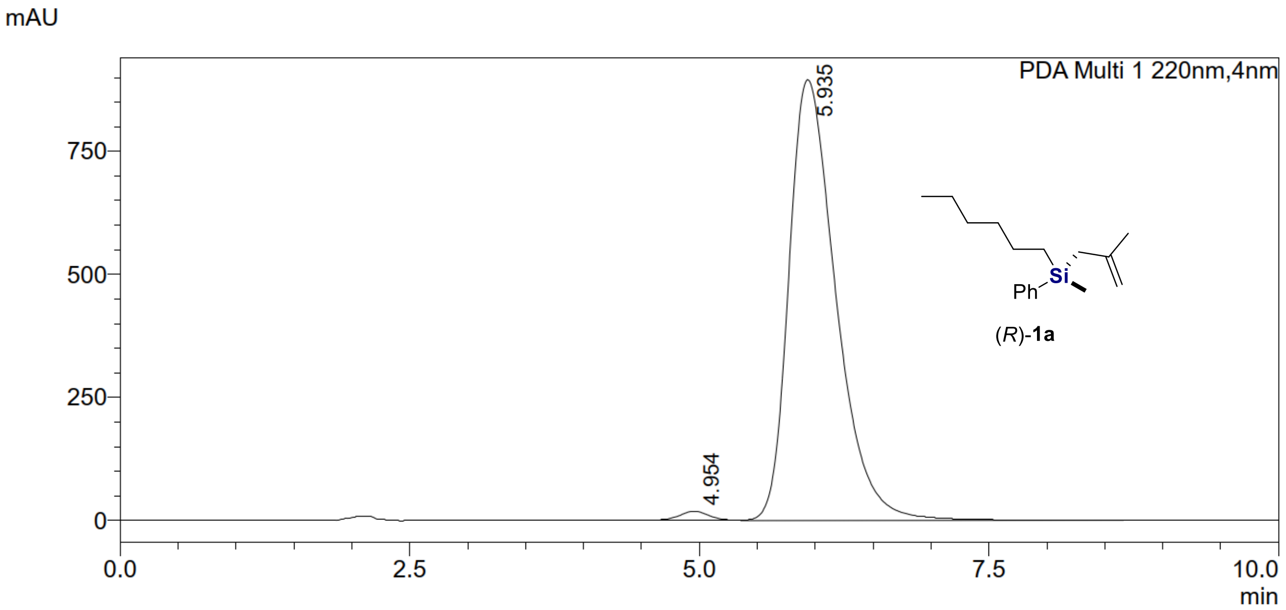

Peak #	t_R/min	% peak area
1	5.0	1.31
2	5.9	98.69
Total		100
